# Discovery of *N*-Hydroxypyridinedione-Based Inhibitors of HBV RNase H: Design, Synthesis, and Extended SAR Studies

**DOI:** 10.3390/ijms262010239

**Published:** 2025-10-21

**Authors:** Dea Chotzalli, Vasiliki Pardali, Holly M. Walden, Dimitrios Perivolaris, Dimitrios Moianos, Maria Makri, Antonios Drakopoulos, Erofili Giannakopoulou, Razia Tajwar, Molly E. Woodson, John E. Tavis, Grigoris Zoidis

**Affiliations:** 1 Department of Pharmacy, Division of Pharmaceutical Chemistry, School of Health Sciences, National and Kapodistrian University of Athens, Panepistimiopolis Zografou, 15771 Athens, Greece; chotzallidea@gmail.com (D.C.); pharmacy22pard@gmail.com (V.P.); perivolarisdimitrios@gmail.com (D.P.); moianosjim@gmail.com (D.M.); mariamakri1@hotmail.com (M.M.); evgian@pharm.uoa.gr (E.G.); 2 Molecular Microbiology and Immunology, Saint Louis University School of Medicine, Saint Louis, MO 63104, USA; holly.furby@slu.edu (H.M.W.); razia.tajwar@health.slu.edu (R.T.); molly.woodson@discovery.eurofinsus.com (M.E.W.); john.tavis@health.slu.edu (J.E.T.); 3 Saint Louis University Institute for Drug and Biotherapeutic Innovation, Saint Louis University, Saint Louis, MO 63104, USA; 4 Department of Chemistry and Molecular Biology, University of Gothenburg, 41296 Göteborg, Sweden; drakopoulosantonis@gmail.com

**Keywords:** Hepatitis B virus, Ribonuclease H, *N*-Hydroxypyridinediones, imines, oximes, structure-activity relationships, anti-HBV agents

## Abstract

Hepatitis B Virus (HBV) continues to pose a significant global health challenge, with over 254 million chronic infections and current therapies being non-curative, necessitating lifelong treatment. The HBV ribonuclease H (RNase H) is essential during HBV reverse transcription by cleaving the viral pregenomic RNA after it has been copied into the (−) polarity DNA strand, enabling the viral polymerase to synthesize the (+) DNA strand. Although RNase H inhibition terminates viral replication and thus viral infectiveness, its targeting as an HBV treatment is unexploited. Its catalytic site contains four carboxylates that bind to two Mg^2+^ ions essential for RNA hydrolysis. As part of our ongoing research on RNase H inhibitors, we developed 23 novel *N*-hydroxypyridinedione (HPD) analogues. Specifically, 17 HPD imines, 4 HPD oximes, 1 2,6-diamino-4-((substituted)oxy)pyrimidine 1-oxide derivative, and 1 barbituric acid analogue were designed, synthesized, and tested for their anti-HBV activity. The HPD derivatives could be docked in the RNase H active site to coordinate the two Mg^2+^ ions and effectively inhibited viral replication in cellular assays. The 50% effective concentration (EC_50_) values of these HPD compounds ranged from 0.5 to 73 μM, while the 50% cytotoxic concentration (CC_50_) values ranged from 15 to 100 μM, resulting in selectivity indexes (SIs) up to 112. Furthermore, the novel HPD derivatives exhibited favourable pharmacokinetic-relevant characteristics, including high cellular permeability, good aqueous solubility, and overall drug-like properties. These findings indicate that HPD imines and oximes possess substantial antiviral potency and selectivity against HBV, underscoring the potential of the HPD scaffold as a promising framework for the development of next-generation anti-HBV agents.

## 1. Introduction

Hepatitis B virus (HBV) is an enveloped DNA virus that replicates by reverse transcription and causes Hepatitis B [1]. According to the WHO, 254 million people are chronically infected by HBV and almost 1 million new infections are recorded annually [2]. HBV infections lead to 1.1 million deaths per year due to liver complications, including hepatocarcinoma, liver failure, and cirrhosis [3]. The Food and Drug Administration (FDA) has approved six nucleos (t) ide analogues (NA) and two interferon alpha derivatives (IFN-α) as antiviral medication for chronic hepatitis B infection. Nucleoside Reverse Transcription Inhibitors (entecavir, lamivudine, and telbivudine) and Nucleotide Reverse Transcription Inhibitors (adefovir, tenofovir, and alafenamide [NRTIs]) are orally administered drugs, showing robust antiviral activity and a high safety profile [4]. IFN-α and pegylated interferon α have both antiviral and immunomodulatory properties [5]. Therapy with NAs and IFN-α cause major reductions in viral titers, but they only very rarely eliminate the virus [6]. Consequently, NAs require a lifelong treatment, raising potential for emergence of drug resistance [7] and risking side effects from decades of treatment [8,9]. Pegylated interferon α is relatively rarely used due to serious side effects like flu-like symptoms, bone marrow suppression, fatigue, and depression [10]. Therefore, anti-HBV therapy research is focusing on new molecular targets that will likely need to be used in combinations to achieve functional cures in patients chronically infected with HBV [11,12].

The HBV virion endocytosis occurs following binding to the sodium taurocholate co-transporting polypeptide (NTCP) receptor. The relaxed circular DNA (rcDNA) from the virion is released to the cytoplasm and carried to the nucleus [5]. There, through a multiple-step process including removal of the viral polymerase (P) and RNA oligomers that are covalently attached to 5’ ends of the two DNA strands in the rcDNA, repair of the large gap in the viral (+) polarity DNA strand, and DNA ligation, it is converted to a circular minichromosome-like structure, the covalently closed circular DNA (cccDNA). The cccDNA is the template for viral transcription, including transcription of the pregenomic RNA (pgRNA) [13]. The pgRNA is either translated to make the hepatitis B core protein (HBc) and P, or it is reversely transcribed by P to make rcDNA. The newly synthesized rcDNA within viral capsids either replenishes cccDNA following transport to the nucleus, or it is enveloped and secreted as infectious virions [14]. Reverse transcription is catalyzed by two distinct enzymatic domains on P, the reverse transcriptase (RT) and ribonuclease H (RNase H) [15]. RT synthesizes the (−) polarity DNA and the RNase H concomitantly degrades the pgRNA after it is copied into DNA. The RT then synthesizes the (+) polarity DNA strand [16]. RNA hydrolysis by the RNase H occurs through a metal-chelation system of two Mg^2+^ ions coordinated by a DEDD motif in the active site of RNase H [17].

The absence of structural data for the RT and RNase H domains of HBV P has impeded development of potent inhibitors, particularly for the RNase H activity, that could contribute to a curative therapy for Hepatitis B [17]. We recently used AlphaFold [18] to predict the structure of HBV P and validated the model [15]. We have used this model to help guide the development of N-hydroxypyridinodione analogues as potent RNase H inhibitors using computational chemistry techniques [19,20]. The HPD pharmacophore is a six-membered ring with an oxygen trident which chelates the Mg^2+^ ions in the HBV RNase H active site. Almost all previously reported RNase H inhibitors are in the α-hydroxytropolone (αHT), N-hydroxyisoqinolinedione (HID), N-hydroxypyridinedione (HPD), and N-hydroxynapthyridinone (HNO) chemotypes [21], and they all preserve three electron donors (either O or N atoms) in appropriate positions to chelate the two Mg^2+^ ions in the enzyme’s catalytic site. Among them, HPDs have shown the most promising results in terms of potency and drug-like properties. HPDs exhibit significant selectivity against RNase H, with effective concentration 50% (EC_50_) ranging from the micromolar scale to 61 nM, and low cytotoxicity (high cytotoxic concentration 50% (CC_50_)) levels, leading to selectivity indexes (SI, CC_50_/EC_50_) >1000 for the best HPDs [22]. RNase H inhibitors cause accumulation of RNA:DNA heteroduplexes due to failure to degrade the pgRNA after it is copied into (−) polarity DNA, and this prevents synthesis of the viral (+) polarity DNA strand.

**Figure 1:**
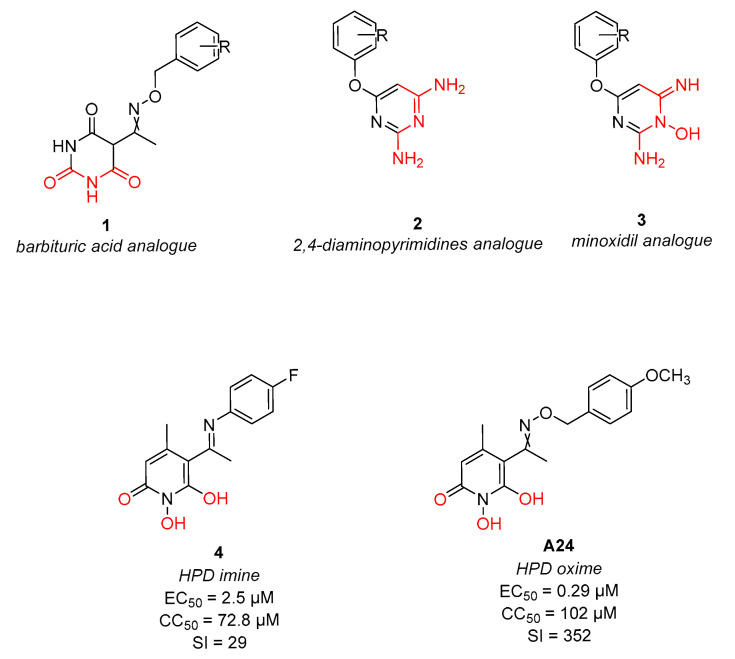
Chemical structures and biological activity of HBV RNase H inhibitors and related compounds from each chemotype. **1**, barbituric acid analogue; **2**, minoxidil analogue; **3**, 2,4-diaminopyrimidines analogue; **4**, *N*-Hydroxypyridinedione (HPD). **A24** is an approximately equimolar mixture of the E/Z isomers. EC_50_, effective concentration 50%; CC_50_, cytotoxic concentration 50%; SI, selectivity index (CC_50_/EC_50_). Red color shows metal chelating-moiety.

**Figure 2:**
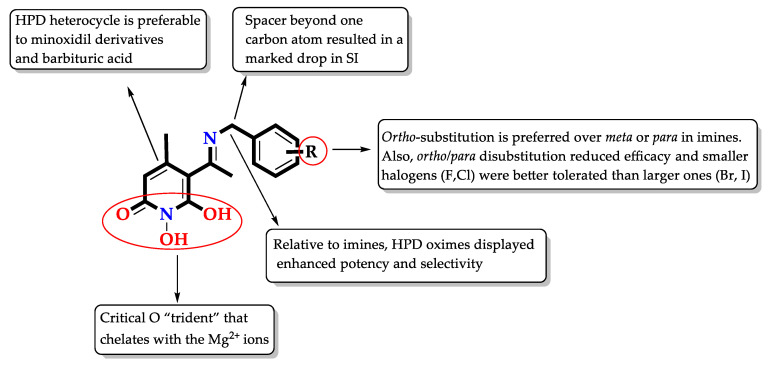
Schematic overview of the designed structural modifications and the corresponding structure–activity relationship (SAR) exploration strategy. Red color shows metal chelating-moiety.

In vitro experiments for cell-membrane permeability, plasma protein binding (PPB), and solubility in pH fluctuation from fasted gastric pH to blood pH, 1.6 to 7, respectively, have been conducted for HPDs [20,22], and extracellular, intracellular rcDNA, cccDNA, and HBsAg secretion were all robustly inhibited in cells infected with HBV that were treated with HPD, HNO, and αHT RNase H inhibitors [23]. Recently, it was shown that some HPD RNase H inhibitors have bimodal activity as they also decrease accumulation of intracellular capsids lacking viral nucleic acids (“empty” capsids). HPDs with this activity have been termed Capsid Assembly Modulators–Inhibitors (CAM-I) [24], as their effects on just empty capsids are distinct from the other CAM classes, CAM-A and CAM-E [25]. Thus, as a part of our ongoing research, we designed, synthesized, identified, and tested 23 compounds in total: 21 novel HPDs, 1 barbituric acid, and 1 minoxidil analogue, to further our understanding of structure–activity relationships for the HPDs and related molecules (Figure 1). A schematic overview of the designed structural modifications and the corresponding structure–activity relationships (SARs) is shown in Figure 2

## 2. Results and Discussion

### 2.1. Chemistry

**Scheme 1:**
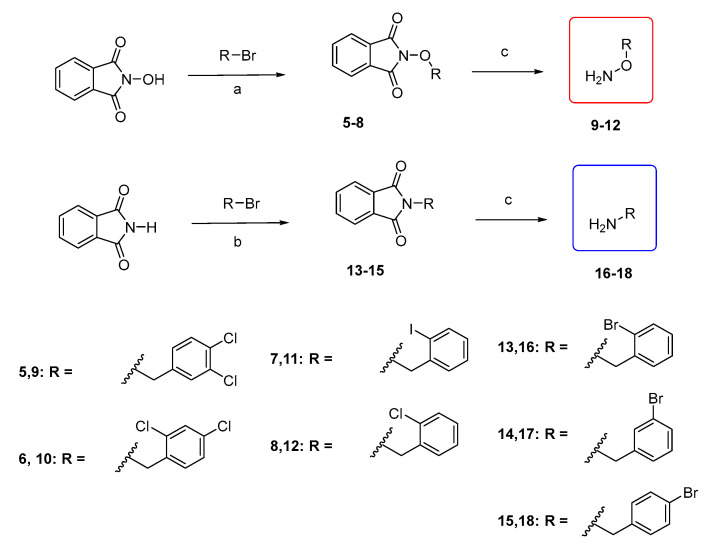
Synthesis of hydroxylamines **9**–**12** and benzylamines **16**–**18**. Reagents and conditions: (a) NaH, DMF, 0 °C to RT, overnight (43–93%); (b) K_2_CO_3_, DMF, 80 °C, Ar, overnight (52–91%) (c) H_2_NNH_2_, DCM or EtOH, RT, 1–16 h (57–99%).

**Scheme 2:**
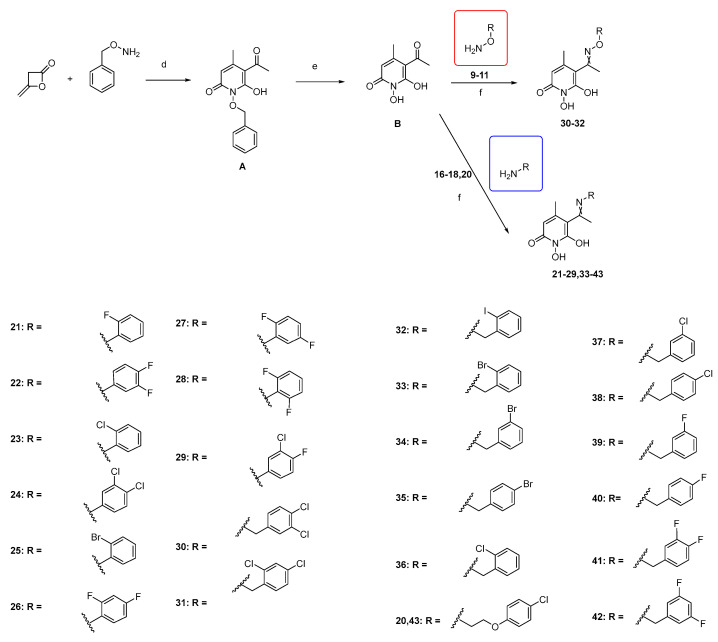
Synthesis of *N*-hydroxypyridinediones **21**–**43**. Reagents and conditions: (d) Et_3_N, dry toluene, 65 °C, 5 h (75%); (e) H_2_, Pd/C 10%, 40 psi, RT, MeOH, 30 min (98%); (f) abs. EtOH, 60 °C, activated 4Å molecular sieves, Ar, overnight (19.6–84%).

The novel HPD compounds were synthesized using a three-step synthetic approach (Scheme 1). The first step involved the Gabriel reaction of *N*-hydroxyphthalimide and of the phtalimide with benzyl bromides to afford compounds **5**–**8**, and **13**–**15**. Subsequently, the *O*-substituted *N*-hydroxyphthalimides **5**–**8** reacted with hydrazine monohydrate to afford the corresponding *O*-substituted hydroxylamines **9**–**12**. Also, the *N*-substituted *N*-hydroxyphthalimides **13**–**15** reacted with hydrazine monohydrate to afford the corresponding benzylamines **16**–**18**. In the final step (Scheme 2), the suitable hydroxylamines and benzylamines were condensed with 5-acetyl-1-(benzyloxy)-6-hydroxy-4-methylpyridin-2(1H)-one **B** in absolute ethanol at reflux to yield the HPD oximes **30**–**32** and imines **21**–**29**, **33**–**42**.

**Scheme 3:**
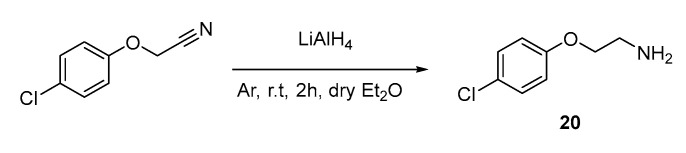
Synthesis of amine **20** (64%).

For the synthesis of amine **20**, a different synthetic procedure was employed (Scheme 3). Reduction of 2-(4-chlorophenoxy)acetonitrile with LiAlH_4,_ gave the corresponding amine. Then, it was followed the general procedure (Scheme 2), condensed with 5-acetyl-1-(benzyloxy)-6-hydroxy-4-methylpyridin-2(1*H*)-one **B** resulting in formation of compound **43**.

There are two geometrical isomers of oximes: *E* and *Z*. Since the two isomers of the final oxime derivatives were not separated in the current work, the compounds are formed as a mixture, with the ratio of the two isomers varying each time. One- and two-dimensional NMR tests verify the presence of the two isomers.

By refluxing a mixture of diketene (2 equiv.) and *O*-benzyl hydroxylamine (1 equiv.) in the presence of triethylamine (1 equiv.) in anhydrous toluene, we were able to synthesize the key intermediate 5-acetyl-1-(benzyloxy)-6-hydroxy-4-methylpyridin-2(1H)-one **A**. The target chemical **B** was then produced nearly quantitatively by catalytically hydrogenolyzing the benzyl group over 10% palladium on carbon (Scheme 2) [20].

A two-step synthetic procedure was used to create the analogue of minoxidil **44** (Scheme 3). The first step is a nucleophilic aromatic substitution of phenols with 6-chloro-2,4-diaminopyrimidine and NaH as a base. To obtain the final minoxidil analogues **44**, mCPBA is next used to oxidize the 6-substituted-2,4-diaminopyrimidines **19**.

**Scheme 4:**
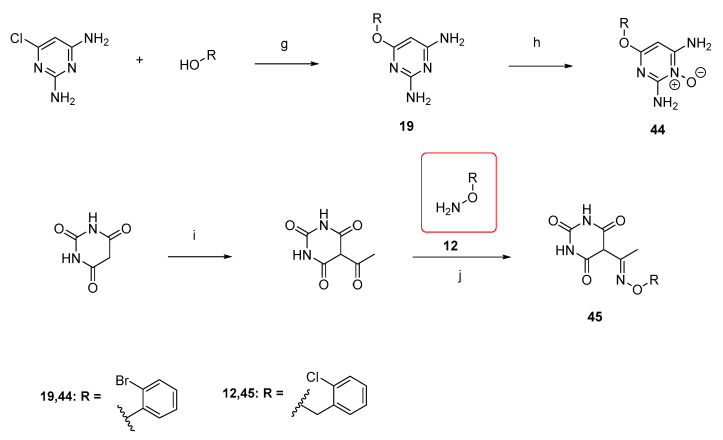
Synthesis of minoxidil analogue **44** and barbituric acid analogue **45**. Reagents and conditions: (g) NaH 60% *w*/*w*, neat, 150–180 °C, 3 h overnight (61%); (h) mCPBA, MeOH, 0 °C, 3 h overnight (50%); (i) (Ac)_2_O, H_2_SO_4_, 110 °C (reflux), 1.5 h (89%); (j) EtOH, RT, molecular sieves, reflux, 3 days (83%).

Analogue **45** of barbituric acid was synthesized via a two-step reaction process originating from barbituric acid (Scheme 4). Initially, barbituric acid underwent acetylation using acetic anhydride under reflux. Subsequently, 5-acetyl barbituric acid was combined with *O*-substituted hydroxylamines (which were prepared as previously outlined) through a coupling reaction in absolute ethanol in the presence of molecular sieves, resulting in the formation of compounds **45**.

### 2.2. Efficacy Against HBV Replication and Cytotoxicity

Starting with the most promising imine HPD previously reported, compound **54** [26] (EC_50_ = 2.5, CC_50_ = 72.8, SI = 28.7), we designed and synthesized 23 novel HPD imines and oximes featuring the same pharmacophore scaffold as the hit compound, but with different halogen substituents and varying numbers of carbon atoms in the linker. Additionally, Table 1 below presents the structurally related compounds **46**–**54**, which have been synthesized in the Zoidis lab and previously published [16,22,26]. These derivatives were included to allow for a more comprehensive discussion of the structure–activity relationships.

Compounds **21**–**29** had no linker; compounds **30**–**32**, which were oximes, had a 1-carbon linker, and compounds **33**–**42** were imines with a 1-carbon linker. Our goal was to further explore the SARs of the HPD scaffold to enhance the compounds’ potency, selectivity, and drug-like properties.

The EC_50_ values of the HPD compounds ranged from 0.5 to 73.7 μΜ, and the CC_50_ values ranged from 15.4 to 100 μΜ, resulting in SIs from 0.7 to 112.4 (Table 1).

Among the synthesized compounds, **22** and **31** were the most potent (EC_50s_ of 0.9 and 0.5 μΜ). Compound **22** is characterized by a 3,4-difluoro substitution in the aromatic ring. Based on this compound, we synthesized compound **41** with a 1-carbon linker, which reduced activity against HBV.

Next, we synthesized imines with a 1-carbon linker, compounds **33**–**42**. Among different halogen substitutions, it was shown that *ortho*-substitution is more favourable than *meta* or *para* substitution, as we can see in compounds **32**–**35** and **36**–**38**. Additionally, the *ortho*-chloro substitution resulted in better SI values than *ortho*-bromo substitution (compound **36** vs. **33**).

Compounds **30**–**32** exhibited greater potency compared to the others due to the oxime group, as mentioned in previous works. This is due to the O group, which forms H bonds with the amino acids of the active site. Among the compounds, compound **31** showed the highest potency, resulting in SI = 111.9.

Overall, incorporation of the oxime moiety led to a pronounced enhancement in potency (Table 1), with compound **39** (SI = 0.7) and compound **53** (SI = 1231) representing the most compelling examples of this trend.

Oxime analogues **46**–**48** and **49**–**51** were more potent than imine analogues **33**–**35** and **36**–**38**, respectively, with the same substitution. Moreover, analogue **47** with a *meta* bromo substitution had greater activity than compounds **46** and **48**. Also, in compounds **49**–**51**, *ortho* substitution was the most promising substitution, as in imines **36**–**38**.

*Ortho*-chloro (compound **49**) and *ortho*-fluoro (compound **52**) substitutions have a greater activity than *ortho*-bromo (compound **46**) and *ortho*-iodo (compound **32**) substitutions. Thus, as far as *ortho*-substitution is concerned, when the atomic radius of the halogen is reduced, we observe higher SI values, independently of oximes or imines analogues and the presence of a spacer. For *meta* and *para* substitutions in imines, the atomic radius of halogen does not have a significant impact on SIs. All halogen mono-substituted imines with a spacer or not demonstrate low SIs ranging from 0.7 to 3.1.

Comparing fluoro-substituted imine analogues 2,4-fluoro (compound **26**), 2,5-fluoro (compound **27**), and 2,6-fluoro (compound **28**) to ortho-fluoro, we observe a reduction in potency almost by half.

In regard to disubstituted aromatic moieties, in combination of different halogens, we came to the conclusion that this brings on a dramatic drop in SIs, mainly due to cytotoxicity increase.

Furthermore, the addition of another spacer seems not to be tolerated, and the displacement of the O to a different position other than next to N, resulting in phenyloxyethyl imine analogues, decreases its potency by more than 100-fold down. (Compound **43**, SI = 1.8 vs. compound **51**, SI = 130)

Overall, HPDs featuring halogen substitutions at the 2’ position of the aromatic moiety were the most effective HBV RNase H inhibitors. Compounds that possess halogen substitutions on the aromatic side chain and a short linker (1-carbon after the oxime group) between the HPD core pharmacophore ring and the side aromatic moiety exhibit the optimal combination of antiviral activity, minimal cytotoxicity, and favourable SI values.

As it has been reported previously, modification of the primary pharmacophore with a minoxidil analogue is not capable of chelating the Mg^2+^ ions at the enzyme’s catalytic site. As a result, compound **44** with the 2-bromobenzyl-substitution, is inactive against HBV, compared to compound **33**, respectively. This may be due to the reduced electronegativity of nitrogen compared to oxygen in the HPD heteroatom.

In compound **45**, where the HPD ring was replaced with the pharmacophore ring of barbituric acid, resulted in lack of inhibition. This is probably because the *N*-hydroxyimide group misses the middle O atom, which is essential for coordinating metal ions within the RNase H active site, as it has been reported in previous works.

Finally, we tested the most active compounds (**22**) for their ability to inhibit recombinant, purified RNase H in biochemical assays to ensure that this class of molecules are acting as RNase H inhibitors, as do other members of the HPD chemotype [16,22]. HBV RNase H carrying the P790L mutation that is needed to stabilize the recombinant enzyme was purified from *E. coli*, and RNase H assays in which an RNA:DNA heteroduplex was incubated with the recombinant enzyme were performed. The RNA strand is FAM-labelled, and the DNA strand carries an IowaBlack quencher. Cleavage of the RNA strand releases the FAM moiety from quenching, causing an increase in fluorescence. Performance limitations with the enzyme limit this assay to just qualitative interpretation [16]. Compounds **22** showed a dose-dependent suppression of RNase H activity, with suppressing activity by ~50% at 100 µM (cf. SI p. 74). These findings indicate that compound **22** can directly inhibit the HBV RNase H.

### 2.3. Compound Solubility and Apparent Passive Permeability

All of the compounds **23**–**45** were highly soluble (solubility limit ≥ 100 μM) in conditions reflecting tissue culture media (pH 7.4) (Table 2). High solubility indicates that these compounds were able to stay in solution at a physiologically relevant pH, which is an essential prerequisite for them to be delivered to the site of action.

Additionally, 22 of the 23 novel compounds tested in parallel artificial membrane assays in conditions reflecting tissue culture media (pH 7.4) had high apparent passive permeability by the industry standard cutoff (≥1 × 10^−6^ cm/s). These data indicate that the compounds may be passively permeable across biological membranes.

### 2.4. Investigation of Oxime Diastereomeric Preference (**30**–**32**)

To rationalize the experimental SAR observed in oxime analogues **30**–**32**, we investigated the relative stability and population of their *E/Z* diastereomers using systematic conformational searches and Boltzmann population analysis. Experimental NMR spectra revealed that compound **31** exists exclusively as a single diastereomer, while **30** and **32** are present as mixtures with *E/Z* ratios of ~2.5–3:1 and ~1.8–2:1, respectively (cf. SI p. 49). However, the NMR spectra did not allow unambiguous assignment of which diastereomer was predominant.

Systematic conformational searches performed via Molecular Mechanics (OPLS3e, implicit DMSO) consistently identified the *E* diastereomer as the global minimum for all three compounds (cf. Figures S1–S3). To quantify equilibrium populations, we computed Boltzmann weights for all conformers and obtained normalized populations (cf. Tables S1–S3; Materials and Methods 3.8). The fractions of the conformational ensembles were defined as the sums of populations of *E* and *Z* conformers, *f*_E_ and *f*_Z_, respectively.

The resulting normalized Boltzmann populations (cf. Table 3 and Table S4) showed that compounds **30** and **31** strongly favour the *E* diastereomer (*f*_E_ = 0.87 and *f*_E_ = 0.86, respectively), whereas compound **32** exhibited a lower but still substantial *E* population (*f*_E_ = 0.74). Importantly, sensitivity tests involving systematic energy shifts of ±1 and ±2 kJ·mol^−1^ did not alter these values significantly (<0.001 absolute difference), confirming that the results are robust to typical force-field uncertainties (cf. Table S4). In addition, cumulative population curves further demonstrated that for **30** and **31** the ensemble is rapidly dominated by low-energy *E* conformers, while **32** includes a larger proportion of low-lying *Z* conformers, accounting for its reduced *f*_E_ (cf. Figure S4). Although **31** was found to be purely one diastereomer via NMR, the calculated *f*_E_ of **30** appears slightly larger (0.87 vs. 0.86). The difference is well within the method uncertainty of force-field modelling (~1.5%) and it arises because **31** has more *Z* conformers within 5 kJ from the global minimum than **30** (cf. Table S4 and Figure S4), diluting its *E* fraction slightly, even though experimentally it appears pure *E*. It is reasonable that NMR may not detect a very small *Z* population (below detection threshold), while the systematic conformational search calculation should include all conformers down to higher energies.

Subsequently, these populations were related to the biological activity of the compounds. The experimental EC_50_ values are 1.77 ± 0.7 µM for **30**, 0.53 ± 0.3 µM for **31**, and 1.77 ± 0.5 µM for **32** (cf. Table 2 and Table 3). For each mixture, the implied intrinsic potency of the *E* isomer, EC_50_^E^, was calculated from the observed mixture activity EC_50_^mix^ and the fraction *f*_E_ (cf. Table 3, Materials and Methods). This analysis showed that the intrinsic activities of *E* in **30** and **32** are comparable to that of pure **31**, suggesting that the observed weaker potencies of **30** and **32** arise primarily from dilution by inactive *Z* rather than from reduced affinity of *E* itself. Conversely, predicted mixture potencies EC_50_^pred^ for **30**, **32** were calculated using **31** (pure *E*) as a reference EC_50_^Ref_E^ and the respective *f*_E_ values (cf. Table 3, Materials and Methods). For both **30** and **32**, the predicted mixture activities closely matched the experimental values within error, further validating the model (cf. Table 3).

Taken together, these results coherently suggest that the *E* diastereomer is the active form of the oximes **30**, **31**, **32**, consistent with its identification as the global minimum in conformational searches. The reduced potencies of **30** and **32** are quantitatively explained by their equilibrium mixtures of *E* and *Z* while the exclusive presence of *E* in compound **31** is also explained. The excellent agreement between computational populations, NMR ratios, and EC_50_ data highlights how conformational and diastereomeric equilibria can directly shape observed biological activity.

### 2.5. Molecular Docking

**Figure 3:**
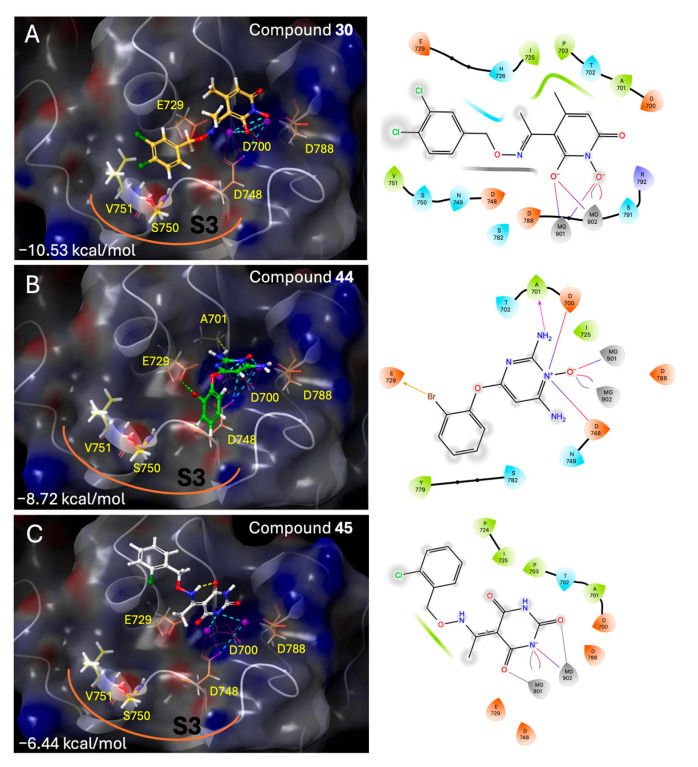
Compounds **30**, **44**, and **45** docked into the RNase H active site of HBV P. In panel (**A**), side chain compound **30** (orange) placed in S3 pocket and solvent exposed. In panel (**B**), compound **44** (green) is making halogen bond with only E729 (orange), H-bond with A701 (yellow), and salt bridge with D700 (orange). In panel (**C**), the side chain of compound **45** is surface exposed. Surface diagram (**left**), ligand interaction diagram (**right**), Mg^2+^ ions (light pik), catalytic residues (orange).

**Figure 4:**
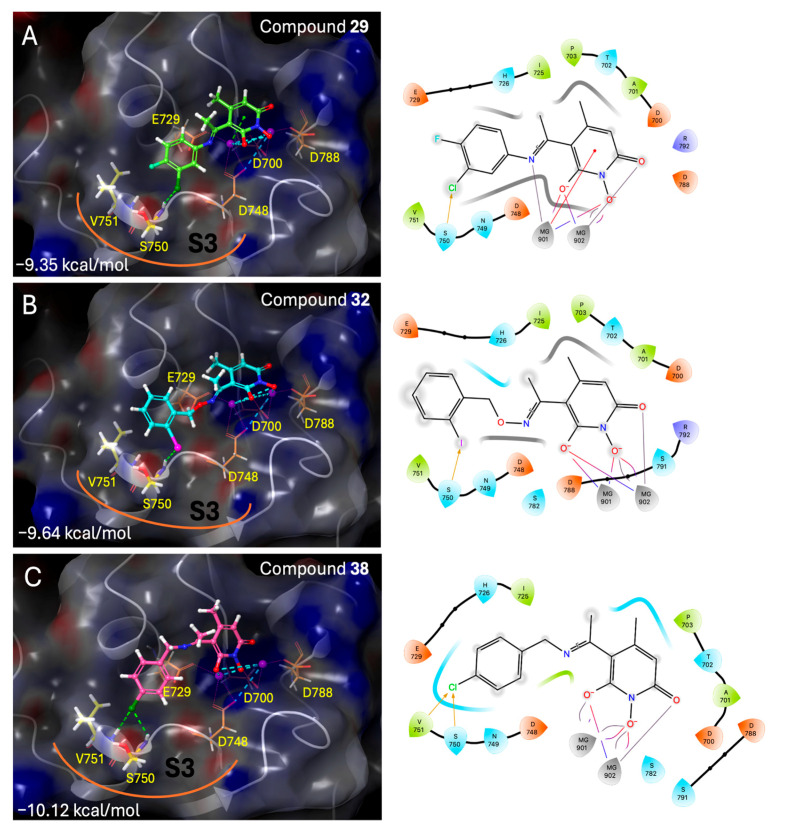
Compounds **29**, **32**, and **38** docked into the HBV RNase H active site. Surface diagram (**left**) and ligand interaction diagram (**right**) showing that in (**A**,**B**) side chains of compounds **29** (green) and **32** (cyan) are making halogen bond with only S750 (yellow), while in (**C**) compound **38** (pink) is making halogen bonds with residues S750 and V751 (yellow). Mg^2+^ ions (light pink), catalytic residues (orange).

Induced fit docking (IFD) experiments were performed to analyze the binding poses of these compounds into the active site of RNase H domain of P protein. At least five binding poses for each compound were produced to observe the binding diversity. All compounds except **44** and **45** contain two hydroxyl (–OH) groups on their main pharmacophore which deprotonated at pH 7.5 and chelated two Mg^2+^ ions in the active site of the RNase H. The deprotonated –OH groups on the main pharmacophore contributed salt bridge interactions with each of the Mg^2+^ ions. The docking scores of compounds ranged from −6.44 to −10.53 kcal/mol, while the EC_50_ values ranged from 0.5 to 100 µM (Table 1). We found a good correlation between EC_50_ values and docking scores for 17 out of 25 compounds. The side chains of compounds in the binding poses with highest docking scores were placed in the pocket S3, but the side chains of only four compounds had interactions with residues in the pocket, while others were solvent exposed (Figure 3). Compounds **29**, **32**, and **38** made halogen bonds with residue S750, while the side chain of **38** made an additional halogen bond with residue V751 in the pocket (Figure 4). The side chain of **44** made a halogen bond with E729 and the –NH_2_ group on its pharmacophore made a salt bridge with D748 and D700, and the other –N^+^ group made a hydrogen bond with A701 (Figure 3). Compound **44** has an EC_50_ value of >100 µM; despite having a docking score of −8.72 kcal/mol, it made only two salt bridge interactions with Mg^2+^ ions. Thus, the good docking score may relate more to interactions contributed by its side chain than the amino groups on its pharmacophore. Compound **45** has a docking score of −6.44 kcal/mol, predicting that it would bind more weakly than other compounds, and this is consistent with its lack of activity. The side chain of **45** was surface exposed and positioned towards S3 pocket (Figure 3).

## 3. Materials and Methods

### 3.1. Chemistry—General Part

During the conduct of the experimental part of the present study the following materials, apparatuses, and techniques were used. Melting points were determined using a Büchi capillary apparatus and are uncorrected. NMR experiments were performed to elucidate the structure and determine the purity of the newly synthesized compounds. ^1^H NMR and 2D NMR spectra (COSY, HSQC-DEPT, HMBC) were recorded on a Bruker DRX400 (Karlsruhe, Germany), DRX500 spectrometer (Karlsruhe, Germany) (400.13 MHz, 500.11 ^1^H NMR), and a Bruker Ultrashield™Plus Avance III 600 spectrometer (Karlsruhe, Germany) (600.11 MHz, ^1^H NMR). ^13^C NMR spectra were recorded on a Bruker DRX400 (Karlsruhe, Germany), DRX-500 spectrometer (Karlsruhe, Germany) (100.61 MHz, 125.77 ^13^C NMR), and a Bruker Ultrashield™Plus Avance III 600 spectrometer (Karlsruhe, Germany) (150.9 MHz, ^13^C NMR). Chemical shifts *δ* (*delta*) are reported in parts per million (ppm) downfield from the NMR solvent, with the tetramethylsilane or solvent (DMSO-*d6*) as internal standard. Data processing including Fourier transformation, baseline correction, phasing, peak peaking, and integrations was performed using MestReNova software v.12.0.0. Splitting patterns are designated as follows: s, singlet; d, doublet; t, triplet; q, quartet; dd, doublet of doublets; td, triplet of doublets; tt, triplet of triplets; tq, triplet of quartets; ddt, doublet of doublets of triplets; m, multiplet; complex m, complex multiplet. Coupling constants (*J*) are expressed in units of Hertz (Hz). The spectra were recorded at 293 K (20 °C) unless otherwise specified. The solvent used to obtain the spectra was deuterated DMSO, DMSO-*d6* (quin, 2.50 ppm, ^1^H NMR; septet, 39.52 ppm, ^13^C NMR). Analytical thin-layer chromatography (TLC) was used to monitor the progress of the reactions, as well as to authenticate the compounds. TLCs were conducted on, precoated with normal-phase silica gel, aluminum sheets (Silica gel 60 F254, Merck) (Darmstadt, Germany) (layer thickness 0.2 mm), precoated with reverse phase silica gel, aluminum sheets (Silica gel 60 RP-18 F254s, Merck) and precoated aluminum oxide plates (TLC aluminum oxide 60 F254, neutral). Developed plates were examined under a UV light source, at wavelengths of 254 nm, or after being stained by iodine vapours. The Retention factor (R*f*) of the newly synthesized compounds, that equals to the distance migrated over the total distance covered by the solvent, was also measured on the chromatoplates. Elemental analyses (C, H, N) were performed by the Service Central de Microanalyse at CNRS (France) and were within ±0.4% of the theoretical values. Elemental analysis results for the tested compounds correspond to ˃95% purity. The commercial reagents were purchased from Alfa Aesar (Leicestershire, UK), Sigma-Aldrich (St. Louis, MO, USA), and Merck (Darmstadt, Germany), and were used without further purification. Solvent abbreviations: ACN, acetonitrile; AcOEt, ethyl acetate; Et_2_O, diethyl ether; EtOH, ethanol; MeOH, methanol.

### 3.2. Chemistry—Experimental Procedures and Compound Characterization

#### 3.2.1. Synthesis of 5-Acetyl-1,6-dihydroxy-4-methylpyridin-2(1H)-one (Β)

**5-Acetyl-1-(benzyloxy)-6-hydroxy-4-methylpyridin-2(1H)-one** (**A**)**.**

After cooling in an ice bath, diketene (4.07 g, 3.73 mL, 48.4 mmol, 2.0 eq) was added dropwise to a stirred solution of *O*-benzylhydroxylamine (2.98 g, 24.2 mmol, 1.0 equiv.) and triethylamine (2.45 g, 3.38 mL, 24.2 mmol, 1.0 equiv.) in 19 mL dry toluene.

After 4.5 h of stirring at 65 °C under Argon, the mixture was concentrated to dryness under reduced pressure, and 150 mL10% HCl was added. The residue was partitioned between the aqueous phase and AcOEt (300 mL), the organic phase was extracted once more with HCl 10% (150 mL), and the combined aqueous phases were extracted once more with 150 mL AcOEt. The combined organic phases were washed with brine (3 × 200 mL), dried over anhydrous Na_2_SO_4,_ and the solvent was removed in vacuo. The residual brownish solid was triturated with Et_2_O and AcOEt sequentially to afford the title compound **A** as a beige crystalline solid (4.95 g, 75%); mp 144–146 °C (MeOH, AcOEt/n-pentane), R*_f_*_(*NP-TLC*)_ = 0.25 (AcOEt). ^1^H NMR (600.11 MHz, DMSO-*d_6_*) *δ* 2.34 (s, 3H, 4-CH_3_), 2.60 (s, 3H, 7-CH_3_), 5.05 (s, 2H, CH_2_Ph) 5.83 (s, 1H, H_3_), 7.37–7.44 (complex m, 3H, H_3’_, H_4’_, H_5’_), 7.54 (dd, 2H, *J*_1_ = 7.5 Hz, *J*_2_ = 1.7 Hz, H_2’_, H_6’_). ^13^C NMR (50.32 MHz, DMSO- *d_6_*) *δ* 23.8 (4-CH_3_), 29.0 (7-CH_3_), 76.9 (CH_2_Ph), 104.8 (C_5_), 106.7 (C_3_), 128.2 (C_3’_, C_5’_), 128.6 (C_4’_), 129.3 (C_2’_, C_6’_), 134.9 (C_1’_), 150.5 (C_4_), 159.0 (C_2_), 164.8 (C_6_), 193.4 (C_7_). Elemental analysis calcd. (%) for C_15_H_15_NO_4_: C, 65.92; H, 5.53; N, 5.13; found: C, 66.00; H, 5.59; N, 5.08 [20].

**5-Acetyl-1,6-dihydroxy-4-methylpyridin-2(1H)-one** (**B**)**.**

A solution of **A** (2.0 g, 7.32 mmol) in 120 mL MeOH was hydrogenated for 20 min at rt and 40 psi pressure, in the presence of 200 mg Pd/C (10 wt.%) as a catalyst.

After filtering out the catalyst and washing it with hot MeOH (3 × 20 mL), the combined filtrates evaporated under reduced pressure. The beige crystalline product was treated with AcOEt to yield the *N*-hydroxypyridinedione **B** almost quantitatively (1.32 g, 98%); mp 178–179 °C (MeOH/*n*-pentane, dry Et_2_O), R*_f_*_(*NP-TLC*)_ = 0.06 (AcOEt), R*_f_*_(*RP-TLC*)_ = 0.85 (H_2_O/ACN 7:3). ^1^H NMR (600.11 MHz, DMSO-*d6*) *δ* 2.32 (s, 3H, 4-CH_3_), 2.56 (s, 3H, 7-CH_3_), 5.80 (s, 1H, H_3_). ^13^C NMR (100.61 MHz, DMSO-*d6*) *δ* 23.3 (4-CH_3_), 28.0 (7-CH_3_), 104.7 (C_5_), 107.6 (C_3_), 149.1 (C_4_), 157.9 (C_2_), 165.1 (C_6_), 194.3 (C_7_). Elemental analysis calcd. (%) for C_8_H_9_NO_4_: C, 52.46; H, 4.95; N, 7.65; found: C, 52.51; H, 5.00; N, 7.58 [20].

#### 3.2.2. Synthesis of O-substituted N-hydroxyphthalimides (**5**–**8**)

General procedure:

To a solution of N-hydroxyphthalimide (2.45 mmol, 1 equiv.) in anhydrous DMF (2.4 mL), NaH (60% *w*/*w*, 3.07 mmol, 1.25 equiv.) is added at 0 °C. The mixture is stirred at RT for 30 min. Thereafter, the appropriate benzylalkylhalogenide (3.68 mmol, 1.5 equiv.) is added and the reaction is stirred at RT overnight. Then, water is added, and a solid precipitate is formed. The precipitate is filtered under vacuum and washed with water and a 7:3 solution of *n*-pentane/Et_2_O. The solid is then dried over P_2_O_5_ to afford the desired product.

**2-((3,4-dichlorobenzyl)oxy)isoindoline-1,3-dione** (**5**)

To a solution of *N*-hydroxyphthalimide (2.0 g, 12.26 mmol, 1.0 equiv.) in dry DMF (12 mL), K_2_CO_3_ (3.4 g, 24.52 mmol, 2.0 eq.) was added, and the mixture obtained a red colour. Then, 3,4-dichlorobenzyl chloride was added (3.59 g, 24.52 mmol, 2.0 eq.) and the mixture was stirred at 80 °C for 18 h, under Argon. After the completion, white solid was precipitated. The precipitate was filtered under vacuum and washed with water and *n*-pentane to afford **5** as a white solid (1.82 g, 46%) 1H NMR (400 MHz, CDCl_3_) d: 7.82 (dt, J ¼ 8.4, 3.2 Hz, 2H, Ar-H), 7.75 (dt, J ¼ 8.4, 3.2 Hz, 2H, Ar-H), 7.64 (d, J ¼ 1.6 Hz, 1H, Ar-H), 7.46 (d, J ¼ 8.4 Hz, 1H, Ar-H), 7.39 (dt, J ¼ 8.4, 1.6 Hz, 1H, Ar-H), 5.15 (s, 2H, Ar-CH_2_O-) [28].

**2-((2,4-dichlorobenzyl)oxy)isoindoline-1,3-dione** (**6**), was synthesized from 1-(bromomethyl)-2,4-dichlorobenzene according to the general procedure. White solid (745.4 mg, 94%). R*_f_* = 0.40 (CH_2_Cl_2_), mp: 154–156 °C, ^1^H NMR (400 MHz, CDCl_3_) *δ*: 7.82 (dt, *J* = 8.4, 3.2 Hz, 2H, Ar-Pthalimide), 7.75 (dt, *J* = 8.4, 3.2 Hz, 2H, Ar-Pthalimide), 7.59 (d, *J* = 8.4 Hz, 1H, Ar), 7.41 (d, *J* = 1.6 Hz, 1H, Ar), 7.27 (dt, *J* = 8.4, 1.6 Hz, 1H, Ar), 5.32 (s, 2H, CH_2_O). ^13^C NMR (100 MHz, CDCl_3_) *δ*: 163.3 (C_1_, C_3_), 135.8 (C_1’_), 135.3 (C_4’_), 134.5 (C_2’_), 132.4 (C_7a_, C_3a_), 130.5 (C_6_, C_5_), 129.5 (C_3’_), 128.7 (C_6’_), 127.4 (C_5’_), 123.6 (C_7_, C_4_), 75.7 (1’-CH_2_). Anal. Calcd for C_15_H_9_Cl_2_NO_3_: C, 55.93; H, 2.82; N, 4.35. Found: C, 55.97; H, 2.86; N, 4.39.

**2-((2-iodobenzyl)oxy)isoindoline-1,3-dione** (**7**)

To a solution of *N*-hydroxyphthalimide (357.1 mg, 2.19 mmol, 1.3 equiv.) in anhydrous THF (6 mL), ET_3_N (2.53 mmol, 1.5 eq.) was added, and the mixture was stirred at r.t. for 15 min. Then, 1-(bromomethyl)-2-iodobenzene (1.68 mmol, 1 eq.) was added and the mixture was stirred for 18 h at r.t. (overnight). The solvent was concentrated under reduced pressure, and 40 mL of H_2_O was added to the residue. The product was extracted with CH_2_Cl_2._ The combined organic layers were washed with H_2_O (3 × 20 mL) and with brine solution (20 mL). Then, they were dried with anh. Na_2_SO_4_ and the solvents were concentrated under reduced pressure. The white solid formed was purified by flash column chromatography (1:1 n-hexane/EtOAc and then EtOAc) to obtain a yellow solid (2710 mg, 43%). ^1^H NMR (400 MHz, DMSO) δ 7.90 (dd, *J* = 7.9, 1.2 Hz, 1H), 7.86 (s, 4H), 7.61 (dd, *J* = 7.7, 1.7 Hz, 1H), 7.44 (td, *J* = 7.5, 1.3 Hz, 1H), 7.15 (td, *J* = 7.7, 1.7 Hz, 1H), 5.25 (s, 2H) [29].

**2-((2-chlorobenzyl)oxy)isoindoline-1,3-dione** (**8**) was synthesized from 1-chloro-2-(chloromethyl)benzene (465 μL, 3.68 mmol) according to the general procedure. Pink solid (652.6 mg, 93%). ^1^H NMR (600 MHz, CDCl_3_) δ 7.81 (dd, *J* = 5.4, 3.1 Hz, 2H), 7.74 (dd, *J* = 5.5, 3.1 Hz, 2H), 7.65–7.62 (m, 1H), 7.41–7.38 (m, 1H), 7.33–7.28 (m, 2H), 5.37 (s, 2H) [28].

#### 3.2.3. Synthesis of *O*-substituted Hydroxylamines (**9**–**12**)

General procedure:

To a solution of the appropriate *N*-hydroxyphthalimide (0.78 mmol, 1 equiv.) in CH_2_Cl_2_ (3 mL), H_2_NNH_2_ 64% *w*/*w* (2 equiv.) is added, and the reaction is stirred at RT for 1–24 h. After the reaction is complete, the formed white precipitate is filtered, washed with CH_2_Cl_2_, and the filtrate is concentrated to afford the corresponding hydroxylamine.

***O*-(3,4-dichlorobenzyl)hydroxylamine** (**9**)

To a solution of the compound (**5**) (1.72 g, 5.34 mmol, 1 equiv.) in CH_2_Cl_2_ (18 mL), hydrazine monohydrate 64% *w*/*w* (534 mg, 10.68 mmol, 2 equiv.) is added, and the reaction is stirred at RT for 4 h. The formed white precipitate is filtered, washed with CH_2_Cl_2_, and the filtrate is concentrated to afford an off-yellow oil, which was further purified by flash column chromatography (CH_2_Cl_2_). (720 mg, 70%). ^1^H NMR (400 MHz, CDCl_3_) d: 7.40 (s, 1H, Ar-H), 7.36 (d, J ¼ 8.0 Hz, 1H, Ar-H), 7.13 (d, J ¼ 8.0 Hz, 1H, Ar-H), 5.45 (brs, 2H, –NH_2_), 4.57 (s, 2H, Ar-CH_2_O-) [28].

***O*-(2,4-dichlorobenzyl)hydroxylamine** (**10**) was synthesized from compound (**6**) according to the general procedure, to afford an off-yellow oil (140 mg, 94%), R*_f_* = 0.20 (CH_2_Cl_2_), ^1^H NMR (400 MHz, CDCl_3_) *δ*: 7.34 (s, 1H, Ar), 7.32 (d, *J* = 8.0 Hz, 1H, Ar), 7.20 (d, *J* = 8.0 Hz, 1H, Ar), 5.50 (broad s, 2H, –NH_2_), 4.72 (s, 2H, CH_2_O). ^13^C NMR (100 MHz, CDCl_3_) *δ*: 134.2 (C_1_), 134.0 (C_2,_ C_4_), 130.6 (C_3_), 129.2 (C_6_), 126.9 (C_5_), 74.2 (OCH_2_).

***O*-(2-iodobenzyl)hydroxylamine** (**11**) was synthesized from compound (**7**) (0.71 mmol) according to the general procedure (24 h), to afford an off-yellow oil (176 mg, 99%), ^1^H NMR (400 MHz, CDCl_3_) δ 7.85 (d, *J* = 7.9 Hz, 1H), 7.43–7.31 (m, 2H), 7.00 (t, *J* = 7.5 Hz, 1H), 5.51 (br, s, 2H), 4.73 (s, 2H) [29].

***O*-(2-chlorobenzyl)hydroxylamine** (**12**) was synthesized from compound (**8**) (0.87 mmol) according to the general procedure (3 h), to afford a green oil (87.9 mg, 64%) ^1^H NMR (400 MHz, CDCl_3_) δ 7.38–7.40 (m, 1H), 7.31–7.33 (m, 1H), 7.17–7.21 (m, 2H), 5.26 (brs, 2H), 4.76 (s, 2H) [28].

#### 3.2.4. Synthesis of *N*-substituted *N*-hydroxyphthalimides (**13**–**15**)

General procedure:

To a solution of phthalimide (500 mg, 3.40 mmol, 1 equiv.) in anhydrous DMF (3.4 mL), K_2_CO_3_ (1.5 eq.) was added, and the appropriate benzyl bromide (1.2 eq.). The mixture is stirred at 80 °C for 16–18 h, under Argon. After the completion of the reaction, H_2_O is added, and the product is extracted with EtOAc (3 × 30 mL). The combined organic layers were washed with brine solution (3 × 30 mL). Then, they were dried with anh. Na_2_SO_4_ and the solvents were concentrated under reduced pressure. The residue formed is purified by flash column chromatography or by recrystallization with EtOH.

**2-(2-bromobenzyl)isoindoline-1,3-dione** (**13**) was synthesized according to the general procedure from 1-(bromomethyl)-2-bromobenzene (558.6 μL, 4.08 mmol). The solid was purified by recrystallization with EtOH, to obtain the desired product as a white solid (919.9 mg, 91%), ^1^H NMR (400 MHz, CDCl_3_) δ 7.92–7.87 (m, 2H), 7.78–7.73 (m, 2H), 7.60–7.55 (m, 1H), 7.26–7.20 (m, 1H), 7.17–7.10 (m, 2H), 4.98 (s, 2H) [30].

**2-(3-bromobenzyl)isoindoline-1,3-dione** (**14**)

To a solution of phthalimide (500 mg, 3.40 mmol, 1.1 equiv.) in anhydrous DMF (7.7 mL), K_2_CO_3_ (3 eq.) and 1-(bromomethyl-)3-bromobenzene (1 eq.) were added. The mixture is stirred at 40 °C overnight, under Argon. After the completion of the reaction, 40 mL of cold H_2_O is added, and a white solid is precipitated. The solid formed was filtered off in vacuo and washed with 100 mL H_2_O and 80 mL of a solution of PE/EtOAc 10/1. The desired product is obtained as a white solid (505,1 mg, 52%), ^1^H NMR (400 MHz, DMSO) δ 7.92–7.84 (m, 4H), 7.54 (s, 1H), 7.47 (dt, *J* = 6.6, 2.2 Hz, 1H), 7.32–7.27 (m, 2H), 4.77 (s, 2H) [31].

**2-(4-bromobenzyl)isoindoline-1,3-dione** (**15**)

To a solution of phthalimide (500 mg, 3.40 mmol, 1.1 equiv.) in anhydrous DMF (3.9 mL), Cs_2_CO_3_ (2 eq.) and 1-(bromomethyl-)4-bromobenzene (1 eq.) were added. The mixture is stirred at r.t. for 22 h, under Argon. After the completion of the reaction, 70 mL of H_2_O is added, and the product is extracted from CH_2_Cl_2_ (2 × 70 mL). The combined organic layers were washed with brine solution (2 × 50 mL). Then, they were dried with anh. Na_2_SO_4_ and the solvents were concentrated under reduced pressure. The residue formed is purified by column chromatography (gravity, PE/EtOAc 3/1). The desired product is obtained as a white solid (607.9 mg, 56%), ^1^H NMR (400 MHz, CDCl_3_) δ 7.87–7.81 (m, 2H), 7.74–7.68 (m, 2H), 7.44 (d, *J* = 8.4 Hz, 2H), 7.31 (d, *J* = 8.5 Hz, 2H), 4.79 (s, 2H) [31].

#### 3.2.5. Synthesis of Substituted Benzylamines (**16**–**18**)

General procedure:

To a solution of the appropriate phthalimide (0.98 mmol, 1 equiv.) in absolute EtOH (3 mL), hydrazine monohydrate 64% *w*/*w* (2 equiv.) is added, and the reaction is refluxed for 1 h. The appropriate amine is obtained with a different procedure each time.

**(2-bromophenyl)methanamine** (**16**) was synthesized from compound (**13**) (200 mg, 0.63 mmol). After the completion of the reaction, the solvent is concentrated under reduced pressure. Then, 5 mL of conc. HCl is added, and the mixture is stirred at 80 °C for 1 h. Next, solution of NaOH 2 N is added to the mixture, until it becomes basic (pH = 8). The product is extracted with EtOAc (3 × 50 mL). The combined organic layers were washed with 50 mL H_2_O and then with 50 mL brine solution. Then, they were dried with anh. Na_2_SO_4_ and the solvents were concentrated under reduced pressure. The desired product is obtained as a yellow oil (108.7 mg, 62%), ^1^H NMR (400 MHz, CDCl_3_) δ 7.55 (d, *J* = 8.0 Hz, 1H), 7.38 (d, *J* = 6.9 Hz, 1H), 7.30 (t, *J* = 7.4 Hz, 1H), 7.13 (dt, *J* = 8.9, 4.5 Hz, 1H), 3.93 (s, 2H) [32].

**(3-bromophenyl)methanamine** (**17**) was synthesized from compound (**14**) (300 mg, 0.95 mmol). After the completion of the reaction, the solvent is concentrated under reduced pressure. Then, 5 mL of conc. HCl is added, and the mixture is stirred at 80 °C for 1 h. Next, solution of aq. NaOH 2 N is added to the mixture, until it becomes basic (pH = 8). The product is extracted with EtOAc (3 × 50 mL). The combined organic layers were washed with 50 mL H_2_O and then with 50 mL brine solution. Then, they were dried with anhydrous. Na_2_SO_4_ and the solvents were concentrated under reduced pressure. The desired product is obtained as a yellow oil (100.0 mg, 57%), ^1^H NMR (400 MHz, CDCl_3_) δ 7.48 (t, *J* = 1.9 Hz, 1H), 7.38 (dt, *J* = 7.5, 1.8 Hz, 1H), 7.25–7.17 (m, 2H), 3.86 (s, 2H) [33].

**(4-bromophenyl)methanamine** (**18**) was synthesized from compound (**15**) (200 mg, 0.63 mmol). After the completion of the reaction, the solvent is concentrated under reduced pressure. Then, 5 mL of conc. HCl is added, and the mixture is stirred at 80 °C for 1 h. Next, it is added to the mixture solution of aq. NaOH 2 N, until it becomes basic (pH = 8). The product is extracted with EtOAc (3 × 50 mL). The combined organic layers were washed with 50 mL H_2_O and then with 50 mL brine solution. Then, they were dried with anh. Na_2_SO_4_ and the solvents were concentrated under reduced pressure. The desired product is obtained as a yellow oil (103.8 mg, 59%), ^1^H NMR (400 MHz, CDCl_3_) δ 7.46 (d, *J* = 8.4 Hz, 2H), 7.20 (d, *J* = 8.4 Hz, 2H), 3.85 (s, 2H), 3.12 (s, 4H) [32].

#### 3.2.6. Synthesis of 6-Substituted 2,4-diaminopyrimidines (**19**)

General procedure:

To a flask containing the stirring corresponding alcohol (7.4 equiv.), NaH (1.2 equiv.) is added slowly, and the mixture is stirred at 150 °C for 1.5 h. Then, 6-chloropyrimidine-2,4-diamine is added (1 equiv.) and the resulting mixture is stirred at 140–150 °C overnight, under Argon. The brown residue is extracted with EtOAc (3 × 50 mL). The combined organic phases are washed with brine (3 × 40 mL), dried over anh. Na_2_SO_4_, filtered, and concentrated. The resulting crude mixture is purified by gravity column chromatography (1:1 CH_2_Cl_2_: AcOEt, AcOEt, 3:1 AcOEt: MeOH as eluent or recrystallized with EtOAc or triturated with Et_2_O).

**6-(2-bromophenoxy)pyrimidine-2,4-diamine** (**19**) was synthesized according to the general procedure, and it was purified by column chromatography and triturated as it has been described in the general procedure to afford (**19**) as an off-white solid. (591.6 mg, 61%). R*_f_* = 0.38 (AcOEt), ^1^H NMR (500 MHz, DMSO) δ 7.68 (d, *J* = 8.0 Hz, 1H, Ph), 7.41 (t, *J* = 7.9 Hz, 1H, Ph), 7.26–7.13 (m, 2H, Ph), 6.27 (s, 2H, NH_2_), 5.99 (s, 2H, NH_2_), 5.05 (s, 1H, CHCNH_2_). ^13^C NMR (126 MHz, DMSO) δ 169.50 (C_6_), 166.50 (C_4_), 163.21 (C_2_), 150.21 (C_1’_), 133.23 (C_3’_), 129.00 (C_5’_), 126.70 (C_4’_), 124.32 (C_6’_), 115.96 (C_2’_), 76.77 (C_5_). Anal. Calcd for C_10_H_9_BrN_4_O: C, 42.73; H, 3.23; N, 19.93. Found: C, 42.76; H, 3.26; N, 19.96.

#### 3.2.7. Synthesis of 2-(4-Chlorophenoxy)ethan-1-amine (**20**)

In a solution of (4-chlorophenoxy)acetonitrile (200 mg, 1,19 mmol, 1 eq) in anhydrous Et_2_O in ambient temperature, LiAlH_4_ (135.8 mg, 3.58 mmol, 3 eq) is added, and the mixture is stirred for 2 h. After the completion of the reactions, the mixture is cooled to 0 °C and EtOH, H_2_O and aq. NaOH 15% *w*/*v* is added sequentially and dropwise, until LiAlH_4_ discoloration to white. The solid is filtered with hot EtOAc using a folded filtered paper. The filtrate is concentrated under reduced pressure to obtain colourless oil (130 mg, 64%).

^1^H NMR (300 MHz, CDCl_3_, δ): 7.28–7.21 (m, 2H), 6.89–6.81 (m, 2H), 4.26 (t, *J* = 6.4 Hz, 2H), 3.62 (t, *J* = 6.4 Hz, 2H) [34].

#### 3.2.8. Synthesis of *N*-hydroxypyridinedione Imines (**21**–**29**)

**5-(1-((2-fluorophenyl)imino)ethyl)-1,6-dihydroxy-4-methylpyridin-2(1H)-one** (**21**)

To a stirred solution of **B** (200 mg, 1.09 mmol, 1.0 eq) in 4 mL of absolute EtOH at 75 °C activated 4Å molecular sieves, 2-fluoroaniline (133 mg, 116 μL, 1.2 mmol, 1.1 eq), and a drop of sulfuric acid were added successively. The mixture was refluxed for 4.5 h at 60 °C under Argon and for 2.5 h at 30 °C. During the reaction, a yellow solid was formed. The precipitate was filtered off in vacuo and washed with small portions of EtOH and EtOAc to afford (**21**) as a yellow crystalline solid (180 mg, 60%); mp 190–193 °C (AcOEt/n-pentane), R*_f_*_(*alox*)_ = 0.05 (MeOH), R*_f_*_(*RP-TLC*)_ = 0.07 (H_2_O/ACN 7:3); ^1^H NMR (600.11 MHz, DMSO-d6) δ (ppm) 2.38 (s, 3H, 4-CH_3_), 2.46 (s, 3H, 7-CH_3_), 5.73 (s, 1H, H_3_), 7.30-7.37 (m, 1H, H4’), 7.41–7.47 (complex m, 2H, H_3’_, H_6’_), 7.49 (t, 1H, *J* = 8.0 Hz, H_5’_), 10.11 (s, 1H, 1-OH), 14.78 (s, 1H, 6-OH); ^13^C NMR (150.9 MHz, DMSO-d6) δ (ppm) 20.4 (7-CH_3_), 25.1 (4-CH_3_), 99.7 (C_5_), 110.8 (C_3_), 116.4, 116.5 (d, *J*_C-F_ = 19.5 Hz, C_3’_), 124.6, 124.7 (d, *J*_C-F_ = 12.2 Hz, C_1’_), 125.11, 125.12 (d, *J*_C-F_ = 2.8 Hz, C_4’_), 128.2 (C_5’_), 129.38, 129.43 (d, *J*_C-F_ = 7.9 Hz, C_6’_), 148.3 (C_4_), 155.0, 156.6 (d, *J*_C-F_ = 247.1 Hz, C_2’_), 158.8 (C_2_), 164.6 (C_6_), 169.1 (C_7_). Anal. Calcd for C_14_H_13_FN_2_O_3_: C, 60.87; H, 4.74; N, 10.14. Found: C, 60.91; H, 4.79; N, 10.18.

**5-(1-((3,4-difluorophenyl)imino)ethyl)-1,6-dihydroxy-4-methylpyridin-2(1H)-one** (**22**)

To a stirred solution of **B** (200 mg, 1.09 mmol, 1.0 eq) in 4 mL of absolute EtOH activated 4Å molecular sieves, 3,4-difluoroaniline (155 mg, 119 μL, 1.2 mmol, 1.1 eq), and a drop of sulfuric acid were added successively. The mixture was refluxed for 4.5 h at 60 °C under Argon. The precipitate formed was filtered off in vacuo and washed with EtOH to afford (**22**) as a beige crystalline solid (240 mg, 75%); mp 217–219 °C (EtOH), R*_f_*_(*alox*)_ = 0.05 (MeOH), R*_f_*_(*RP-TLC*)_ = 0.05 (H_2_O/ACN 7:3); ^1^H NMR (600.11 MHz, DMSO-*d_6_*) *δ* (ppm) 2.36 (s, 3H, 4-CH_3_), 2.45 (s, 3H, 7-CH_3_), 5.71 (s, 1H, H_3_), 7.22 (ddd, 1H, *J*_1_ = 9.2 Hz, *J*_2_ = 4.3 Hz, *J*_3_ = 2.1 Hz, H_6’_), 7.54–7.60 (complex m, 2H, H_2’_, H_5’_), 10.09 (s, 1H, 1-OH), 14.77 (s, 1H, 6-OH); ^13^C NMR (150.9 MHz, DMSO-*d_6_*) *δ* (ppm) 20.7 (7-CH_3_), 25.1 (4-CH_3_), 99.5 (C_5_), 110.6 (C_3_), 115.6, 115.8 (d, *J*_C-F_ = 19.1 Hz, C_5’_), 118.0, 118.2 (d, *J*_C-F_ = 18.2 Hz, C_2’_), 123.0 (C_6’_), 133.7 (C_1’_), 147.6, 147.7, 148.4, 148.5 (d, *J*_1(C-F)_ = 121.2 Hz, *J*_2(C-F)_ = 13.2 Hz, C_4’_), 148.3 (C_4_), 149.2, 149.3, 150.1, 150.1 (d, *J*_1(C-F)_ = 122.5 Hz, *J*_2(C-F)_ = 13.0 Hz, C_3’_), 158.8 (C_2_), 164.4 (C_6_), 168.8 (C_7_). Anal. Calcd for C_14_H_12_F_2_N_2_O_3_: C, 57.15; H, 4.11; N, 9.52. Found: C, 57.19; H, 4.05; N, 9.58.

**5-(1-((2-chlorophenyl)imino)ethyl)-1,6-dihydroxy-4-methylpyridin-2(1H)-one** (**23**)

To a stirred solution of **B** (200 mg, 1.09 mmol, 1.0 eq) in 4 mL of absolute EtOH at 75 °C activated 4Å molecular sieves, 2-chloroaniline (153 mg, 126 μL, 1.2 mmol, 1.1 eq), and a drop of sulfuric acid were added successively. The mixture was refluxed for 24 h at 60 °C. After the competition of the reaction the solvent was evaporated in half of its volume, and the solid formed was filtered off in vacuo and washed with small portions of EtOH to afford (**23**) as a yellow crystalline solid (80 mg, 25%); mp 184186 °C (AcOEt/n-pentane), R*_f_*_(*alox*)_ = 0.01 (MeOH), R*_f_*_(*RP-TLC*)_ = 0.05 (H_2_O/ACN 7:3); ^1^H NMR (600.11 MHz, DMSO-d_6_) δ (ppm) 2.39 (s, 3H, 4-CH_3_), 2.42 (s, 3H, 7-CH_3_), 5.73 (s, 1H, H_3_), 7.43 (td, 1H, *J*_1_ = 7.7 Hz, *J*_2_ = 1.7 Hz, H_5’_), 7.49 (td, 1H, *J*_1_ = 7.6 Hz, *J*_2_ = 1.5 Hz, H_4’_), 7.54 (dd, 1H, *J*_1_ = 7.9 Hz, *J*_2_ = 1.7 Hz, H_3’_), 7.68 (dd, 1H, *J*_1_ = 8.0 Hz, *J*_2_ = 1.5 Hz, H_6’_), 10.12 (s, 1H, 1-OH), 14.94 (s, 1H, 6-OH); ^13^C NMR (150.9 MHz, DMSO-d_6_) δ (ppm) 20.6 (7-CH_3_), 25.2 (4-CH_3_), 99.5 (C_5_), 110.8 (C_3_), 28.1 (C_4’_), 128.6 (C_3’_), 129.1 (C_2’_), 129.2 (C_5’_), 130.1 (C_6’_), 134.4 (C_1’_), 148.4 (C_4_), 158.8 (C_2_), 164.7 (C_6_), 168.9 (C_7_). Anal. Calcd for C_14_H_13_ClN_2_O_3_: C, 57.45; H, 4.48; N, 9.57. Found: C, 57.53; H, 4.50; N, 9.47.

**5-(1-((3,4-dichlorophenyl)imino)ethyl)-1,6-dihydroxy-4-methylpyridin-2(1H)-one** (**24**)

To a stirred solution of **B** (200 mg, 1.09 mmol, 1.0 eq) in 4 mL of absolute EtOH activated 4Å molecular sieves, 3,4-dichloroaniline (194 mg, 1.2 mmol, 1.1 eq), and a drop of sulfuric acid were added successively. The mixture was refluxed for 4 h at 60 °C under Argon. The precipitate formed was filtered off in vacuo and washed with EtOH to afford (**24**) as a yellow crystalline solid (250 mg, 70%); mp 217–219 °C (EtOH), R*_f_*_(*alox*)_ = 0.01 (MeOH), R*_f_*_(*RP-TLC*)_ = 0.01 (H_2_O/ACN 7:3); ^1^H NMR (600.11 MHz, DMSO-*d_6_*) *δ* (ppm) 2.37 (s, 3H, 4-CH_3_), 2.48 (s, 3H, 7-CH_3_), 5.73 (s, 1H, H_3_), 7.35 (dd, 1H, *J*_1_ = 8.6 Hz, *J*_2_ = 2.5 Hz, H_6’_), 7.71 (d, 1H, *J* = 2.5 Hz, H_2’_), 7.75 (d, 1H, *J* = 8.5 Hz, H_5’_), 10.10 (s, 1H, 1-OH), 14.79 (s, 1H, 6-OH); ^13^C NMR (150.9 MHz, DMSO-*d_6_*) *δ* (ppm) 20.8 (7-CH_3_), 25.0 (4-CH_3_), 99.9 (C_5_), 110.8 (C_3_), 126.1 (C_6’_), 127.7 (C_2’_), 129.8 (C_4’_), 131.2 (C_5’_), 131.7 (C_3’_), 137.0 (C_1’_), 148.3 (C_4_), 158.8 (C_2_), 164.4 (C_6_), 168.4 (C_7_). Anal. Calcd for C_14_H_12_Cl_2_N_2_O_3_: C, 51.40; H, 3.70; N, 8.56. Found: C, 51.44; H, 3.65; N, 8.60.

**5-(1-((2-bromophenyl)imino)ethyl)-1,6-dihydroxy-4-methylpyridin-2(1H)-one** (**25**)

To a stirred solution of **B** (300 mg, 1.64 mmol, 1.0 eq) in 5 mL of absolute EtOH at 75 °C activated 4Å molecular sieves, 2-bromoaniline (310 mg, 1.8 mmol, 1.1 eq), and 2 drops of sulfuric acid were added successively. The mixture was refluxed for 48 h at 65 °C under Argon. The solvent was concentrated under reduced pressure, and the brown residue was triturated with 3 mL EtOAc. The precipitate formed was filtered off in vacuo and washed with MeOH. The filtrate was concentrated under reduced pressure and chromatographed on a Sephadex LH-20 column eluted with MeOH. The solid was triturated with dry Et_2_O and abs. EtOH to afford (**25**) as a yellow crystalline solid (140 mg, 25%); mp: 198–200 °C (MeOH/dry Et_2_O), R*_f_*_(*alox*)_ = 0.13 (MeOH), R*_f_*_(*RP-TLC*)_ = 0.09 (H_2_O/ACN 7:3). ^1^H NMR (600.11 MHz, DMSO-*d_6_*) *δ* (ppm): 2.38 (s, 3H, 4-CH_3_), 2.40 (s, 3H, 7-CH_3_), 5.73 (s, 1H, H_3_), 7.36 (ddd, 1H, *J*_1_ = 8.5 Hz, *J*_2_ = 5.8 Hz, *J*_3_ = 3.3 Hz, H_4’_), 7.50–7.55 (m, 2H, H_5’_, H_6’_), 7.83 (d, 1H, *J* = 7.6 Hz, H_3’_), 10.14 (s, 1H, 1-OH), 14.92 (s, 1H, 6-OH); ^13^C NMR (150.9 MHz, DMSO-*d_6_*) *δ* (ppm): 20.6 (7-CH_3_), 25.3 (4-CH_3_), 99.4 (C_5_), 110.7 (C_3_), 119.8 (C_2’_), 128.8 (C_5’_), 128.8 (C_6’_), 129.5 (C_4’_), 133.3 (C_3’_), 136.0 (C_1’_), 148.5 (C_4_), 158.8 (C_2_), 164.7 (C_6_), 168.9 (C_7_). Anal. Calcd for C_14_H_13_BrN_2_O_3_: C, 49.87; H, 3.89; N, 8.31. Found: C, 49.79; H, 3.86; N, 8.34.

**5-(1-((2,4-difluorophenyl)imino)ethyl)-1,6-dihydroxy-4-methylpyridin-2(1H)-one** (**26**)

To a stirred solution of **B** (200 mg, 1.09 mmol, 1.0 eq) in 4 mL of absolute EtOH at 75 °C activated 4Å molecular sieves, 2,4-difluoroaniline (155 mg, 120 μL, 1.2 mmol, 1.1 eq), and a drop of sulfuric acid were added successively. The mixture was refluxed for 4 h at 60 °C under Argon. The precipitate formed was filtered off in vacuo and washed with EtOH to afford **(26)** as a yellow crystalline solid (270 mg, 84%); mp 230–233 °C (EtOH), R*_f_*_(*alox*)_ = 0.01 (MeOH), R*_f_*_(*RP-TLC*)_ = 0.02 (H_2_O/ACN 7:3); ^1^H NMR (600.11 MHz, DMSO-*d_6_*) *δ* (ppm) 2.38 (s, 3H, 4-CH_3_), 2.43 (s, 3H, 7-CH_3_), 5.73 (s, 1H, H_3_), 7.24 (td, 1H, *J*_1_ = 8.3 Hz, *J*_2_ = 2.8 Hz, H_5’_), 7.52 (ddd, 1H, *J*_1_ = 11.1 Hz, *J*_2_ = 8.9 Hz, *J*_3_ = 2.8 Hz, H_3’_), 7.56 (td, 1H, *J*_1_ = 9.0 Hz, *J*_2_ = 5.9 Hz, H_6’_), 10.11 (s, 1H, 1-OH), 14.64 (s, 1H, 6-OH); ^13^C NMR (150.9 MHz, DMSO-*d_6_*) *δ* (ppm) 20.3 (7-CH_3_), 25.1 (4-CH_3_), 99.7 (C_5_), 105.1 (d, *J*_C-F_ = 26.5 Hz, C_3’_), 110.8 (C_3_), 112.18 (dd, *J*_1(C-F)_ = 22.5 Hz, *J*_2(C-F)_ = 2.8 Hz, C_5’_), 121.44 (d, *J*_C-F_ = 13.4 Hz, C_1’_), 129.51 (d, *J*_C-F_ = 13.4 Hz, C_6’_), 148.3 (C_4_), 156.09 (dd, *J*_1(C-F)_ = 249.5 Hz, *J*_2(C-F)_ = 13.2 Hz, C_2’_), 158.8 (C_2_), 160.97 (dd, *J*_1(C-F)_ = 247.4 Hz, *J*_2(C-F)_ = 11.7 Hz, C_4’_), 164.6 (C_6_), 169.3 (C_7_). Anal. Calcd for C_14_H_12_F_2_N_2_O_3_: C, 57.15; H, 4.11; N, 9.52. Found: C, 57.20; H, 4.15; N, 9.50.

**5-(1-((2,5-difluorophenyl)imino)ethyl)-1,6-dihydroxy-4-methylpyridin-2(1H)-one** (**27**)

To a stirred solution of **B** (200 mg, 1.09 mmol, 1.0 eq) in 4 mL of absolute EtOH at 75 °C, 2,5-difluoroaniline (155 mg, 120 μL, 1.2 mmol, 1.1 eq) was added, and the mixture was refluxed for 4 h at 60 °C under Argon. The solvent was concentrated under reduced pressure, and the residue was triturated with ET_2_O. The precipitate formed was filtered off in vacuo and washed with EtOH. The solid was chromatographed on a Sephadex LH-20 column eluted with MeOH to afford (**27**) as a yellow crystalline solid (230 mg, 72%); mp 173–178 °C (EtOH), R*_f_*_(*alox*)_ = 0.01 (MeOH), R*_f_*_(*RP-TLC*)_ = 0.01 (H_2_O/ACN 7:3); ^1^H NMR (600.11 MHz, DMSO-*d_6_*) *δ* (ppm) 2.38 (s, 3H, 4-CH_3_), 2.49 (s, 3H, 7-CH_3_), 5.76 (s, 1H, H_3_), 7.30 (ddd, 1H, *J*_1_ = 8.9 Hz, *J*_2_ = 6.9 Hz, *J*_3_ = 3.6 Hz, H_4’_), 7.47–7.53 (m, 2H, H_3’_, H_6’_), 10.19 (s, 1H, 1-OH), 14.79 (s, 1H, 6-OH); ^13^C NMR (150.9 MHz, DMSO-*d_6_*) *δ* (ppm) 20.5 (7-CH_3_), 25.0 (4-CH_3_), 100.2 (C_5_), 111.2 (C_3_), 115.04 (d, *J*_C-F_ = 26.3 Hz, C_6’_), 115.46 (dd, *J*_1(C-F)_ = 23.9 Hz, *J*_2(C-F)_ = 8.0 Hz, C_4’_), 117.50 (dd, *J*_1(C-F)_ = 22.6 Hz, *J*_2(C-F)_ = 9.6 Hz, C_3’_), 125.85 (dd, *J*_1(C-F)_ = 14.2 Hz, *J*_2(C-F)_ = 11.5 Hz, C_1’_), 148.2 (C_4_), 152.28 (d, *J*_C-F_ = 243.2 Hz, C_2’_), 157.72 (d, *J*_C-F_ = 242.1 Hz, C_5’_), 158.8 (C_2_), 164.7 (C_6_), 168.7 (C_7_). Anal. Calcd for C_14_H_12_F_2_N_2_O_3_: C, 57.15; H, 4.11; N, 9.52. Found: C, 57.11; H, 4.08; N, 9.55.

**5-(1-((2,6-difluorophenyl)imino)ethyl)-1,6-dihydroxy-4-methylpyridin-2(1H)-one** (**28**)

To a stirred solution of **B** (200 mg, 1.09 mmol, 1.0 eq) in 4 mL of absolute EtOH at 75 °C, 2,6-difluoroaniline (155 mg, 120 μL, 1.2 mmol, 1.1 eq) was added and the mixture was refluxed for 16 h at 60 °C under Argon. The solvent was concentrated under reduced pressure and the orange residue was chromatographed on a Sephadex LH-20 column eluted with MeOH to afford (**28**) as a yellow crystalline solid (145 mg, 45%); mp 188–194 °C (EtOH/dry Et_2_O), R*_f_*_(*alox*)_ = 0.02 (MeOH), R*_f_*_(*RP-TLC*)_ = 0.06 (H_2_O/ACN 7:3); ^1^H NMR (600.11 MHz, DMSO-*d_6_*) *δ* (ppm) 2.38 (s, 3H, 4-CH_3_), 2.49 (s, 3H, 7-CH_3_), 5.76 (d, 1H, *J* = 1.1 Hz, H_3_), 7.30 (ddd, 1H, *J*_1_ = 8.9 Hz, *J*_2_ = 6.9 Hz, *J*_3_ = 3.6 Hz, H_4’_), 7.47–7.53 (m, 2H, H_3’_, H_6’_), 10.15 (s, 1H, 1-OH), 14.51 (s, 1H, 6-OH); ^13^C NMR (150.9 MHz, DMSO-*d_6_*) *δ* (ppm) 20.5 (7-CH_3_), 25.0 (4-CH_3_), 99.9 (C_5_), 111.3 (C_3_), 115.04 (d, *J*_C-F_ = 26.3 Hz, C_6’_), 115.46 (dd, *J*_1(C-F)_ = 23.9 Hz, *J*_2(C-F)_ = 8.0 Hz, C_4’_), 117.50 (dd, *J*_1(C-F)_ = 22.6 Hz, *J*_2(C-F)_ = 9.6 Hz, C_3’_), 125.85 (dd, *J*_1(C-F)_ = 14.2 Hz, *J*_2(C-F)_ = 11.5 Hz, C_1’_), 148.3 (C_4_), 152.28 (d, *J*_C-F_ = 243.2 Hz, C_2’_), 157.72 (d, *J*_C-F_ = 242.1 Hz, C_5’_), 158.8 (C_2_), 164.9 (C_6_), 169.6 (C_7_). Anal. Calcd for C_14_H_12_F_2_N_2_O_3_: C, 57.15; H, 4.11; N, 9.52. Found: C, 57.20; H, 4.13; N, 9.52.

**5-(1-((3-chloro-4-fluorophenyl)imino)ethyl)-1,6-dihydroxy-4-methylpyridin-2(1H)-one** (**29**)

To a stirred solution of **B** (200 mg, 1.09 mmol, 1.0 eq) in 4 mL of absolute EtOH activated 4Å molecular sieves, 3-chloro-4-fluoroaniline (175 mg, 1.2 mmol, 1.1 eq) and a drop of sulfuric acid were added successively, and a yellow solid was formed. The mixture was refluxed for 20 h at 60 °C under Argon. The precipitate formed was filtered off in vacuo and washed with EtOH and MeOH to afford (**29**) as a pale yellow crystalline solid (195 mg, 57%). Mp: 189–191 °C (EtOH/dry Et_2_O), R*_f_*_(*alox*)_ = 0.10 (MeOH), R*_f_*_(*RP-TLC*)_ = 0.07 (H_2_O/ACN 7:3). ^1^H NMR (600.11 MHz, DMSO-*d_6_*) *δ* (ppm): 2.36 (s, 3H, 4-CH_3_), 2.45 (s, 3H, 7-CH_3_), 5.71 (s, 1H, H_3_), 7.34–7.40 (m, 1H, H_6’_), 7.55 (t, 1H, *J* = 8.9 Hz, H_5’_), 7.68 (dd, 1H, *J*_1_ = 6.7 Hz, *J*_2_ = 2.6 Hz, H_2’_), 10.08 (s, 1H, 1-OH), 14.74 (s, 1H, 6-OH); ^13^C NMR (150.9 MHz, DMSO-*d_6_*) *δ* (ppm): 20.7 (7-CH_3_), 25.0 (4-CH_3_), 99.5 (C_5_), 110.5 (C_3_), 117.5, 117.6 (d, *J*_C-F_ = 22.3 Hz, C_5’_), 120.1, 120.2 (d, *J*_C-F_ = 18.9 Hz, C_3’_), 126.7 (C_6’_), 128.1 (C_2’_), 134.1 (C_1’_), 148.3 (C_4_), 155.3, 156.9 (d, *J*_C-F_ = 247.2 Hz, C_4’_), 158.8 (C_2_), 164.3 (C_6_), 168.8 (C_7_). Anal. Calcd for C_14_H_12_ClFN_2_O_3_: C, 54.12; H, 3.89; N, 9.02. Found: C, 54.16; H, 3.92; N, 9.08.

#### 3.2.9. Synthesis of *N*-hydroxypyridinedione Oximes (**30**–**32**)

General procedure:

5-acetyl-1,6-dihydroxy-4-methylpyridin-2(1H)-one (**B**) (0.55 mmol, 1 equiv.) is added to a solution of the appropriate hydroxylamine (0.57 mmol, 1.1 equiv.) in abs. EtOH (2 mL), and the reaction mixture is stirred at RT, under Argon, for 1.5–22 h. Thereafter, the solvent is evaporated under vacuum. The solid residue is triturated with Et_2_O under ice to afford the desired compound.

**5-(1-(((3,4-dichlorobenzyl)oxy)imino)ethyl)-1,6-dihydroxy-4-methylpyridin-2(1H)-one** (**30**)

To a solution of the *O*-(3,4-dichlorobenzyl)hydroxylamine (**9**) (330 mg, 1.64 mmol, 1.0 equiv.) in abs. EtOH (6 mL), 5-acetyl-1,6-dihydroxy-4-methylpyridin-2(1*H*)-one (**B**) (300 mg, 1.72 mmol, 1.05 equiv.) is added, and the reaction mixture is stirred at RT, under Argon, for 24h. Thereafter, the solvent is evaporated under vacuum. The solid residue is triturated with 2 mL Et_2_O under ice. The precipitate formed was filtered off in vacuo and chromatographed on a Sephadex LH-20 column eluted with MeOH. The green solid was triturated with dry Et_2_O and drops of EtOAc to afford [30] as a green crystalline solid (150 mg, 26%); mp 103–105 °C (AcOEt/dry Et_2_O), R*_f_*_(*NP-TLC*)_ = 0.10 (AcOEt), R*_f_*_(*RP-TLC*)_ = 0.03 (H_2_O/ACN 7:3); ^1^H NMR (600.11 MHz, DMSO-*d_6_*) *δ* (ppm) 1.76, 1.88 (s + s, 2.85H, 4-CH_3_), 1.93, 1.96 (s + s, 0.8H, 7-CH_3_), 1.99, 2.03 (s + s, 1.9H, 7-CH_3_), 4.96, 5.01 (s + s, 0.55H, OCH_2_3,4-Cl_2_C_6_H_4_), 5.09, 5.13 (s + s, 1.4H, OCH_2_3,4-Cl_2_C_6_H_4_), 5.48, 5.52 (s + s, 1H, H_3_), 7.28 (dd, 0.3H, *J*_1_ = 8.2 Hz, *J*_2_ = 2.0 Hz, H_6’_), 7.35 (dd, 0.7H, *J*_1_ = 8.2 Hz, *J*_2_ = 2.0 Hz, H_6’_), 7.54 (d, 0.3H, *J* = 2.0 Hz, H_2’_), 7.59 (d, 0.7H, *J* = 1.9 Hz, H_2’_), 7.59 (d, 0.3H, *J* = 8.3 Hz, H_5’_), 7.63 (d, 0.7H, *J* = 8.3 Hz, H_5’_), 10.18 (low), 11.60 (s + v br s, 1H, 1-OH, 6-OH); ^13^C NMR (150.9 MHz, DMSO-*d_6_*) *δ* (ppm) 15.9 (7-CH_3_), 19.3, 19.4 (4-CH_3_), 19.8 (7-CH_3_), 72.8 (OCH_2_3,4-Cl_2_C_6_H_4_), 91.0 (C_3_), 112.0 (C_5_), 127.8 (C_6’_), 129.5 (C_2’_), 129.9 (C_4’_), 130.4 (C_5’_), 130.8 (C_3’_), 139.8, 140.1 (C_1’_), 146.4, 149.5 (C_4_), 153.7, 155.0 (C_7_), 155.1 (C_6_), 156.4 (C_2_). Anal. Calcd for C_15_H_14_Cl_2_N_2_O_4_: C, 50.44; H, 3.95; N, 7.84. Found: C, 50.48; H, 4.04; N, 7.89.

**5-(1-(((2,4-dichlorobenzyl)oxy)imino)ethyl)-1,6-dihydroxy-4-methylpyridin-2(1H)-one** (**31**) was synthesized from compound (**10**) according to the general procedure. Green solid (93.6 mg, 50%), R*_f_* 0.20 (AcOEt), mp: 145–150 °C, ^1^H NMR (400 MHz, DMSO) δ 7.65–7.39 (m, 3H, Ar), 5.48 (d, *J* = 19.7 Hz, 1H, CHC=ON), 5.09 (d, *J* = 50.9 Hz, 2H, OCH_2_), 2.00 (d, *J* = 28.0 Hz, 3H, CH_3_C=N), 1.88 (d, *J* = 6.2 Hz, 3H, CH_3_). ^13^C NMR (101 MHz, DMSO) δ 132.50 (C_3’_), 129.22 (C_5’_), 128.28 (C_6’_), 92.19 (C_3_), 71.56 (OCH_2_), 20.42 (7-CH_3_), 16.42 (4-CH_3_). Anal. Calcd for C_15_H_14_Cl_2_N_2_O_4_: C, 50.44; H, 3.95; N, 7.84. Found: C, 50.48; H, 3.99; N, 7.88.

**1,6-dihydroxy-5-(1-(((2-iodobenzyl)oxy)imino)ethyl)-4-methylpyridin-2(1H)-one** (**32**), was synthesized from compound (**11**) (170 mg, 0.68 mmol) according to the general procedure**.** Green solid (99.5 mg, 39%), mp 131–133 °C (deco), R*_f_* = 0.07 (EtOAc/MeOH 3:1). ^1^H NMR (600 MHz, DMSO) δ 7.86 (dd, *J* = 19.2, 8.0 Hz, 1H, Ar), 7.45–7.34 (m, 2H, Ar), 7.17–6.99 (m, 2H, Ar), 5.39 (s, 1H, H_3_), 5.06 (s, 2H, –CH_2_-), 2.06 (s, 3H, 7-CH_3_), 1.88 (s, 3H, 4-CH_3_). ^13^C NMR (151 MHz, DMSO) δ 154.38 (C_2_), 153.09 (C_6_), 150.81 (C_7_), 136.73 (C_2‘_), 133.67 (C_3‘_), 135.54 (C_1‘_), 131.17 (C_4‘_), 128.10 (C_5‘_), 127.09 (C_6‘_), 125.58 (C_5_), 122.25 (C_3_), 91.19 (C_4_), 73.67 (–CH_2_-), 22.10 (4-CH_3_), 19.78 (7-CH_3_). Anal. Calcd for C_15_H_15_IN_2_O_4_: C, 43.50; H, 3.65; N, 6.76. Found: C, 43.51; H, 3.64; N, 6.78.

#### 3.2.10. Synthesis of N-hydroxypyridinedione Imines (**33**–**43**)

General procedure:

To a solution of **A** (1 eq) in absolute EtOH at 60 °C, the appropriate amine (1.1 eq) and activated 4Å molecular sieves were added. The mixture was refluxed overnight at 60 °C under Argon. After the completion of the reaction, the solvent is evaporated under vacuum. The solid residue is triturated with Et_2_O under ice to afford the desired compound as a solid.

**5-(1-((2-bromobenzyl)imino)ethyl)-1,6-dihydroxy-4-methylpyridin-2(1H)-one** (**33**) was synthesized from compound (**16**) (100 mg, 0.54 mmol) according to the general procedure. Green solid (130.2 mg, 76%)%). mp 140–142 °C, R*_f_* = 0.06 (EtOAc/MeOH 3:1). ^1^H NMR (400 MHz, DMSO) δ 7.71 (d, *J* = 7.9 Hz, 2H, -OH), 7.45 (d, *J* = 4.5 Hz, 2H, Ar), 7.37–7.27 (m, 2H, Ar), 5.58 (s, 1H, H_3_), 4.82 (s, 2H, H_10_), 2.35 (s, 2H, H_9_), 2.32 (s, 3H, 4-CH_3_), 2.16 (s, 1H, H_9_). ^13^C NMR (101 MHz, DMSO) δ 170.67 (C_2_), 163.86 (C_8_), 158.71 (C_6_), 148.04 (C_2’_), 135.60 (C_1’_), 133.02 (C_3’_), 130.18 (C_5’_), 130.05 (C_4’_), 128.37 (C_6’_), 123.02 (C_3_), 109.13 (C_5_), 101.25 (C_4_), 98.35 (C_10_), 25.24 (C_9_), 23.86 (C_4_). Anal. Calcd for C_15_H_15_BrN_2_O_3_: C, 51.30; H, 4.31; N, 7.98. Found: C, 51.35; H, 4.34; N, 7.99.

**5-(1-((3-bromobenzyl)imino)ethyl)-1,6-dihydroxy-4-methylpyridin-2(1H)-one** (**34**) was synthesized from compound (**17**) (100 mg, 0.54 mmol) according to the general procedure. Green solid (116.2 mg, 68%). mp 146-148 °C, R*_f_* = 0.06 (EtOAc/MeOH 3:1). ^1^H NMR (500 MHz, DMSO) δ 7.60 (s, 1H, Ar), 7.55 (s, 2H, Ar), 7.38 (s, 1H, Ar), 5.57 (s, 1H, H_3_), 4.80 (s, 2H, H_10_), 2.48 (s, 3H, H_9_), 2.31 (s, 3H, H_4_). ^13^C NMR (126 MHz, DMSO) δ 170.72 (C_2_), 163.80 (C_8_), 158.72 (C_6_), 148.03 (C_5’_), 139.56 (C_2’_), 131.06 (C_1’_), 130.64 (C_6’_), 130.32 (C_4’_), 126.59 (C_3’_), 122.04 (C_5_), 109.01 (C_4_), 98.35 (C_3_), 46.52 (C_10_), 25.18 (C_4_), 19.09 (C_9_). Anal. Calcd for C_15_H_15_BrN_2_O_3_: C, 51.30; H, 4.31; N, 7.98. Found: C, 51.32; H, 4.35; N, 8.00.

**5-(1-((4-bromobenzyl)imino)ethyl)-1,6-dihydroxy-4-methylpyridin-2(1H)-one** (**35**) was synthesized from compound (**18**) (103.8 mg, 0.56 mmol), according to the general procedure. Green solid (117.3 mg, 66%), mp 146–148 °C, R*_f_* = 0.06 (EtOAc/MeOH 3:1). ^1^H NMR (400 MHz, DMSO) δ 7.61 (d, *J* = 8.1 Hz, 2H, Ar), 7.51 (br, s, 2H, –OH), 7.39–7.23 (m, 2H, Ar), 5.57 (s, 1H, H_3_), 4.77 (s, 2H, H_10_), 2.35 (s, 1H, H_9_), 2.31 (s, 3H, H_7_), 2.16 (s, 2H, H_9_). ^13^C NMR (101 MHz, DMSO) δ 170.74 (C_2_), 163.79 (C_8_), 158.74 (C_6_), 148.03 (C_4’_), 136.23 (C_1’_), 131.78 (C_3’,5’_), 131.22 (C_2’,6’_), 129.75 (C_3_), 120.89 (C_5_), 108.96 (C_4_), 46.57 (C_10_), 25.18 (C_9_), 19.10 (C_7_). Anal. Calcd for C_15_H_15_BrN_2_O_3_: C, 51.30; H, 4.31; N, 7.98. Found: C, 51.36; H, 4.40; N, 7.99.

**5-(1-((2-chlorobenzyl)imino)ethyl)-1,6-dihydroxy-4-methylpyridin-2(1H)-one** (**36**) was synthesized from 2-chlorobenzylamine, (42.5 mg, 36.2 μL, 0.3 mmol, 1.1 eq) according to the general procedure. The solid residue was purified by MPLC method Column: FP Si 12g, Mode: Flash; Solvent A: EtOAc, Solvent B: MeOH; Gradient (%2nd: 0–30%; Flow Rate: 30 mL/min). The solid was then triturated with Et_2_O and EtOAc and filtered off under vacuum to afford the title compound as a yellow solid. (53.9 mg, 64.4%); m.p 116–118 °C, R*_f_* = 0.26 (EtOAc/MeOH 3:1), R*_f_*_(*RP-TLC*)_ = 0.66 (H_2_O/ACN 7:3). ^1^H NMR (400 MHz, DMSO-*d*_6_) δ 7.55 (dd, *J* = 5.7, 3.5 Hz, 1H,Ar), 7.44–7.39 (m, 1H,Ar), 7.36–7.24 (m, 2H, Ar), 5.58 (s, 1H, CHC=ON), 4.93 (d, *J* = 68.8 Hz, 2H,1’-CH_2_), 2.35 (s, 3H,4-CH_3_), 2.17 (s, 3H,7-CH_3_). ^13^C NMR (101 MHz, DMSO) δ 170.71 (C_7_), 161.18 (C_6_), 157.63 (C_2_), 148.82 (C_4_), 133.99 (C_1’_), 132.66 (C_2’_), 129.96 (Ar), 128.48 (Ar), 127.81 (Ar), 127.19 (Ar), 109.08 (C_3_), 98.35 (C_5_), 45.37 (1’-CH_2_), 25.22 (4-CH_3_), 23.82 (7-CH_3_). Anal. Calcd for C_15_H_15_ClN_2_O_3_: C, 58.73; H, 4.93; Cl, 11.56; N, 9.13; O, 15.65. Found: C, 60.00; H, 5.05; Cl, 11.58; N, 9.50; O, 15.75.

**5-(1-((3-chlorobenzyl)imino)ethyl)-1,6-dihydroxy-4-methylpyridin-2(1H)-one** (**37**) was synthesized from 3-chlorobenzylamine, (102.1 mg, 87 μL, 0.7 mmol, 1.1 eq) according to the general procedure. The solid residue was triturated with Et_2_O and n-pentane. The solid was filtered off under vacuum to afford the title compound as a brown solid. (137.40 mg, 68.4%); m.p 102–120 °C (dec.), R*_f_* = 0.27 (EtOAc/MeOH 3:1), R*_f_*_(*RP-TLC*)_ = 0.60 (H_2_O/ACN 7:3). ^1^H NMR (500 MHz, DMSO-*d*_6_) δ 7.48–7.25 (m, 4H,Ar), 5.57 (s, 1H,H_3_), 4.92 (d, *J* = 113.2 Hz, 2H,-1’-CH_2_), 2.48 (s, 1H,7-CH_3_), 2.33 (d, *J* = 22.1 Hz, 3H,4-CH_3_), 2.17 (s, 2H, 7-CH_3_). ^13^C NMR (126 MHz, DMSO-*d*_6_) δ 170.75 (C_7_),158.77 (C_2_), 148.92 (C_6_), 148.02 (C_4_), 133.45 (C_1’_), 130.79 (C_3’_), 127.75 (Ar), 127.43 (Ar), 127.27 (Ar), 126.22 (Ar), 109.00 (C_3_), 101.15 (1’-CH_2_), 98.39 (C_5_), 46.61 (1’-CH_2_), 32.50 (4-CH_3_), 25.18 (7-CH_3_), 23.81 (7-CH_3_), 19.10 (7-CH_3_). Anal. Calcd for C_15_H_15_ClN_2_O_3_: C, 58.73; H, 4.93; Cl, 11.56; N, 9.13; O, 15.65. Found: C, 60.00; H, 5.05; Cl,12.00; N, 9.50; O, 16.00.

**5-(1-((4-chlorobenzyl)imino)ethyl)-1,6-dihydroxy-4-methylpyridin-2(1H)-one** (**38**) was synthesized from 4-chlorobenzylamine, (102.1 mg, 87.7 μL, 0.7 mmol, 1.1 eq) according to the general procedure. The solid residue was triturated with Et_2_O and drops of EtOH. The solid was filtered off under vacuum to afford the title compound as a brown solid. (165.80 mg, 82.5%); m.p 115–118 °C, R*_f_* = 0.30 (EtOAc/MeOH 3:1), R*_f_*_(*RP-TLC*)_ = 0.66 (H_2_O/ACN 7:3). ^1^H NMR (400 MHz, DMSO-*d*_6_) δ 7.48 (d, *J* = 7.9 Hz, 2H, H_2’,6’_), 7.40 (d, *J* = 8.3 Hz, 2H, H_3’,5’_), 5.57 (s, 1H, H_3_), 4.79 (s, 2H, 1’-CH_2_), 2.48 (s, 2H, 7-CH_3_), 2.31 (s, 3H, 4-CH_3_), 2.16 (s, 1H, 7-CH_3_). ^13^C NMR (101 MHz, DMSO) δ 170.70 (C_7_), 158.71 (C_2_), 148.75 (C_6_) 147.99 (C_4_), 135.79 (C_4’_), 132.36 (C_1’_), 129.42 (C_2’,6’_), 128.84 (C_3’,5’_), 108.93 (C_3_), 98.32 (C_5_), 46.51 (1’-CH_2_), 25.15 (4-CH_3_), 19.08 (7-CH_3_). Anal. Calcd for C_15_H_15_ClN_2_O_3_: C, 58.73; H, 4.93; Cl, 11.56; N, 9.13; O, 15.65. Found: C, 59.00; H, 5.00; Cl,12.05; N, 9.50; O, 16.00.

**5-(1-((3-fluorobenzyl)imino)ethyl)-1,6-dihydroxy-4-methylpyridin-2(1H)-one** (**39**) was synthesized from 3-fluorobenzylamine, (52.6 mg, 47.9 μL, 0.42 mmol, 1.1 eq) according to the general procedure. The solid residue was triturated with Et_2_O and drops of EtOH. The solid was filtered off under vacuum to afford the title compound as a brown solid. (53.3 mg, 48.4%). R*f* = 0.13(EtoAc/MeOH 3:1). mp. 138–140 °C. ^1^H NMR (500 MHz, DMSO) δ 7.47 (q, *J* = 7.3 Hz, 1H), 7.22 (d, *J* = 8.3 Hz, 2H), 7.17 (d, *J* = 8.8 Hz, 1H), 5.57 (s, 1H), 4.81 (s, 2H), 2.35 (s, 1H), 2.31 (s,2H), 2.16 (s, 3H). ^13^C NMR (126 MHz, DMSO) δ 170.75 (C_8_), 163.79 (C_17_), 161.35(C_19_), 158.71 (C_2_), 148.75 (C_4_), 139.65 (C_15_), 130.97 (C_18_), 123.52 (C_20_),115.42 (C_16_),108.97(C_1_), 101.22 (C_5_), 98.35 (C_6_), 46.68 (C_14_), 25.76 (8-CH_3_), 25.16 (6-CH_3_). Anal. Calcd (%) for C_15_H_15_FN_2_O_3_: C, 62.06; H, 5.21; F, 6.54; N,9.65; O, 16.53. Found: C, 61.97; H, 5.24; F, 6.40; N, 9.59; O, 16.63.

**5-(1-((4-fluorobenzyl)imino)ethyl)-1,6-dihydroxy-4-methylpyridin-2(1H)-one** (**40**) was synthesized from 4-fluorobenzylamine, (74.53 mg, 68 μL, 0.3 mmol, 1.1 eq) according to the general procedure. The solid residue was triturated with Et_2_O and drops of EtOH. The solid was filtered off under vacuum to afford the title compound as a brown solid. (74.6 mg, 43%); m.p. 138–140 °C, R*f* = 0.15 (EtOAc/MeOH 3:1). ^1^H NMR (400 MHz, DMSO) δ 7.28–7.20 (m, 4H), 5.56 (s, 1H), 4.76 (s,2H), 2.35 (s, 2H), 2.31 (s, 1H), 2.16 (s, 3H,). ^13^C NMR (101 MHz, DMSO) δ 170.87 (C_11_), 163.52 (C_18_), 157.79 (C_2_), 149.94 (C_6_), 148,91 (C_4_), 133.28 (C_15_), 129.75 (C_16–20_), 129.67 (C_17–19_), 115.52 (C_15_), 108.71 (C_1_), 101.09 (C_5_), 98.93 (C_6_), 48.50(C_14_), 25.05 (11-CH_3_), 23.68 (6-CH_3_)., Anal. Calcd (%) for C_15_H_15_FN_2_O_3_: C, 62.06; H, 5.21; F, 6.54; N, 9.65; O, 16.53. Found: C, 61.97; H, 5.29; F, 6.42; N, 9.61; O, 16.60.

**5-(1-((3,4-difluorobenzyl)imino)ethyl)-1,6-dihydroxy-4-methylpyridin-2(1H)-one** (**41**) was synthesized from 3,4-difluorobenzylamine, (103.2 mg, 85.3 μL, 0.7 mmol, 1.1 eq) according to the general procedure. The solid residue was triturated with Et_2_O and drops of EtOH. The solid was filtered off under vacuum to afford the title compound as a brown solid. (101 mg, 50%); m.p 102–107 °C, R*_f_* = 0.19 (EtOAc/MeOH 3:1), R*_f_*_(*RP-TLC*)_ = 0.28 (H_2_O/ACN 7:3). ^1^H NMR (400 MHz, DMSO-*d*_6_) δ 7.51–7.45 (m, 1H,H_6’_), 7.39–7.32 (m, 1H,H_5’_), 7.24 (s, 1H,H_2’_), 5.57 (s, 1H,H_3_), 4.78 (s, 2H,1’-CH_2_), 2.48 (s, 3H,7-CH_3_), 2.31 (s, 3H,4-CH_3_). ^13^C NMR (101 MHz, DMSO) δ 170.89 (C_7_), 163.96 (C_6_), 158.92 (C_2_), 148.19 (C_4_), 134.78 (C_1’_), 124.66 (C_2’_), 118.22 (C_4’_), 118.05 (C_3’_), 117.13 (C_6’_), 116.95 (C_5’_), 109.16 (C_3_), 98.62 (C_5_), 46.37 (1’-CH_2_), 25.33 (4-CH_3_), 19.28 (7-CH_3_). Anal. Calcd for C_15_H_14_F_2_N_2_O_3_: C, 58.44; H, 4.58; F, 12.33; N, 9.09; O, 15.57. Found: C, 59.00; H, 5.60; F,12.35; N, 9.15; O, 15.65

**5-(1-((3,5-difluorobenzyl)imino)ethyl)-1,6-dihydroxy-4-methylpyridin-2(1H)-one** (**42**) was synthesized from 3,5-difluorobenzylamine, (41.3 mg, 34.1 μL, 0.3 mmol, 1.1 eq) according to the general procedure. The solid residue was triturated with Et_2_O and drops of EtOH. The solid was filtered off under vacuum to afford the title compound as a brown solid. (64.3mg, 80.2%); m.p 136–140 °C, R*_f_* = 0.24 (EtOAc/MeOH 3:1), R*_f_*_(*RP-TLC*)_ = 0.60 (H_2_O/ACN 7:3). ^1^H NMR (400 MHz, DMSO-*d*_6_) δ 7.40–6.99 (m, 3H, Ar), 5.58 (s, 1H, H_3_), 4.92 (d, *J* = 75.5 Hz, 2H, 1’-CH_2_), 2.33 (d, *J* = 15.1 Hz, 3H, 4-CH_3_), 2.19–2.04 (m, 3H, 7-CH_3_). ^13^C NMR (101 MHz, DMSO) δ 171.43 (C_7_), 164.30 (C_6_), 158,66 (C_2_) 148.55 (C_4_), 131.88 (C_1’_), 111.05 (Ar), 109.61 (C_3_), 103.96 (Ar), 103.70 (Ar), 103.45 (Ar), 98.99 (C_5_), 46.81 (1’-CH_2_), 25.64 (7-CH_3_), 19.55 (4-CH_3_). Anal. Calcd for C_15_H_14_F_2_N_2_O_3_: C, 58.44; H, 4.58; F, 12.33; N, 9.09; O, 15.57. Found: C, 59.05; H, 5.65; F,12.40; N, 9.13; O, 15.70.

**5-(1-((2-(4-chlorophenoxy)ethyl)imino)ethyl)-1,6-dihydroxy-4-methylpyridin-2(1H)-one** (**43**) was synthesized from 2-(4-chlorophenoxy)ethan-1-amine (**20**), (101 mg, 0.58 mmol, 1.2 eq) according to the general procedure. The solid residue was triturated with Et_2_O, EtOAc, and drops of EtOH. The solid was filtered off under vacuum to afford the title compound as a red solid. (30 mg, 19.6%) R*f* = 0.27 (EtOAc/MeOH 3:1). mp. 138–140 °C. ^1^H NMR (400 MHz, DMSO) δ 7.35 (dd, *J* = 132.1, *J* = 8.6 Hz, 2H), 7.03 (dd, *J* = 132.1, 9.0 Hz, 2H), 5.55 (s, 1H), 4.24 (q, *J* = 5.0 Hz, 2H), 3.91 (q, *J* = 5.0 Hz,2H), 2.35 (s, 2H), 2.31 (s, 1H), 2.16 (s, 3H). ^13^C NMR (101 MHz, DMSO) δ 171.49 (C_11_), 159.7 (C_2_), 157.43 (C_17_),148.52 (C_4_),129.81 (C_18–22_), 125.31 (C_20_), 116.97 (C_19–21_), 108.95 (C_1_), 101.64 (C_5_), 98.59 (C_6_), 66.83 (C_15_), 43.63 (C_14_), 25.66 (11-CH_3_), 24.33 (6-CH_3_). Anal. Calcd (%) for C_16_H_17_ClN_2_O_4_: C, 57.06;H, 5.09; Cl, 10.53; N, 8.32; O, 19.00. Found: C, 57.01; H, 5.02; Cl, 10.48; N,8.31; O, 19.13.

#### 3.2.11. Synthesis of Minoxidil Derivative (**44**)

General procedure:

To a solution of the corresponding 6-substituted 2,4-diaminopyrimidine intermediate (700 mg, 2.49 mmol, 1 equiv.) in MeOH (6.2 mL), a solution of mCPBA (1.1 g, 6.23 mmol, 2.5 equiv.) in MeOH (5 mL) is added dropwise over a time period of 1–1.5 h at 0 °C. The reaction mixture is stirred at 0 °C overnight. Then, aq. NaOH 6 M is added until basic pH = 8. The organic solvent is evaporated under vacuum, and the formed white solid precipitate is filtered under vacuum and washed with ice-cooled water (1 mL). The aqueous filtrate is extracted with EtOAc (4 × 100 mL). The combined organic layers are washed with brine (3 × 50 mL), then dried over anh. Na_2_SO_4_, filtered, and concentrated. The resulting crude oil is purified using flash column chromatography (EtOAc/MeOH, 0–30%). The oil product was then triturated with Et_2_O to afford the product as a white solid.

**2-amino-4-(2-bromophenoxy)-6-iminopyrimidin-1(6H)-ol** (**44**) was synthesized according to the general procedure from the 6-(2-bromophenoxy)pyrimidine-2,4-diamine (**19**), to afford a white solid (250.2 mg, 50%) R*_f_* = 0.30 (3:1 AcOEt: MeOH), mp > 250 °C (dec.: 200 °C), ^1^H NMR (500 MHz, DMSO) δ 7.71 (d, *J* = 7.9 Hz, 1H, Ph), 7.44 (t, *J* = 7.8 Hz, 2H, Ph, OH), 7.30 (d, *J* = 8.1 Hz, 1H, Ph), 7.20 (t, *J* = 7.7 Hz, 1H, Ph), 5.50 (s, 1H, CHC=NH). ^13^C NMR (126 MHz, DMSO) δ 158.72 (C_4_), 154.31 (C_6_), 152.58 (C_2_), 150.04 (C_1’_), 133.45 (C_3’_), 129.27 (C_5’_), 127.14 (C_4’_), 124.17 (C_6’_), 115.82 (C_2’_), 76.92 (C_5_). HRMS/ESI+ (m/z): Calcd for C_10_H_9_BrN_4_O_2_: 295.9909; Found: 296.9979. Anal. Calcd for C_10_H_9_ BrN_4_O_2_: C, 40.43; H, 3.05; N, 18.86. Found: C, 40.46; H, 3.08; N, 18.89.

#### 3.2.12. Synthesis of 5-Acetyl Barbituric Acid

Acetic anhydride (23.4 mL) was used to suspend barbituric acid (1.0 g, 7.81 mmol), then 7 drops of concentrated H_2_SO_4_ were added. The barbituric acid was dissolved completely after 10 min, resulting in a yellow–brown solution. The reaction mixture was stirred at 110 °C for 1.5 h. Thereafter, the mixture was concentrated to half its volume and cooled down to 0 °C. The formed precipitate was filtered off and washed with hot water and acetone to afford a beige crystalline solid (1.15 g, 89%). ^1^H NMR (400 MHz, DMSO) *δ* 11.77 (s, 1H), 11.05 (s, 1H,), 2.58 (s, 3H) ppm [35].

#### 3.2.13. Synthesis of Barbituric Acid Analogue (**45**)

**5-(1-(((2-chlorobenzyl)oxy)imino)ethyl)pyrimidine-2,4,6(1H,3H,5H)-trione** (**45**)

5-Acetyl-barbituric acid (0.386 mmol, 1 equiv.) is suspended in abs. EtOH (2 mL) at ~90 °C, and molecular sieves and the appropriate hydroxylamine (**12**) (82.2 mg, 0.52 mmol, 1.1 eq) are added. The mixture is refluxed for 3 days at 70 °C under an Argon atmosphere. The suspended solid formed is filtered under vacuum and washed with Et_2_O and EtOH. The molecular sieves are removed to afford the desired product as a pink solid. (122 mg, 83%). mp >250 °C, R*_f_* = 0.26 (EtOAc/MeOH 3:1). ^1^H NMR (400 MHz, DMSO) δ 10.58 (s, 1H, H_5_), 7.61–7.52 (m, 2H, Ar), 7.49–7.38 (m, 2H, Ar), 5.20 (s, 2H, –CH_2_-), 2.54 (s, 3H, H_8_). ^13^C NMR (125 MHz, DMSO) δ 167.28 (C_4,6_), 161.29 (C_7_), 151.41 (C_2_), 133.87 (C_1’_), 133.51 (C_2’_), 129.94 (C_6’_), 129.42 (C_3’_), 128.88 (C_4’_), 127.49 (C_5’_), 75.31 (C_9_), 54.39 (C_5_), 19.20 (C_8_). Anal. Calcd for C_13_H_12_ClN_3_O_4_: C, 50.42; H, 3.91; N, 13.57. Found: C, 50.44; H, 3.92; N, 13.55.

### 3.3. Cells and Cell Culture

HepDES19 cells are a HepG2 cell-line derivative that has been stably transfected with an HBV genotype D genome containing a tetracycline repressible promoter [36]. HepDES19 cells were maintained on collagen-coated plates in Dulbecco’s modified Eagle’s medium (DMEM/F12) (Cytiva Life Sciences, Marlborough, MA, USA) supplemented with 10% fetal bovine serum (FBS), streptomycin (100 µg/mL), and penicillin (100 IU/mL), and incubated at 37 °C with 5% CO_2_ and saturating humidity. Cells were cultured with 1 µg/mL tetracycline to inhibit the expression of the stably integrated HBV genome, and HBV replication was activated upon removal of tetracycline from the culture medium.

### 3.4. qPCR HBV Replication Inhibition Assay

HBV replication inhibition in HepDES19 cells was assessed using a strand-preferential quantitative PCR assay as previously described [16,37]. HepDES19 cells were seeded in 96 well plates at 4 × 10^4^ cells/well for 48 h followed by the addition of serial diluted compound with a final concentration of 1% DMSO for 72 h, after which cells were lysed, and qPCR was performed as described in Li et al. [37]. Effective concentration 50% (EC_50_) values were calculated from data for the (+) polarity DNA strand data using GraphPad Prism version 10’s four-parameter log (inhibitor) versus response algorithm, with the bottom value set to zero. Three or more replicate assays were performed on different days.

### 3.5. MTS Cytotoxicity Assay

Cell viability was assessed using the CellTiter 96^TM^ Aqueous Non-Radioactive Cell Proliferation assay (MTS) (Promega, Madison, WI, USA) as described previously [16]. HepDES19 cells were seeded in 96 well plates at 1 × 10^4^ cells/well for 48 h. Cells were then incubated with serial diluted compound, with a final concentration of 1% DMSO, for 72 h. Following compound incubation, background absorbance values were subtracted, and the data were converted into percent cell viability. The cytotoxic concentration 50% (CC_50_) values were calculated using GraphPad Prism’s four-parameter log (inhibitor) versus response algorithm, with the bottom value set to zero. Three or more replicate assays were performed on different days.

### 3.6. Solubility Limit Assay

Solubility limits of compounds were tested as previously reported [16] in DMEM-F12 without phenol red (Gibco, Grand Island, NY, USA) but with the media supplemented with 10% FBS (pH 7.4) to mimic cell culture experiments. In a 384-well optically clear plate, compounds were serially diluted in pH 7.4 buffer (upper limit 200 µM, DMSO 1%) then read at 620 nm. Solubility limits were determined by plotting compound concentration (µM) against optical density (OD) and identifying an inflexion point. The inflexion point is defined by a substantial increase in OD brought on by increasing turbidity, and the compound concentration at this point is known as the solubility limit [22]. Each compound had at least two replicate assays performed on different days.

### 3.7. Parallel Artificial Membrane Permeability Assay

The evaluation of apparent passive permeability (P_app_) at pH 7.4 was performed using a donor/acceptor cassette (Innovative Laboratory Products, Phoenix, AZ, USA and Sigma Aldrich, Burlington, MA, USA), which simulates the apical and basolateral sides of the small intestine epithelium, as previously described [38]. An artificial membrane made of 1% *w*/*v* lecithin/dodecane was applied to the poly (vinylidene fluoride) (PVDF) membrane filter and allowed to dry. Following drying, compounds (200 µM) were diluted in buffer at pH 7.4 and added to the donor side of the cassette. The same pH buffer was added on the acceptor side of the cassette, after which it was assembled and incubated at room temperature with shaking at 250 rpm for 2 h, before reading the absorbance of 100 µL from the acceptor well on a plate reader between 230 and 700 nm. The P_app_ (cm/s) values for compounds were determined by normalizing to the compound absorbance at equilibrium using the peak absorbance point for each compound, buffer volume, incubation time, and membrane porosity [10,39]. Two or more replicate assays were conducted on different days.

### 3.8. Systematic Conformational Search, Boltzmann Population Analysis, and EC_50_ Modelling of Oximes ***30***–***32***

All compounds prior to the conformational search were minimized in Macromodel [40,41] (Schrödinger 2017-1 platform, Schrödinger, LLC, New York, NY, USA, 2017) using the OPLS3e forcefield [42] in gas phase (ε = 1) and an RMS-gradient of 0.0001 kcal/mol Å. The systematic search was conducted in Macromodel using the Systematic Pseudo Monte Carlo (SPMC) method [43].

The conformational search was conducted in OPLS3e and the potential treatment was set according to the dielectric standard of DMSO (ε = 46.68) to simulate the NMR measurements. The convergence threshold for minimization was set at 0.001 kcal/mol and the maximum iteration limit was set at 2500. Miscellaneous technical characteristics: maximum number of steps—1000; steps per rotational bond—10,000; energy window for saving structures—21 kJ·mol^−1^.

For each conformer, the relative potential energy (ΔU, kJ·mol^−1^) was taken with respect to the global minimum, and Boltzmann weights were computed as

$$w_{i}=exp\left(-\frac{\Delta U_{i}}{RT}\right),\;R=8.314\times 10^{-3}\;kJ\cdot {mol}^{-1}K^{-1},\;T=298\;K$$

Normalized populations were obtained as $p_{i}=w_{i}/\sum_{j}w_{j}$. The active diastereomer (*E*) cumulative fraction was then calculated as

$$f_{E}=\sum_{i\in E}p_{i}$$

Per conformer RMS derivative values generated by Macromodel were used as σΔU_i_ to propagate uncertainties into *f*_E_. Uncertainty in each weight was estimated as

$$\sigma_{w_{i}}=\frac{w_{i}}{RT}\sigma \left(\Delta U_{i}\right)$$

and propagated to *f*_E_ using standard linear error propagation

$$\sigma_{f_{E}}^{2}=\frac{\sum_{i}{\left(\delta_{i,E}-f_{E}\right)}^{2}\sigma_{w_{i}}^{2}}{{\left(\sum_{j}w_{j}\right)}^{2}}$$

Experimental EC_50_ values (µM, ±SD) were combined with population uncertainties to derive intrinsic and predicted potencies. For each compound, the intrinsic potency of the active *E*-isomer was estimated as

EC_50_^E^ = EC_50_^mix^ × *f*_E_

with uncertainty propagation from both experimental EC_50_ error and computed σ*f*_E_. Although compounds **30** and **32** show weaker overall activity, their implied intrinsic potencies for *E* overlap with compound **31**, suggesting that the apparent differences arise from varying proportions of *E* and *Z* rather than intrinsic activity differences.

Using compound **31** as a reference (pure E by NMR), predicted mixture activities were calculated as

EC_50_^pred^ = EC_50_^Ref_E^/*f*_E_

For compounds **30** and **32**, predicted EC_50_^pred^ values matched the observed values within uncertainty, demonstrating that the model quantitatively accounts for the effect of inactive *Z* on apparent potency.

Additional diagnostics included cumulative *E*/*Z* population curves (cf. Figure S4), conformer counts within ΔU ≤ 3 and 5 kJ·mol^−1^, sensitivity analyses with global ±1 and ±2 kJ·mol^−1^ energy shifts (cf. Table S4), and RMS analysis (cf. Table S5).

### 3.9. Docking Studies

The HBV P protein structural model with incorporated Mg^2+^ ions into the active sites of RT and RNase H domains was generated with Alphafold3. The accuracy of the Mg^2+^ ions placement was checked by superimposing co-crystal structures (PDBs: 5J2M, 3K2P) of the HIV RT and RNase H domains with Mg^2+^ ions [44,45]. The Ligprep programme and Protein Preparation Wizard (Schrödinger LLC) were used to prepare ligands and protein by using the same setting as described previously [10]. Energy minimizations of ligands and protein were performed with the OPSL4 force field. The different protonation states of the ligands were produced at pH 7.5 ± 2 using Epik (classic); this added additional metal binding states, retained original chirality, and desalted and tautomerized the compounds. The HBV P model containing Mg^2+^ ions was assigned appropriate ionization states, and the protein protonated at pH 7.5 ± 2; hydrogen bonds were assigned using PROPKA (Schrödinger LLC). To analyze binding poses of the compounds into the RNase H active site, induced fit docking (IFD) was performed (Schrödinger LLC). The receptor grid of 10 Å for compounds docking was defined around the RNase H active site by placing β-thujaplicinol via superposition of the active site from the HIV RNase H co-crystal (PDB: 3K2P) onto the HBV P model. The settings for protein and ligand refinement were same as used in the previous study [10]. A total of 20 poses were retained in the initial docking, residues were refined within 5.0 Å of the ligand poses, and redocking was performed using SP (standard precision) programme with the best structures within 30.0 kcal/mol and the top 20 overall structures.

## 4. Conclusions

This study is part of our ongoing effort to develop safe and effective HBV RNase H inhibitors, focusing on the structural optimization of the HPD (1,6-dihydroxy-pyridin-2-one) pharmacophore. Our strategy centred on systematically exploring the impact of halogen substitution patterns on the side aromatic moiety. To this end, we designed and synthesized a focused library of 23 novel compounds, including 17 HPD imines, 4 HPD oxime analogues, one minoxidil-based derivative, and one barbituric acid analogue. Their antiviral activity was assessed in HepDES19 cells.

Among the synthesized compounds, 21 (compounds **21**–**43**) demonstrated notable anti-HBV activity, ranging from low micromolar to submicromolar IC_50_ values, while only two compounds (**44** and **45**) were inactive. Importantly, 6 compounds surpassed the selectivity index (SI) of our previously identified HPD imine hit compound **54** [26], with compound **22** exhibiting an SI more than five times higher than **54**, highlighting the HPD’s promising therapeutic potential.

The SAR findings derived from this series the following provide critical insights:

(i) The oxygen trident motif is essential for Mg^2+^ chelation, a key feature shared with other RNase H inhibitors. However, HPD scaffolds show superior drug-like properties compared to other chelators such as barbituric acid and minoxidil derivatives, including enhanced cell permeability, aqueous solubility, and minimal off-target effects.
(ii) In agreement with previous findings in [10], HPD oximes consistently outperformed their imine counterparts in terms of potency and selectivity.
(iii) The linker length significantly influenced antiviral activity: extending the aliphatic chain beyond one carbon atom resulted in a marked drop in SI, suggesting reduced compatibility with the lipophilic cavity of the target site (e.g., compound **43**).
(iv) Regarding halogenated aromatic substitutions, the preservation of the aromatic core was critical for activity. *Ortho*-substitution conferred superior potency among the imine series compared to *meta* or *para* positions, while combined *ortho*/*meta* or *ortho*/*para* disubstitutions generally reduced efficacy. Furthermore, halogens with smaller to medium atomic radii (e.g., F, Cl) were better tolerated than larger ones (e.g., Br, I).
(v) The predominant diastereomer of oximes **30**, **31**, and **32** is the *E*-isomer, which appears to possess higher biological activity.

Collectively, these findings expand the current understanding of the structure–activity relationships within the HPD scaffold and provide a robust framework for further lead optimization. This work not only identifies promising new anti-HBV candidates but also reinforces the HPD core as a privileged scaffold for targeting HBV RNase H.

## Figures and Tables

**Table 1:** Compound structures, physical properties, efficacy, and cytotoxicity.

a/a	Structure	logP ^1^	tPSA ^2^	Docking Score ^3^	EC_50_^4^	CC_50_^4^	SI ^5^
**21**	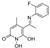	1.49	73.13	−9.68	2.2 ± 0.5	86.3 ± 10.2	39.2
**22**	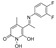	1.64	73.13	−9.01	0.89 ± 0.4	100.0 ± 0	112.4
**23**	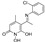	1.89	73.13	−9.38	2.42 ± 1.0	76.8 ± 5.9	31.7
**24**	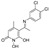	2.44	73.13	−9.21	2.17 ± 0.8	69.0 ± 0	31.8
**25**	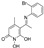	2.16	73.13	−8.48	4.53 ± 2.5	45.8 ± 4.6	10.1
**26**	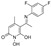	1.64	73.13	−9.49	2.83 ± 1.1	54.1 ± 27.7	19.1
**27**	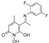	1.64	73.13	−10.03	1.97 ± 0.6	58.4 ± 21.9	29.7
**28**	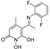	1.64	73.13	−9.87	2.73 ± 1.0	52.6 ± 20.5	19.3
**29**	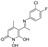	2.04	73.13	−9.35	3.25 ± 1.0	70.8 ± 18.4	21.8
**30**	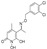	2.71	82.36	−10.53	1.77 ± 0.7	49.7 ± 16.4	28.2
**31**	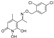	2.71	82.36	−10.48	0.53 ± 0.3	34.4 ± 14.1	65.1
**32**	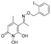	2.95	82.36	−9.64	1.77 ± 0.5	54.1 ± 13.7	30.5
**33**	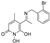	2.23	73.13	−7.53	8.45 ± 3.5	51.3 ± 20.9	6.1
**34**	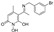	2.23	73.13	−9.58	13.6 ± 2.8	22.6 ± 16.7	1.7
**35**	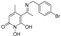	2.23	73.13	−9.38	15.0 ± 10.7	44.4 ± 34.4	3.0
**36**	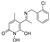	1.96	73.13	−9.77	2.77 ± 0.8	52.7 ± 29.3	19.0
**37**	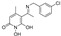	1.96	73.13	−9.61	4.73 ± 4.4	34.0 ± 23.0	7.2
**38**	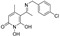	1.96	73.13	−10.12	18.9 ± 8.4	22.9 ± 7.0	1.2
**39**	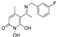	1.56	73.13	−10.24	27.6 ± 3.7	19.3 ± 10.5	0.7
**40**	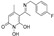	1.56	73.13	−9.75	16.5 ± 3.5	15.4 ± 7.4	0.9
**41**	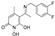	1.71	73.13	−9.93	10.7 ± 0.5	23.9 ± 11.7	2.2
**42**	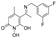	1.71	73.13	−9.85	73.7 ± 26.3	82.9 ± 24.6	1.1
**43**	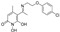	1.89	82.36	−9.61	17.2 ± 6.6	30.1 ± 12.5	1.8
**44**	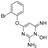	−	94.93	−8.72	100 ± 0.0	100 ± 0.0	1.0
**45**	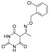	1.07	98.86	−6.44	100 ± 0.0	100 ± 0.0	1.0
**46** [22]	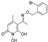	2.42	82.36	−10.3	2.00 ± 1.6	80.8 ± 27.5	40.0
**47** [22]	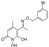	2.42	82.36	−9.9	0.52 ± 0.33	78.5 ± 32.4	151
**48** [22]	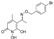	2.42	82.36	−9.2	1.13 ± 0.19	76.7 ± 30.3	67.9
**49** [22]	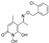	2.15	82.36	−9.8	0.24 ± 0.09	58.2 ± 29.1	243
**50** [22]	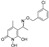	2.15	82.36	−9.6	1.35 ± 1.03	56.9 ± 31.1	42.1
**51** [22]	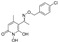	2.15	82.36	−9.9	0.36 ± 0.24	46.6 ± 13.6	130
**52** [16]	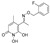	1.75	82.36	−9.8	0.32 ± 0.2	90.0 ± 6.0	281
**53** [22]	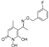	1.75	82.36	−10.0	0.06 ± 0.01	73.9 ± 28.6	1231
**54** [26]	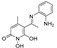	1.33	73.13	−9.9	0.69 ± 0.2	15 ± 7	22

^1^ LogP (<5) is the log of the partition coefficient of a solute between octanol and water. Predicted with FAF4 online server [27]. ^2^ tPSA (<140 Å2) is the Topological Polar Surface Area (Å2). ^3^ Induced fit docking score to the HBV RNase H active site; Kcal/mol. ^4^ pH 7.4; values in µM. ^5^ CC_50_/EC_50._

**Table 2:** Compound solubility limits and apparent passive permeability at pH 7.4.

	Solubility Limit ^1^		P_app_^2^	
Compound No	-	Interpretation	cm/s	Interpretation
**21**	200	H	5.72 × 10^−6^	H
**22**	200	H	2.99 × 10^−6^	H
**23**	200	H	2.83 × 10^−6^	H
**24**	200	H	2.24 × 10^−6^	H
**25**	200	H	3.12 × 10^−6^	H
**26**	200	H	3.81 × 10^−6^	H
**27**	200	H	2.40 × 10^−6^	H
**28**	200	H	2.48 × 10^−6^	H
**29**	200	H	3.65 × 10^−6^	H
**30**	200	H	1.59 × 10^−6^	H
**31**	200	H	4.00 × 10^−6^	H
**32**	100	H	3.29 × 10^−6^	H
**33**	200	H	1.22 × 10^−6^	H
**34**	200	H	1.11 × 10^−6^	H
**35**	200	H	3.15 × 10^−6^	H
**36**	166.7	H	3.04 × 10^−6^	H
**37**	200	H	5.41 × 10^−6^	H
**38**	200	H	3.07 × 10^−6^	H
**39**	200	H	1.89 × 10^−6^	H
**40**	200	H	1.10 × 10^−6^	H
**41**	200	H	2.54 × 10^−6^	H
**42**	200	H	1.53 × 10^−6^	H
**43**	200	H	8.60 × 10^−7^	L

^1^ Values are in µM. H, Highly soluble (>100 µM). ^2^ H: High rate of apparent passive permeability (>1 × 10^−6^ cm/s); L: Low rate of apparent passive permeability (<1 × 10^−6^ cm/s).

**Table 3:** Summary of diastereomeric populations and EC_50_ modelling of **30–32**.

Compound	*f*_E_ (Boltzmann, 298 K) ^1^	EC_50_^mix^ (μM) ^2^	EC_50_^E^ (μΜ) ^3^	EC_50_^pred^ (μM) ^4^	NMR Observation ^5^
**30**	0.87 ± 0.01	1.77 ± 0.70	1.54 ± 0.65	0.61 ± 0.35	Mixture (2.5–3:1)
**31**	0.86 ± 0.01	0.53 ± 0.30	0.46 ± 0.27	0.62 ± 0.35 *	Single diastereomer
**32**	0.74 ± 0.01	1.77 ± 0.50	1.31 ± 0.46	0.72 ± 0.41	Mixture (1.8–2:1)

^1^ *f*_E_: *E* population fraction from normalized Boltzmann analysis with uncertainty propagated from conformer RMS values. ^2^ EC_50_^mix^: Experimental potencies. ^3^ EC_50_^E^: Implied intrinsic potency of *E* estimated from EC_50_^E^ = EC_50_^mix^ × *f*_E_. ^4^ EC_50_^pred^: Expected mixture potency using **31** as pure-*E* reference, EC_50_^pred^ = EC_50_^Ref_E^/*f*_E_. ^5^ NMR observation: Qualitative *E*/Z ratios consistent with computed populations. * Predicted mixture potency for **31** equals the reference since it is pure *E*.

## Data Availability

The original contributions presented in this study are included in the article/Supplementary Material. Further inquiries can be directed to the corresponding author.

## Supplementary Materials

The following supporting information can be downloaded at https://www.mdpi.com/article/10.3390/ijms262010239/s1.
