# Supplementary material for: Discovery of N-Hydroxypyridinedione-Based Inhibitors of HBV RNase H: Design, Synthesis, and Extended SAR Studies
Source: Int J Mol Sci. 2025 Oct 21;26(20):10239. doi: 10.3390/ijms262010239 (PMC12564302; doi:10.3390/ijms262010239)
Supplement: Supplementary file 1 [file ijms-26-10239-s001.zip › ijms-3831128-supplementary.pdf]

Supporting Information

Discovery of *N*-Hydroxypyridinedione-Based Inhibitors of HBV RNase H: Design, Synthesis and Extended SAR Studies

Dea Chotzalli [1,‡], Vasiliki Pardali [1,‡], Holly M. Walden [2,3], Dimitrios Perivolaris [1], Dimitrios Moianos [1], Maria Makri [1], Antonios Drakopoulos [4], Erofili Giannakopoulou [1], Razia Tajwar [2,3], Molly E. Woodson [2,3], John E. Tavis [2,3] and Grigoris Zoidis [*,1]

[1] Department of Pharmacy, Division of Pharmaceutical Chemistry, School of Health Sciences, National and Kapodistrian University of Athens, Panepistimiopolis Zografou, 15771 Athens, Greece; chotzallidea@gmail.com (D. C.); pharmacy22pard@gmail.com (V.P.); perivolarisdimitrios@gmail.com (D.P.); moianosjim@gmail.com (D.M.); mariamakri1@hotmail.com (M.M.); evgian@pharm.uoa.gr (E.G.)

[2] Molecular Microbiology and Immunology, Saint Louis University School of Medicine, Saint Louis, MO 63104, United States; molly.woodson@discovery.eurofinsus.com (M.W.); razia.tajwar@health.slu.edu (R.T.); holly.furby@slu.edu (H.W.); john.tavis@health.slu.edu (J.E.T.)

[3] Saint Louis University Institute for Drug and Biotherapeutic Innovation, Saint Louis University, Saint Louis, MO 63104, United States molly.woodson@slu.edu (M.W.); razia.tajwar@health.slu.edu (R.T.); holly.furby@slu.edu (H.W.); john.tavis@health.slu.edu (J.E.T.)

[4] Department of Chemistry and Molecular Biology, University of Gothenburg, Göteborg, Sweden; drakopoulosantonis@gmail.com (A.D.)

‡ These authors contributed equally to this work.

* Correspondence: zoidis@pharm.uoa.gr; https://orcid.org/0000-0002-9442-5186, Tel.: +30-210-7274809

List of Contents

**I. Copies of NMR spectra**	**4**
NMR spectra of compound **21** (^{1}H, ^{13}C, COSY, HSQC-DEPT, HMBC)	4
NMR spectra of compound **22** (^{1}H, ^{13}C, COSY, HSQC-DEPT, HMBC)	9
NMR spectra of compound **23** (^{1}H, ^{13}C, COSY, HSQC-DEPT, HMBC)	14
NMR spectra of compound **24** (^{1}H, ^{13}C, COSY, HSQC-DEPT, HMBC)	19
NMR spectra of compound **25** (^{1}H, ^{13}C, COSY, HSQC-DEPT, HMBC)	24
NMR spectra of compound **26** (^{1}H, ^{13}C, COSY, HSQC-DEPT, HMBC)	29
NMR spectra of compound **27** (^{1}H, ^{13}C, COSY, HSQC-DEPT, HMBC)	34
NMR spectra of compound **28** (^{1}H, ^{13}C, COSY, HSQC-DEPT, HMBC)	39
NMR spectra of compound **29** (^{1}H, ^{13}C, COSY, HSQC-DEPT, HMBC)	44
NMR spectra of compound **30** (^{1}H, ^{13}C, COSY, HSQC-DEPT, HMBC)	49
NMR spectra of compound **31** (^{1}H, ^{13}C, COSY, HSQC-DEPT, HMBC)	54
NMR spectra of compound **34** (^{1}H, ^{13}C, COSY, HSQC-DEPT, HMBC)	59
NMR spectra of compound **35** (^{1}H, ^{13}C, COSY, HSQC-DEPT, HMBC)	64
NMR spectra of compound **41** (^{1}H, ^{13}C, COSY, HSQC-DEPT, HMBC)	69
Dose-dependent reduction in RNase H activity Compound **22**	74
Boltzmann Population Analysis and EC_{50} Modeling - Conformers of minimum energy Fig. S1: Umin conformer of **30**	75
Fig. S2: Umin conformer of **31**	76
Fig. S3: Umin conformer of **32**	77
Conformational search – Boltzmann population Tables - Table S1: Conformational search of compound **30**	78
Table S2: Conformational search of compound **31**	84
Table S3: Conformational search of compound **32**	90

Table S4: Boltzmann population analysis summary and sensitivity analysis 97
Table S5: RMS statistics 99

Copies of NMR spectra

^{1}H NMR of **21** (600.11 MHz, DMSO-d_6)

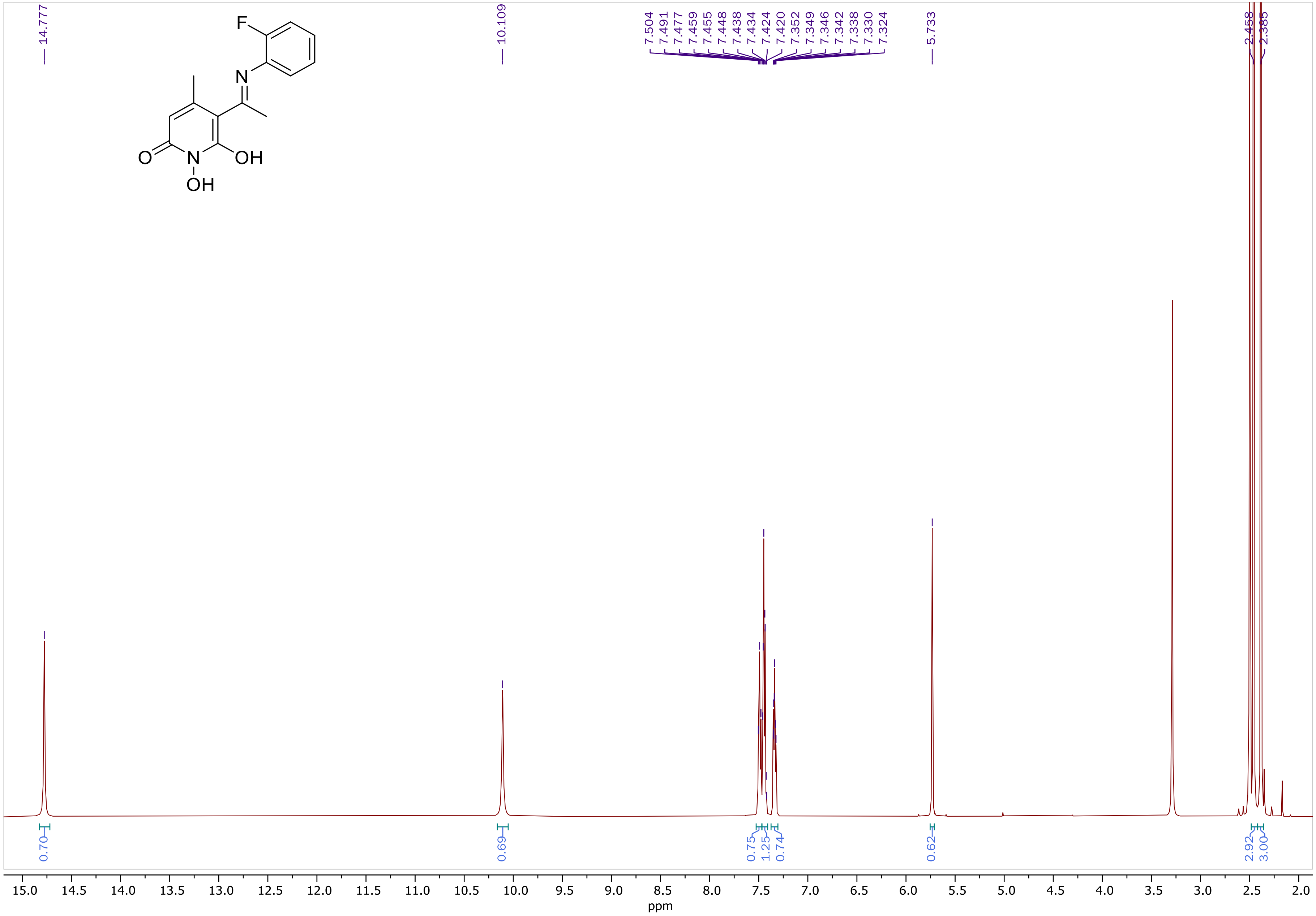

^{13}C NMR of **21** (150.9 MHz, DMSO-d_6)

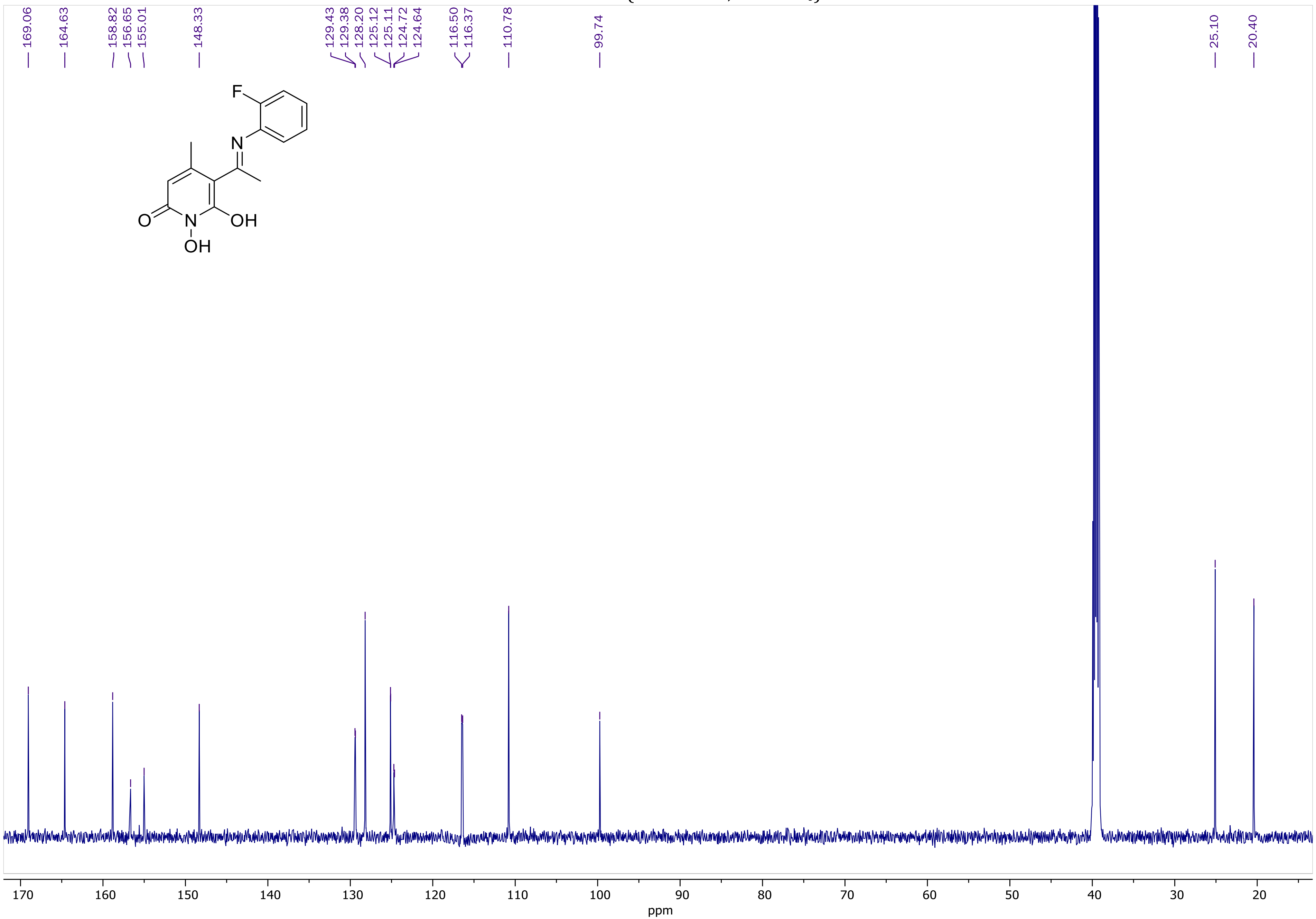

COSY NMR of **21** (600.11 MHz, DMSO-d_6)

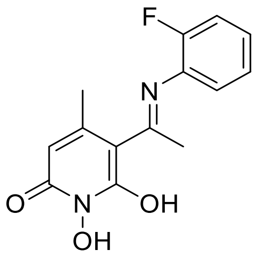

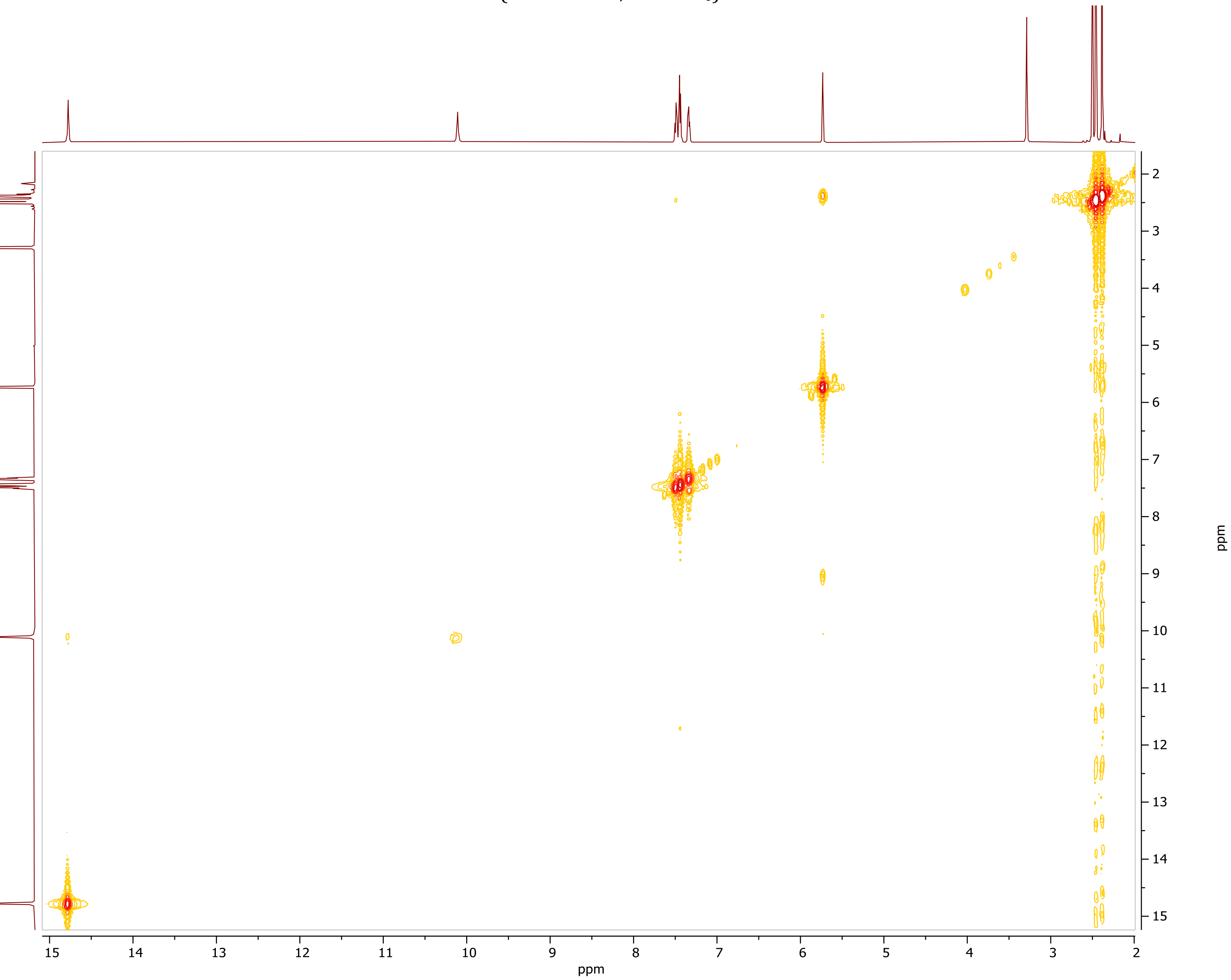

HSQC-DEPT NMR of **21** (600.11 MHz, DMSO-d_6)

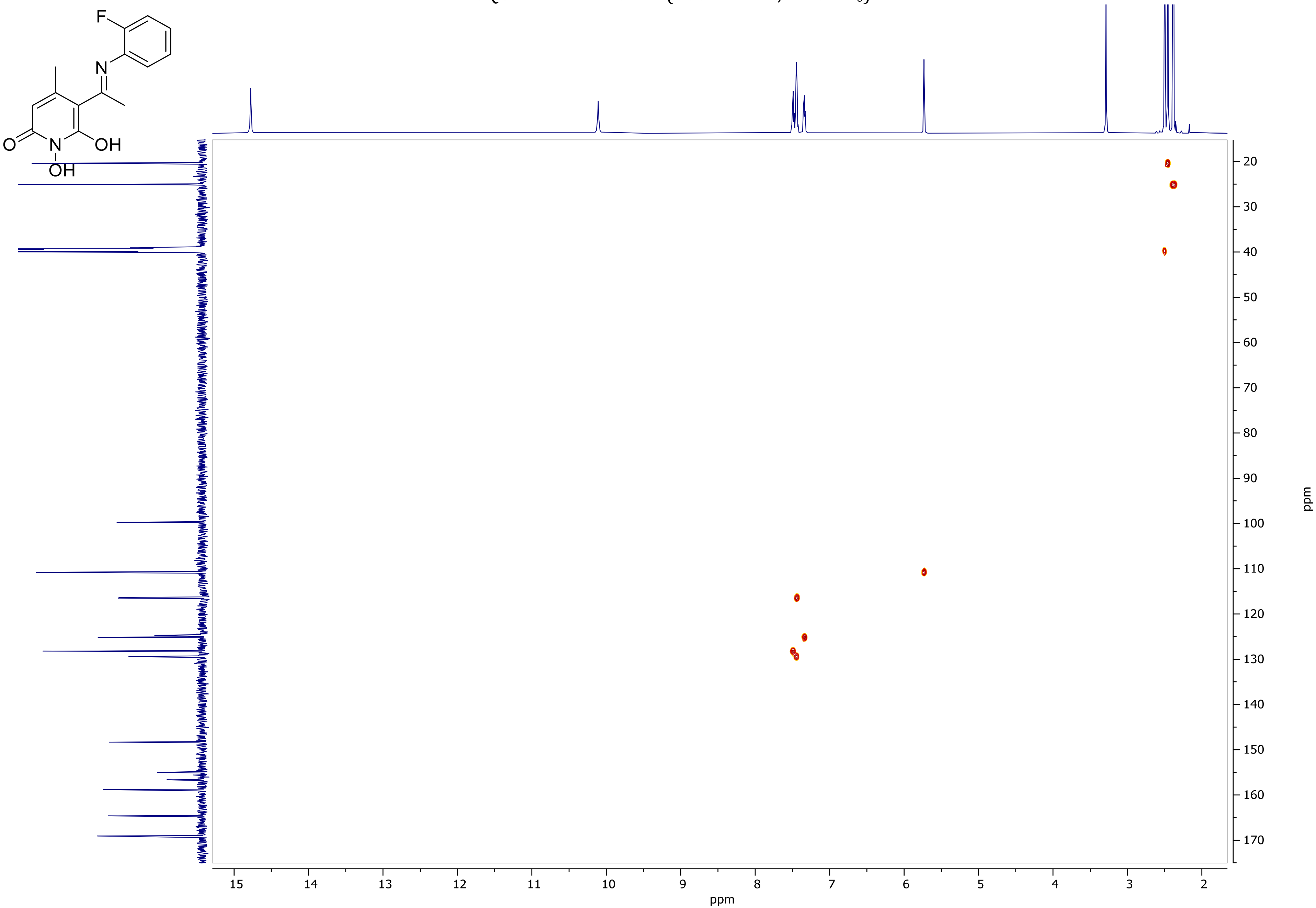

HMBC NMR of **21** (600.11 MHz, DMSO-d_6)

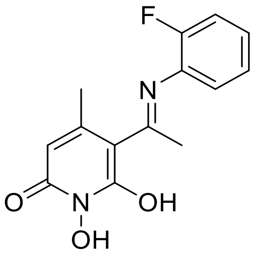

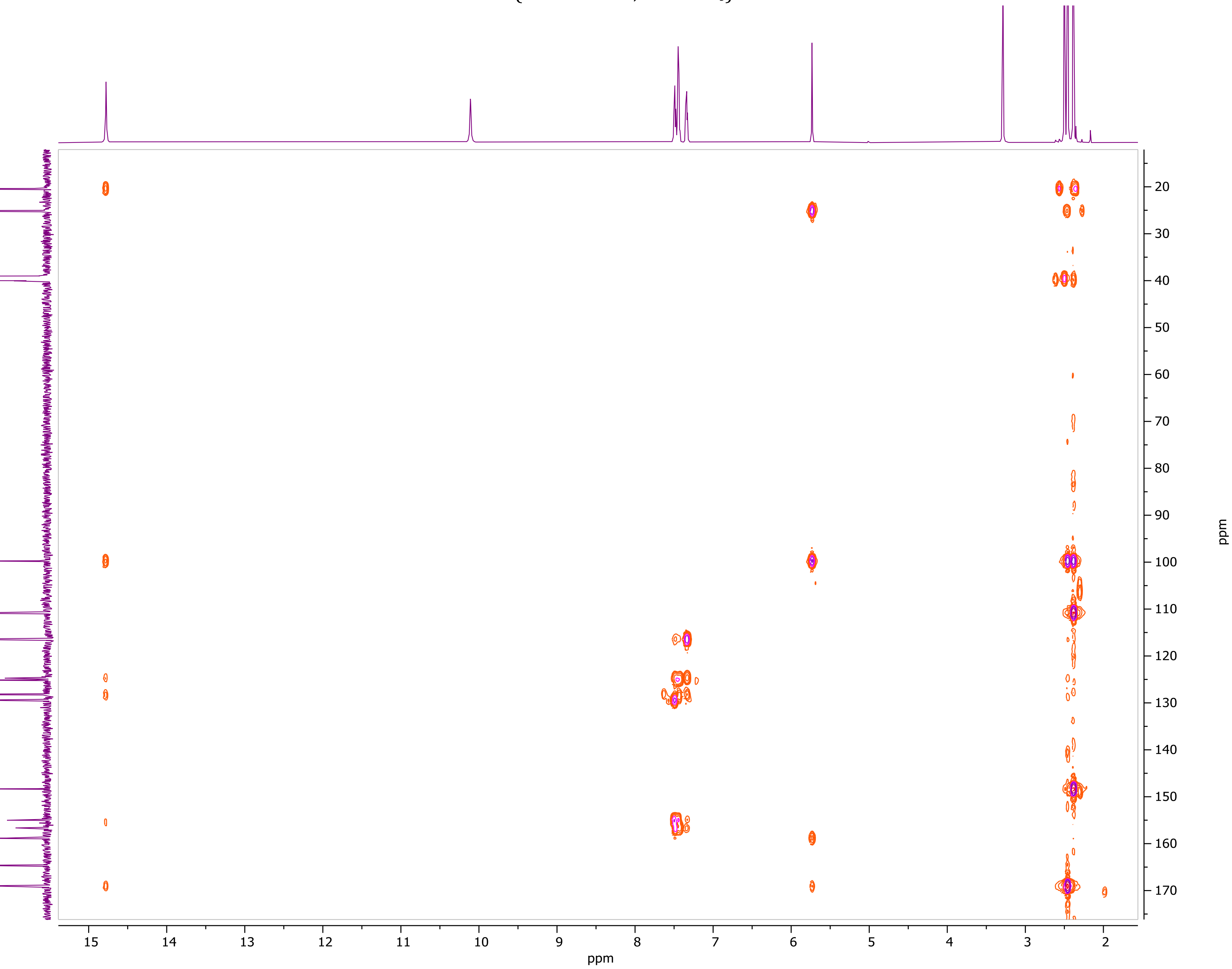

^{1}H NMR of **22** (600.11 MHz, DMSO-d_6)

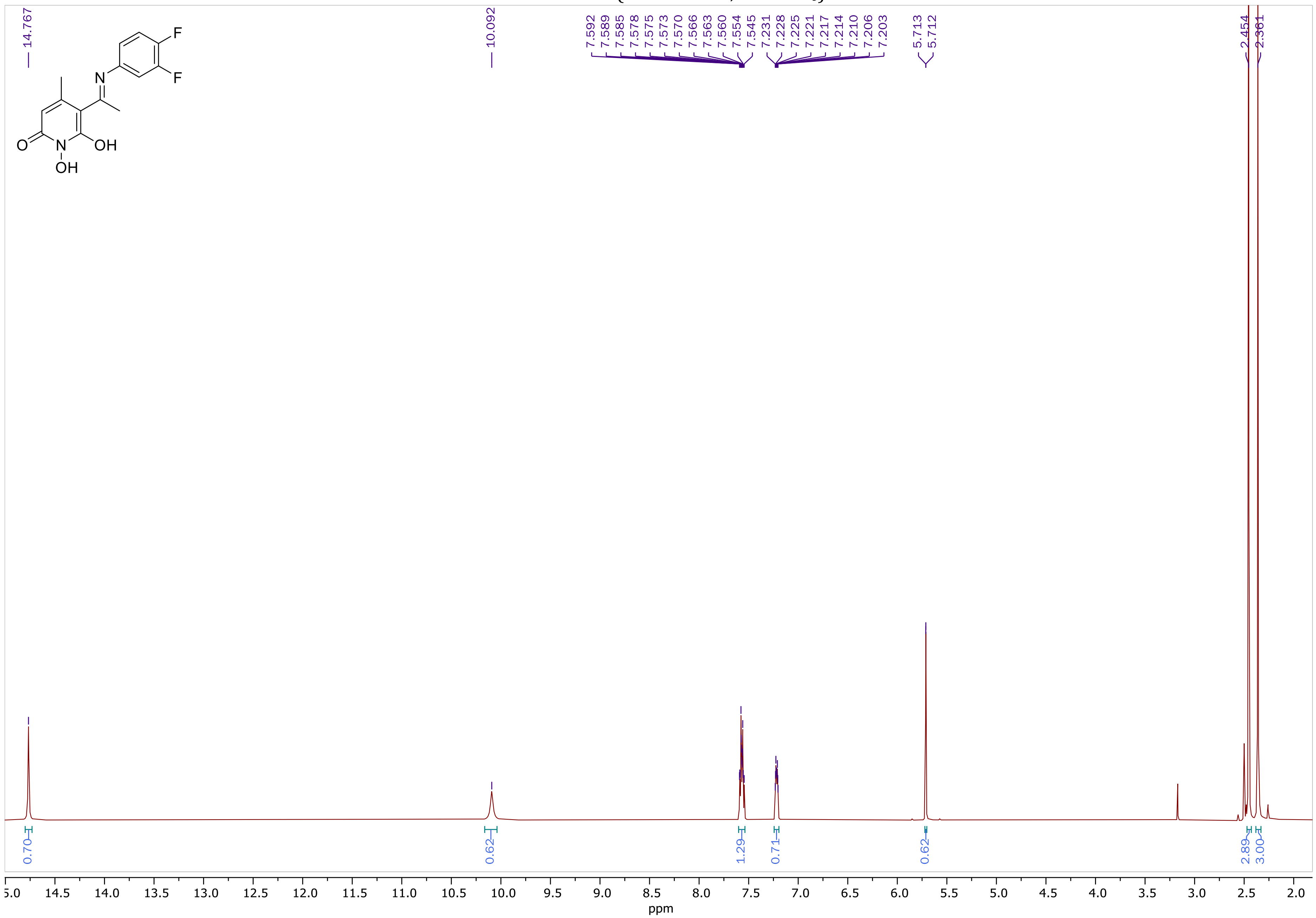

13C NMR of **22** (150.9 MHz, DMSO-d_6)

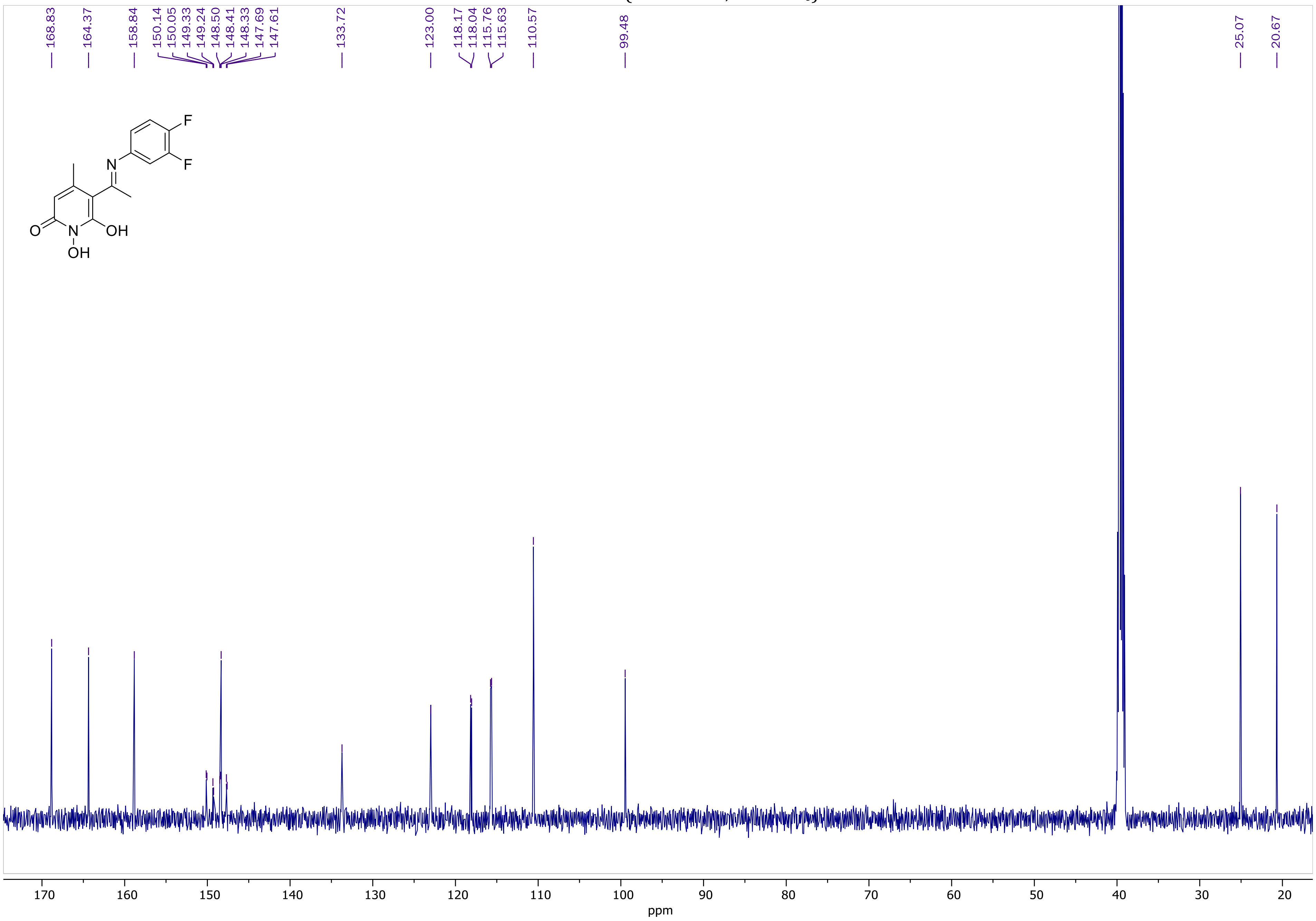

COSY NMR of **22** (600.11 MHz, DMSO-d_6)

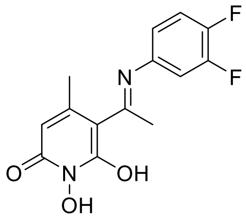

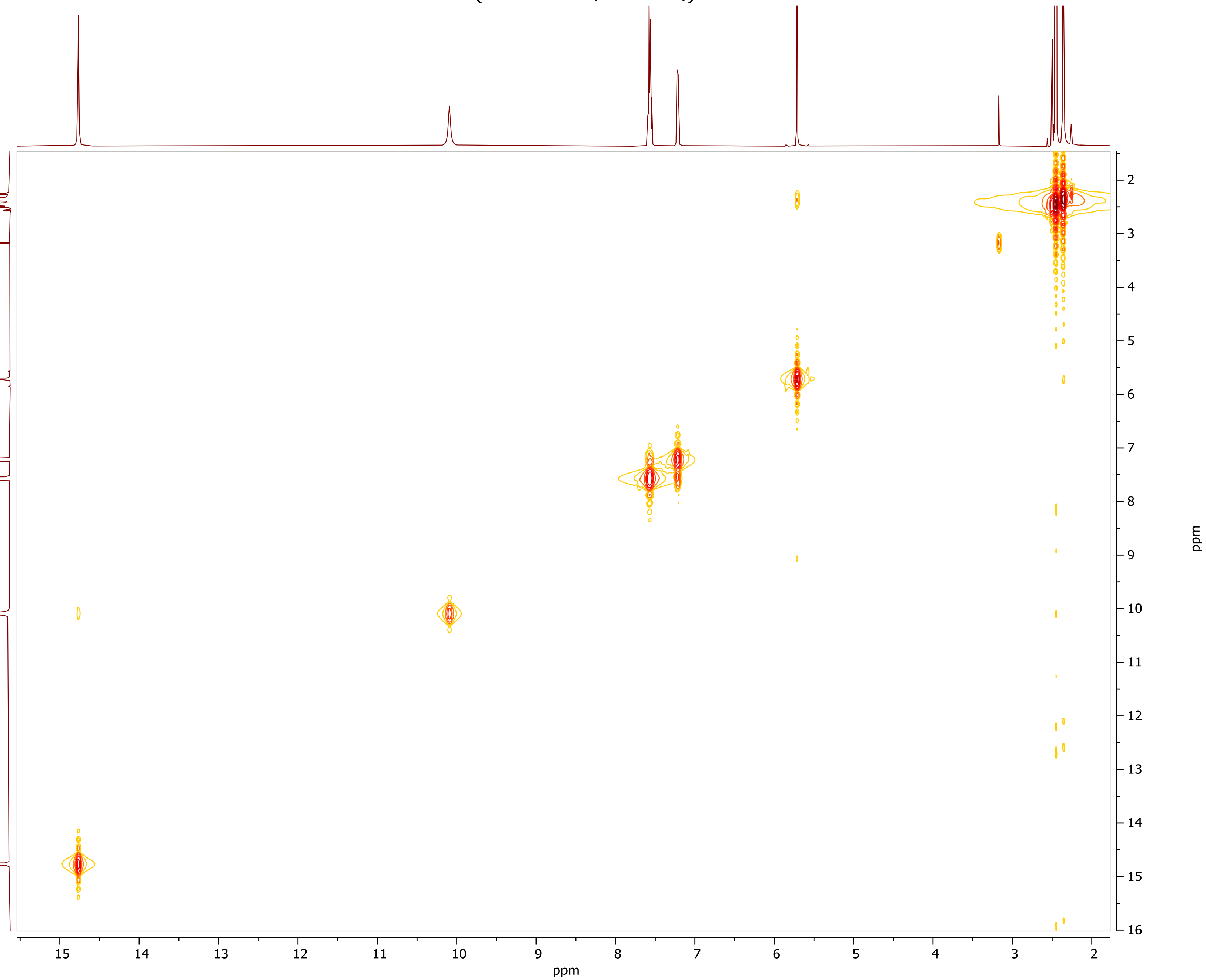

HSQC-DEPT NMR of **22** (600.11 MHz, DMSO-d_6)

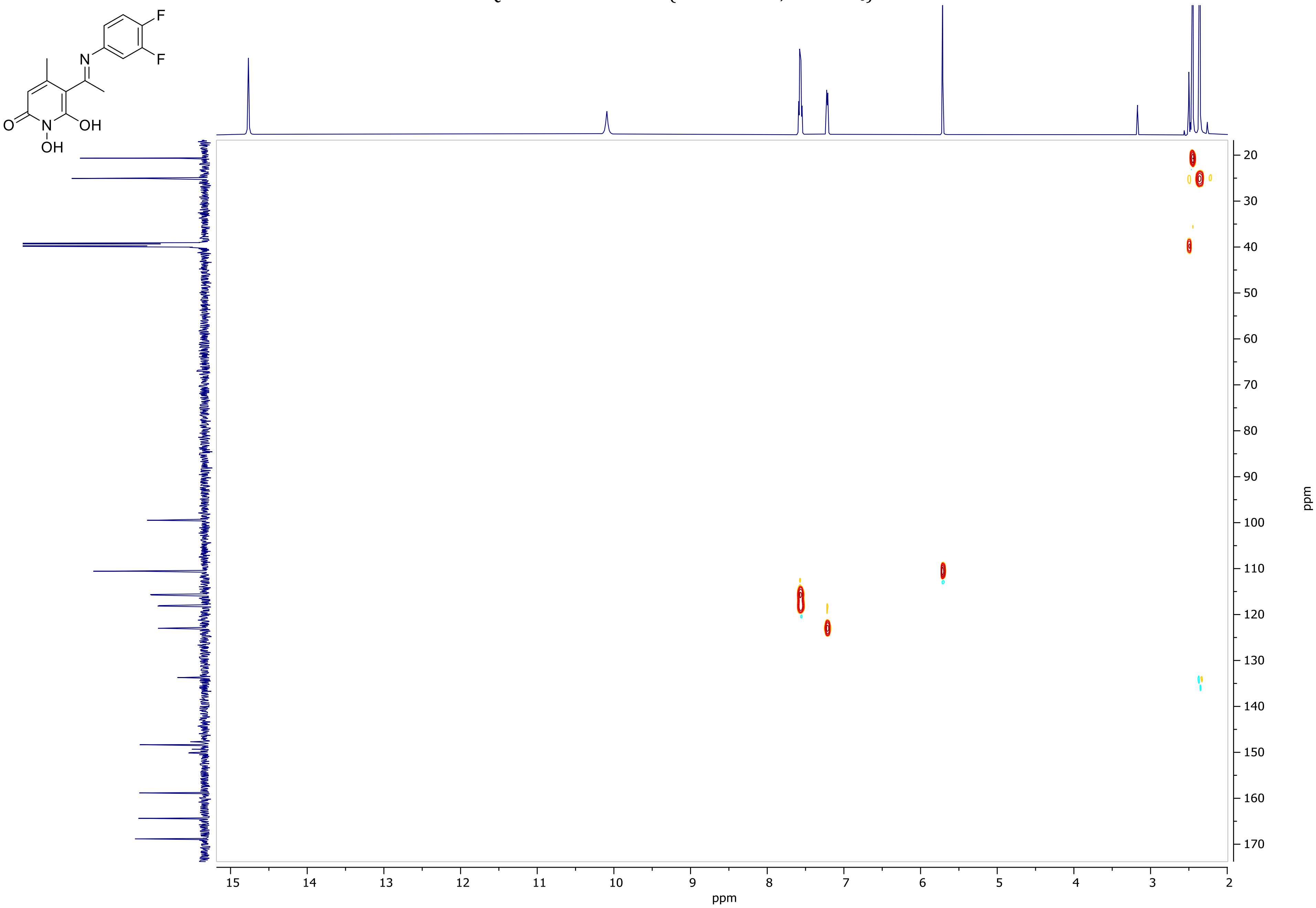

HMBC NMR of **22** (600.11 MHz, DMSO-d_6)

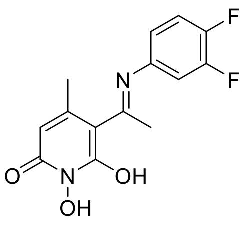

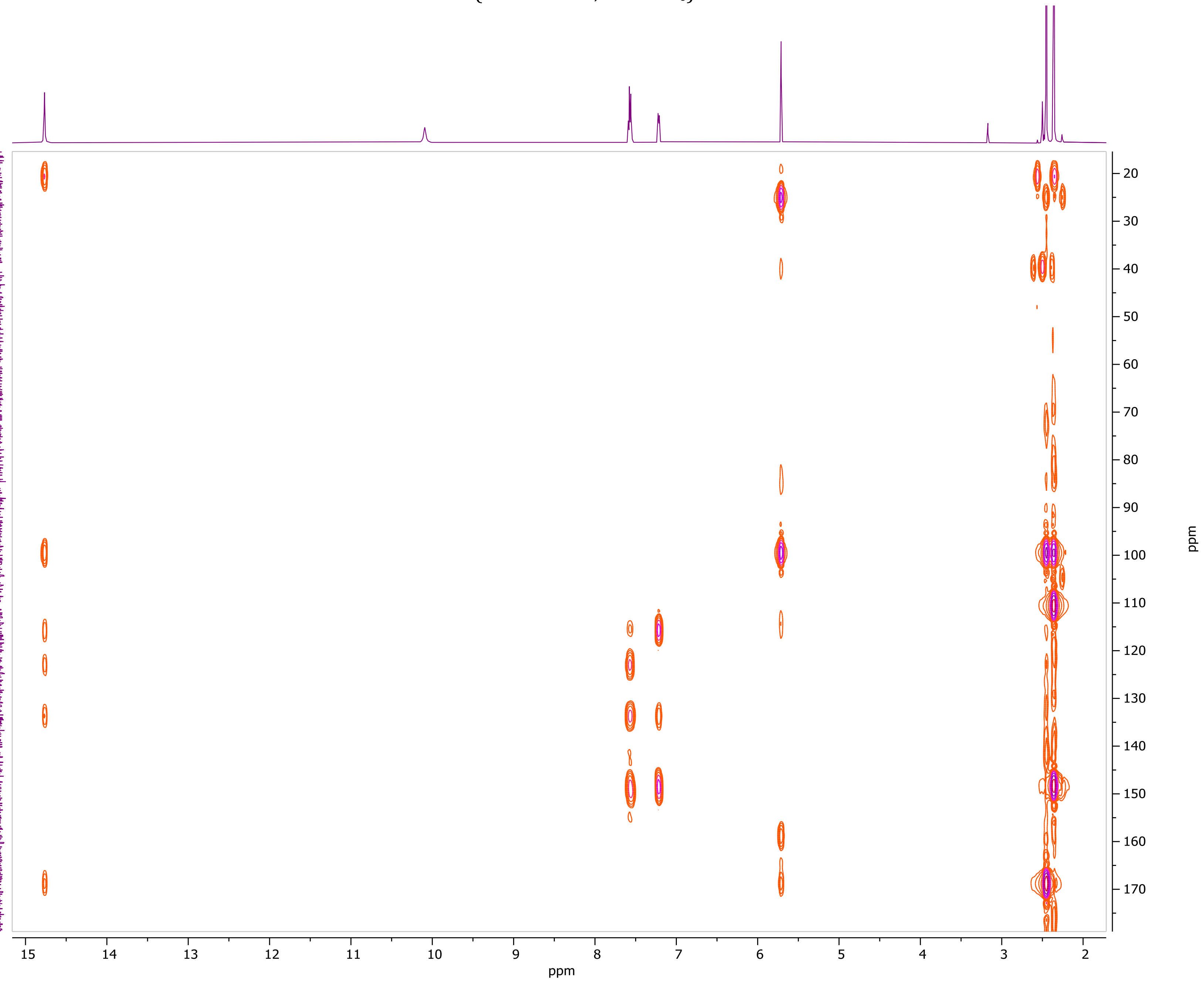

^{1}H NMR of **23** (600.11 MHz, DMSO-d_6)

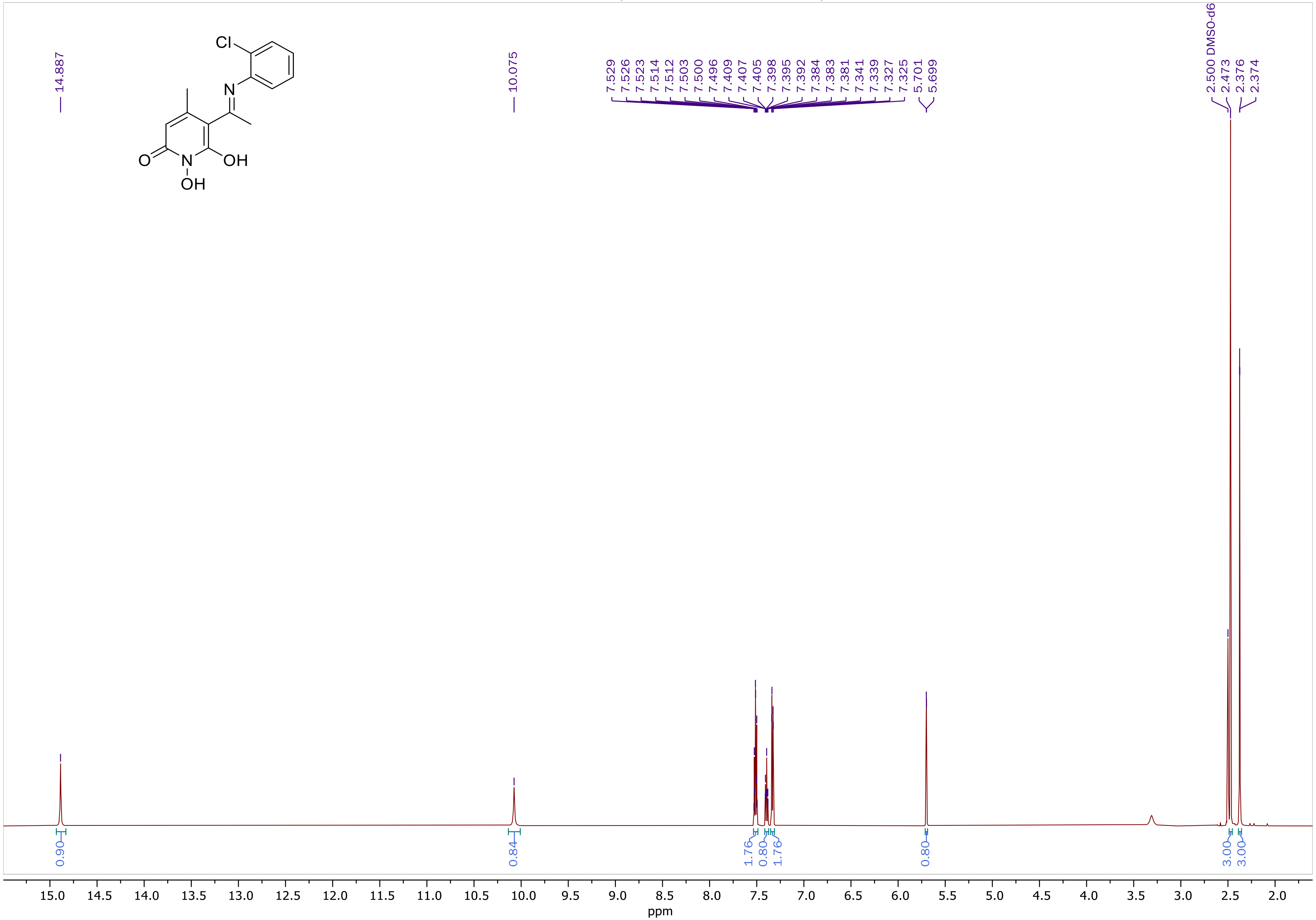

^{13}C NMR of **23** (150.9 MHz, DMSO-d_6)

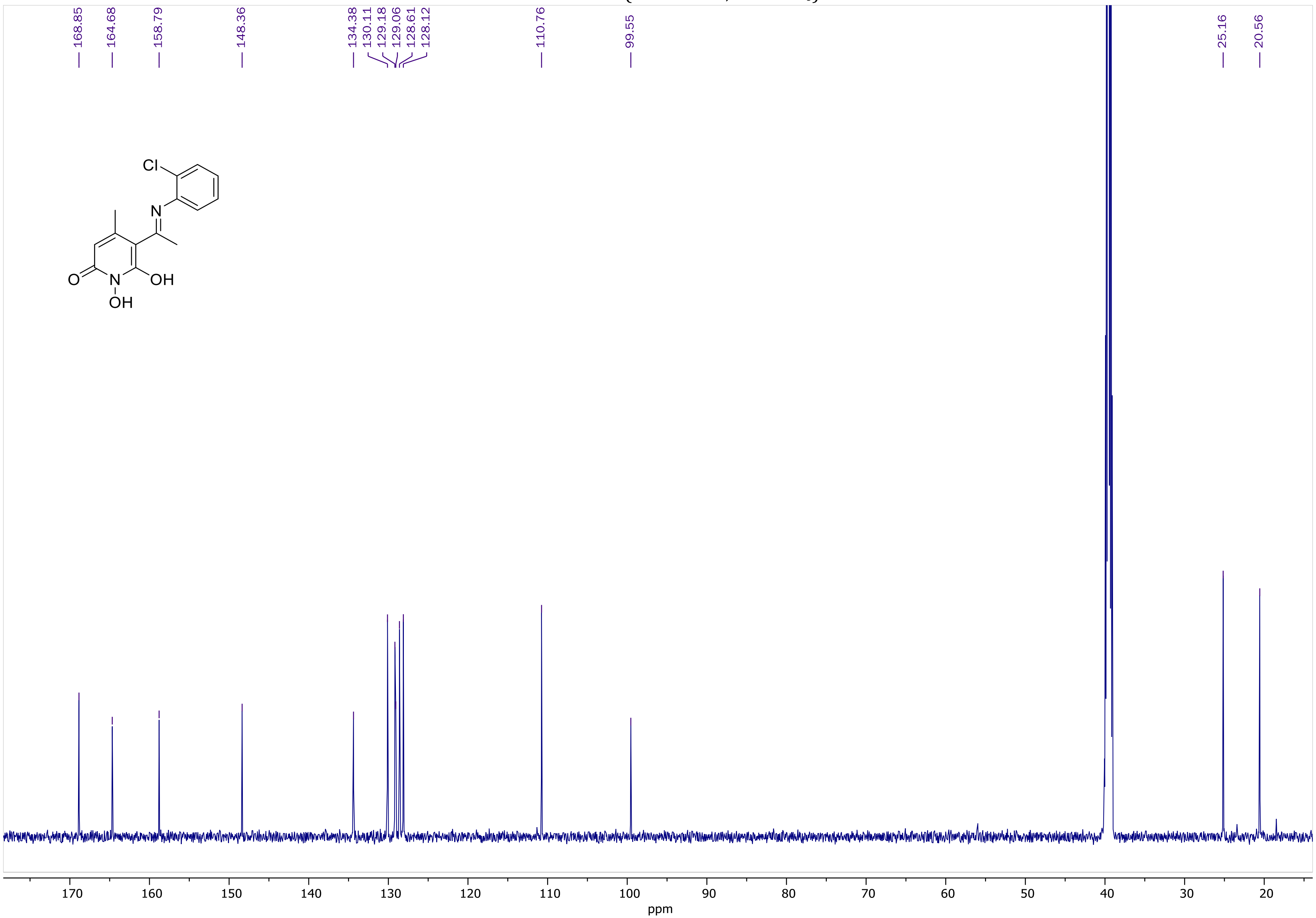

COSY NMR of **23** (600.11 MHz, DMSO-d_6)

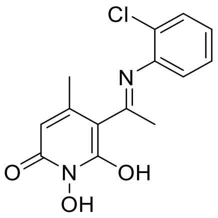

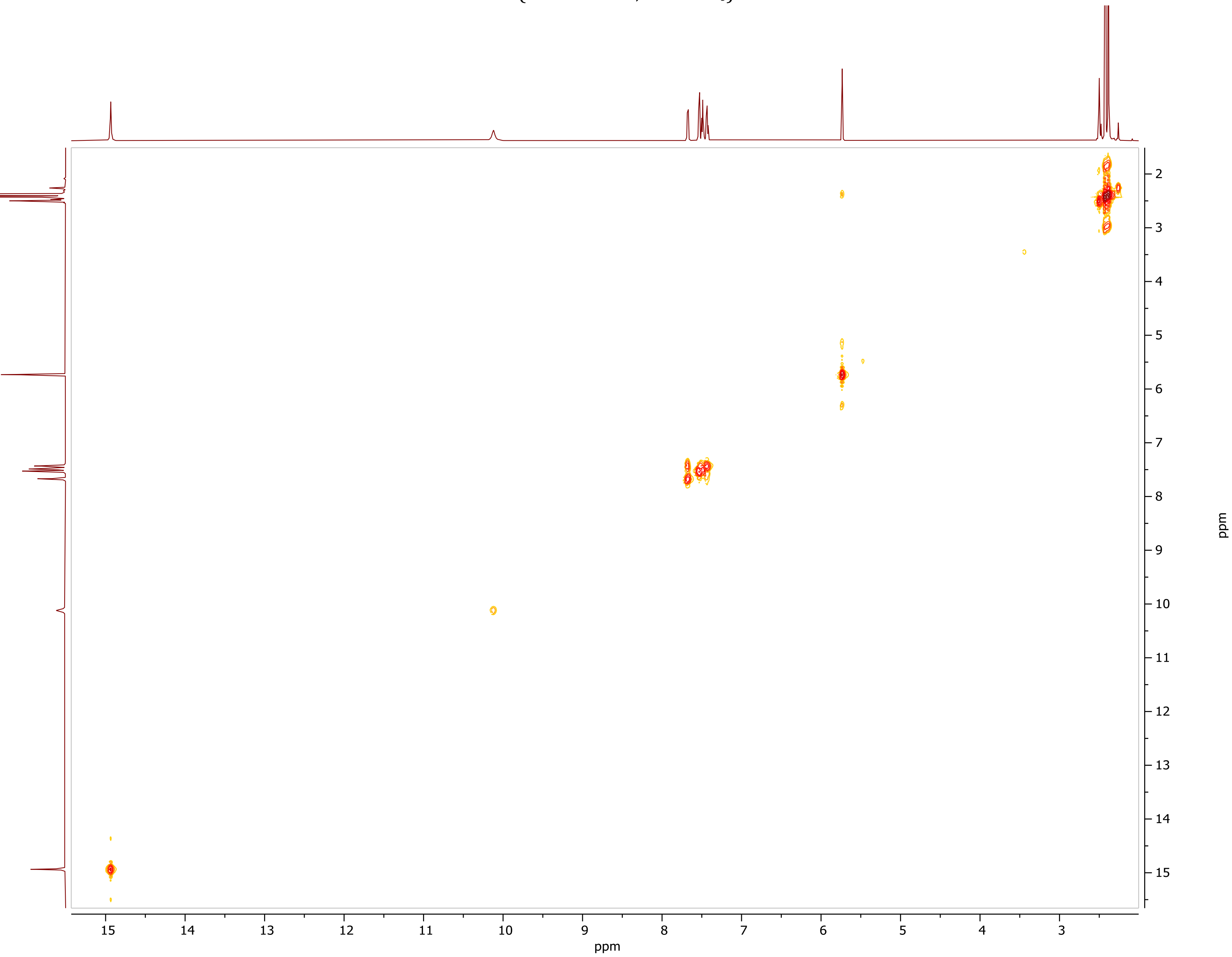

HSQC-DEPT NMR of **23** (600.11 MHz, DMSO-d_6)

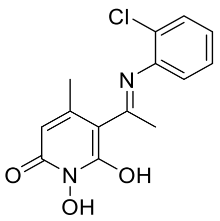

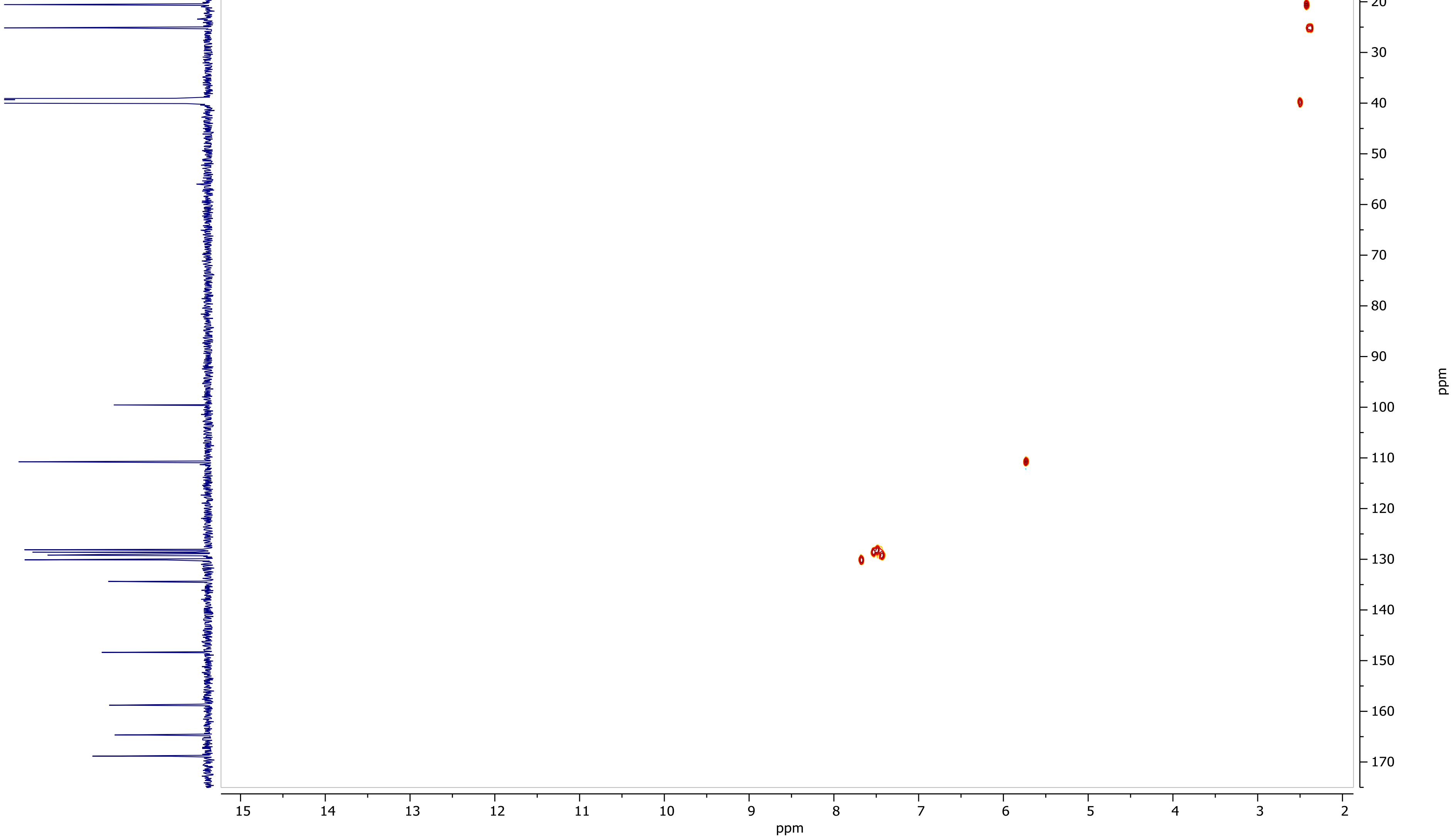

HMBC NMR of **23** (600.11 MHz, DMSO-d_6)

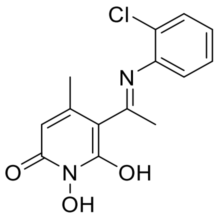

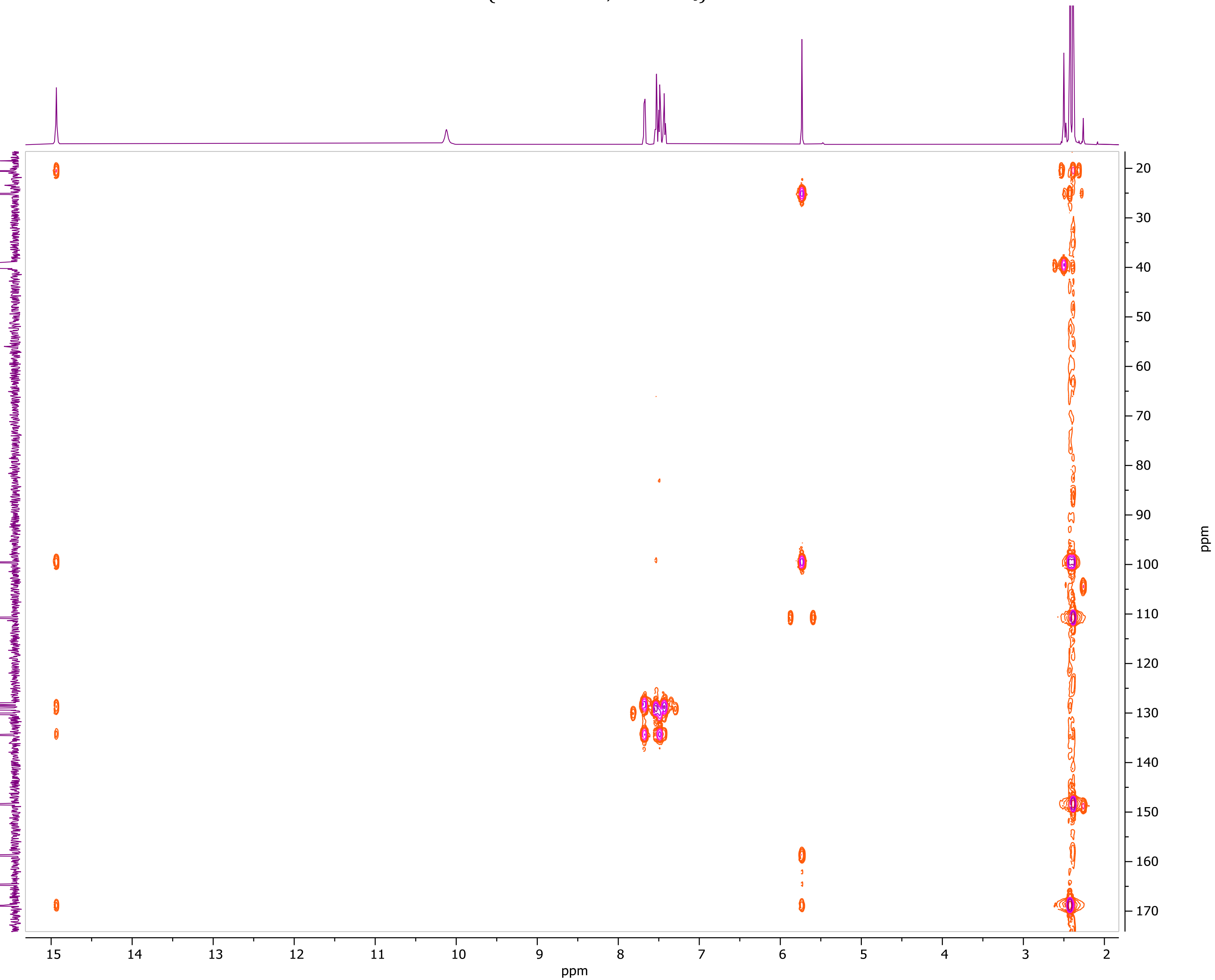

^1H NMR of **24** (600.11 MHz, DMSO-d_6)

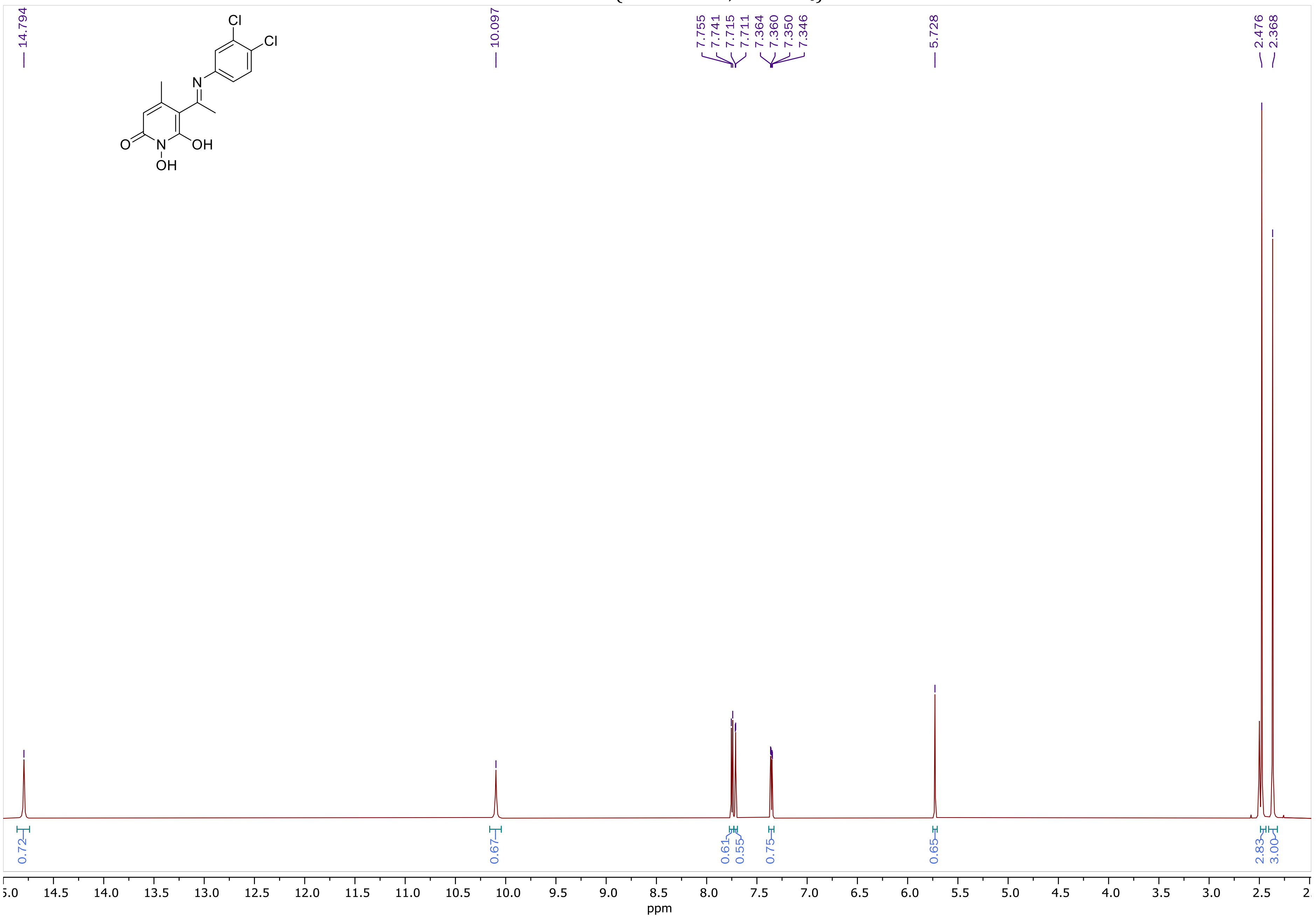

^{13}C NMR of **24** (150.9 MHz, DMSO-d_6)

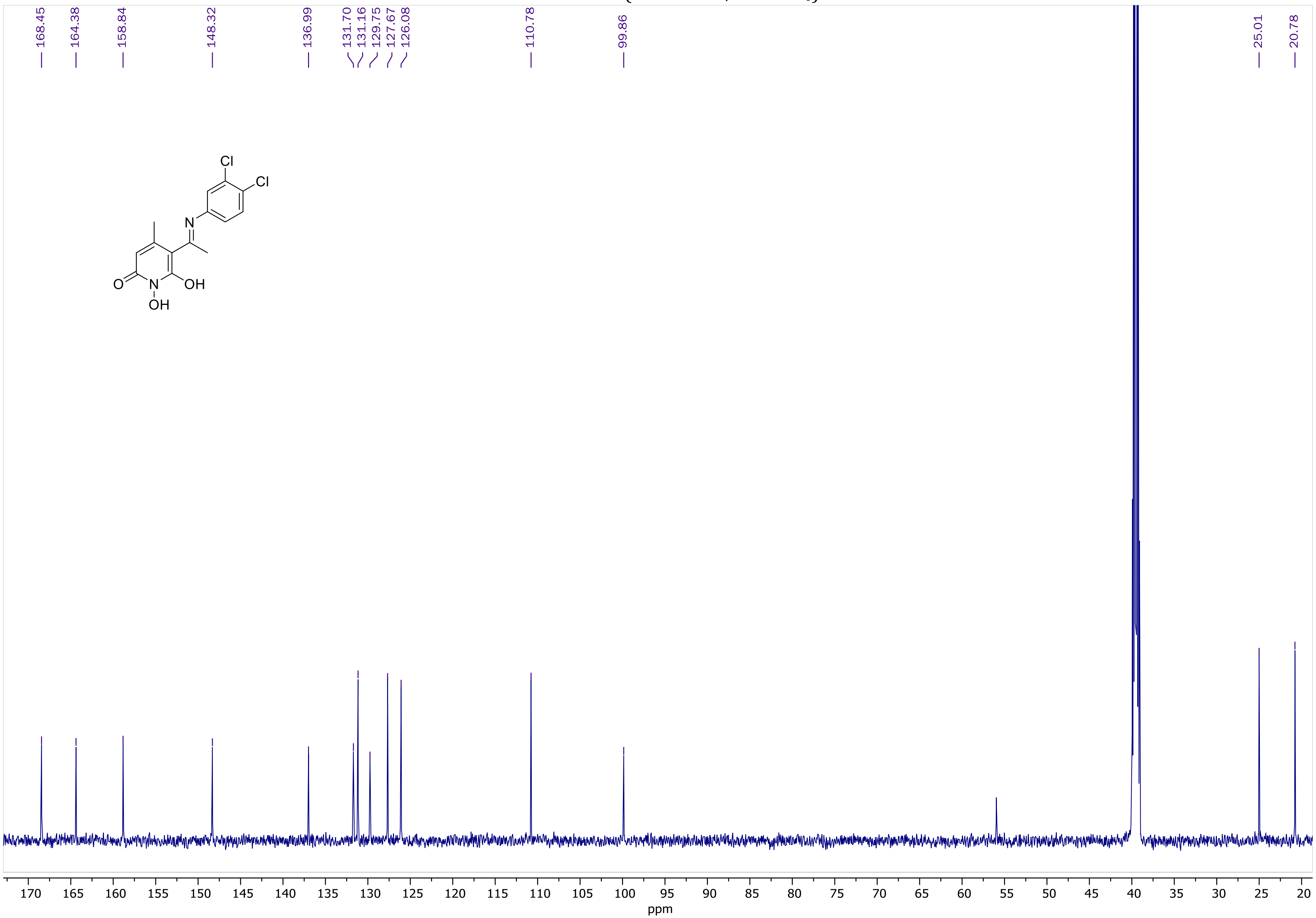

COSY NMR of **24** (600.11 MHz, DMSO-d_6)

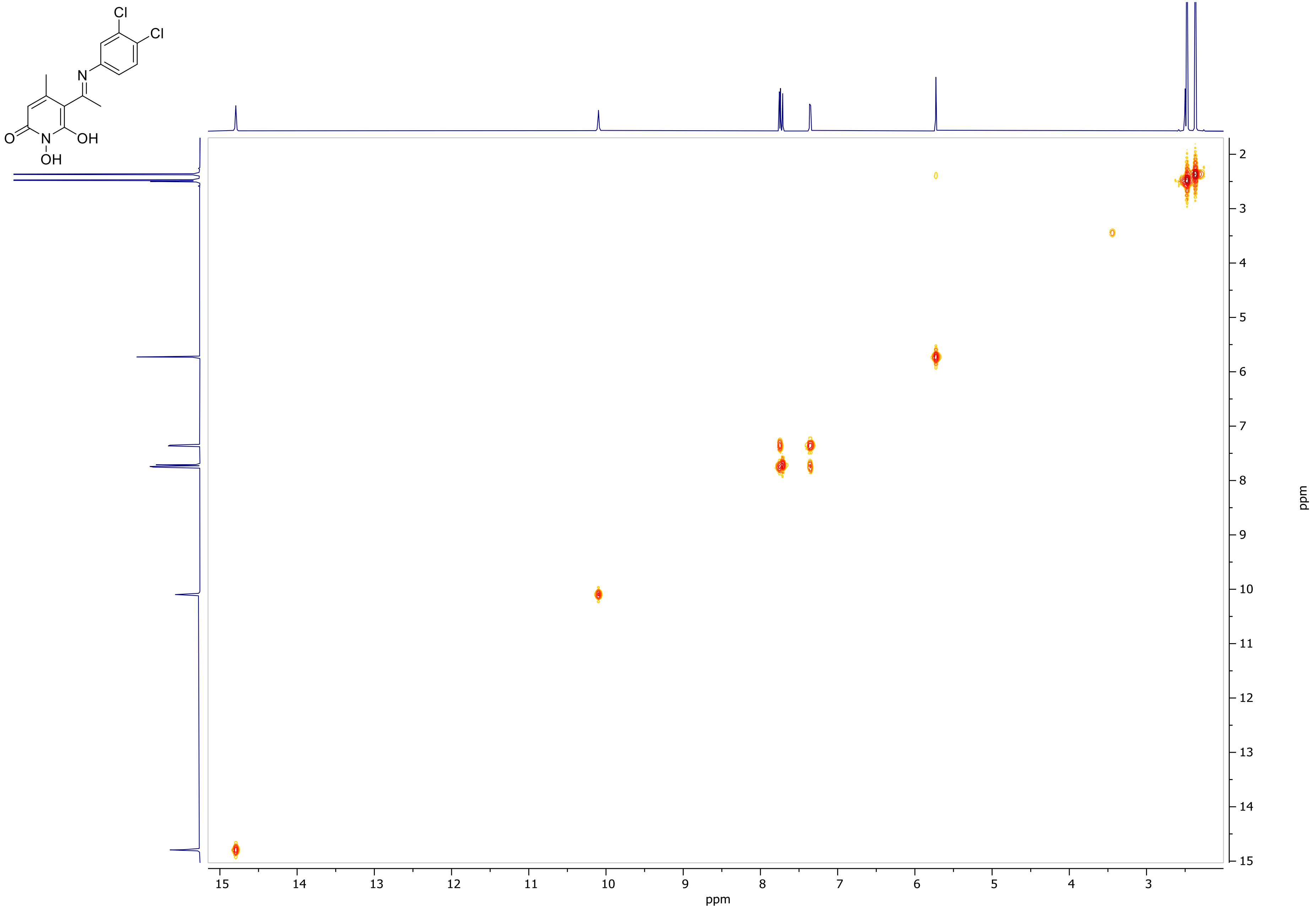

HSQC-DEPT NMR of **24** (600.11 MHz, DMSO-d_6)

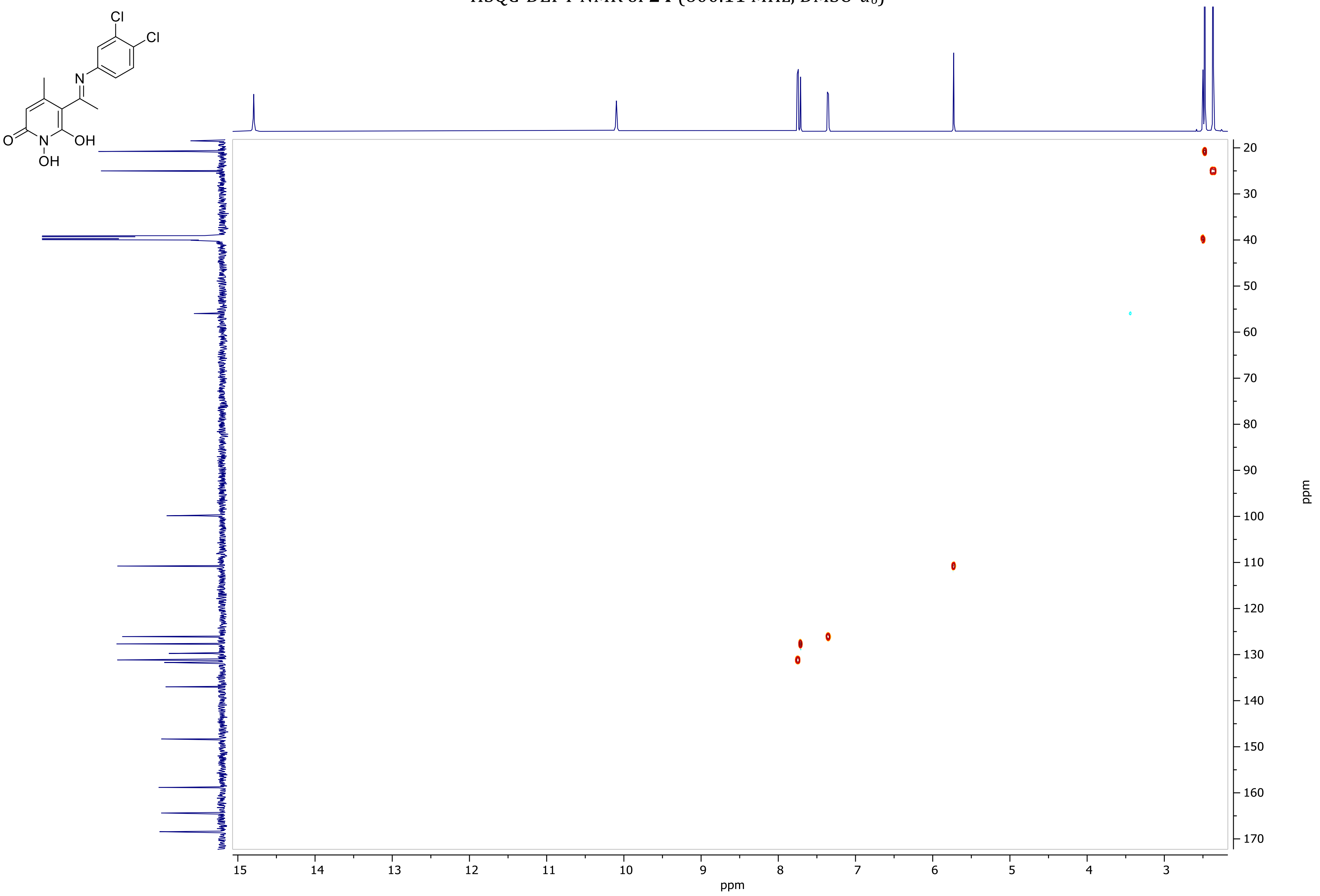

HMBC NMR of **24** (600.11 MHz, DMSO-d_6)

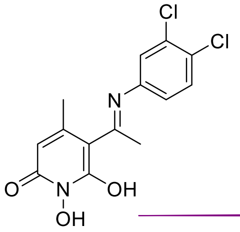

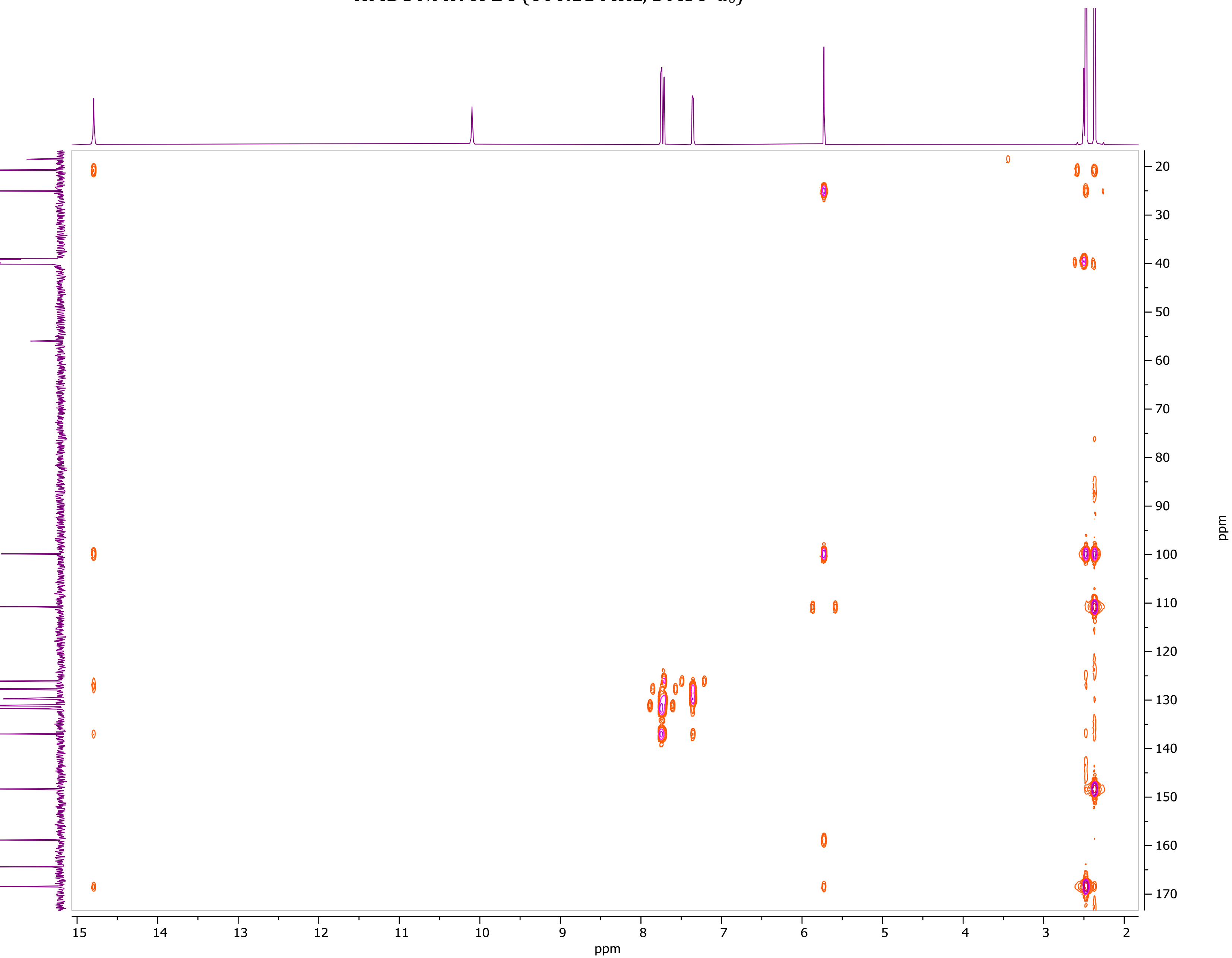

^{1}H NMR of **25** (600.11 MHz, DMSO-d_6)

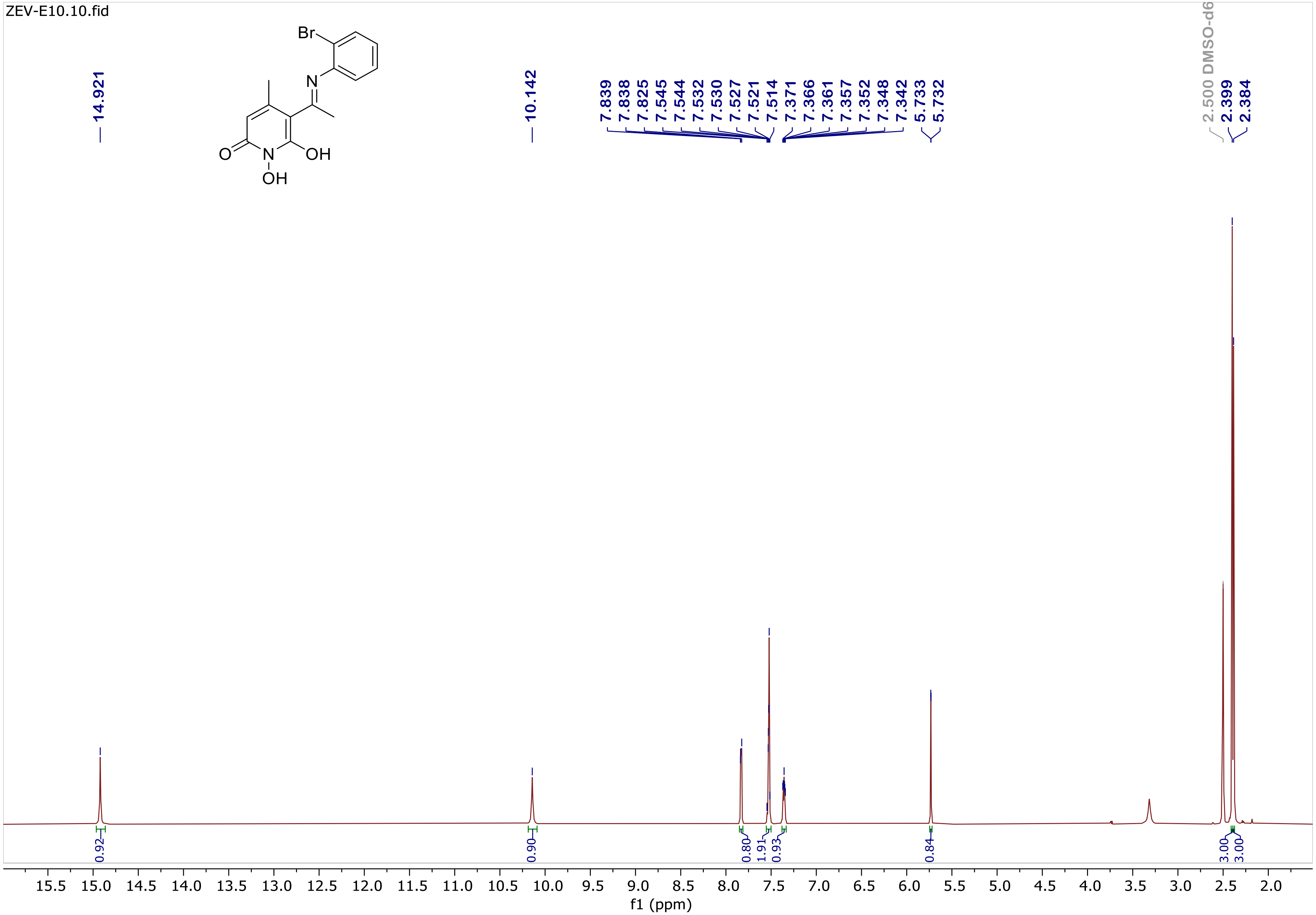

^{13}C NMR of **25** (150.9 MHz, DMSO-d_6)

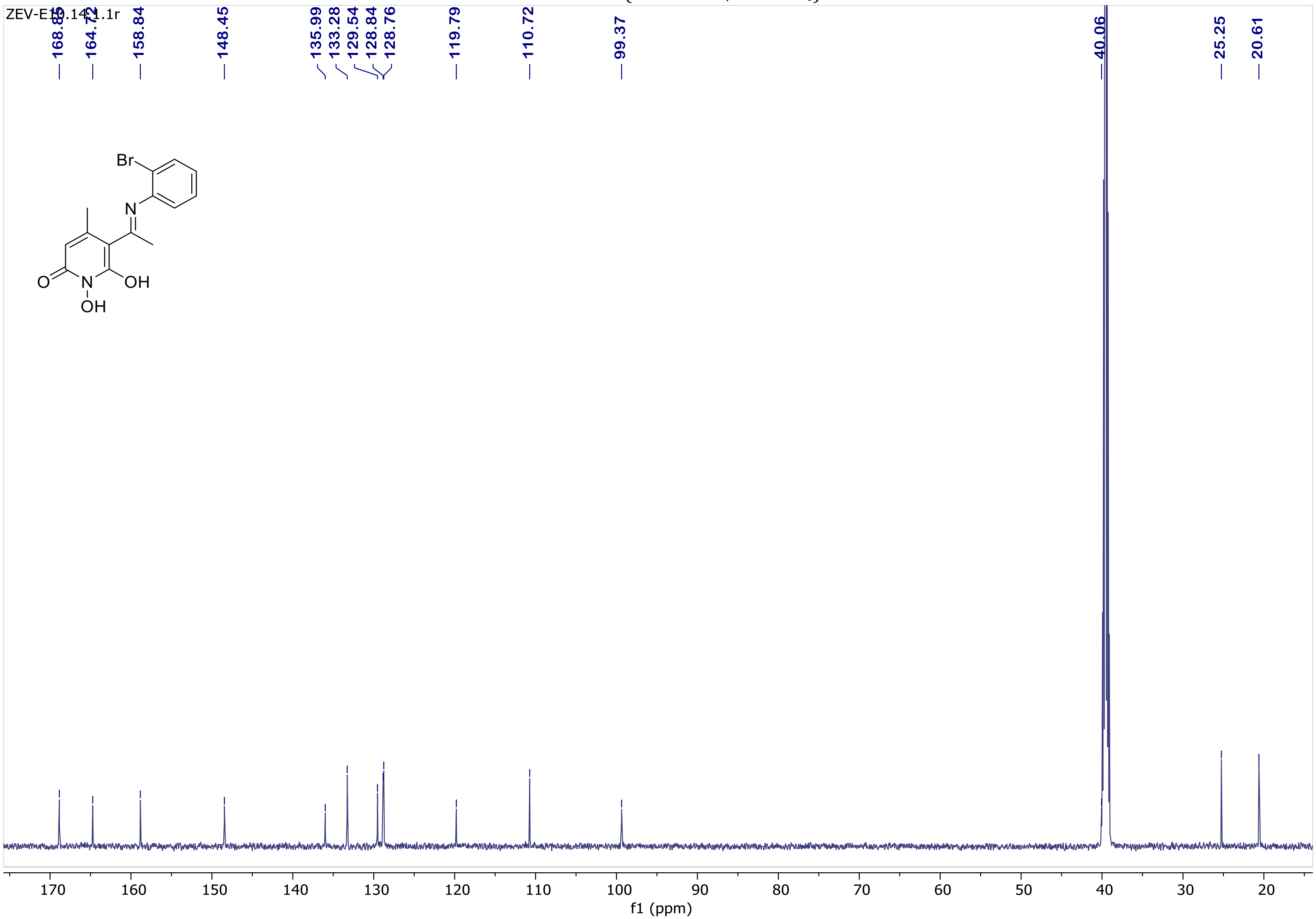

COSY NMR of **25** (600.11 MHz, DMSO-d_6)

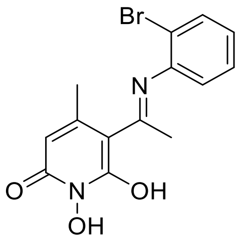

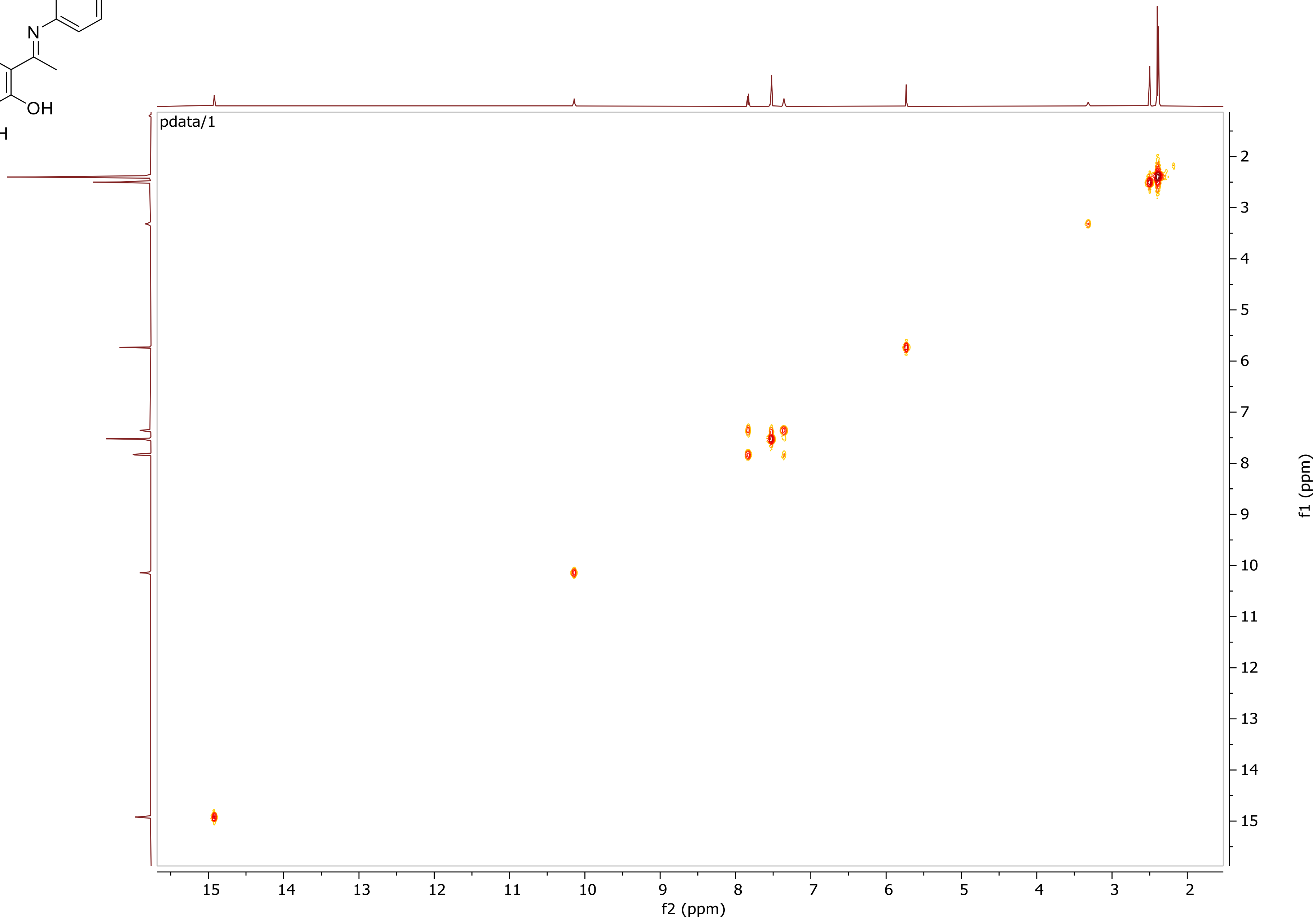

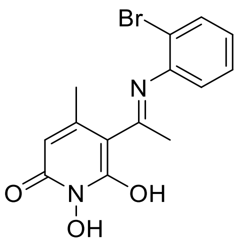

HSQC-DEPT NMR of **25** (600.11 MHz, DMSO-d_6)

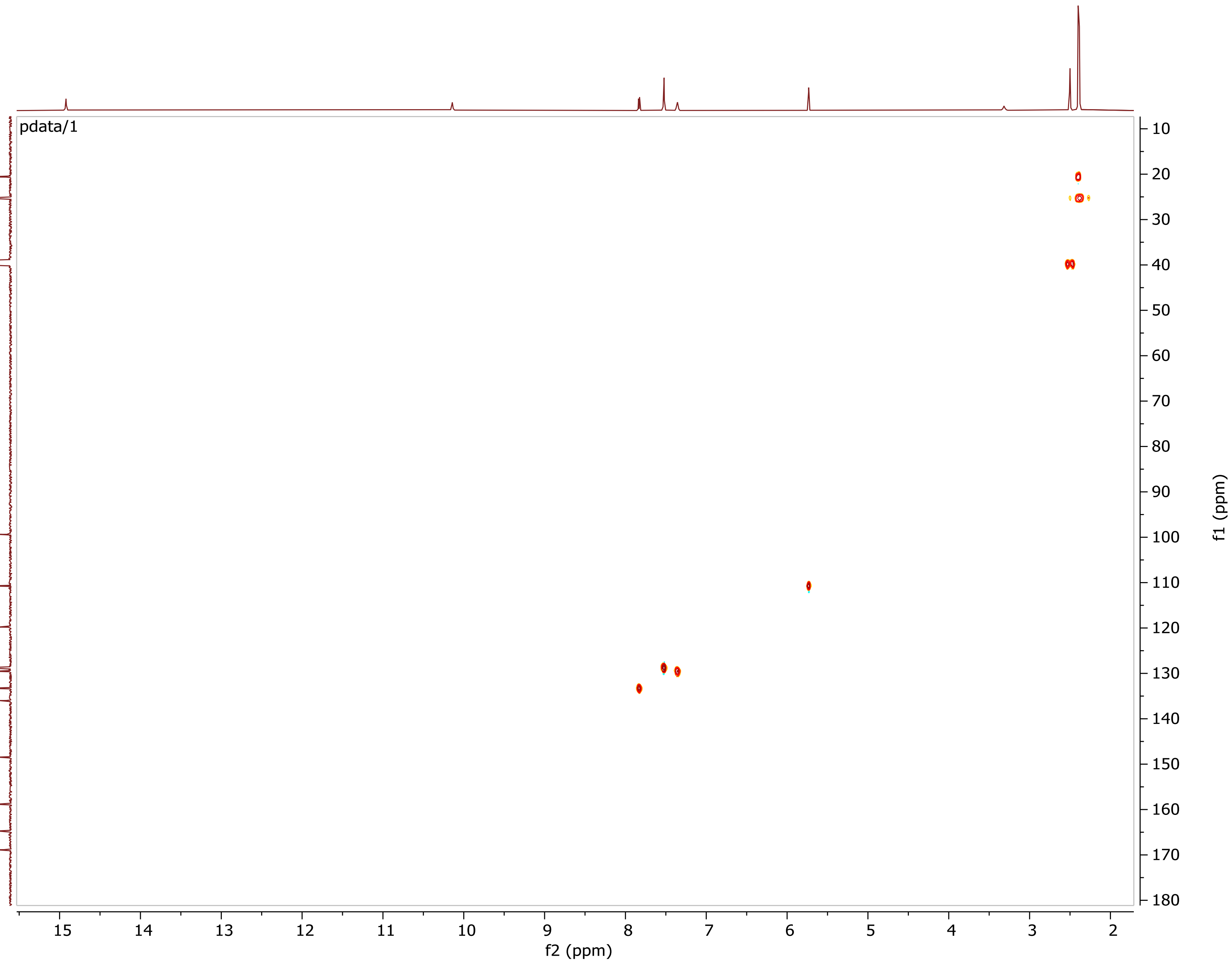

HMBC NMR of **25** (600.11 MHz, DMSO-d_6)

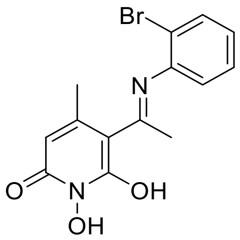

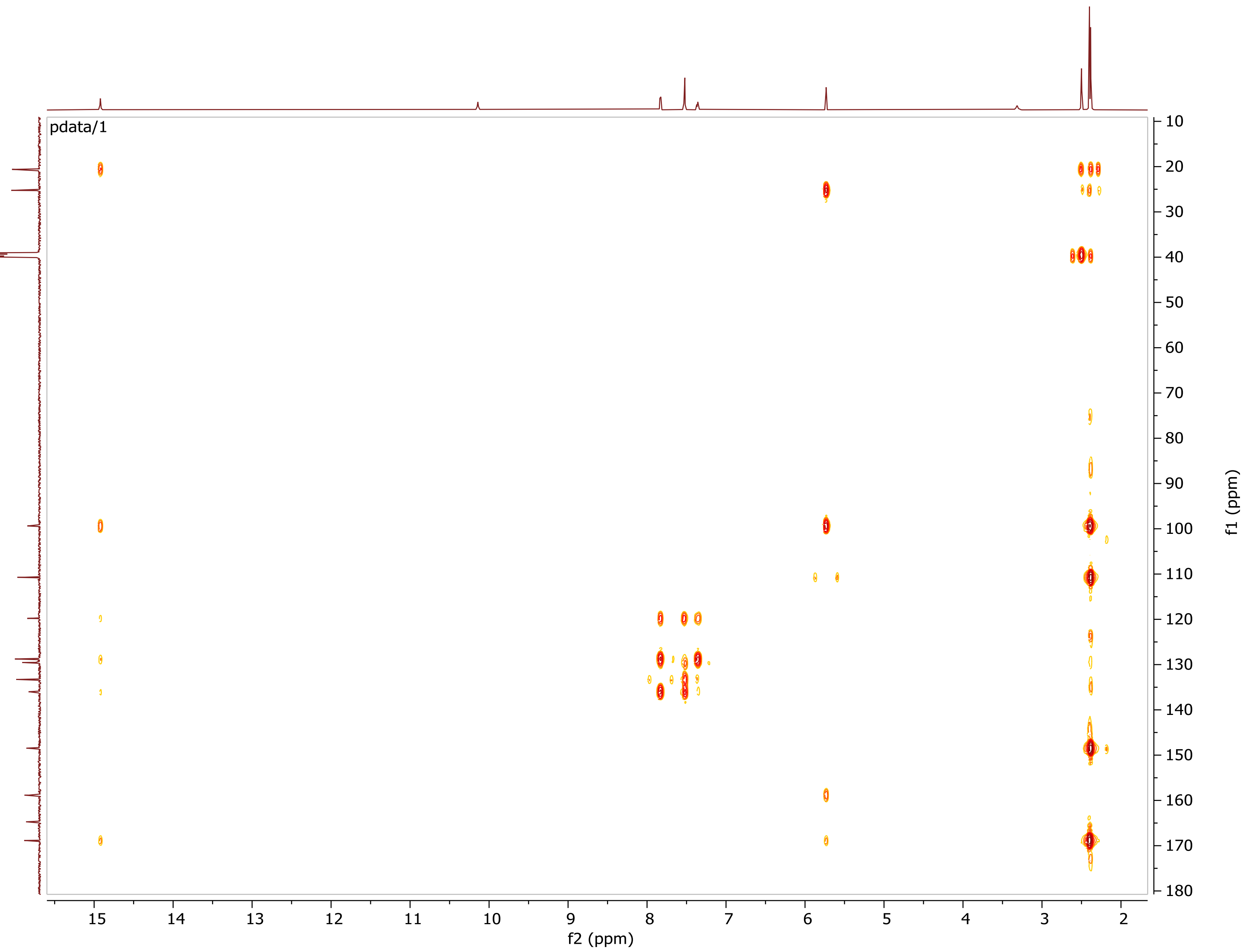

^{1}H NMR of **26** (600.11 MHz, DMSO-d_6)

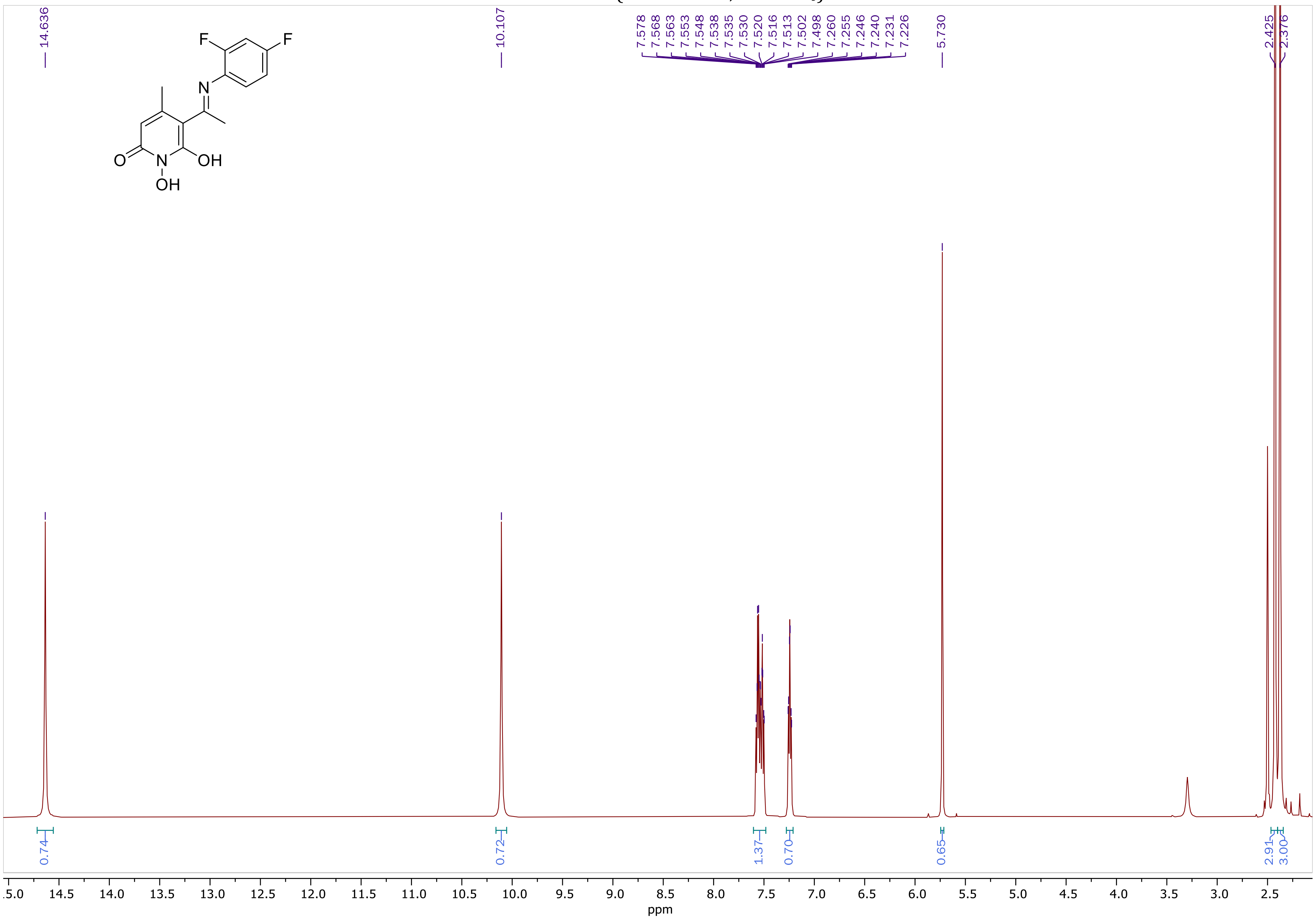

^{13}C NMR of **26** (150.9 MHz, DMSO-d_6)

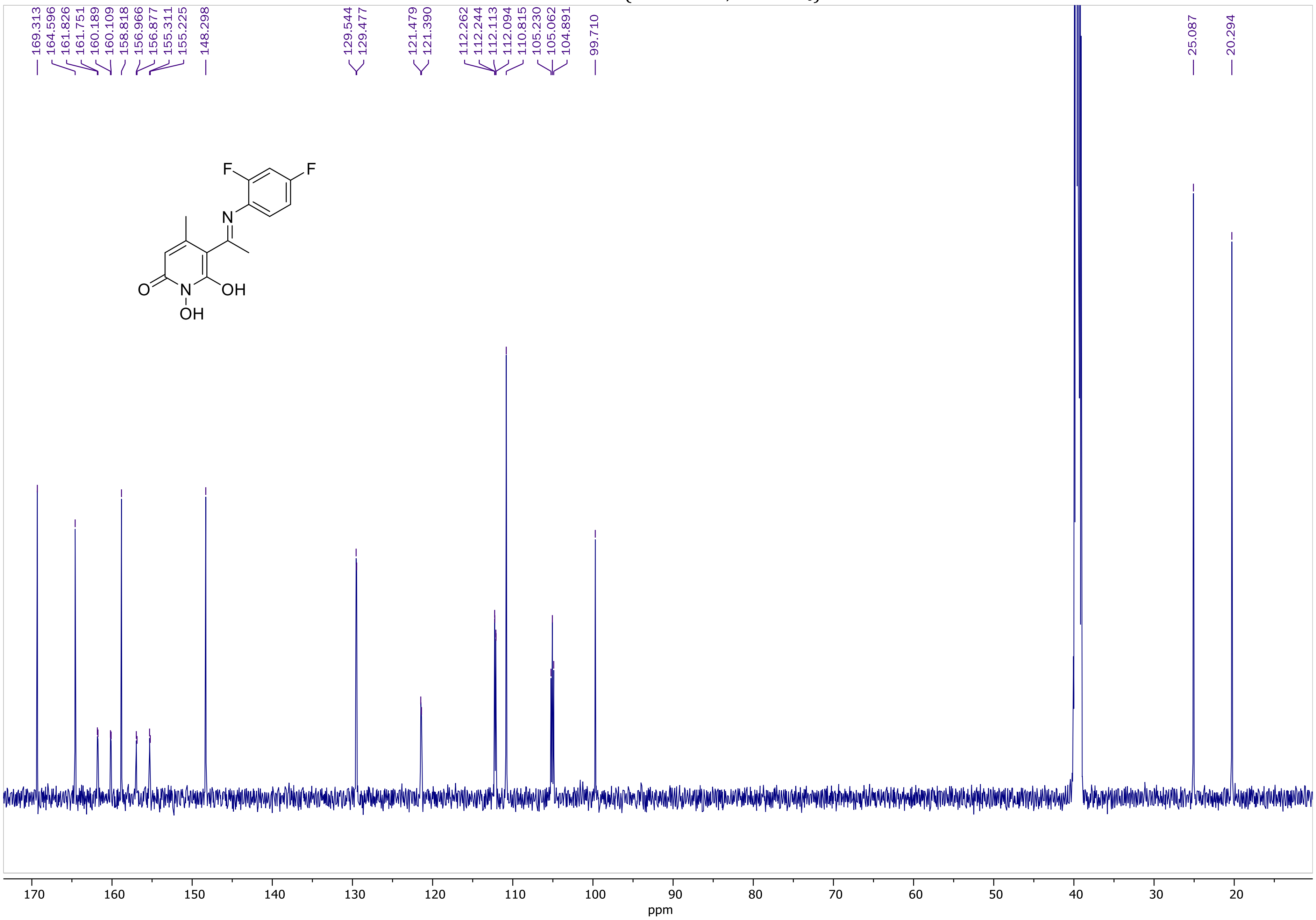

COSY NMR of **26** (600.11 MHz, DMSO-d_6)

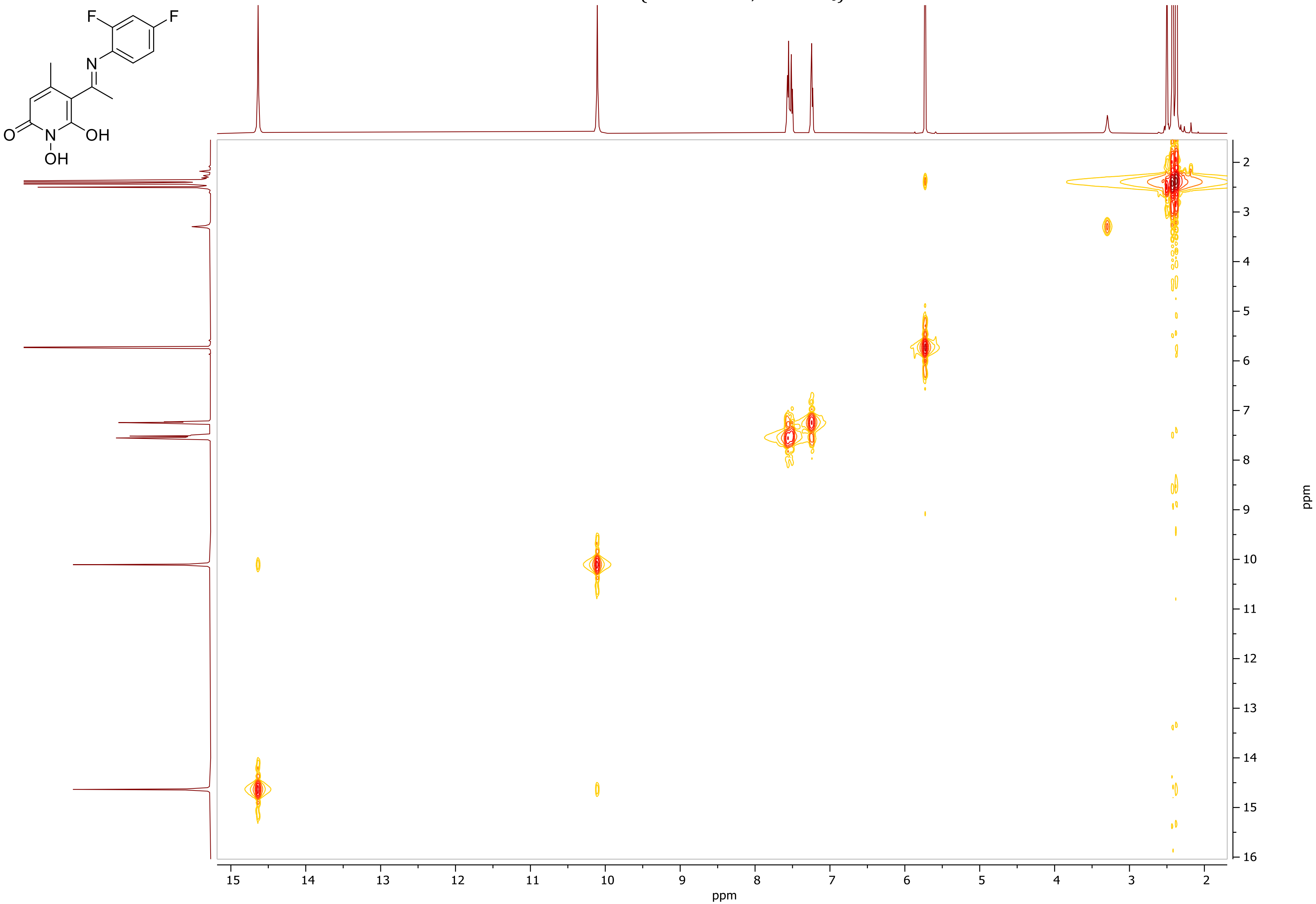

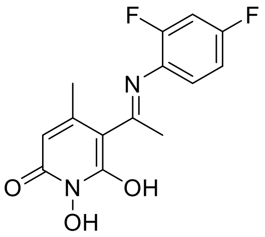

HSQC-DEPT NMR of **26** (600.11 MHz, DMSO-d_6)

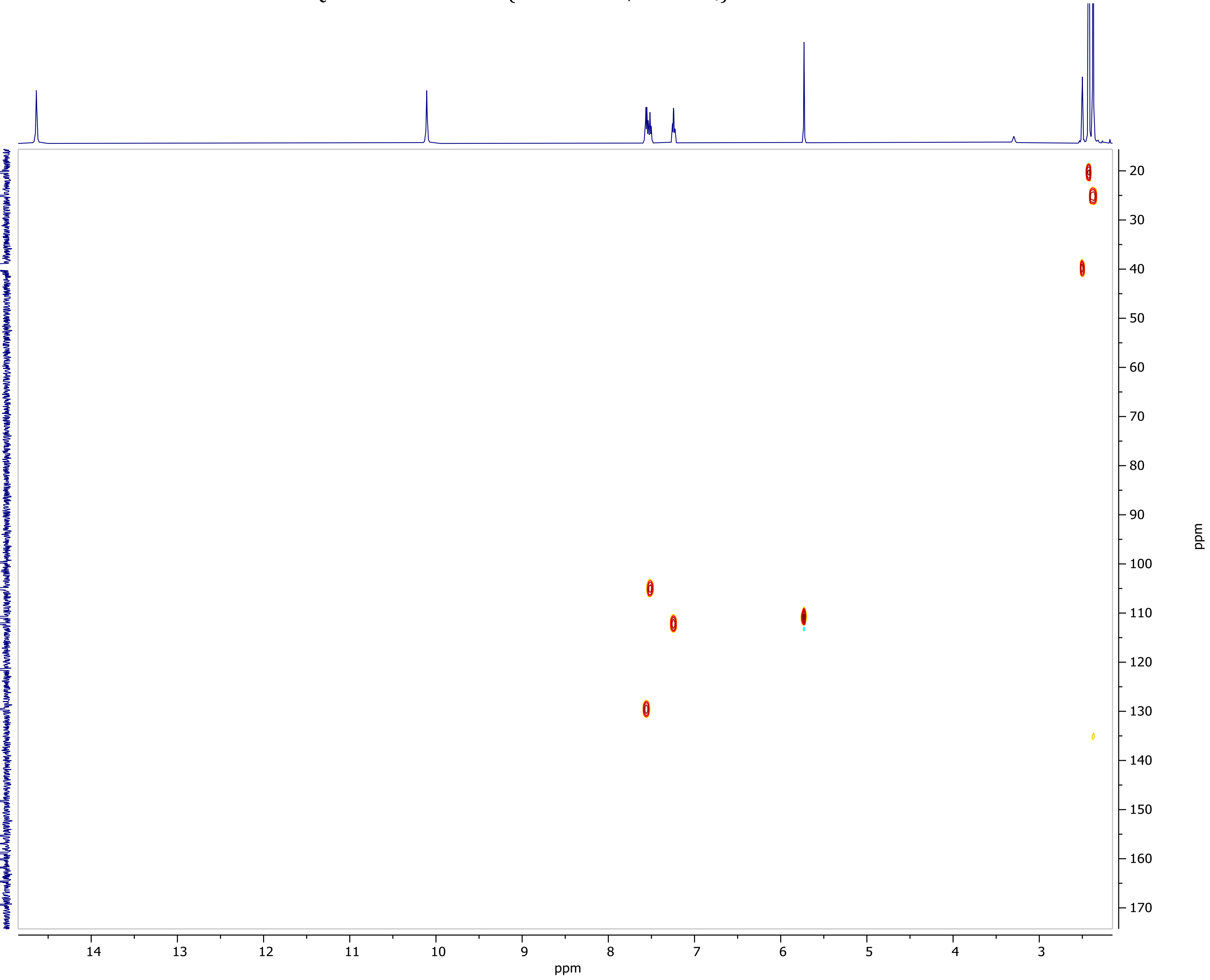

HMBC NMR of **26** (600.11 MHz, DMSO-d_6)

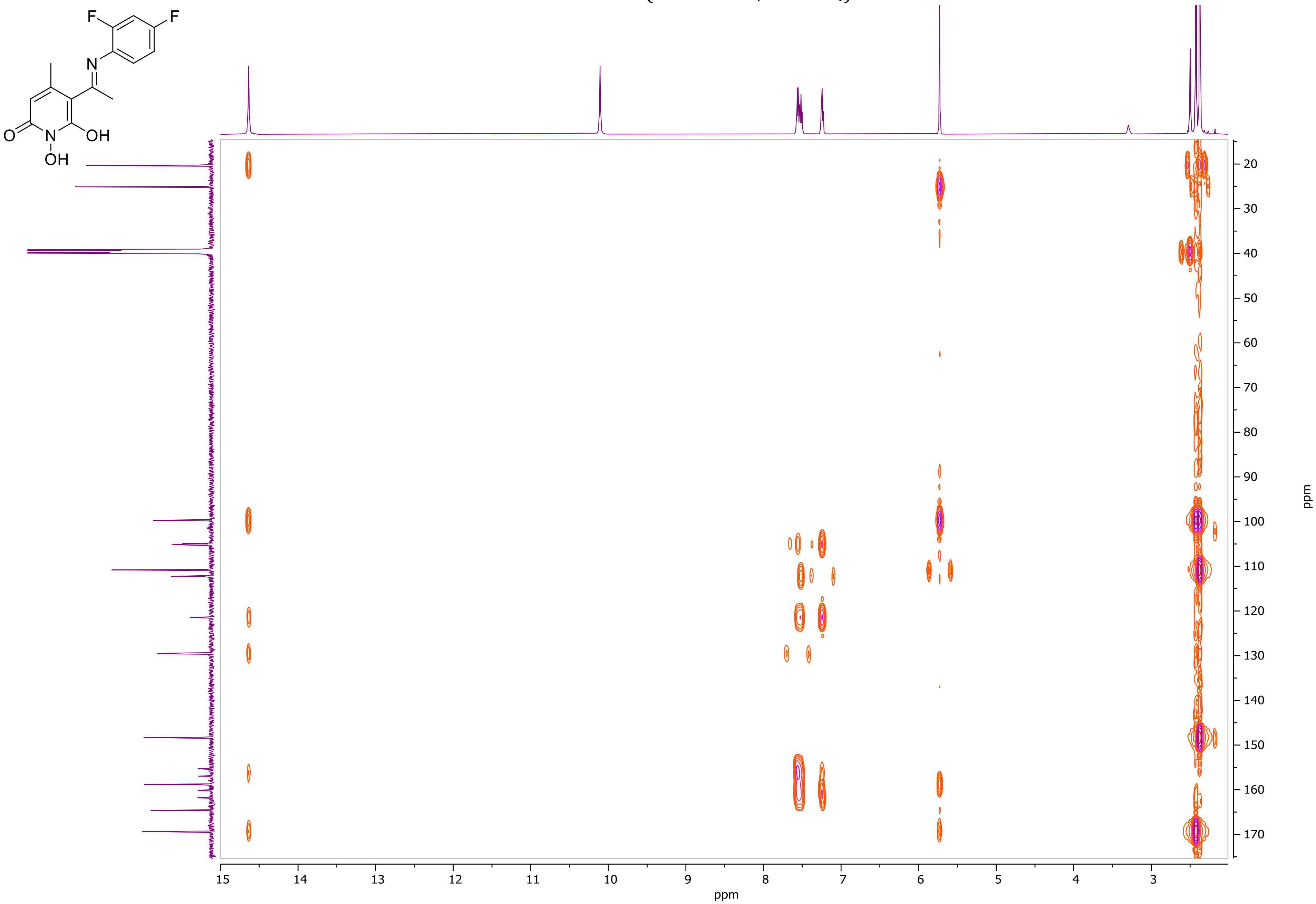

^{1}H NMR of **27** (600.11 MHz, DMSO-d_6)

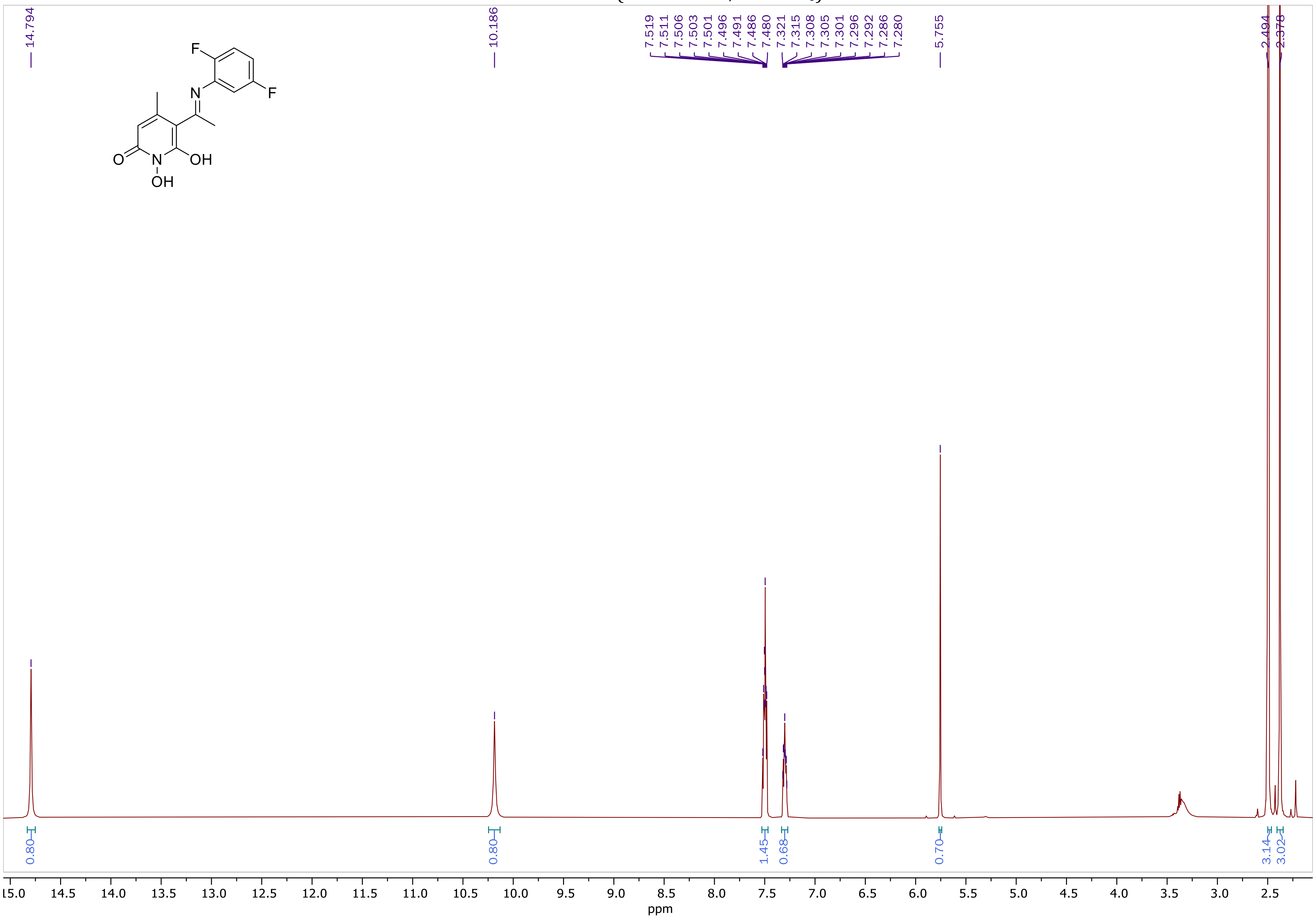

^{13}C NMR of **27** (150.9 MHz, DMSO-d_6)

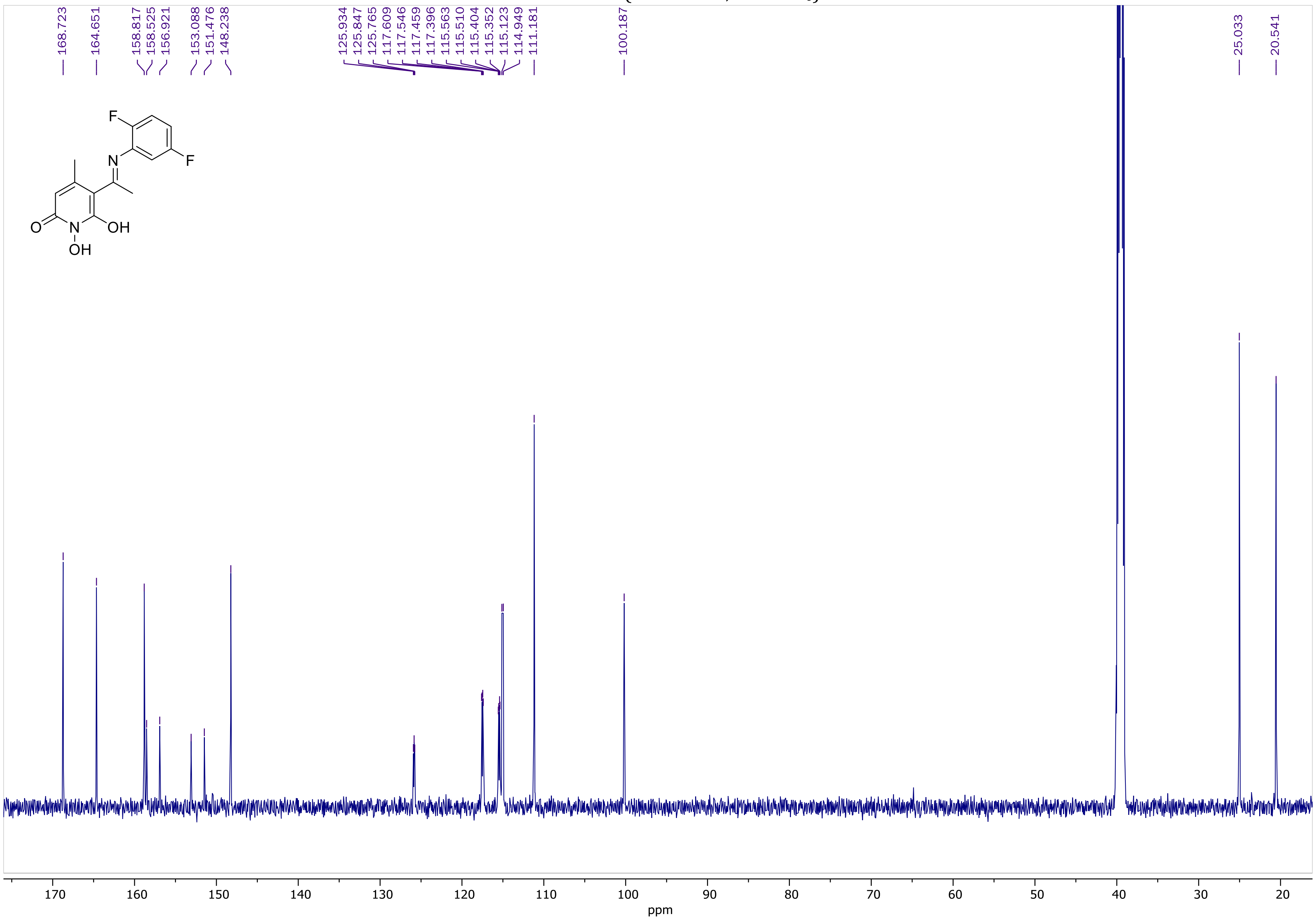

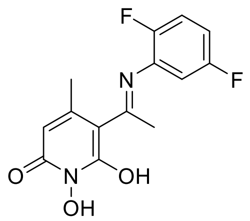

COSY NMR of **27** (600.11 MHz, DMSO-d_6)

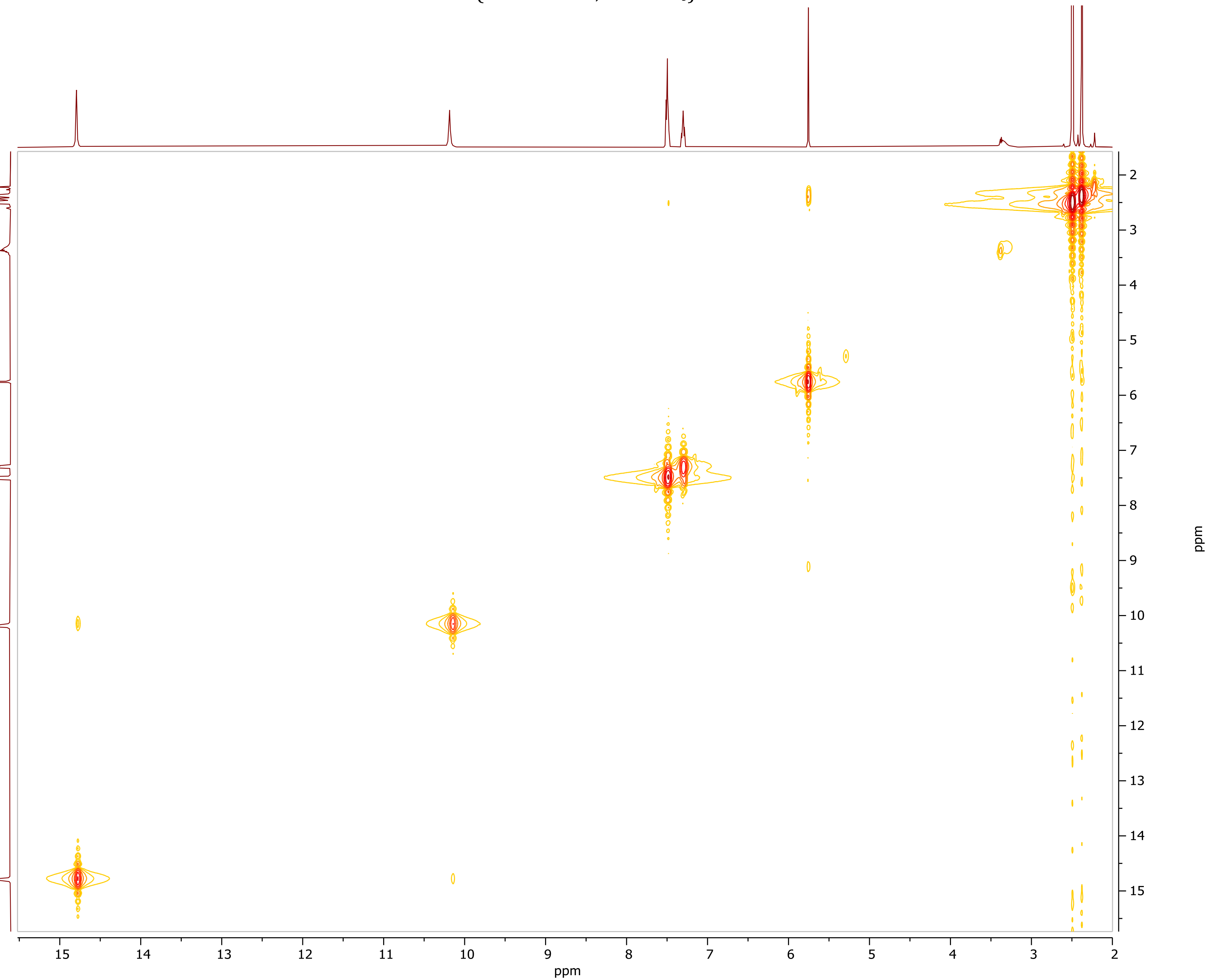

HSQC-DEPT NMR of **27** (600.11 MHz, DMSO-d_6)

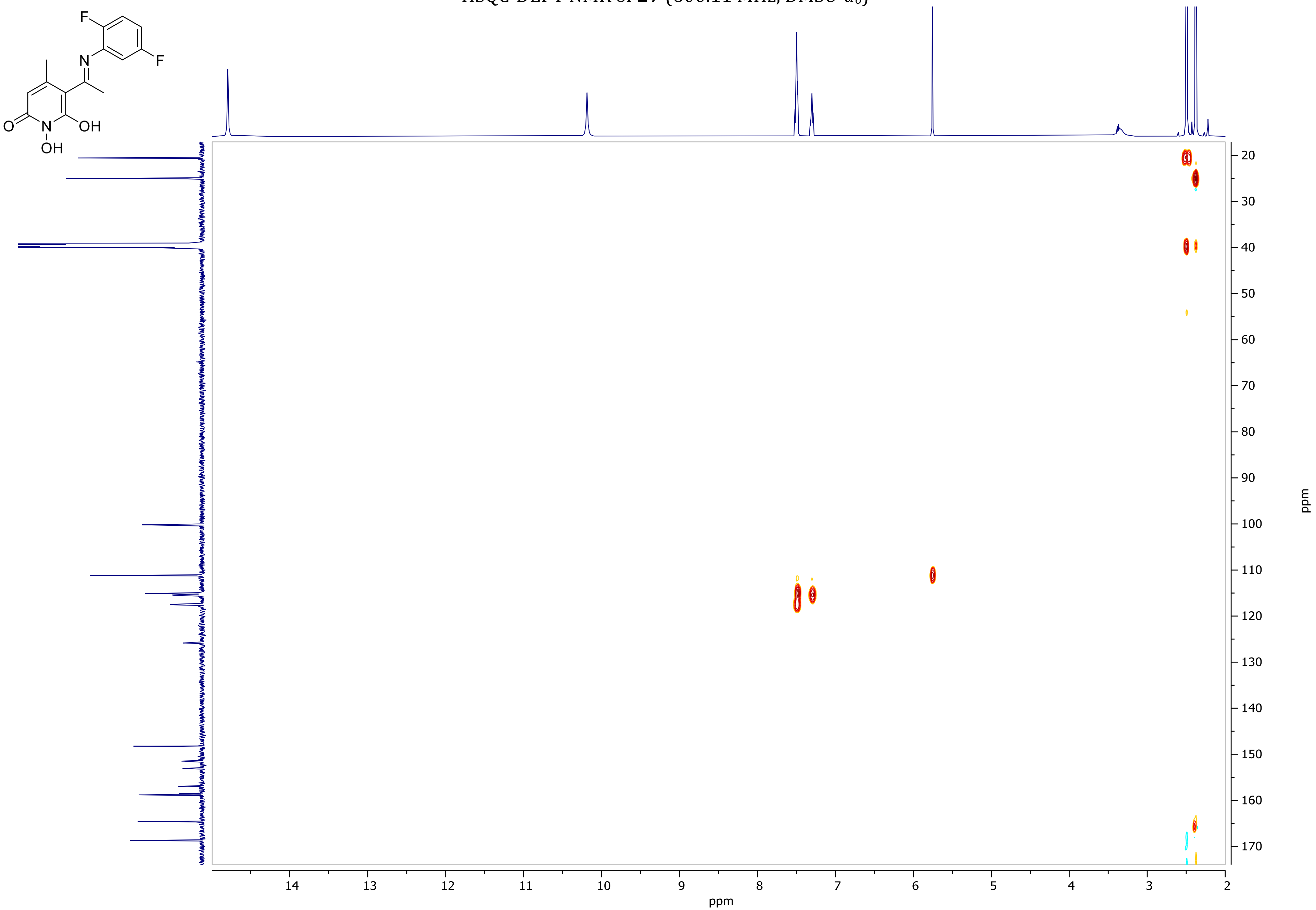

HMBC NMR of **27** (600.11 MHz, DMSO-d_6)

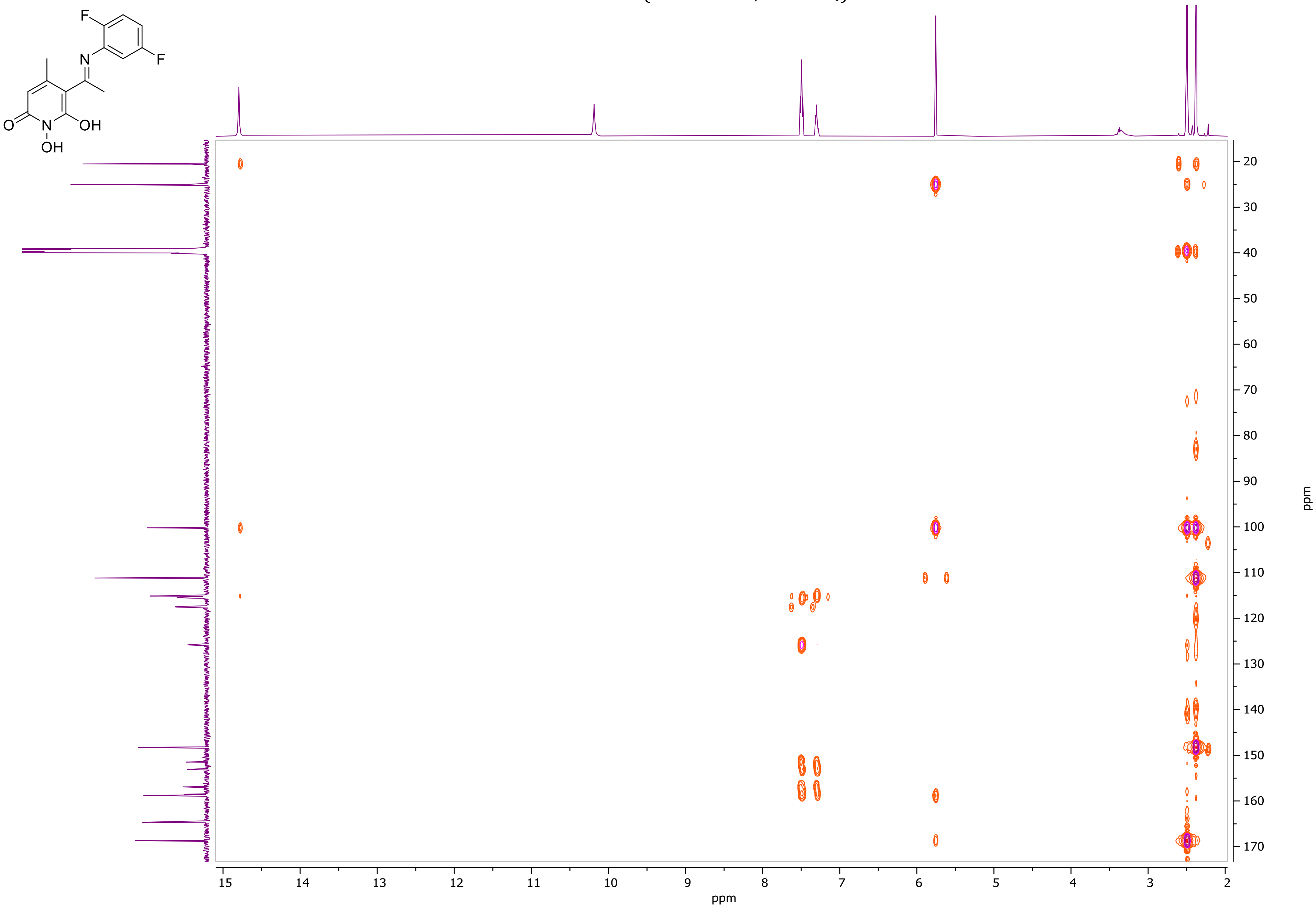

^{1}H NMR of **28** (600.11 MHz, DMSO-d_6)

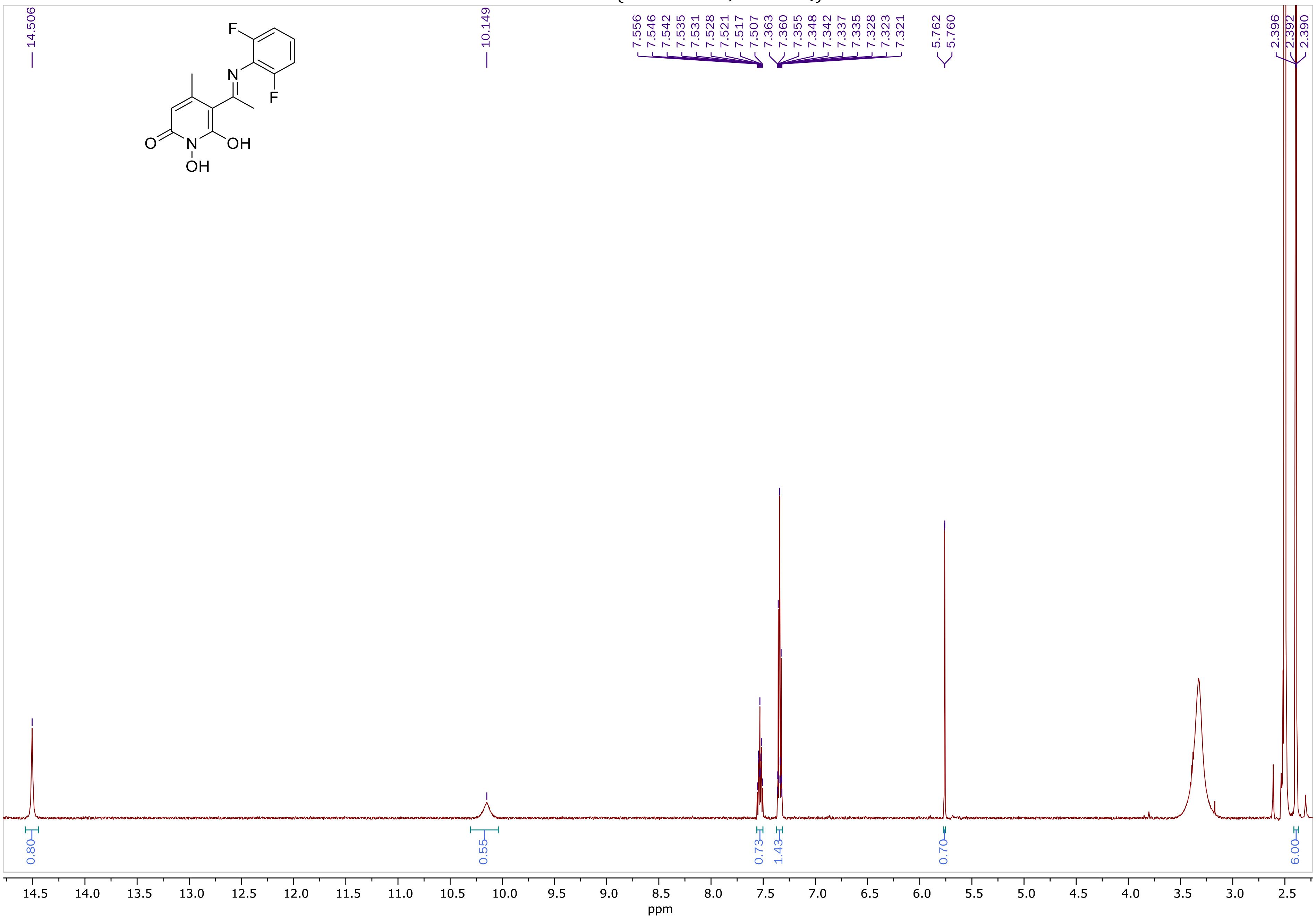

^{13}C NMR of **28** (150.9 MHz, DMSO-d_6)

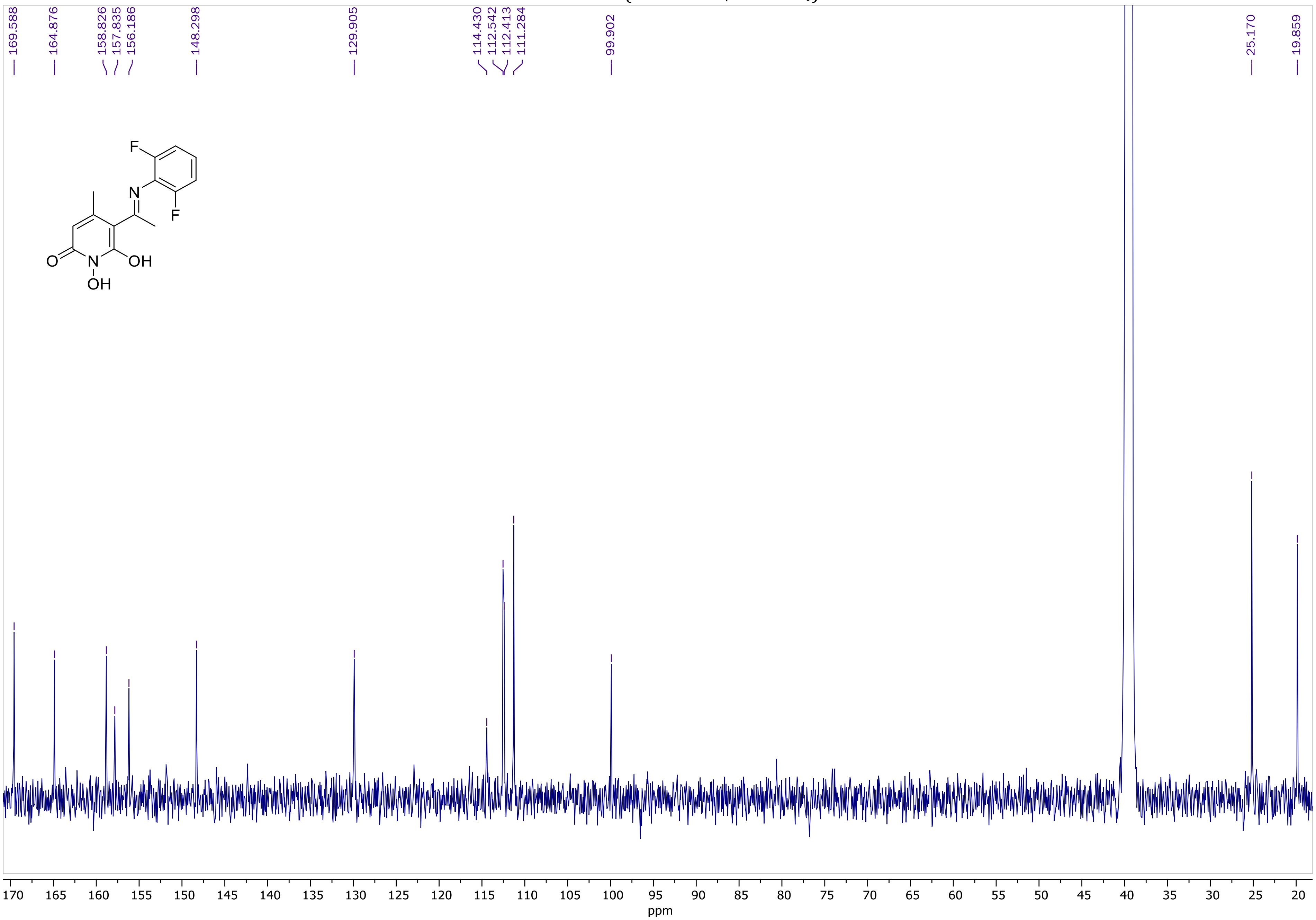

COSY NMR of **28** (600.11 MHz, DMSO-d_6)

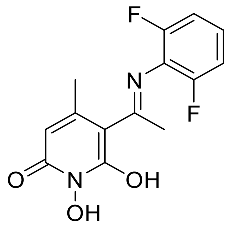

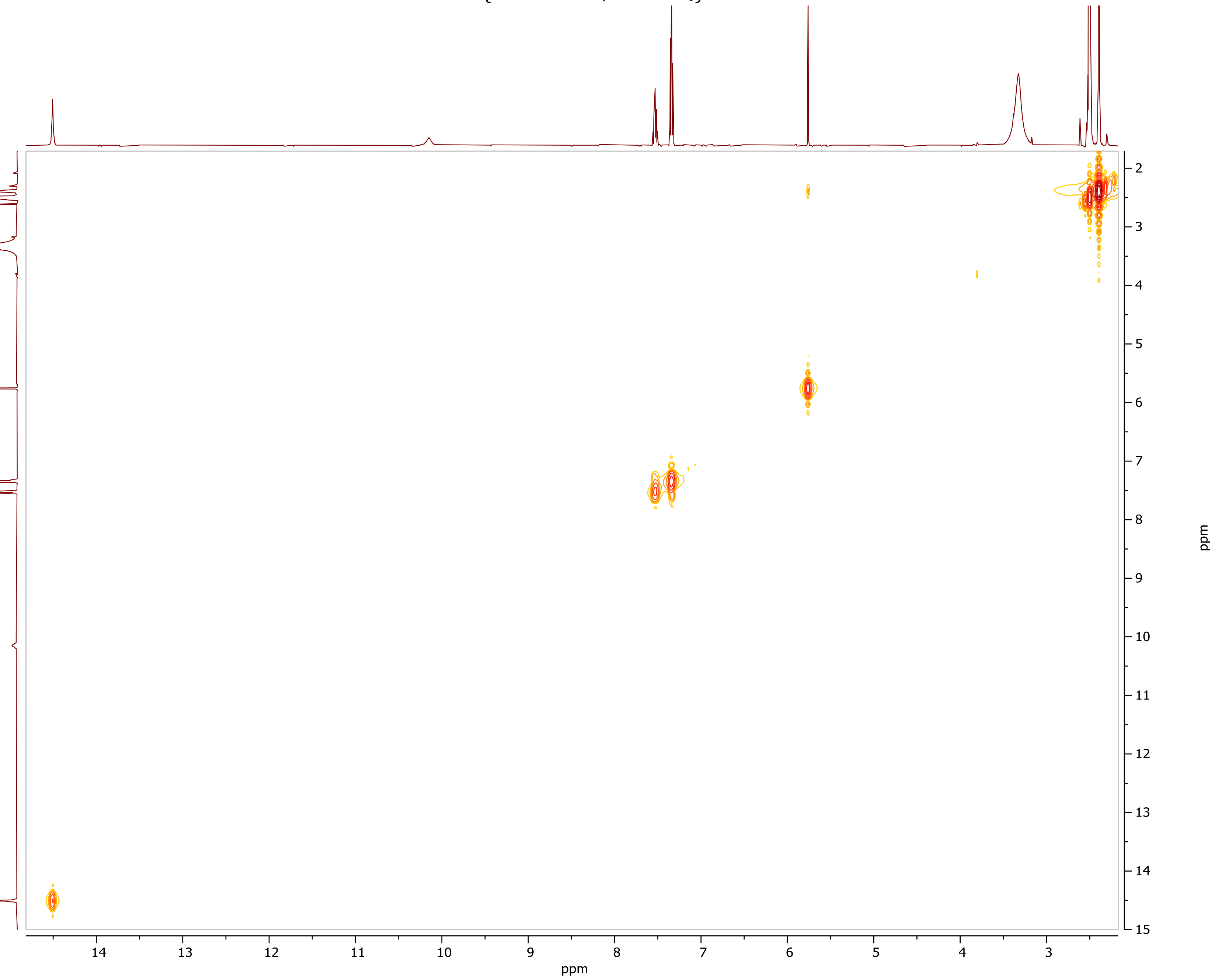

HSQC-DEPT NMR of **28** (600.11 MHz, DMSO-d_6)

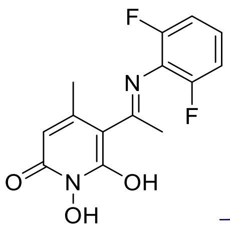

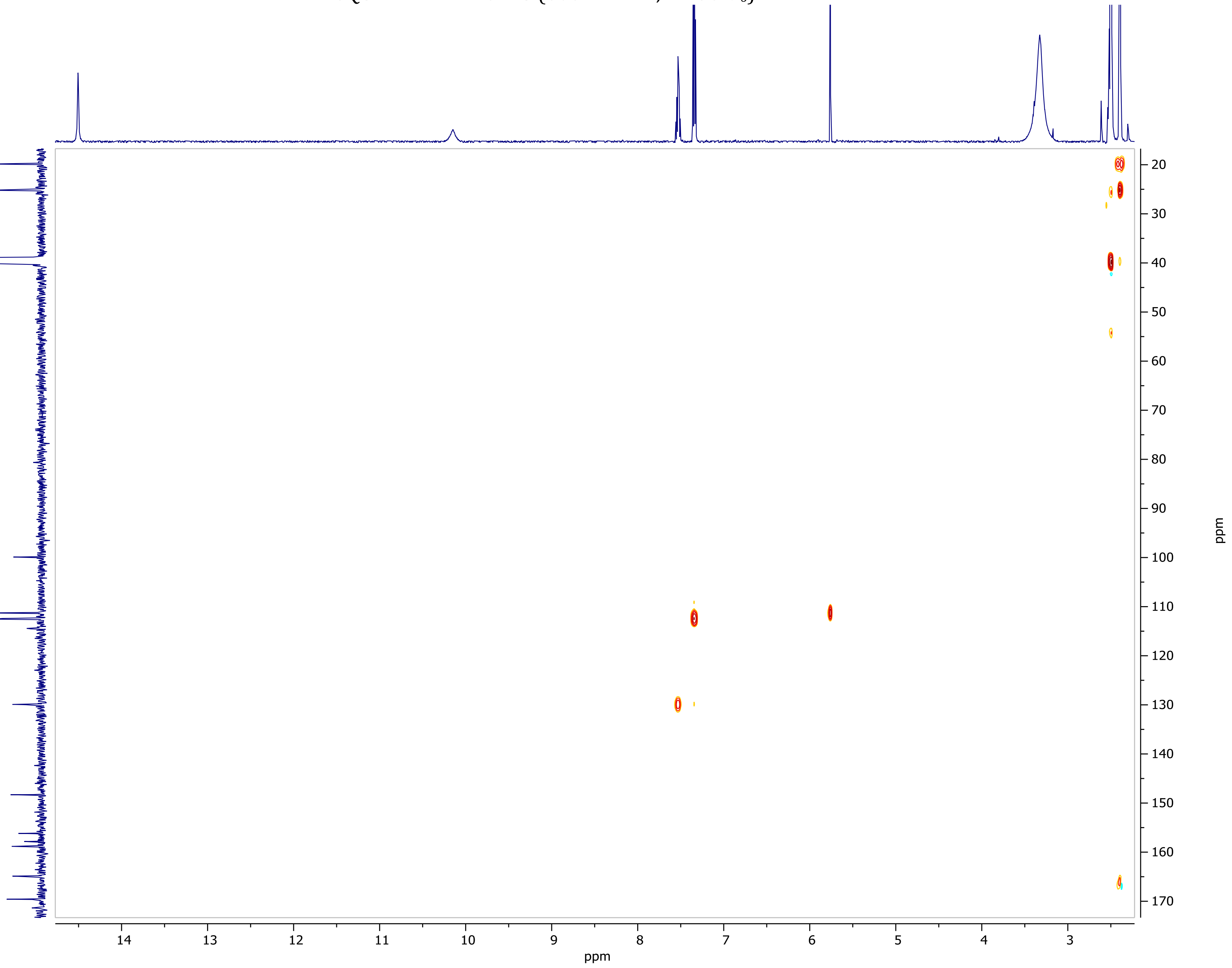

HMBC NMR of **28** (600.11 MHz, DMSO-d_6)

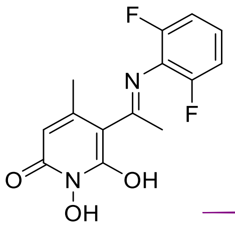

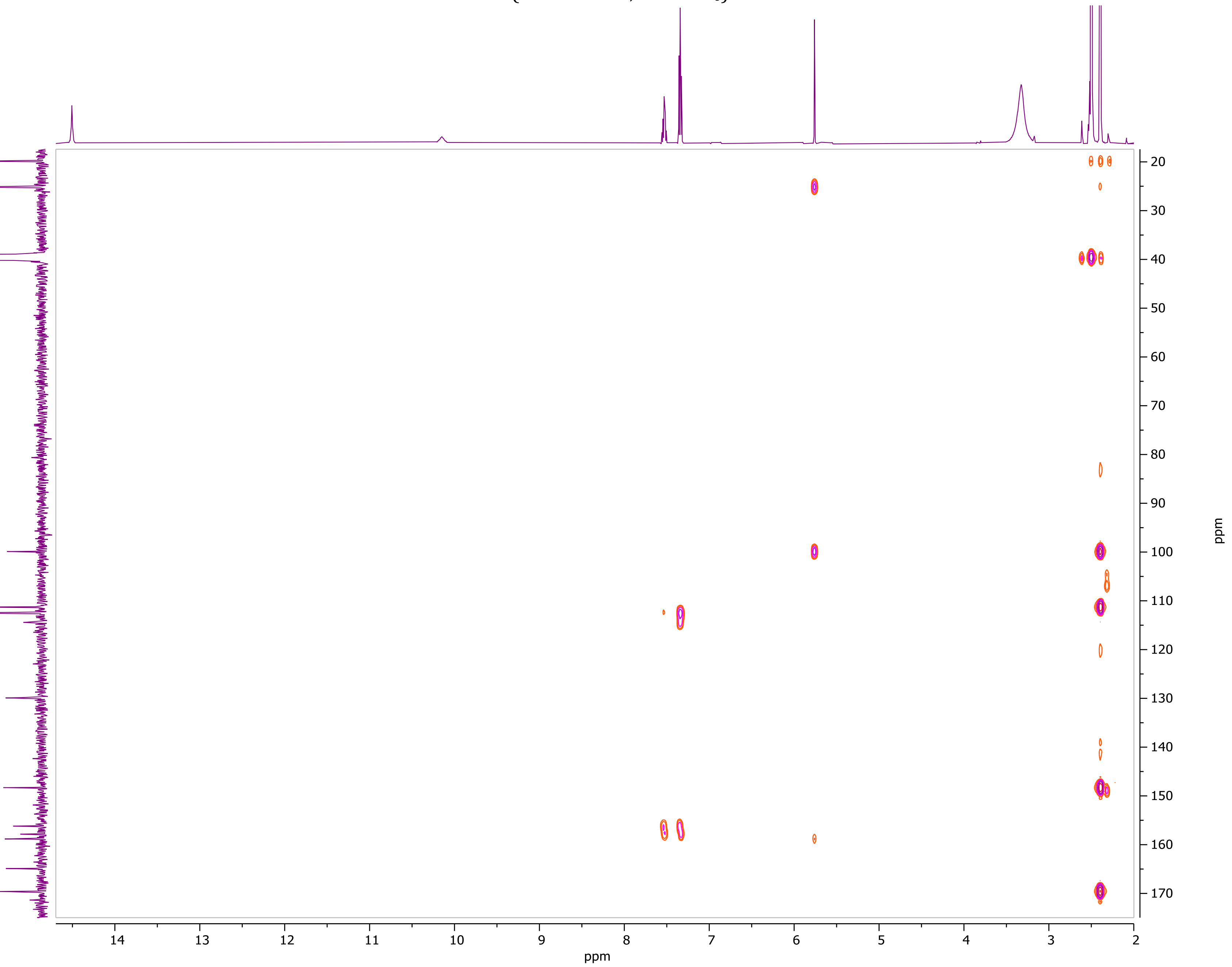

^{1}H NMR of **29** (600.11 MHz, DMSO-d_6)

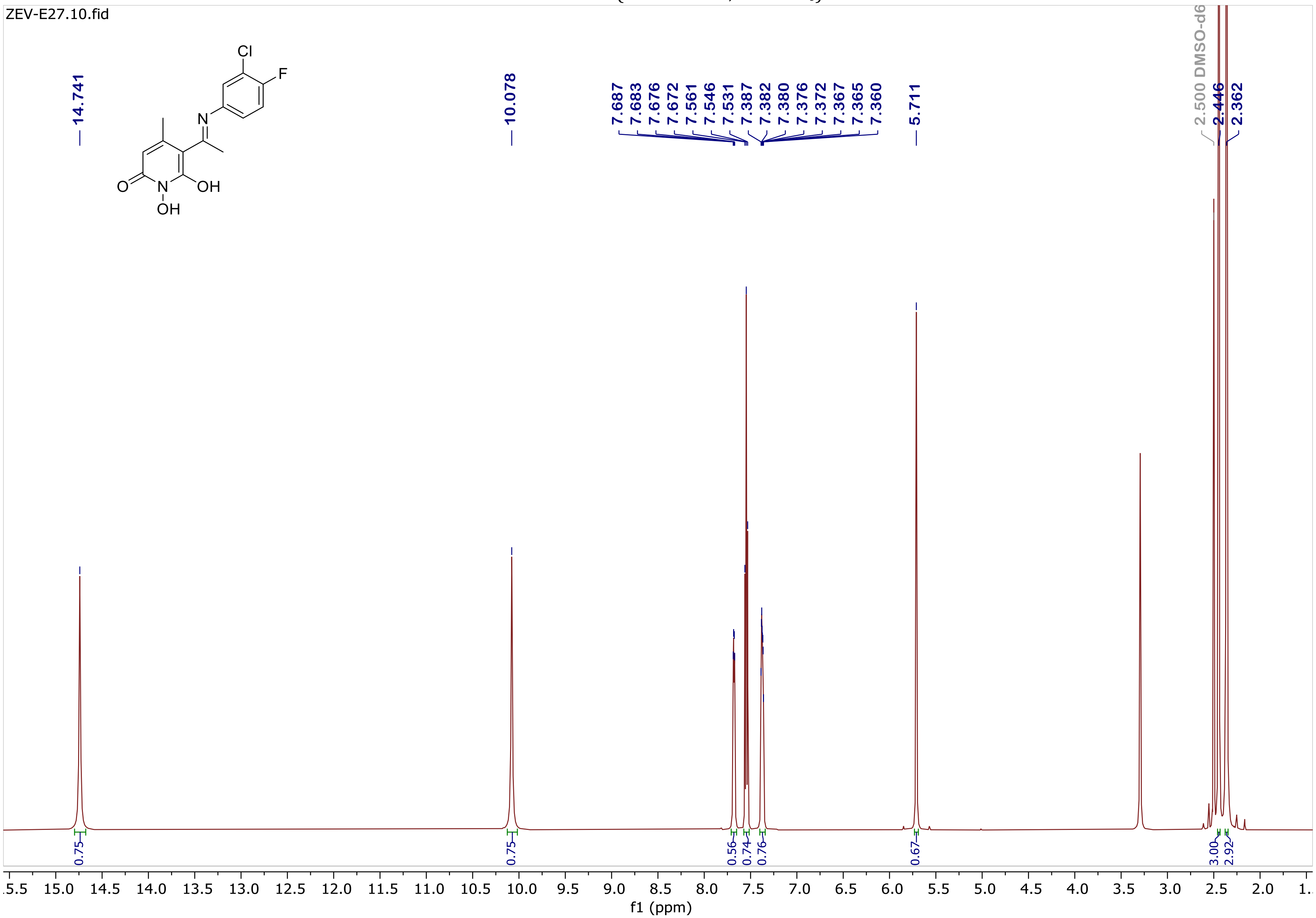

^{13}C NMR of **29** (150.9 MHz, DMSO-d_6)

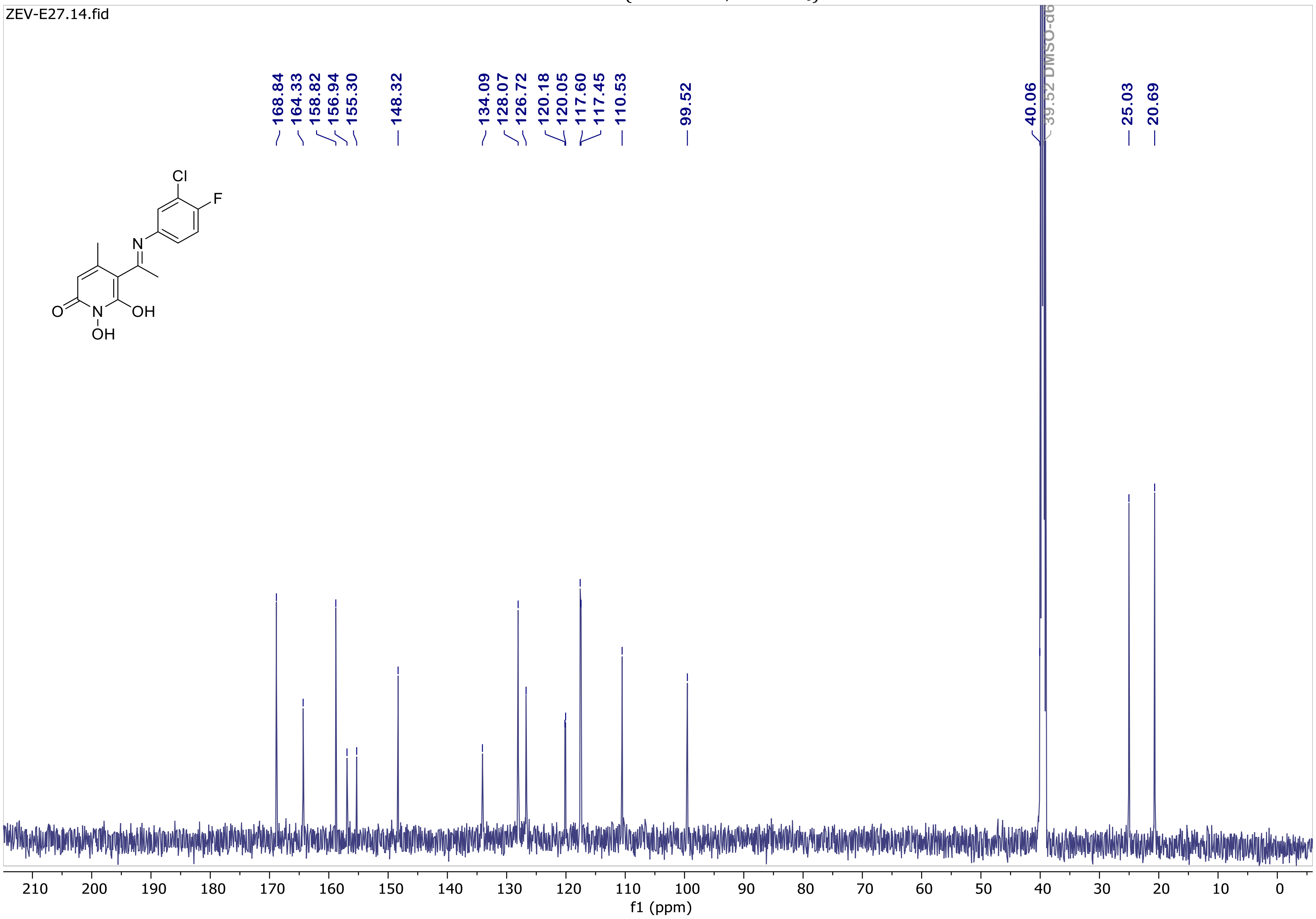

COSY NMR of **29** (600.11 MHz, DMSO-d_6)

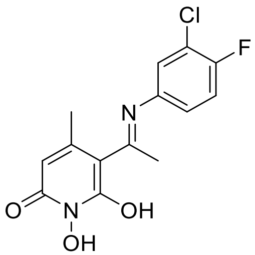

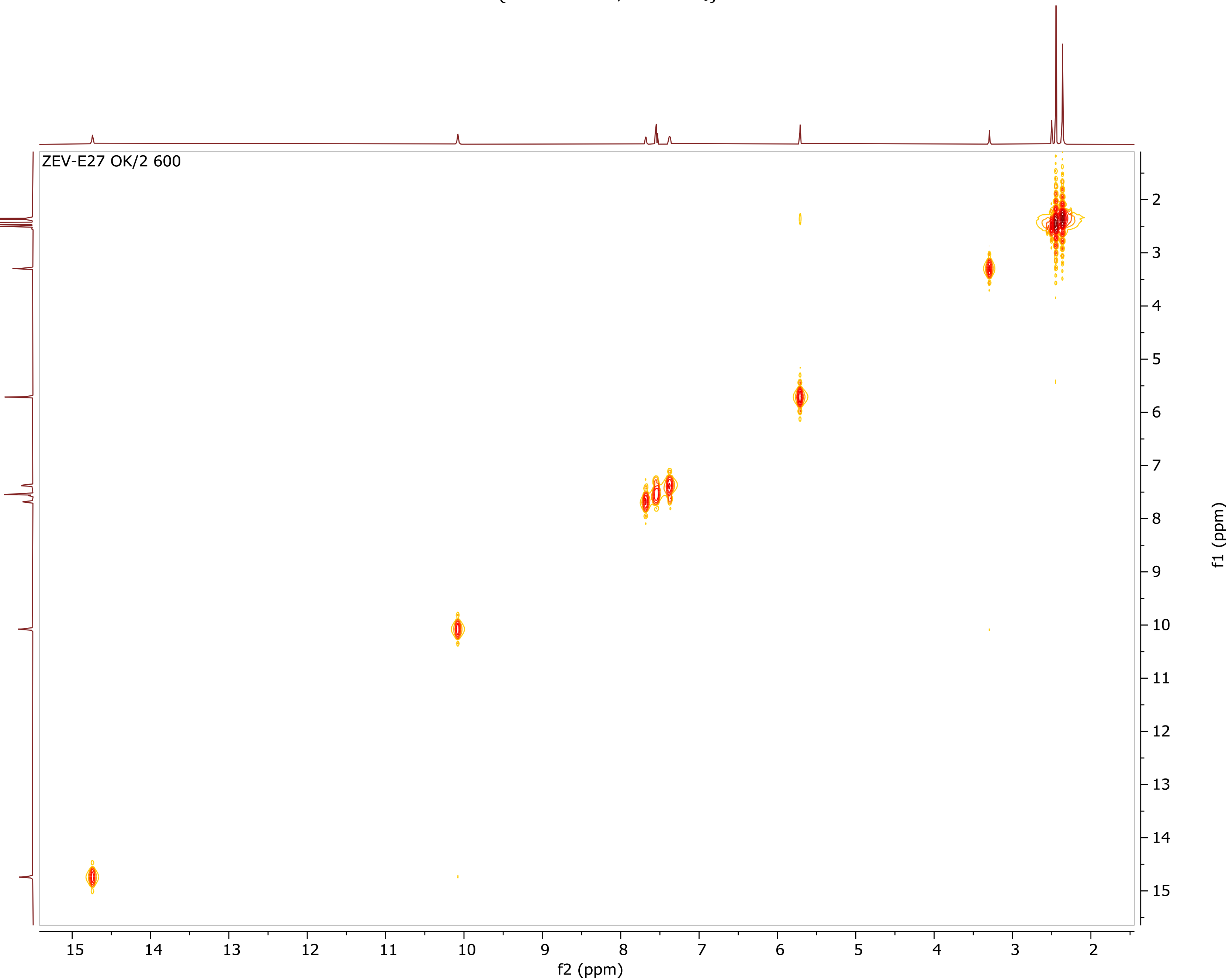

HSQC-DEPT NMR of **29** (600.11 MHz, DMSO-d_6)

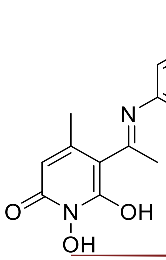

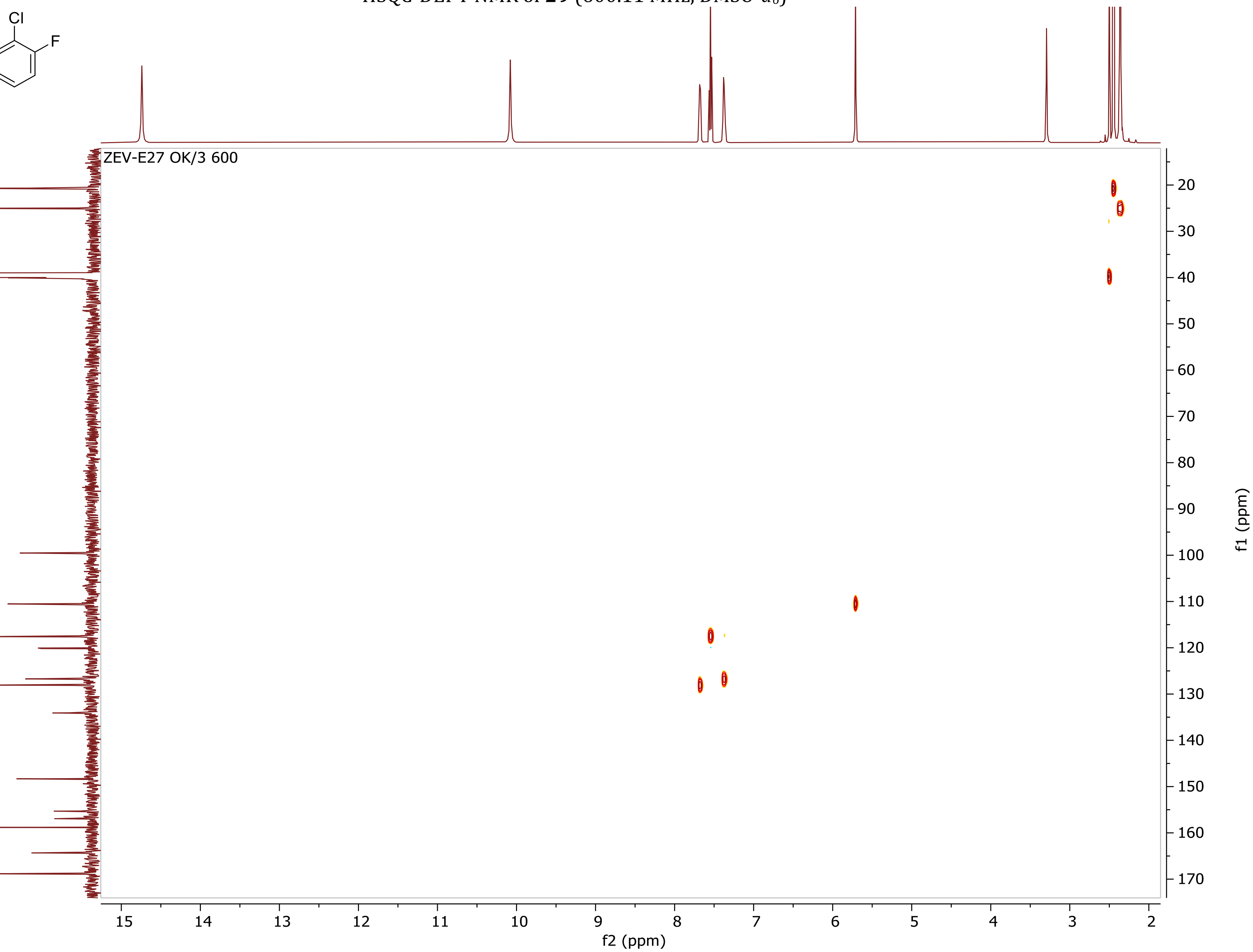

HMBC NMR of **29** (600.11 MHz, DMSO-d_6)

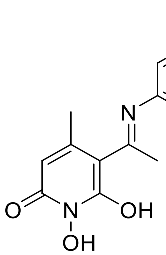

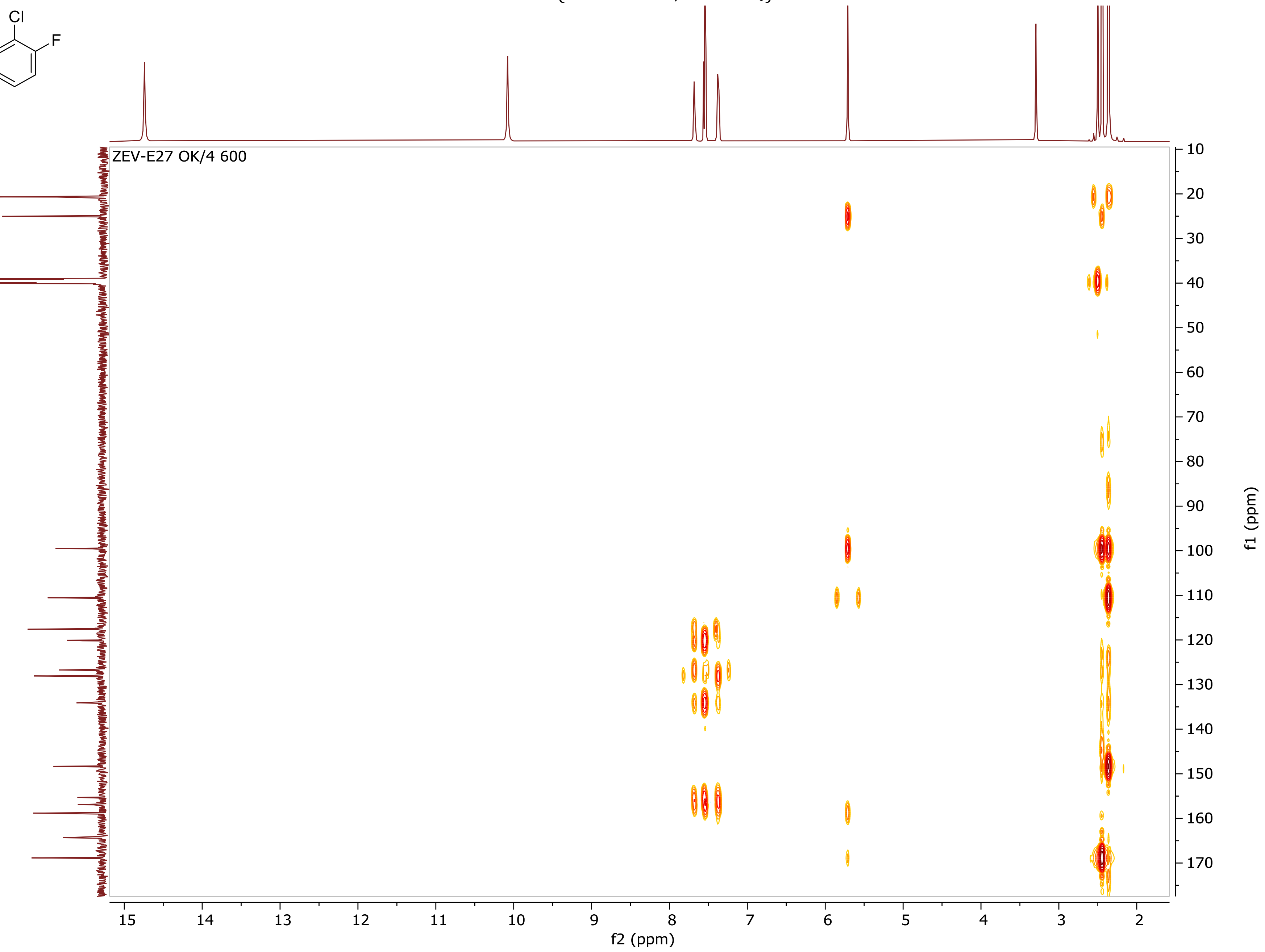

^{1}H NMR of **30** (600.11 MHz, DMSO-d_6)

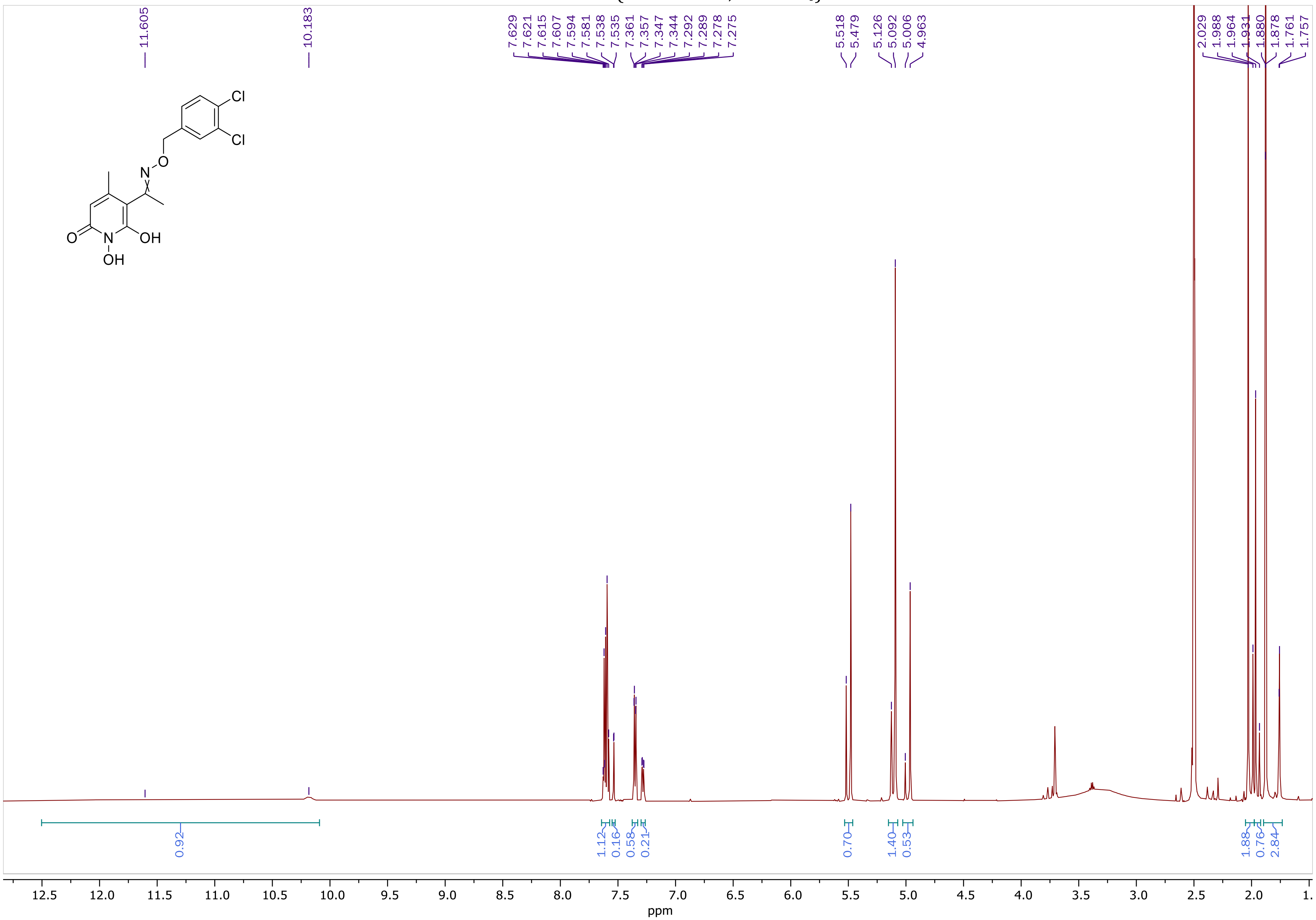

^{13}C NMR of **30** (150.9 MHz, DMSO-d_6)

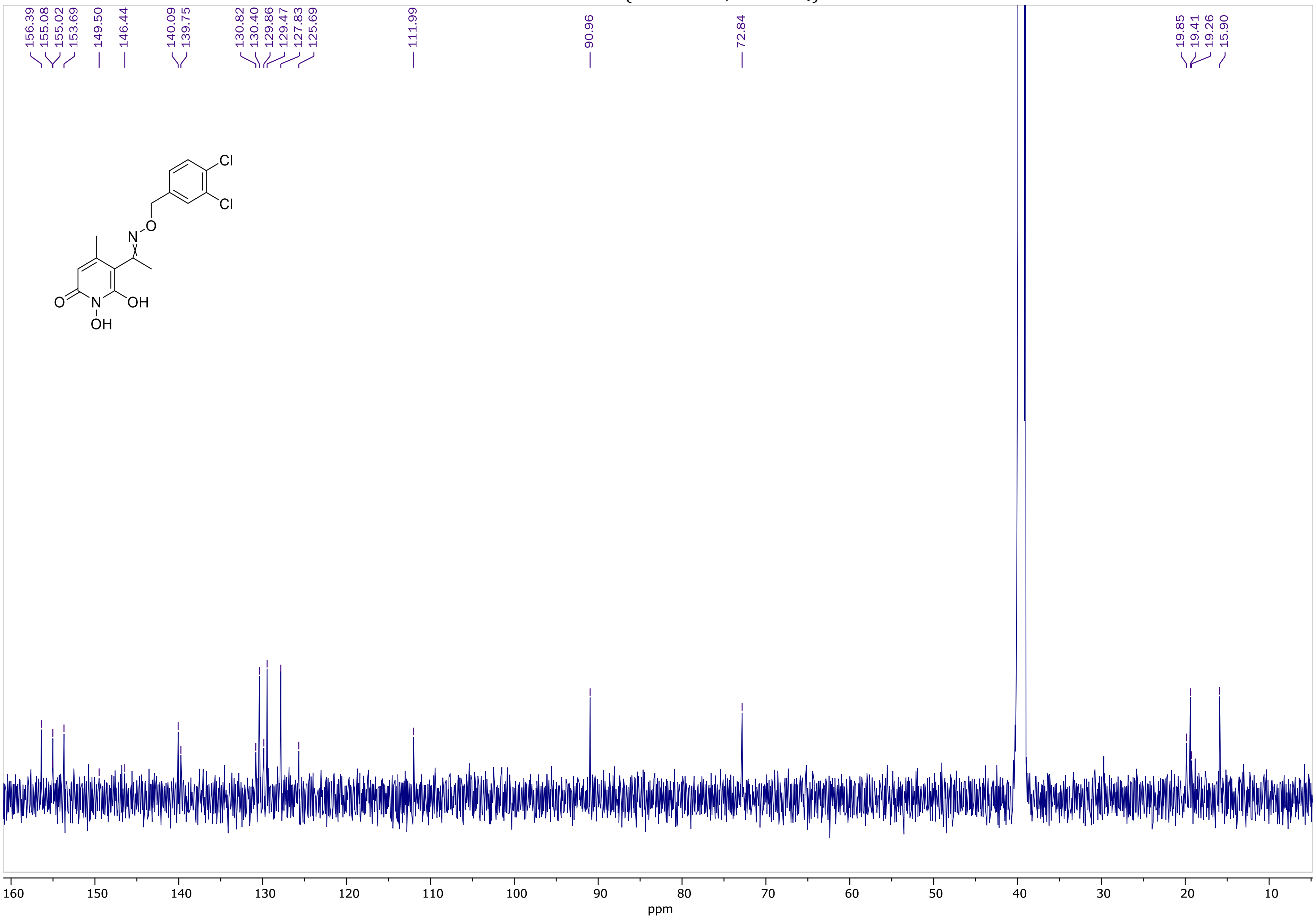

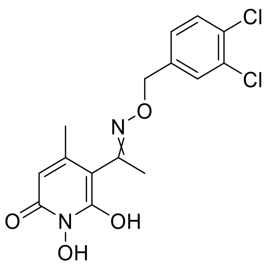

COSY NMR of **30** (600.11 MHz, DMSO-d_6)

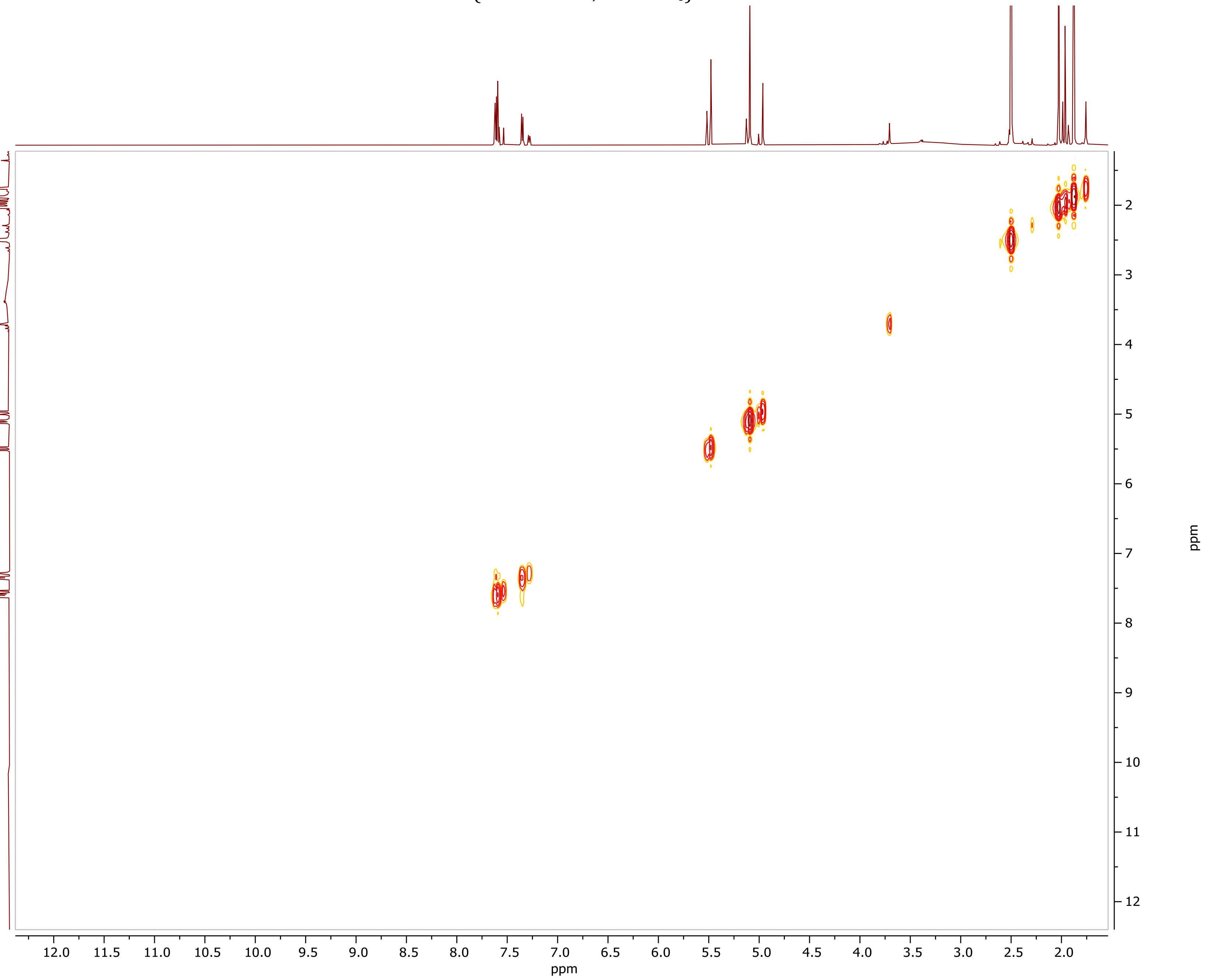

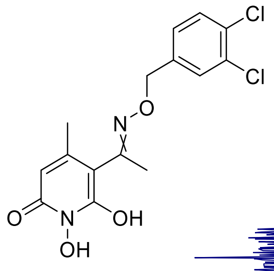

HSQC-DEPT NMR of **30** (600.11 MHz, DMSO-d_6)

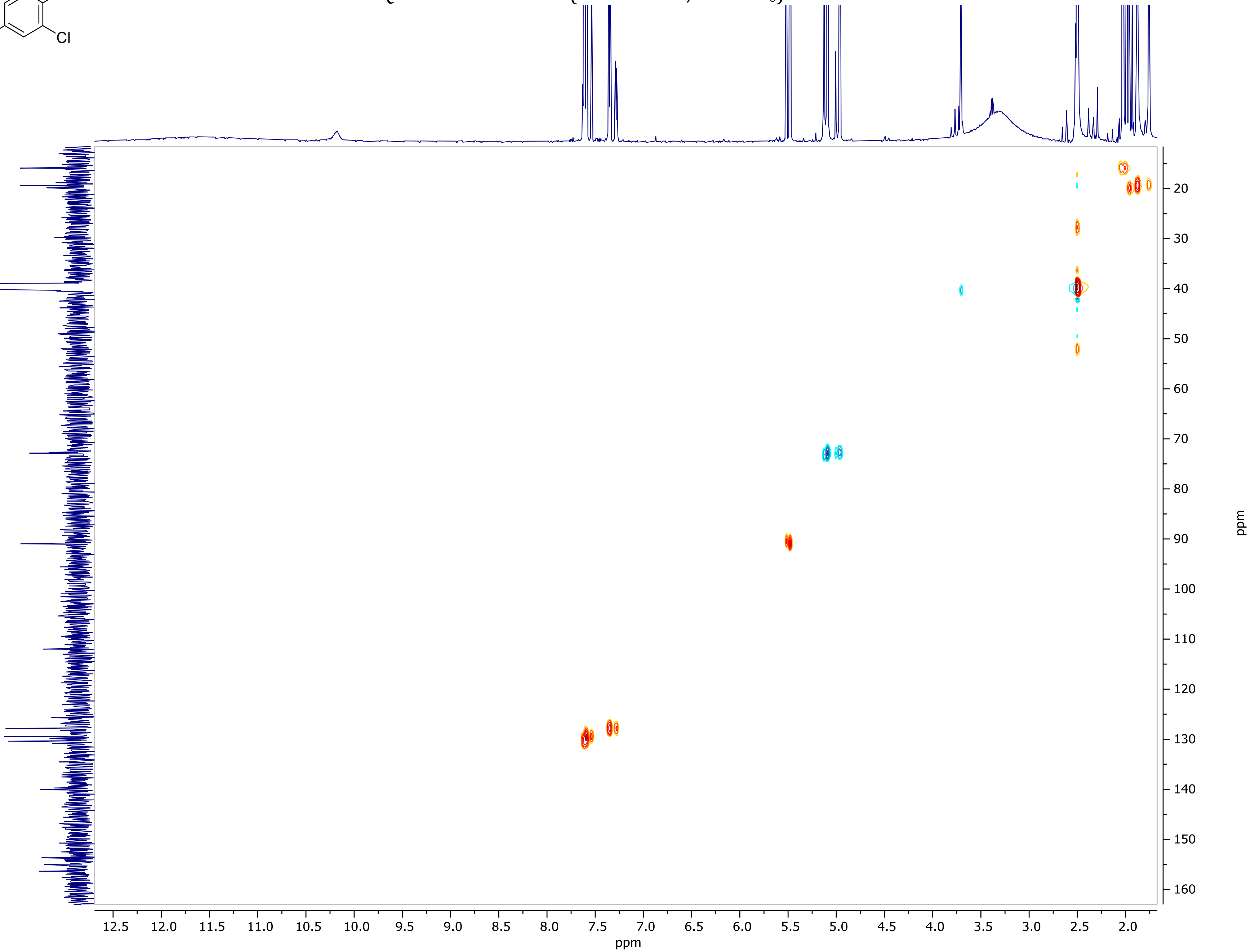

HMBC NMR of **30** (600.11 MHz, DMSO-d_6)

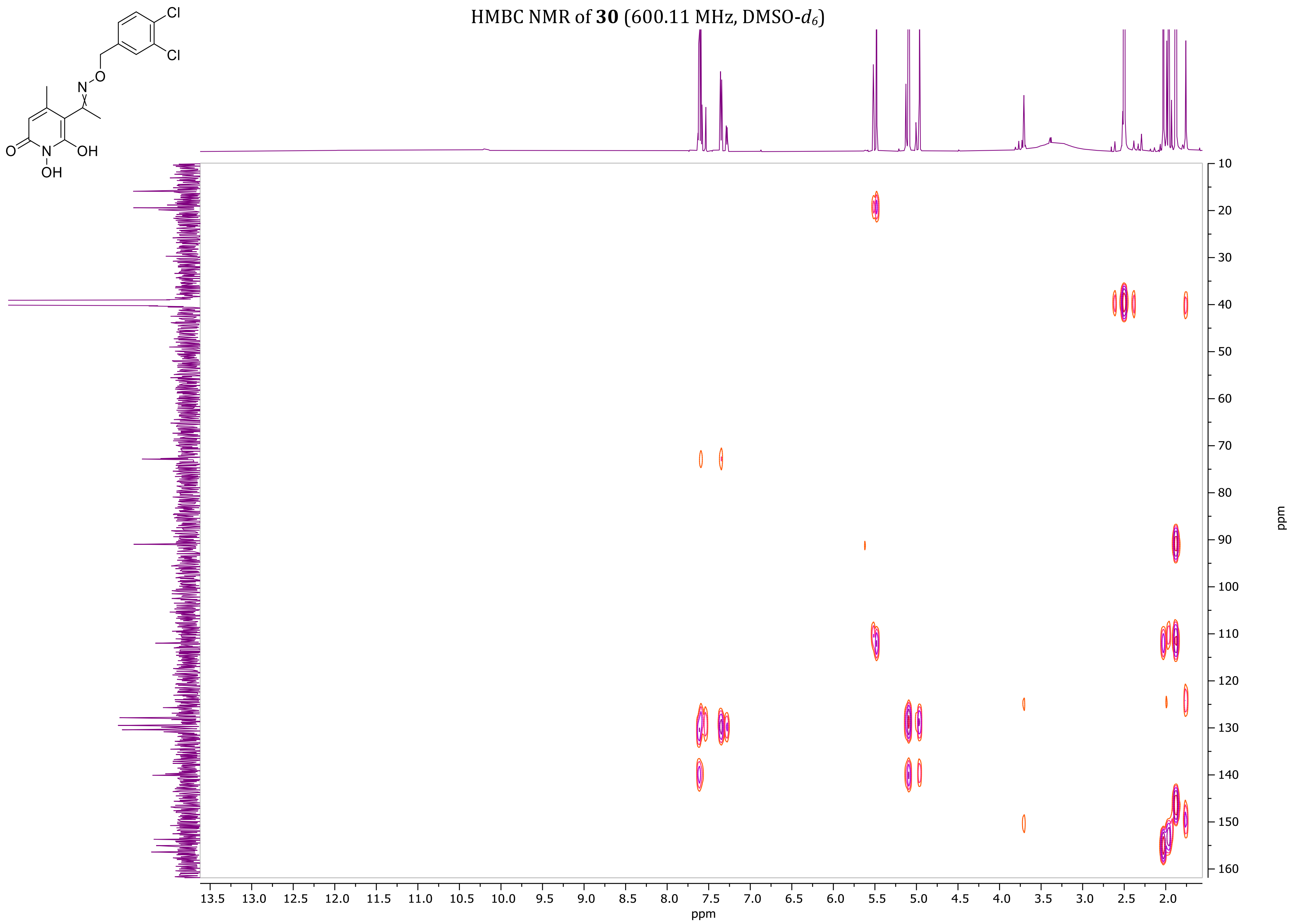

^{1}H NMR of **31** (600.11 MHz, DMSO-d_6)

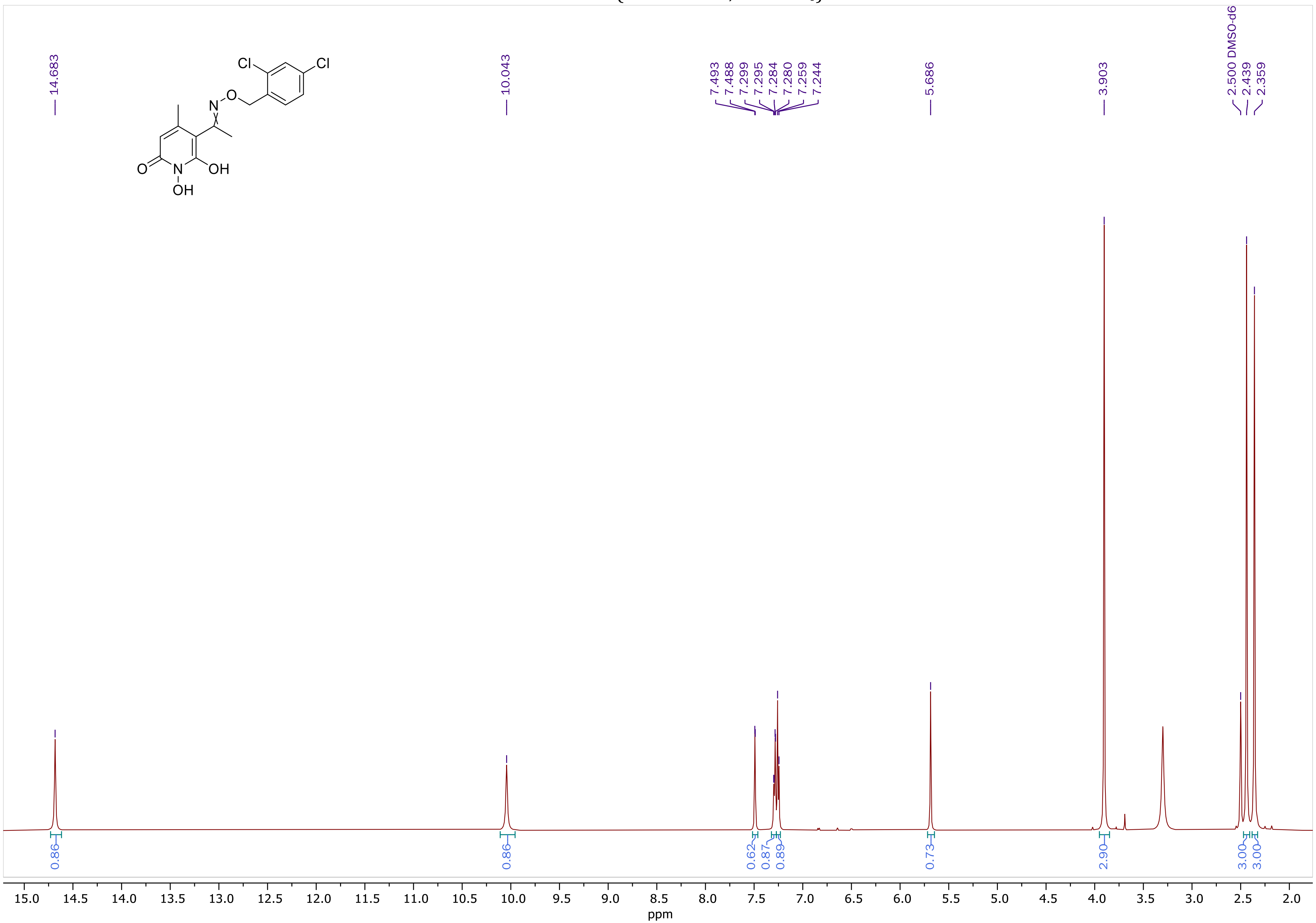

^{13}C NMR of **31** (150.9 MHz, DMSO-d_6)

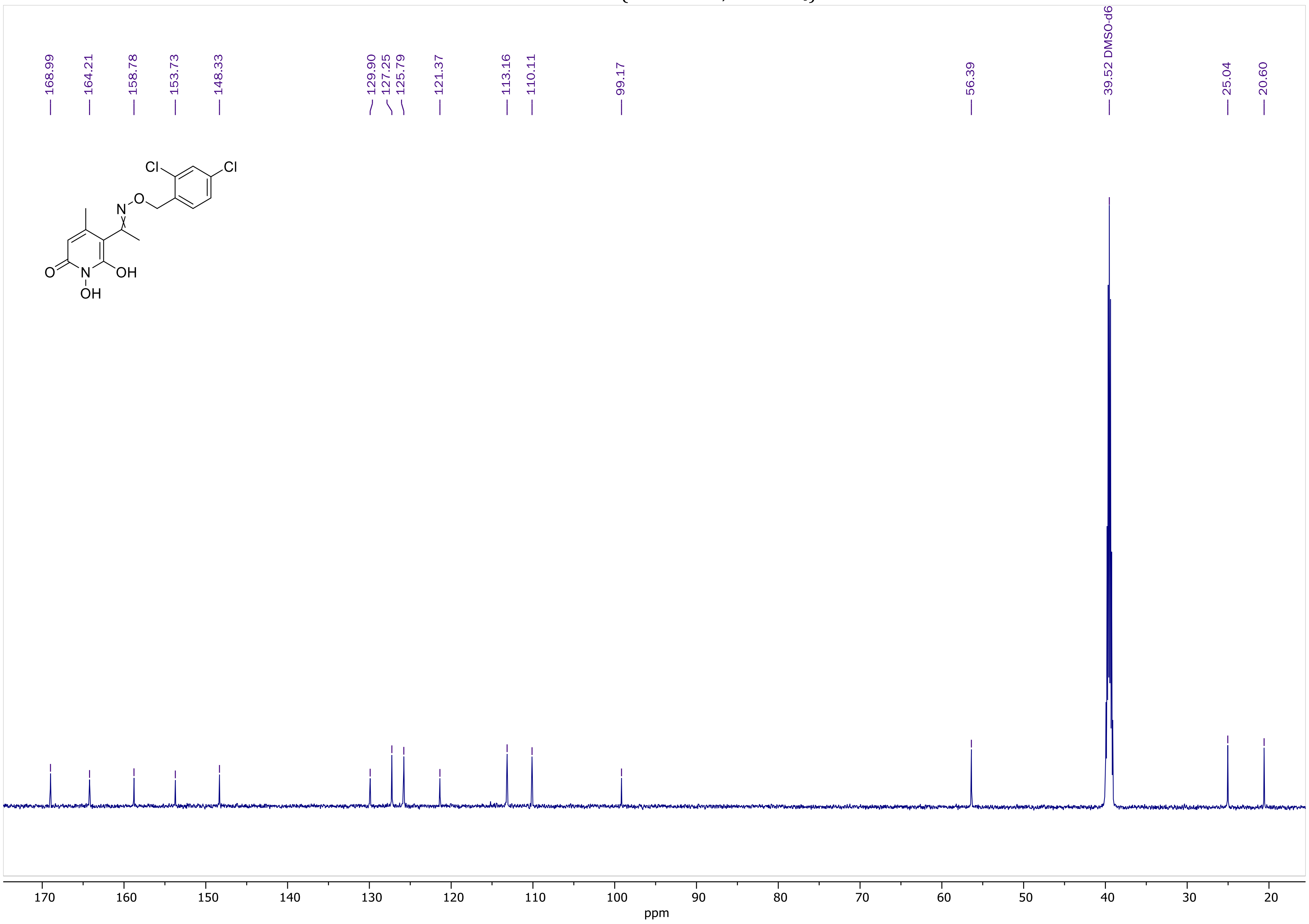

COSY NMR of **31** (600.11 MHz, DMSO-d_6)

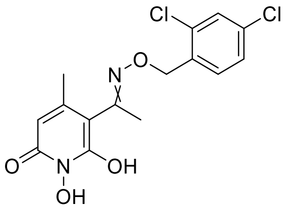

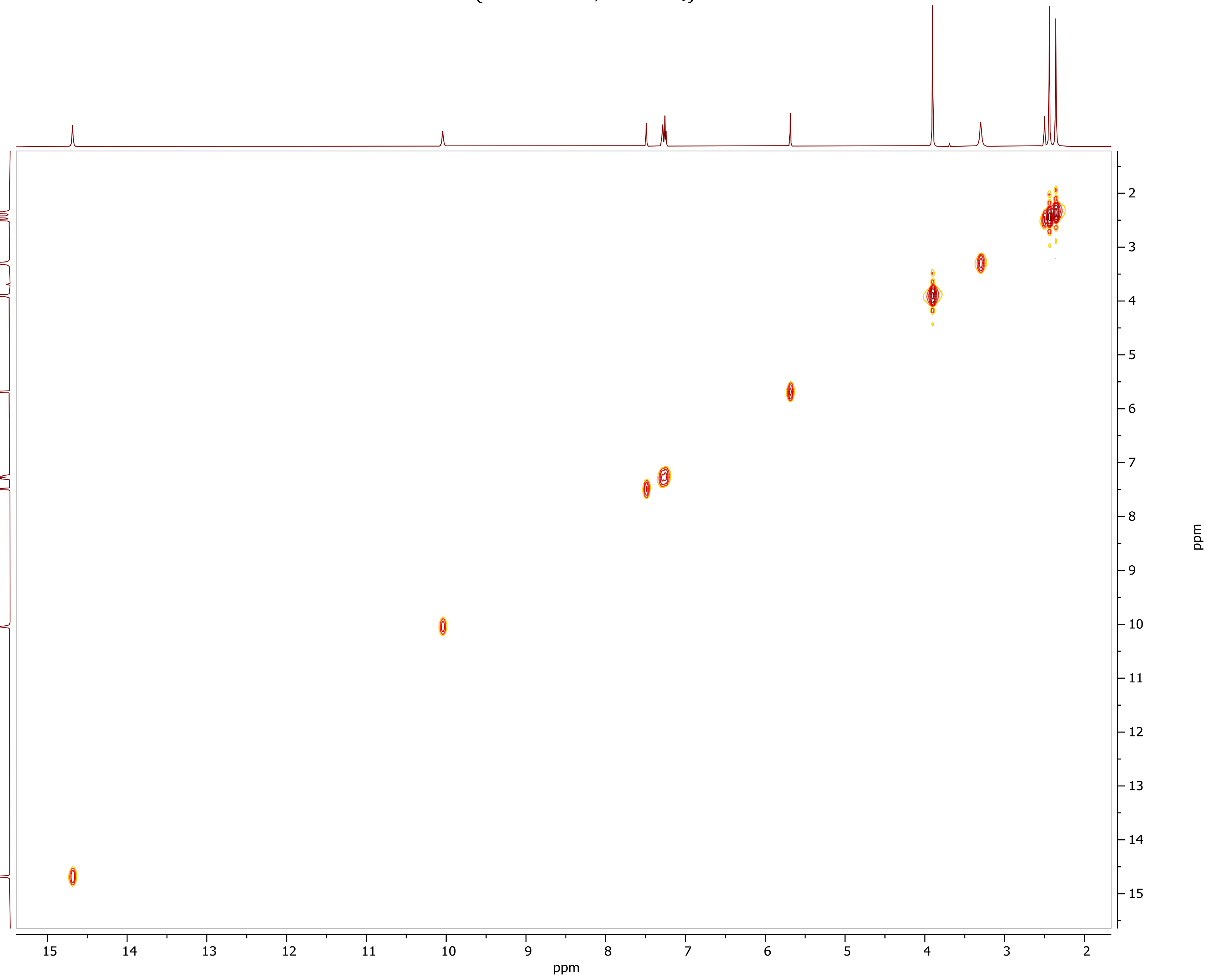

HSQC-DEPT NMR of **31** (600.11 MHz, DMSO-d_6)

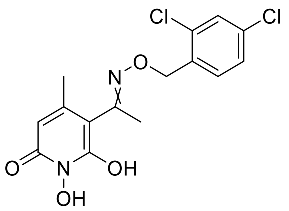

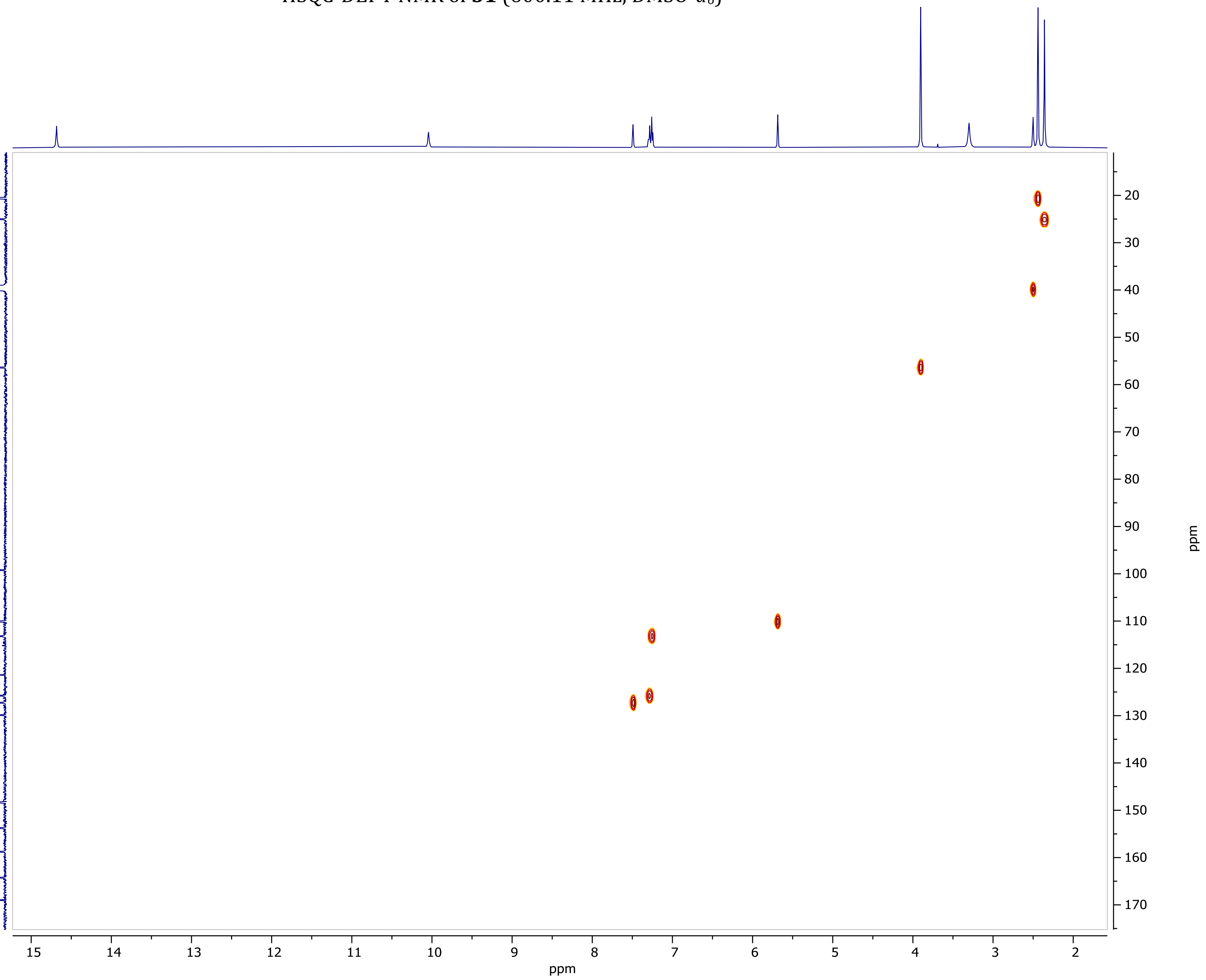

HMBC NMR of **31** (600.11 MHz, DMSO-d_6)

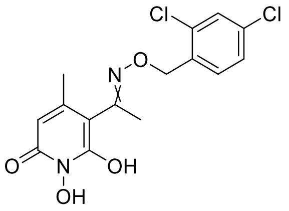

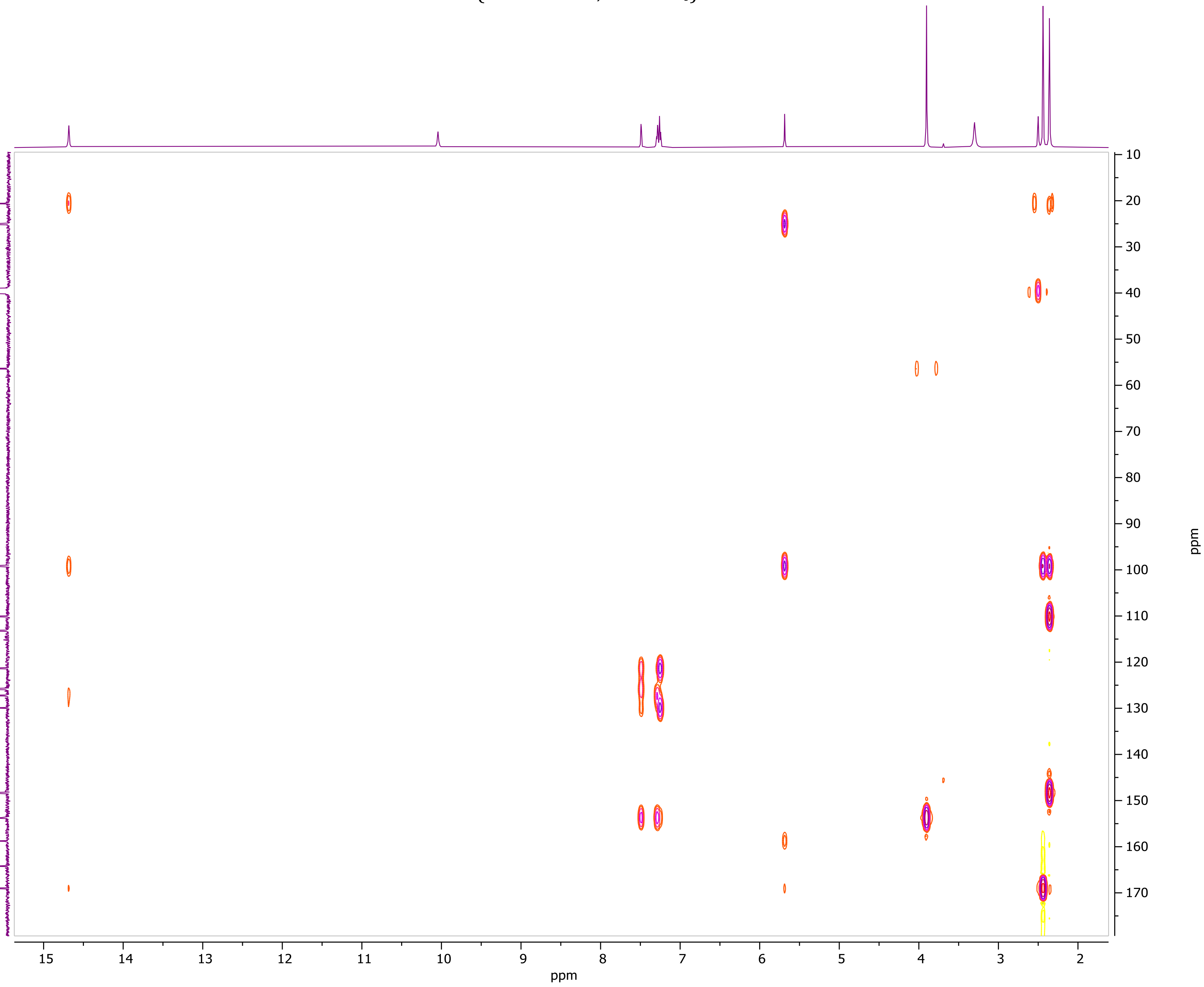

^{1}H NMR of **34** (500 MHz, DMSO-d_6)

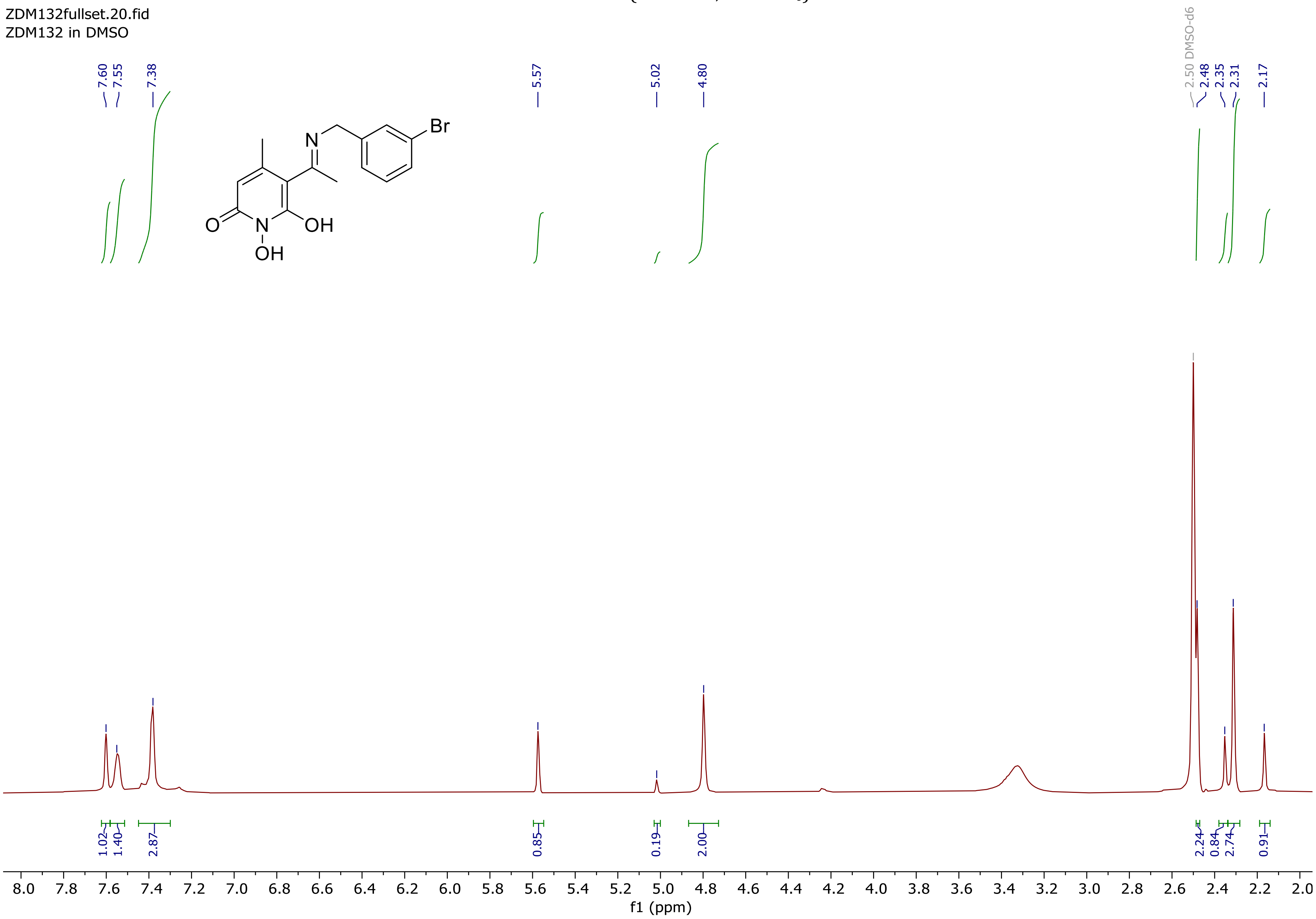

^{13}C NMR of **34** (126 MHz, DMSO-d_6)

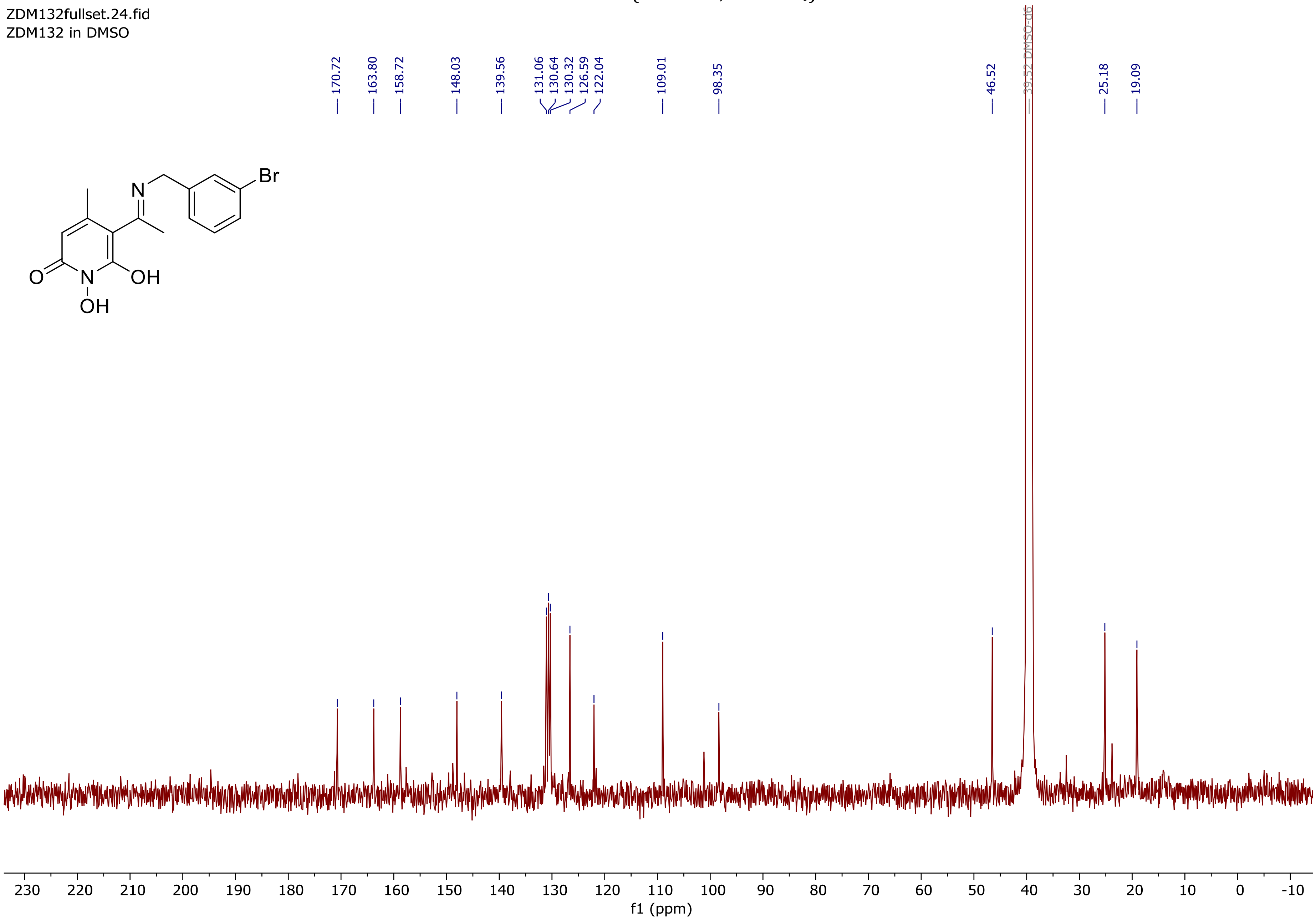

COSY NMR of **34** (500 MHz, DMSO-d_6)

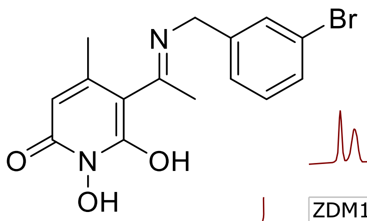

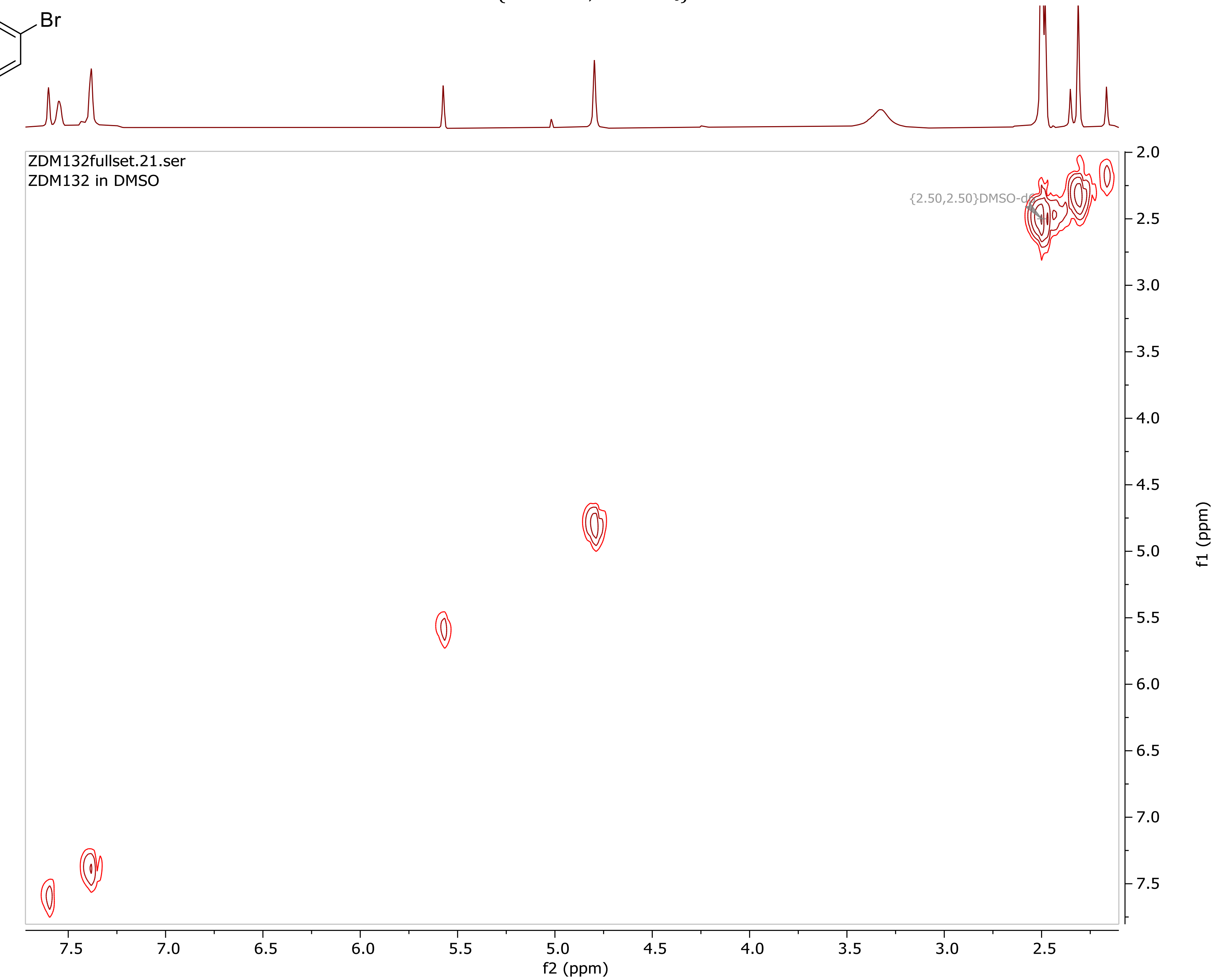

HSQC-DEPT NMR of **34** (500 MHz, DMSO-d_6)

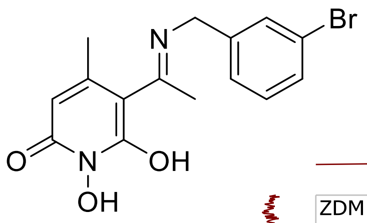

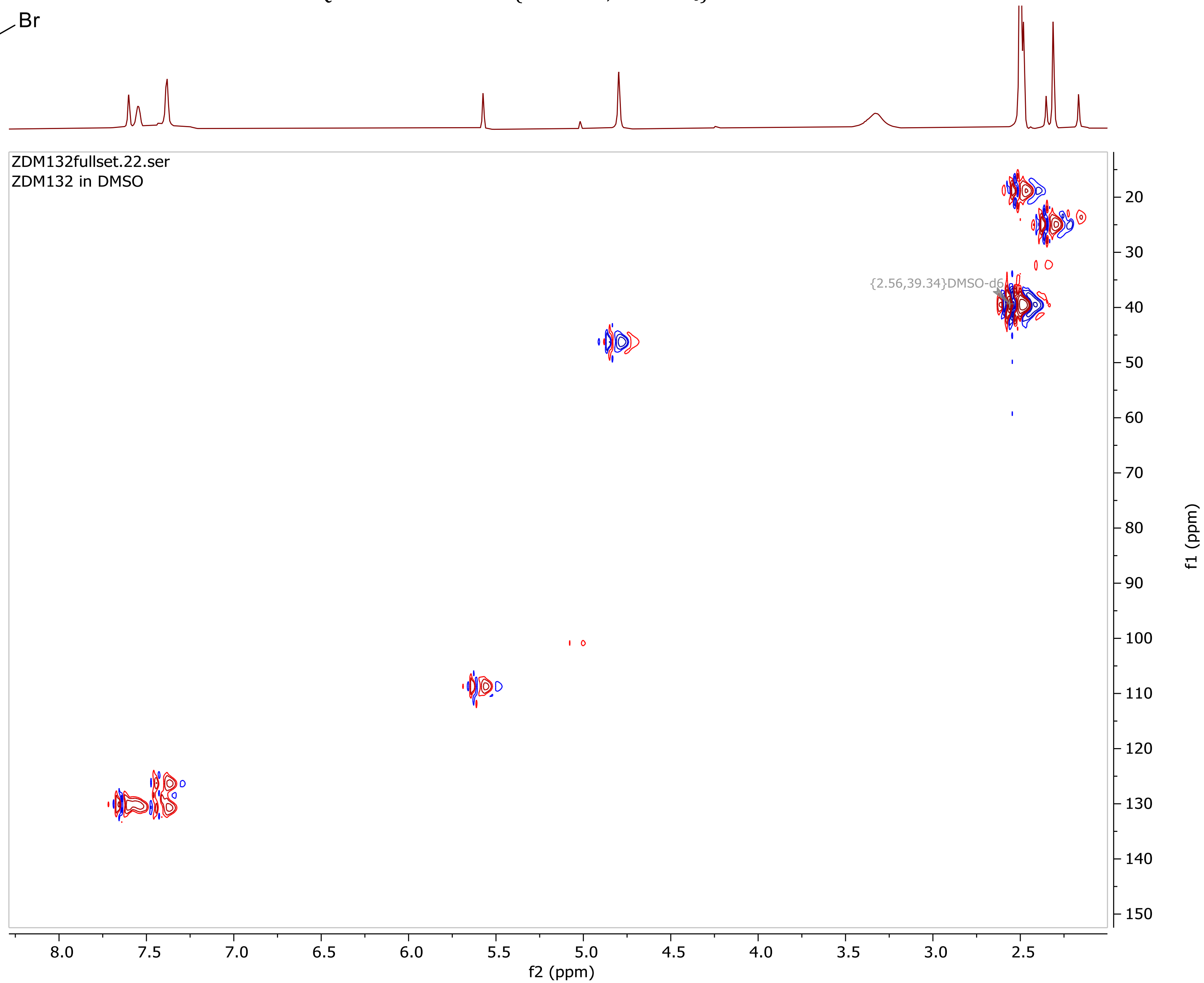

HMBC NMR of **34** (500 MHz, DMSO-d_6)

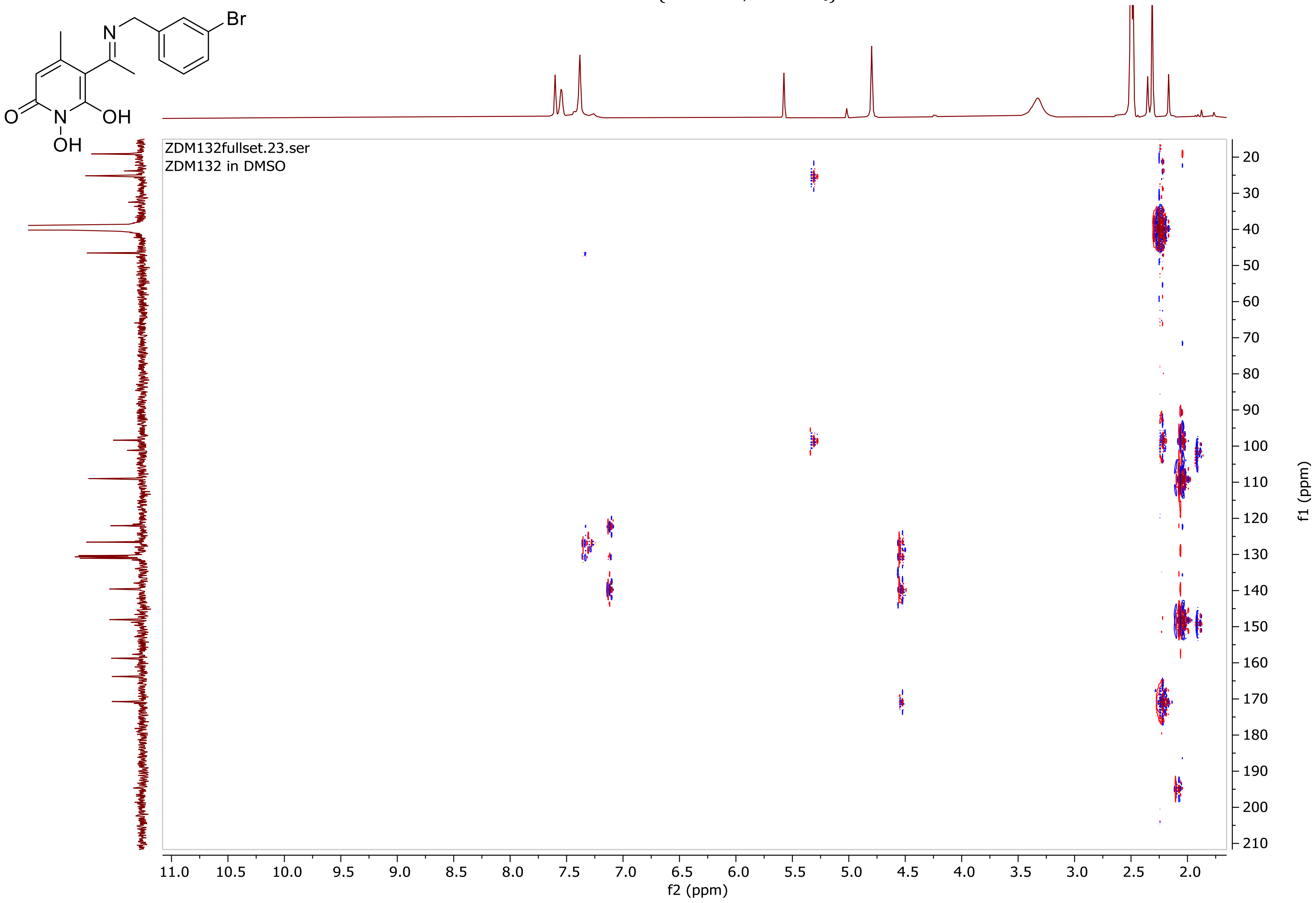

^{1}H NMR of **35** (400 MHz, DMSO-d_6)

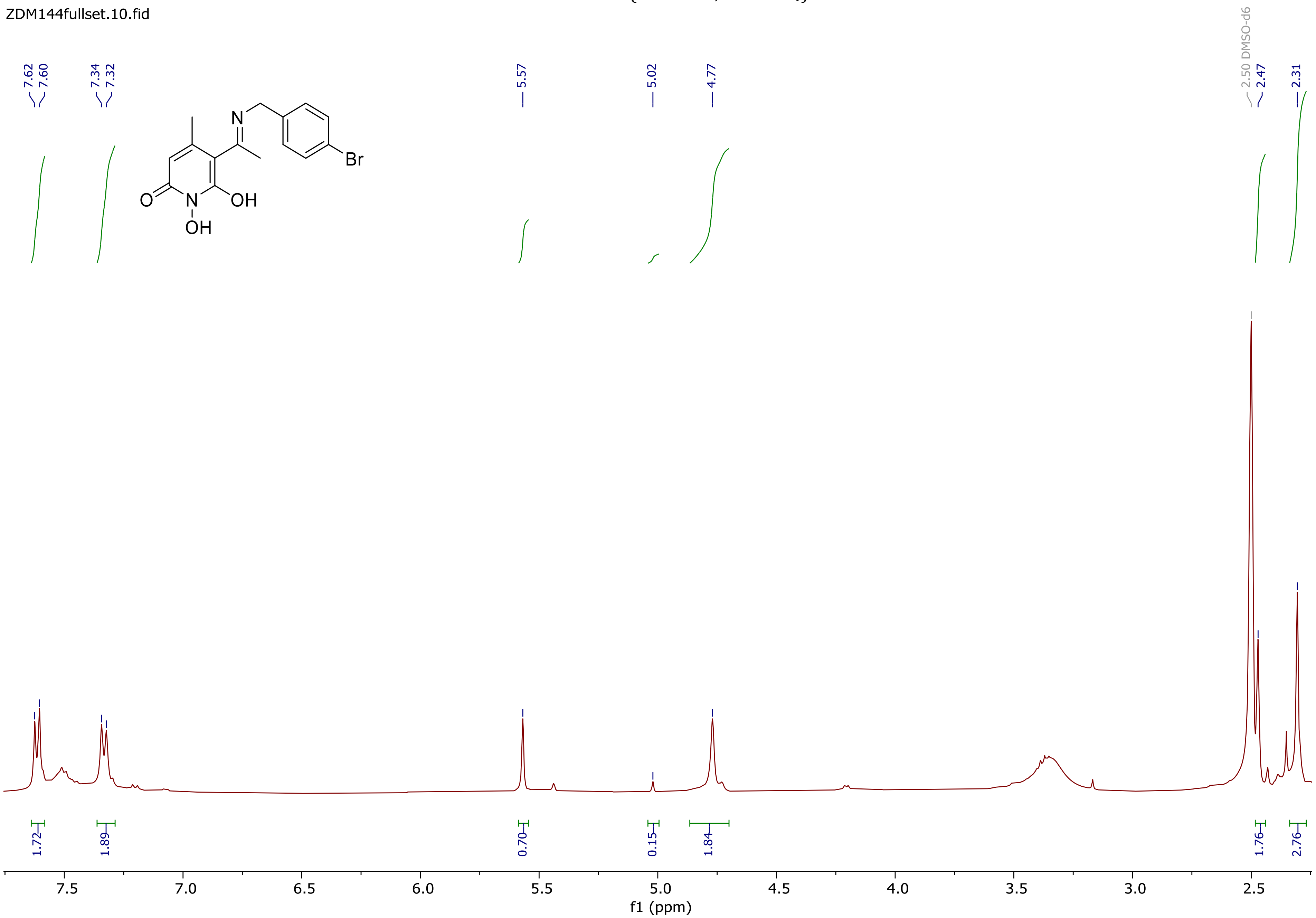

^{13}C NMR of **35** (101 MHz, DMSO-d_6)

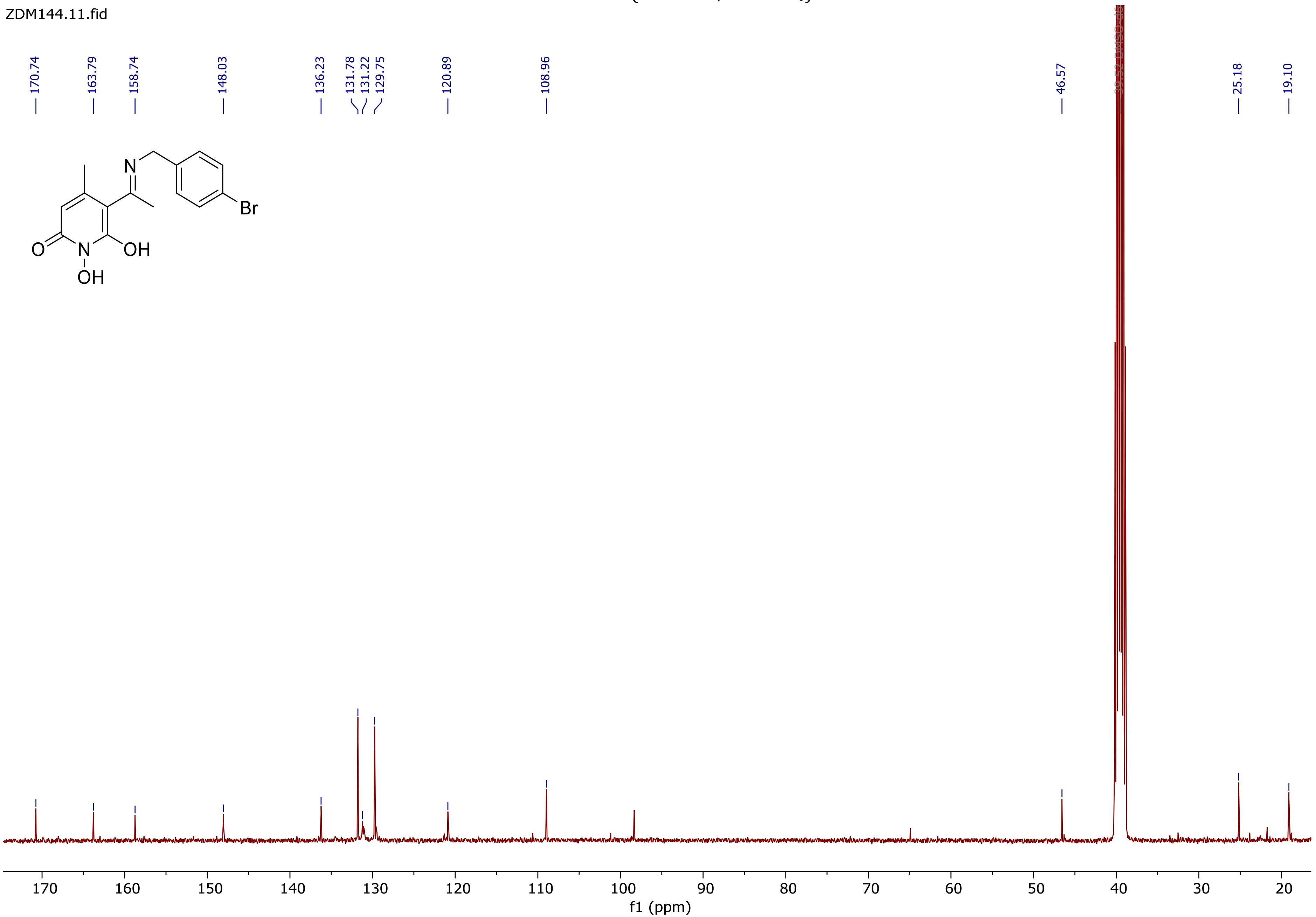

COSY NMR of **35** (400 MHz, DMSO-d_6)

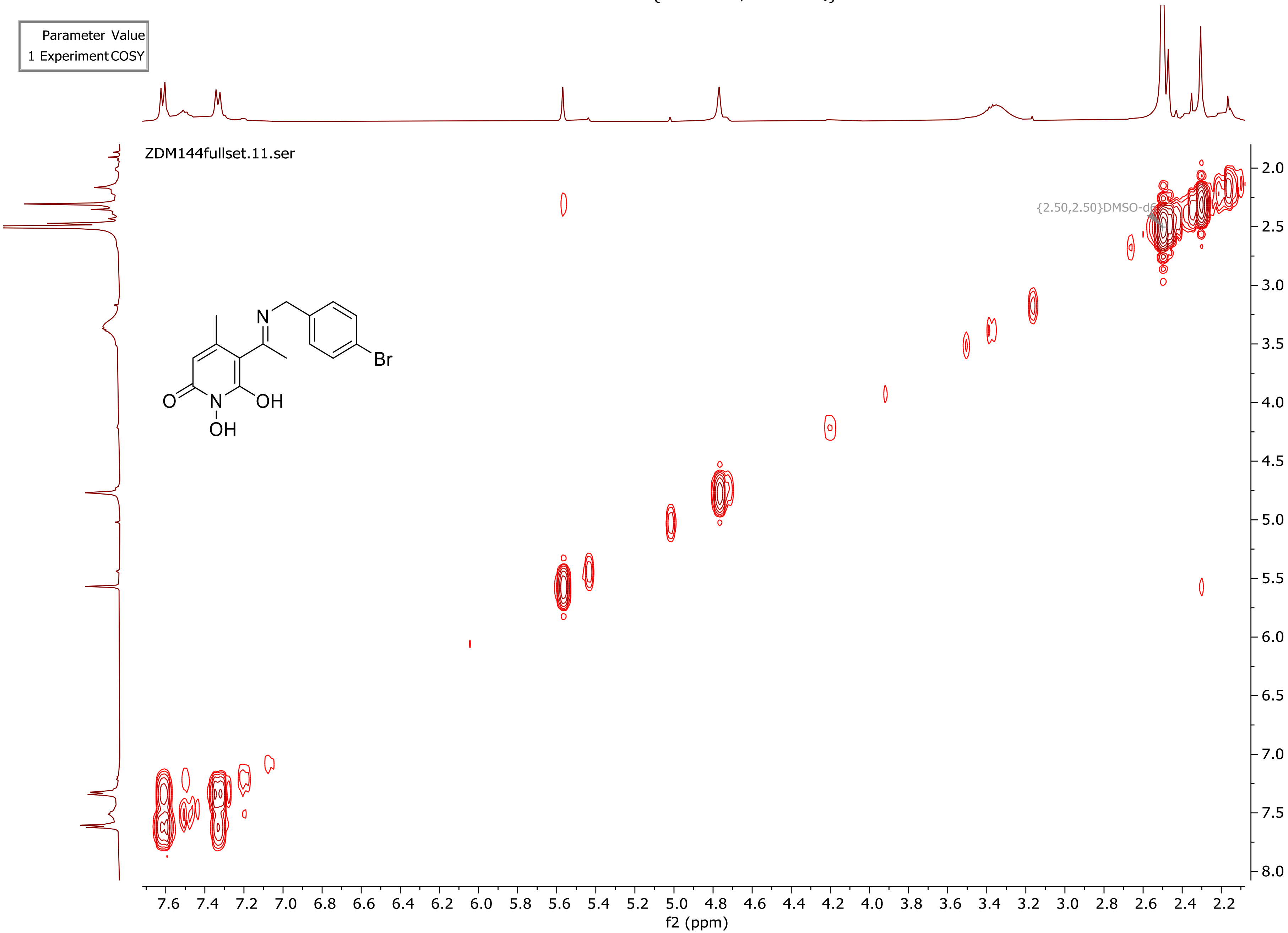

HSQC-DEPT NMR of **35** (400 MHz, DMSO-d_6)

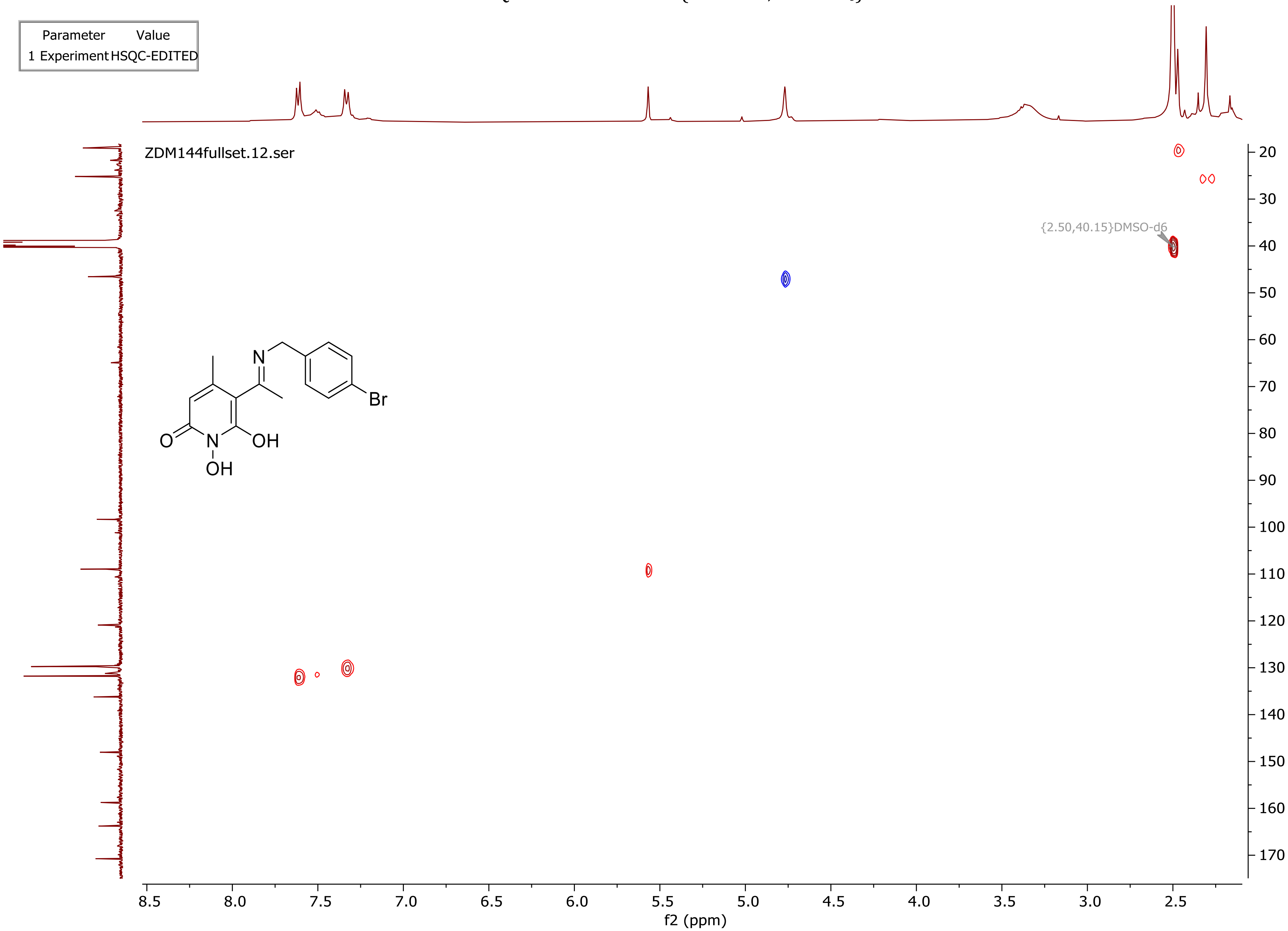

HMBC NMR of **35** (400 MHz, DMSO-d_6)

	Parameter	Value
1	Experiment	HMBC

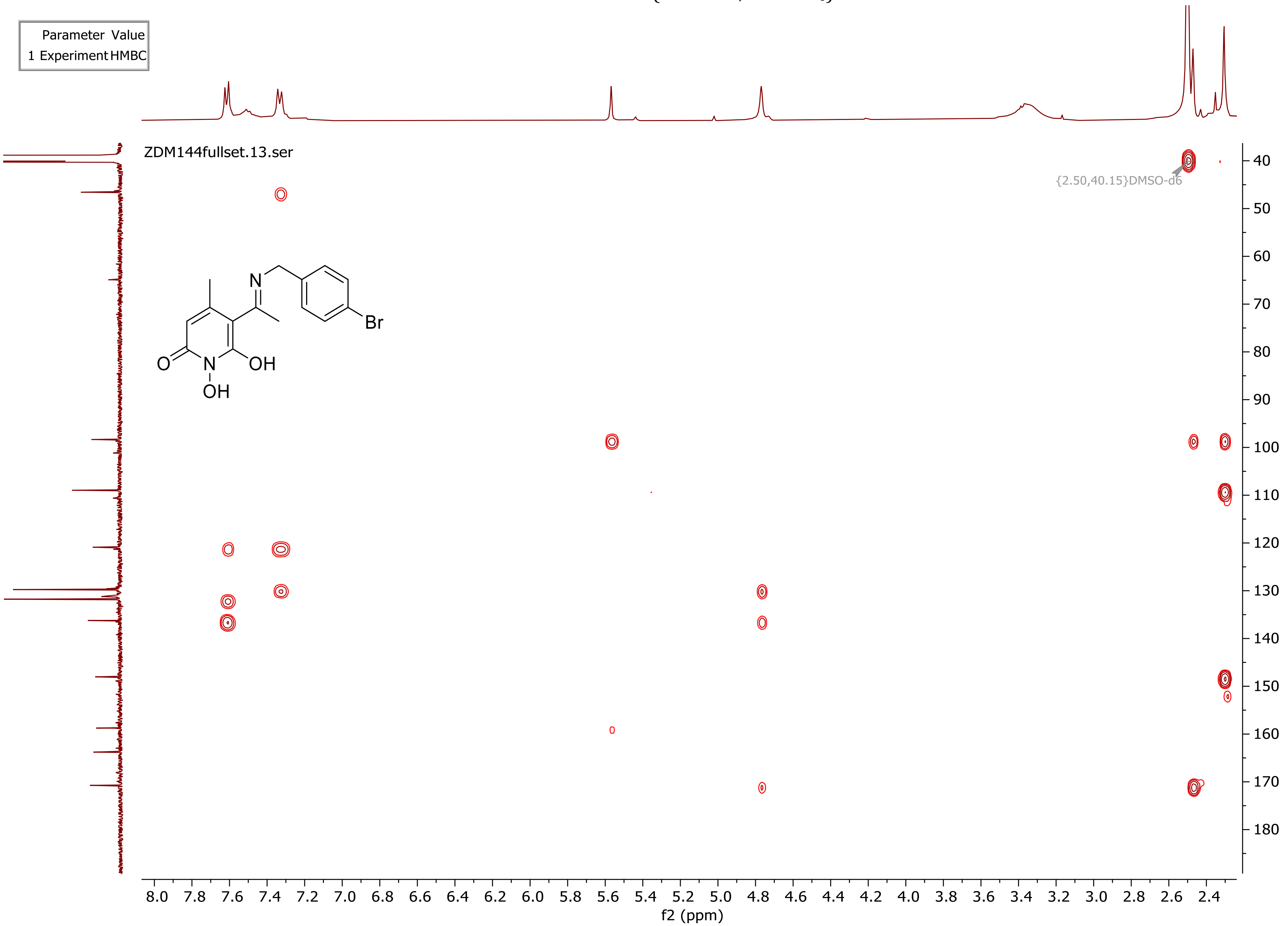

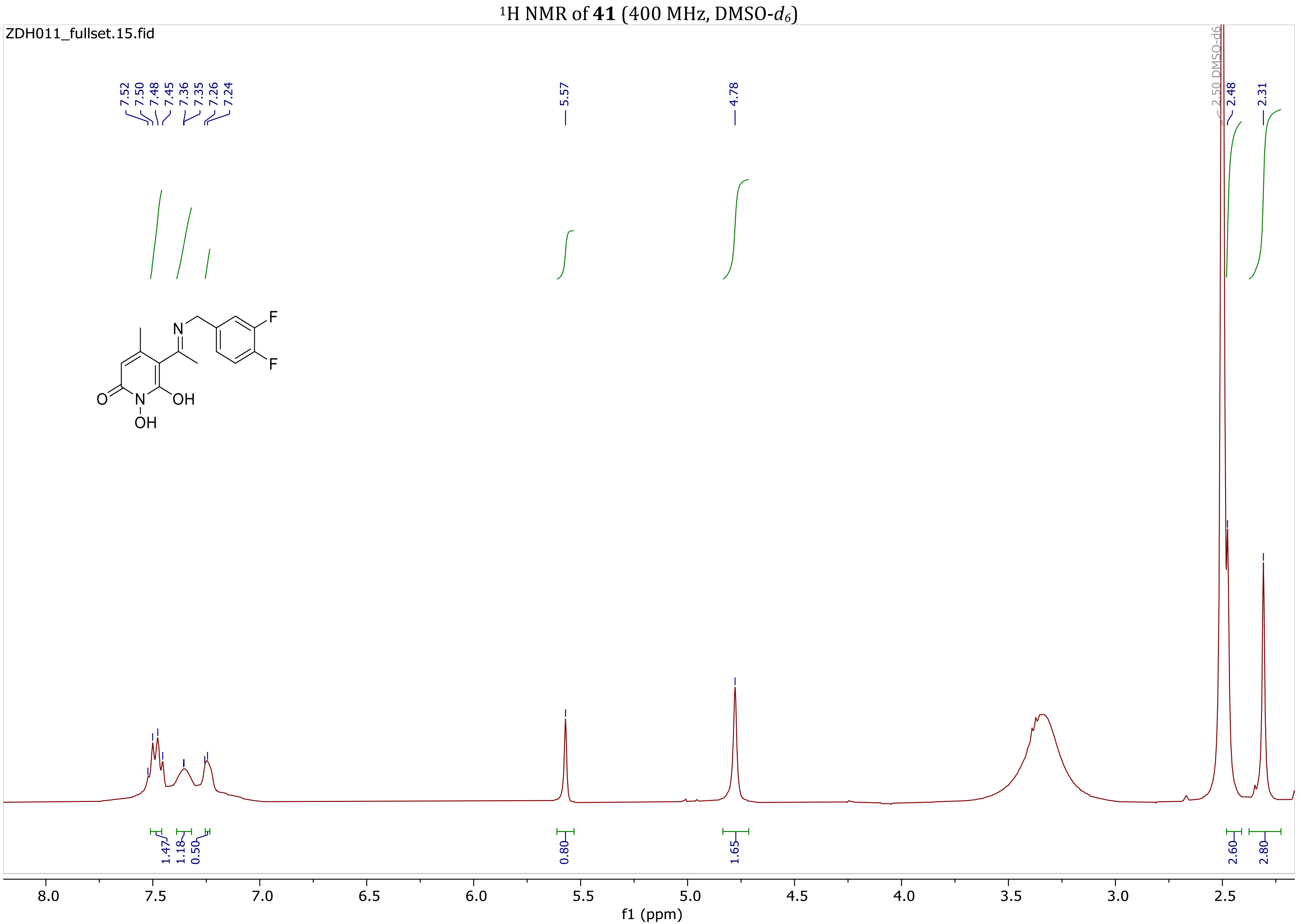

¹H NMR of 41 (400 MHz, DMSO-d6)
ZDH011_fullset.15.fid
7.52
7.50
7.48
7.45
7.36
7.35
7.26
7.24
5.57
4.78
2.50 DMSO-d6
2.48
2.31
1.47
1.18
0.50
0.80
1.65
2.60
2.80
F
F
N
O
N
OH
OH
8.0
7.5
7.0
6.5
6.0
5.5
5.0
4.5
4.0
3.5
3.0
2.5
f1 (ppm)

^{13}C NMR of **41** (101 MHz, DMSO-d_6)

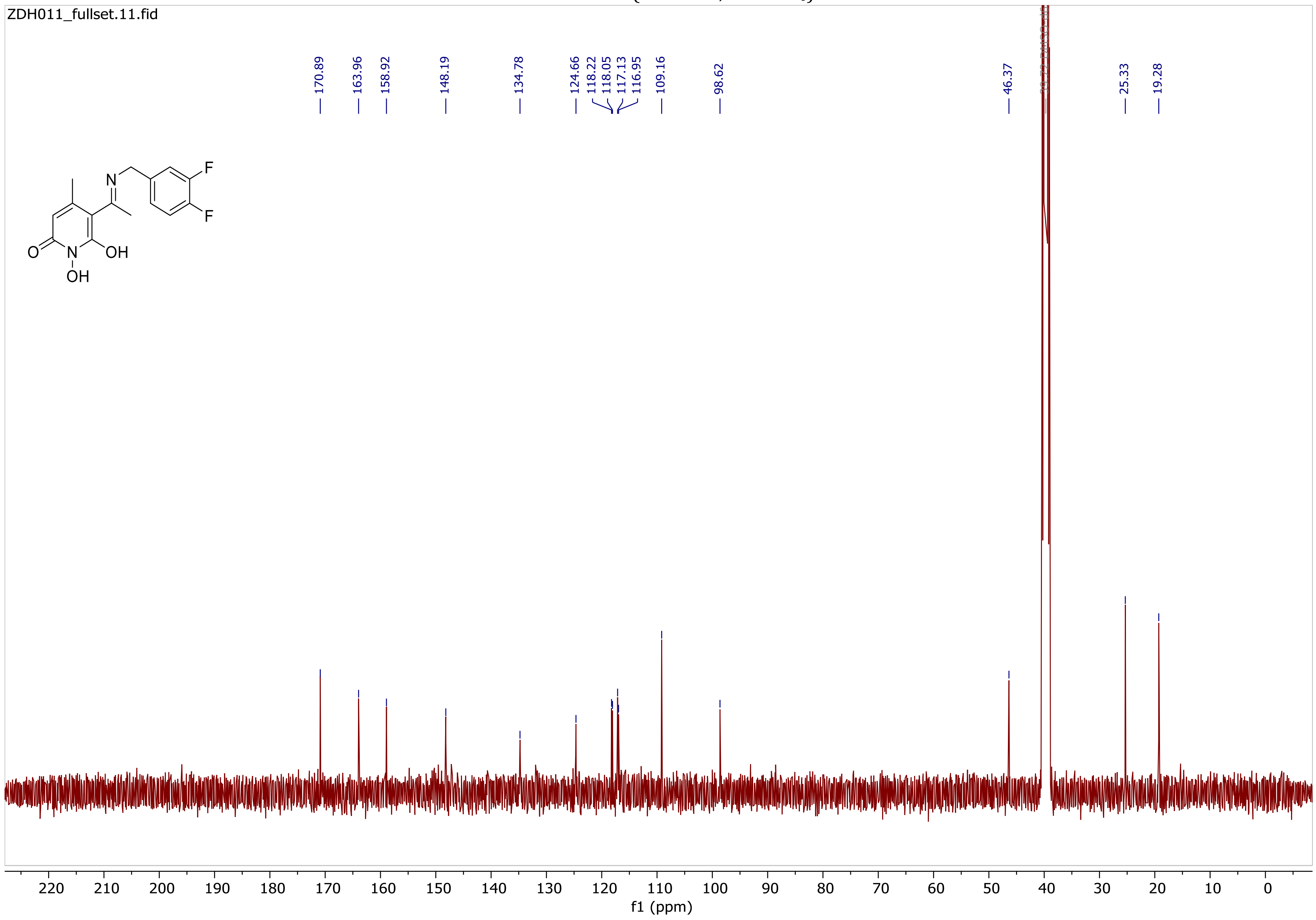

COSY NMR of **41** (400 MHz, DMSO-d_6)

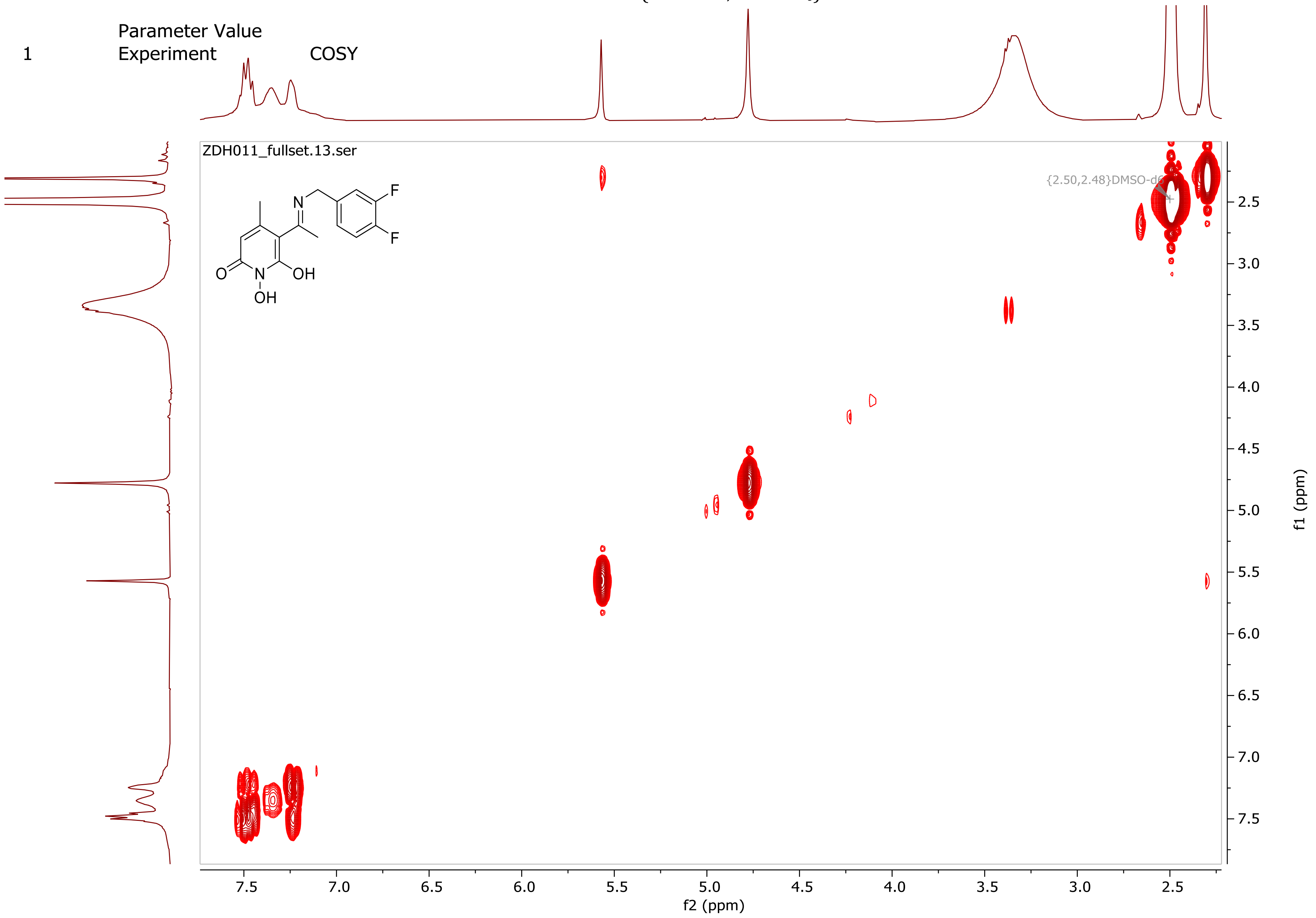

HSQC-DEPT NMR of **41** (400 MHz, DMSO-d_6)

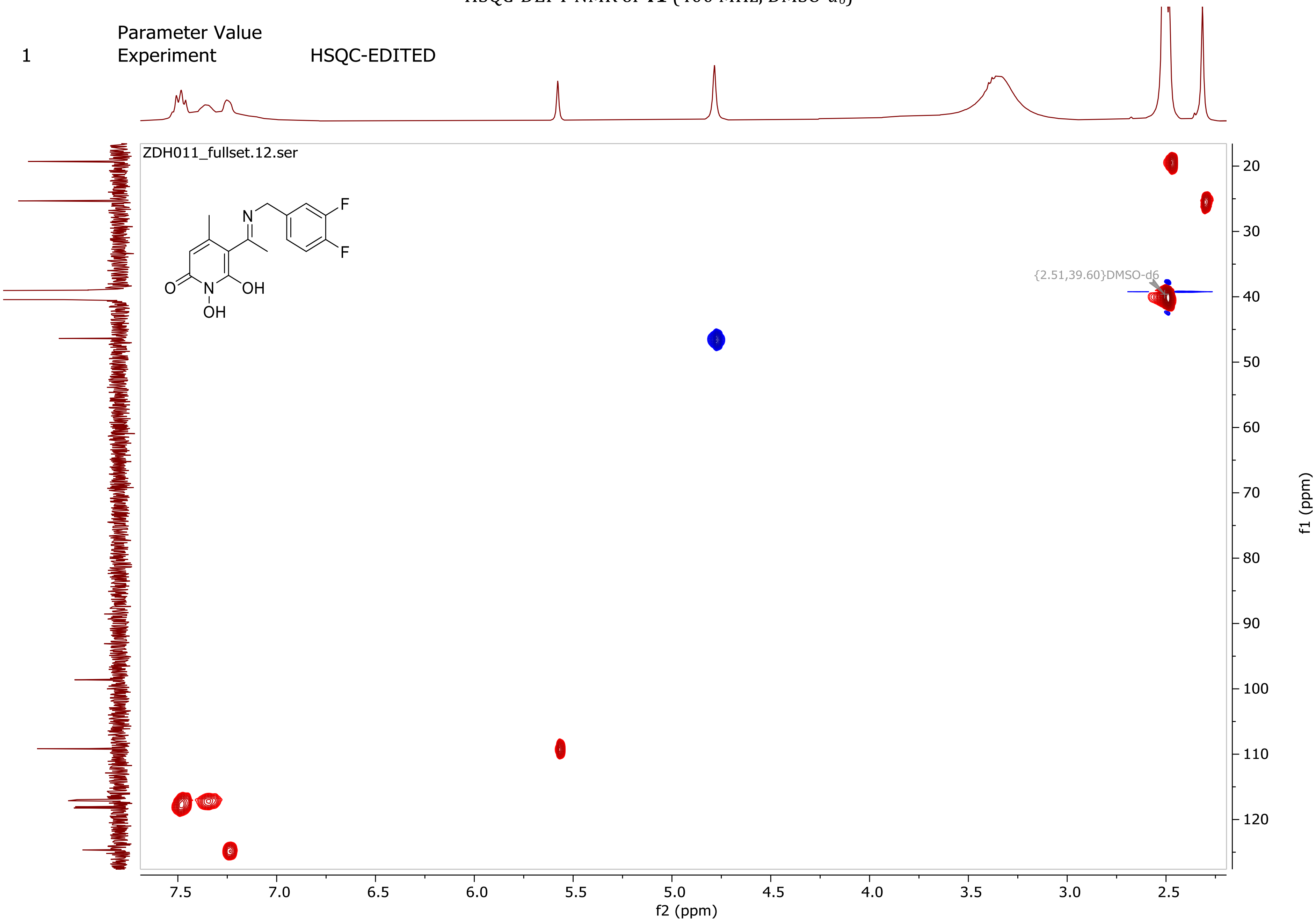

HMBC NMR of **41** (400 MHz, DMSO-d_6)

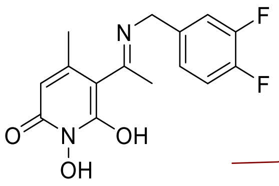

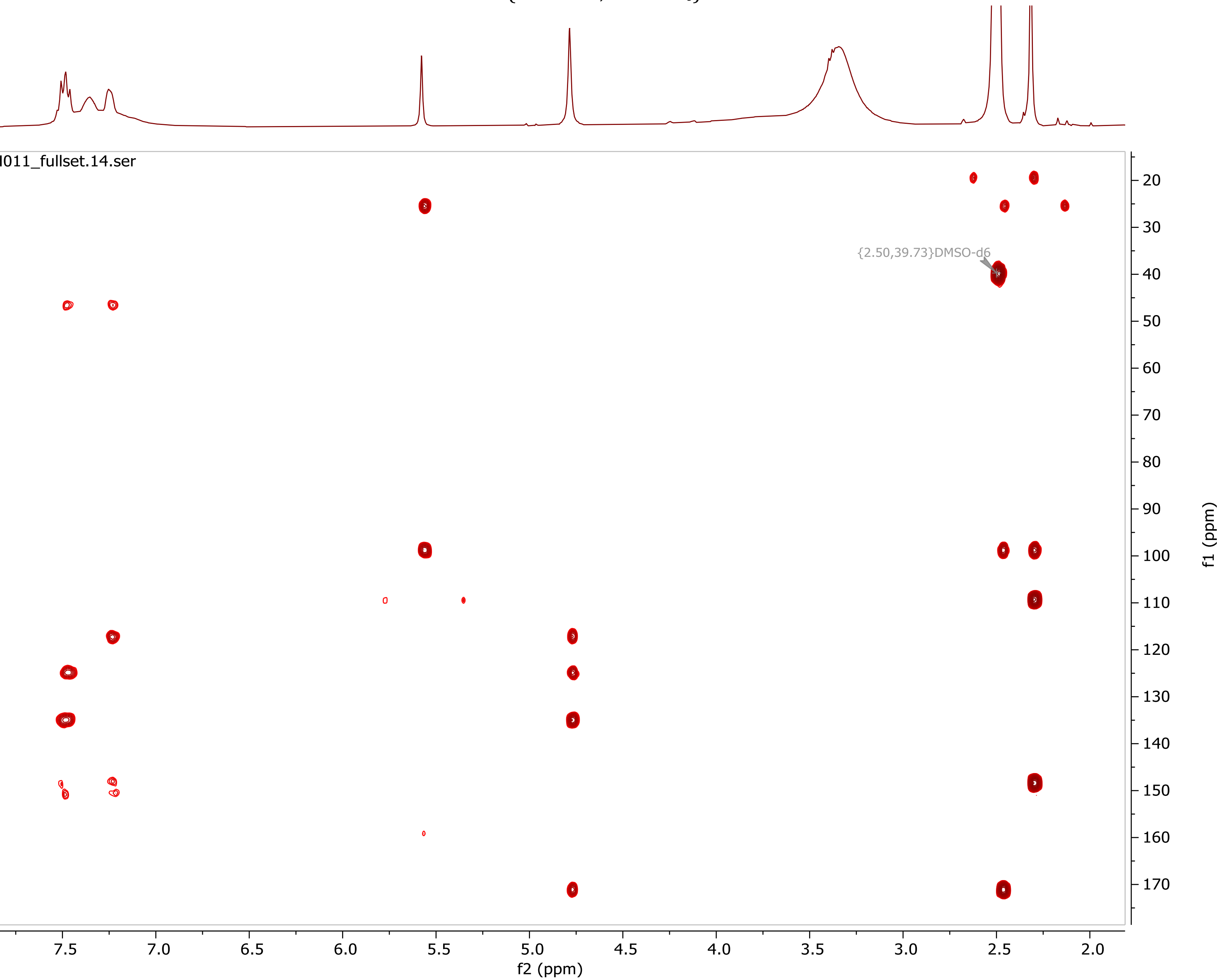

Dose-dependent reduction in RNase H activity

Compound 22

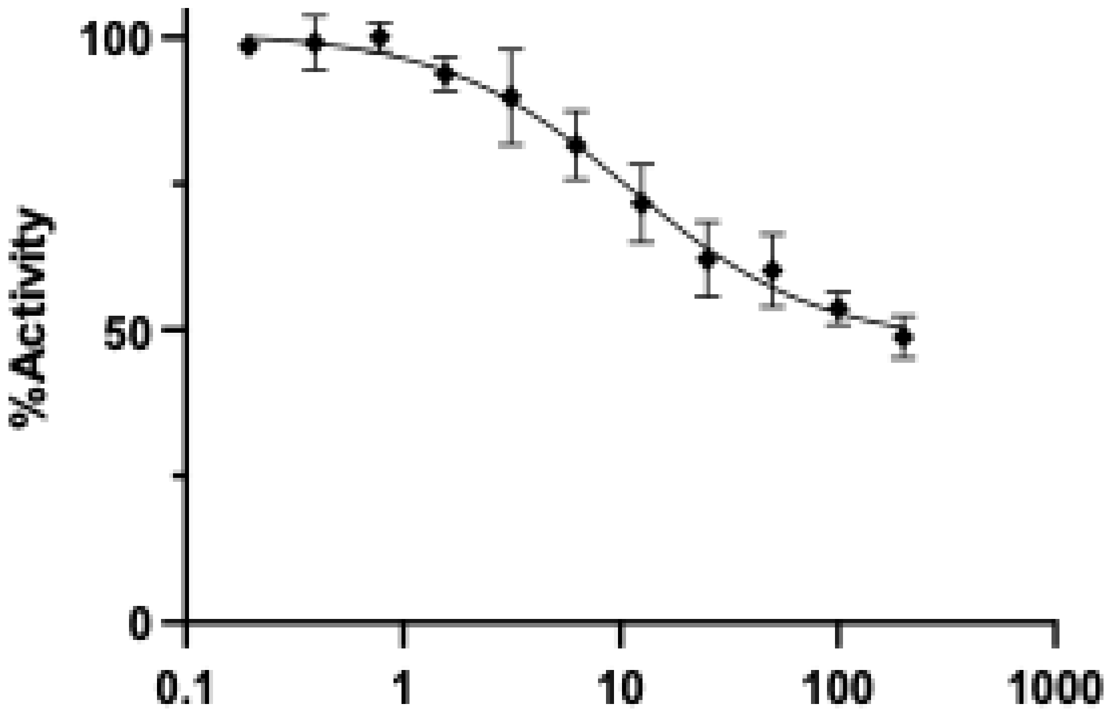

Boltzmann Population Analysis and EC_{50} Modeling

Conformers of minimum energy

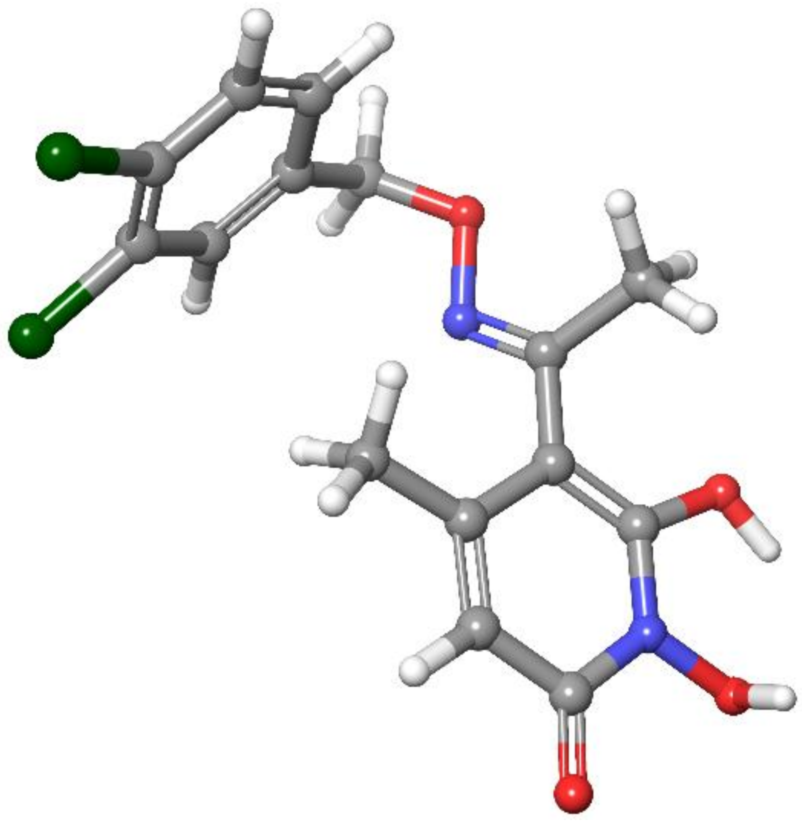

Fig. S1: Umin conformer of 30

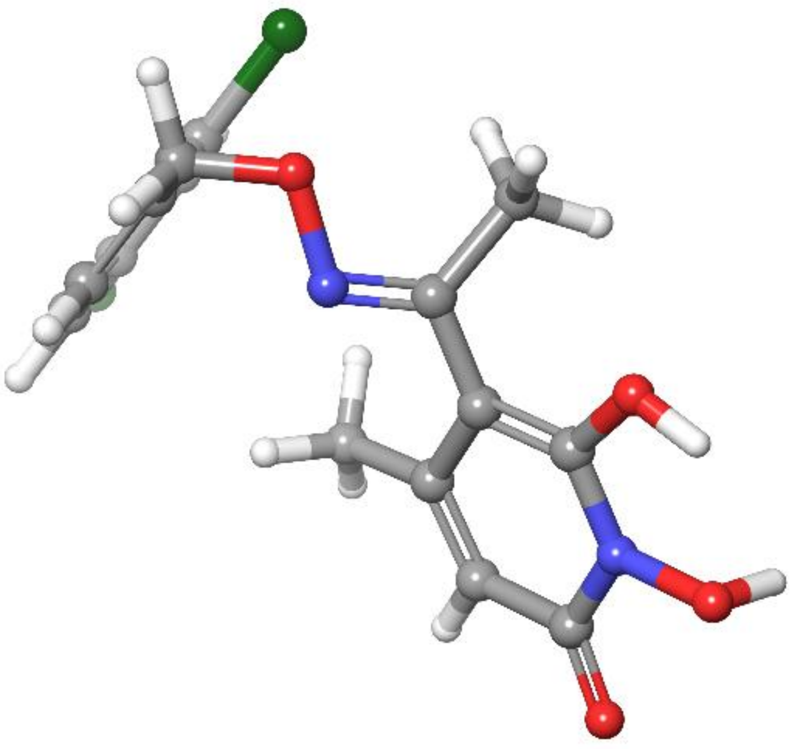

Fig. S2: Umin conformer of 31

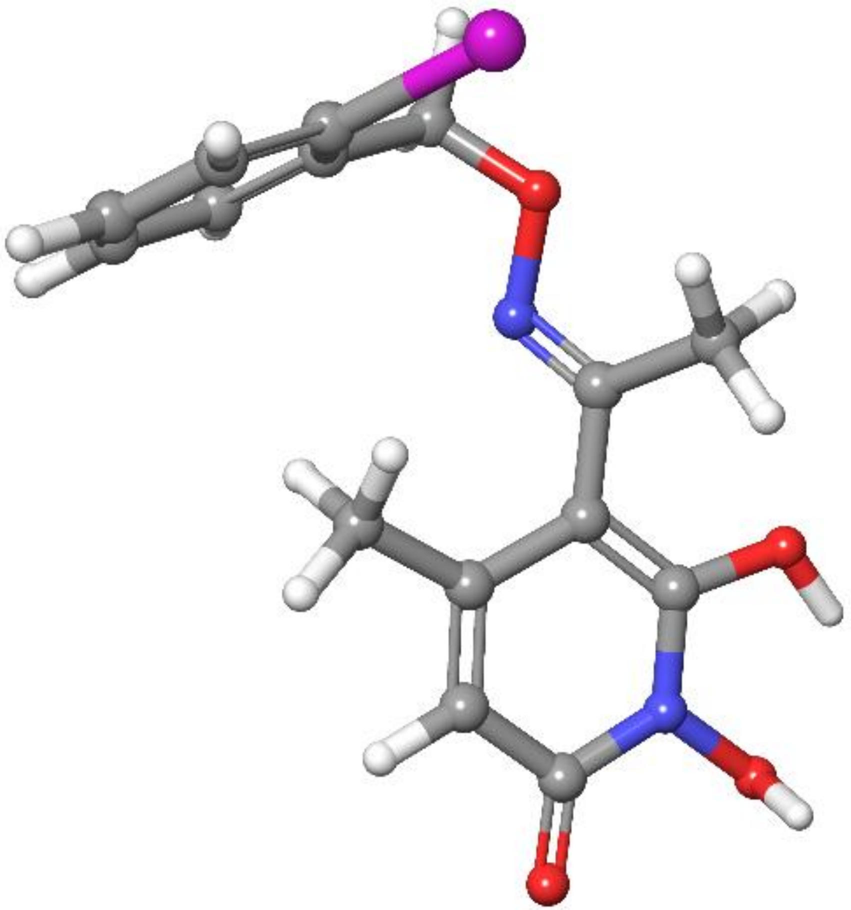

Fig. S3: Umin conformer of 32

Conformational search – Boltzmann population Tables

Table S1: Conformational search of compound 30

Rank	ΔU kJ/mol	RMS	Diastereomer	w_i	p_i	p_i (%)
1	0	0.115	E	1	0.039743	3.974348
2	0.008	0.146	E	0.996776	0.039615	3.961535
3	0.106	0.127	E	0.958118	0.038079	3.807896
4	0.11	0.105	E	0.956573	0.038018	3.801753
5	0.436	0.166	E	0.838636	0.03333	3.33303
6	0.482	0.157	E	0.823209	0.032717	3.271718
7	0.552	0.113	E	0.800276	0.031806	3.180575
8	0.587	0.14	E	0.78905	0.03136	3.135959
9	0.592	0.124	E	0.787459	0.031296	3.129637
10	0.594	0.118	E	0.786824	0.031271	3.127112
11	0.67	0.153	E	0.763054	0.030326	3.032643
12	0.674	0.164	E	0.761823	0.030278	3.027751
13	1.007	0.149	E	0.666013	0.02647	2.646967
14	1.024	0.127	E	0.661459	0.026289	2.628867
15	1.105	0.102	E	0.640183	0.025443	2.54431
16	1.118	0.106	E	0.636833	0.02531	2.530995
17	2.785	0.174	E	0.32495	0.012915	1.291464
18	2.79	0.127	E	0.324295	0.012889	1.28886
19	2.835	0.182	E	0.318458	0.012657	1.265662
20	2.843	0.121	E	0.317431	0.012616	1.261582
21	3.037	0.139	E	0.293524	0.011666	1.166565
22	3.038	0.15	E	0.293405	0.011661	1.166095
23	3.046	0.129	E	0.292459	0.011623	1.162335
24	3.056	0.101	E	0.291281	0.011577	1.157653
25	3.478	0.176	E	0.245663	0.009764	0.976351
26	3.504	0.14	E	0.243099	0.009662	0.966159
27	3.523	0.116	E	0.241242	0.009588	0.958778
28	3.526	0.137	Z	0.24095	0.009576	0.957617
29	3.643	0.121	E	0.229836	0.009134	0.913446
30	3.66	0.114	E	0.228264	0.009072	0.9072

31	3.67	0.173	E	0.227344	0.009035	0.903546
32	3.734	0.158	E	0.221547	0.008805	0.880505
33	3.758	0.174	E	0.219411	0.00872	0.872016
34	3.787	0.137	Z	0.216858	0.008619	0.861869
35	4.316	0.097	Z	0.175165	0.006962	0.696166
36	4.38	0.127	E	0.170698	0.006784	0.678413
37	4.391	0.129	E	0.169942	0.006754	0.675408
38	4.458	0.167	E	0.165408	0.006574	0.657388
39	4.464	0.137	Z	0.165008	0.006558	0.655797
40	4.467	0.141	E	0.164808	0.00655	0.655004
41	4.469	0.11	E	0.164675	0.006545	0.654475
42	4.482	0.167	E	0.163813	0.006511	0.65105
43	4.562	0.092	E	0.158608	0.006304	0.630364
44	4.569	0.168	E	0.158161	0.006286	0.628585
45	4.98	0.117	E	0.133984	0.005325	0.5325
46	4.981	0.168	E	0.13393	0.005323	0.532285
47	5.033	0.101	E	0.131149	0.005212	0.52123
48	5.037	0.099	E	0.130937	0.005204	0.520389
49	5.04	0.143	E	0.130779	0.005198	0.51976
50	5.043	0.142	E	0.13062	0.005191	0.519131
51	5.111	0.142	E	0.127084	0.005051	0.505076
52	5.118	0.135	E	0.126725	0.005037	0.503651
53	5.135	0.134	E	0.125859	0.005002	0.500207
54	5.146	0.114	E	0.125301	0.00498	0.497991
55	5.153	0.11	E	0.124948	0.004966	0.496586
56	5.156	0.087	E	0.124797	0.00496	0.495985
57	5.177	0.125	E	0.123743	0.004918	0.491799
58	5.19	0.168	E	0.123096	0.004892	0.489225
59	5.21	0.162	E	0.122106	0.004853	0.485292
60	5.218	0.131	E	0.121712	0.004837	0.483728
61	5.378	0.173	E	0.114101	0.004535	0.453476
62	5.39	0.13	E	0.113549	0.004513	0.451285
63	5.392	0.12	E	0.113458	0.004509	0.450921
64	5.393	0.14	Z	0.113412	0.004507	0.450739

65	5.403	0.121	E	0.112955	0.004489	0.448923
66	5.42	0.184	E	0.112183	0.004459	0.445853
67	5.424	0.113	Z	0.112002	0.004451	0.445134
68	5.427	0.125	E	0.111866	0.004446	0.444596
69	5.428	0.081	Z	0.111821	0.004444	0.444416
70	5.446	0.153	E	0.111012	0.004412	0.441199
71	5.449	0.109	E	0.110877	0.004407	0.440665
72	5.451	0.156	Z	0.110788	0.004403	0.44031
73	5.472	0.122	Z	0.109853	0.004366	0.436593
74	5.487	0.1	Z	0.10919	0.00434	0.433958
75	5.499	0.131	Z	0.108662	0.004319	0.431861
76	5.527	0.145	Z	0.107441	0.00427	0.427008
77	5.529	0.125	E	0.107354	0.004267	0.426663
78	6.402	0.175	Z	0.075473	0.003	0.299956
79	6.798	0.142	Z	0.064324	0.002556	0.255648
80	7.026	0.102	Z	0.058669	0.002332	0.233172
81	7.421	0.088	Z	0.050023	0.001988	0.198809
82	7.853	0.152	Z	0.042019	0.00167	0.166998
83	7.867	0.127	Z	0.041782	0.001661	0.166057
84	7.908	0.173	Z	0.041096	0.001633	0.163331
85	7.919	0.172	Z	0.040914	0.001626	0.162608
86	7.937	0.178	Z	0.040618	0.001614	0.161431
87	7.944	0.134	Z	0.040504	0.00161	0.160975
88	7.958	0.142	Z	0.040275	0.001601	0.160068
89	8.006	0.111	Z	0.039503	0.00157	0.156997
90	8.06	0.148	Z	0.038651	0.001536	0.153612
91	8.163	0.147	Z	0.037077	0.001474	0.147357
92	8.184	0.082	Z	0.036764	0.001461	0.146113
93	8.187	0.095	Z	0.03672	0.001459	0.145936
94	8.245	0.137	Z	0.03587	0.001426	0.14256
95	8.247	0.193	Z	0.035841	0.001424	0.142445
96	8.299	0.123	Z	0.035097	0.001395	0.139486
97	8.302	0.096	Z	0.035054	0.001393	0.139317
98	8.33	0.121	Z	0.03466	0.001378	0.137752

99	8.334	0.135	Z	0.034604	0.001375	0.13753
100	8.336	0.174	Z	0.034576	0.001374	0.137419
101	8.439	0.144	Z	0.033168	0.001318	0.131823
102	8.64	0.132	Z	0.030584	0.001216	0.121551
103	8.684	0.129	Z	0.030045	0.001194	0.119411
104	8.687	0.138	Z	0.030009	0.001193	0.119267
105	8.728	0.134	Z	0.029517	0.001173	0.117309
106	8.739	0.102	Z	0.029386	0.001168	0.116789
107	8.742	0.141	Z	0.02935	0.001166	0.116648
108	8.757	0.116	Z	0.029173	0.001159	0.115944
109	8.758	0.141	Z	0.029161	0.001159	0.115897
110	8.845	0.112	Z	0.028155	0.001119	0.111898
111	8.851	0.14	Z	0.028087	0.001116	0.111627
112	8.92	0.198	Z	0.027316	0.001086	0.108562
113	9.006	0.133	Z	0.026384	0.001049	0.104858
114	10.338	0.102	Z	0.015412	0.000613	0.061251
115	10.426	0.096	Z	0.014874	0.000591	0.059114
116	10.439	0.185	E	0.014796	0.000588	0.058804
117	10.442	0.097	E	0.014778	0.000587	0.058733
118	10.457	0.111	E	0.014689	0.000584	0.058379
119	10.462	0.146	E	0.014659	0.000583	0.058261
120	10.504	0.114	Z	0.014413	0.000573	0.057282
121	10.535	0.12	Z	0.014234	0.000566	0.056569
122	10.564	0.145	E	0.014068	0.000559	0.055911
123	10.564	0.145	E	0.014068	0.000559	0.055911
124	10.573	0.117	E	0.014017	0.000557	0.055708
125	10.574	0.124	E	0.014011	0.000557	0.055686
126	10.782	0.159	Z	0.012883	0.000512	0.051202
127	10.879	0.162	Z	0.012388	0.000492	0.049236
128	10.912	0.159	Z	0.012225	0.000486	0.048584
129	10.933	0.134	Z	0.012121	0.000482	0.048174
130	11.023	0.102	E	0.011689	0.000465	0.046456
131	11.025	0.129	E	0.011679	0.000464	0.046418
132	11.039	0.087	E	0.011614	0.000462	0.046157

133	11.044	0.139	E	0.01159	0.000461	0.046064
134	11.054	0.128	Z	0.011544	0.000459	0.045878
135	11.065	0.159	Z	0.011492	0.000457	0.045675
136	11.116	0.136	E	0.011258	0.000447	0.044744
137	11.12	0.139	Z	0.01124	0.000447	0.044672
138	11.121	0.109	E	0.011236	0.000447	0.044654
139	11.126	0.093	E	0.011213	0.000446	0.044564
140	11.133	0.149	E	0.011181	0.000444	0.044438
141	11.151	0.162	Z	0.0111	0.000441	0.044117
142	11.327	0.121	E	0.010339	0.000411	0.041091
143	11.332	0.145	E	0.010318	0.00041	0.041009
144	11.335	0.14	E	0.010306	0.00041	0.040959
145	11.336	0.127	E	0.010302	0.000409	0.040943
146	11.397	0.12	E	0.010051	0.000399	0.039947
147	11.401	0.113	E	0.010035	0.000399	0.039882
148	11.403	0.131	E	0.010027	0.000399	0.03985
149	11.405	0.103	E	0.010019	0.000398	0.039818
150	11.583	0.126	E	0.009324	0.000371	0.037058
151	11.585	0.131	E	0.009317	0.00037	0.037028
152	11.586	0.16	E	0.009313	0.00037	0.037013
153	11.587	0.118	E	0.009309	0.00037	0.036998
154	11.62	0.118	E	0.009186	0.000365	0.036508
155	11.62	0.124	E	0.009186	0.000365	0.036508
156	11.62	0.118	E	0.009186	0.000365	0.036508
157	11.621	0.198	E	0.009182	0.000365	0.036494
158	13.892	0.168	Z	0.003672	0.000146	0.014593
159	14.007	0.134	Z	0.003505	0.000139	0.013931
160	14.214	0.142	Z	0.003224	0.000128	0.012814
161	14.309	0.16	Z	0.003103	0.000123	0.012332
162	14.442	0.115	Z	0.002941	0.000117	0.011688
163	14.455	0.135	Z	0.002925	0.000116	0.011626
164	14.48	0.152	Z	0.002896	0.000115	0.01151
165	14.576	0.147	Z	0.002786	0.000111	0.011072
166	14.61	0.09	Z	0.002748	0.000109	0.010921

167	14.617	0.166	Z	0.00274	0.000109	0.01089
168	14.635	0.188	E	0.00272	0.000108	0.010812
169	14.643	0.145	Z	0.002712	0.000108	0.010777
170	14.646	0.108	Z	0.002708	0.000108	0.010764
171	14.77	0.131	Z	0.002576	0.000102	0.010238
172	14.783	0.139	Z	0.002563	0.000102	0.010185
173	14.797	0.152	Z	0.002548	0.000101	0.010127
174	14.882	0.086	Z	0.002462	9.79E-05	0.009786
175	15.08	0.134	E	0.002273	9.03E-05	0.009034
176	15.153	0.191	E	0.002207	8.77E-05	0.008772
177	15.166	0.132	Z	0.002196	8.73E-05	0.008726
178	15.174	0.167	Z	0.002188	8.7E-05	0.008698
179	15.244	0.15	E	0.002128	8.46E-05	0.008456
180	15.264	0.179	Z	0.00211	8.39E-05	0.008388
181	15.278	0.101	Z	0.002099	8.34E-05	0.00834
182	15.318	0.147	Z	0.002065	8.21E-05	0.008207
183	15.318	0.145	Z	0.002065	8.21E-05	0.008207
184	15.32	0.063	E	0.002063	8.2E-05	0.0082
185	15.387	0.136	Z	0.002008	7.98E-05	0.007981
186	15.397	0.125	Z	0.002	7.95E-05	0.007949
187	17.309	0.119	Z	0.000924	3.67E-05	0.003674

Table S2: Conformational search of compound 31

Rank	ΔU kJ/mol	RMS	Diasteromer	w_i	p_i	p_i (%)
1	0	0.143	E	1	0.047143	4.714284
2	0.001	0.132	E	0.999596	0.047124	4.712382
3	0.119	0.075	E	0.953104	0.044932	4.493205
4	0.123	0.142	E	0.951567	0.04486	4.485957
5	0.553	0.107	E	0.799953	0.037712	3.771206
6	0.556	0.162	E	0.798985	0.037666	3.766642
7	0.645	0.176	E	0.770793	0.036337	3.633737
8	0.653	0.129	E	0.768308	0.03622	3.622023
9	1.924	0.112	E	0.459983	0.021685	2.168489
10	1.93	0.121	E	0.45887	0.021632	2.163243
11	2.023	0.187	E	0.441965	0.020835	2.083547
12	2.023	0.116	E	0.441965	0.020835	2.083547
13	2.506	0.142	E	0.363682	0.017145	1.714503
14	2.513	0.111	E	0.362656	0.017097	1.709665
15	2.578	0.126	E	0.353266	0.016654	1.665395
16	2.581	0.153	E	0.352838	0.016634	1.66338
17	3.062	0.139	E	0.290577	0.013699	1.369862
18	3.062	0.125	E	0.290577	0.013699	1.369862
19	3.063	0.145	E	0.29046	0.013693	1.369309
20	3.076	0.116	E	0.288939	0.013621	1.362143
21	3.241	0.158	E	0.270324	0.012744	1.274382
22	3.249	0.084	E	0.269452	0.012703	1.270274
23	3.271	0.205	E	0.26707	0.01259	1.259044
24	3.282	0.103	E	0.265887	0.012535	1.253467
25	3.715	0.129	Z	0.223252	0.010525	1.052476
26	3.718	0.128	Z	0.222982	0.010512	1.051202
27	4.035	0.139	Z	0.196202	0.00925	0.924952
28	4.08	0.171	Z	0.192671	0.009083	0.908304
29	4.128	0.093	Z	0.188974	0.008909	0.890876
30	4.145	0.172	Z	0.187682	0.008848	0.884784
31	4.175	0.131	E	0.185423	0.008741	0.874135

32	4.177	0.176	E	0.185273	0.008734	0.87343
33	4.179	0.092	E	0.185124	0.008727	0.872725
34	4.185	0.08	E	0.184676	0.008706	0.870614
35	4.292	0.15	Z	0.17687	0.008338	0.833815
36	4.293	0.159	E	0.176798	0.008335	0.833478
37	4.299	0.168	E	0.176371	0.008315	0.831462
38	4.3	0.106	Z	0.1763	0.008311	0.831127
39	4.304	0.131	Z	0.176015	0.008298	0.829786
40	4.321	0.136	Z	0.174812	0.008241	0.824112
41	4.412	0.127	E	0.168507	0.007944	0.794392
42	4.417	0.148	E	0.168168	0.007928	0.79279
43	4.423	0.132	E	0.167761	0.007909	0.790873
44	4.425	0.112	E	0.167626	0.007902	0.790234
45	4.441	0.138	Z	0.166547	0.007851	0.785148
46	4.458	0.124	E	0.165408	0.007798	0.779779
47	4.464	0.134	E	0.165008	0.007779	0.777893
48	4.642	0.139	Z	0.153569	0.00724	0.723966
49	4.689	0.121	E	0.150683	0.007104	0.710361
50	4.693	0.14	E	0.15044	0.007092	0.709215
51	4.723	0.122	E	0.148629	0.007007	0.70068
52	4.731	0.096	E	0.14815	0.006984	0.698421
53	4.831	0.206	E	0.142289	0.006708	0.670792
54	4.835	0.197	E	0.14206	0.006697	0.66971
55	4.919	0.178	E	0.137324	0.006474	0.647385
56	4.921	0.159	E	0.137213	0.006469	0.646862
57	4.925	0.144	E	0.136992	0.006458	0.645819
58	4.932	0.147	E	0.136605	0.00644	0.643997
59	4.939	0.112	E	0.13622	0.006422	0.64218
60	4.941	0.138	E	0.13611	0.006417	0.641662
61	5.011	0.157	E	0.132318	0.006238	0.623786
62	5.013	0.164	E	0.132212	0.006233	0.623283
63	5.115	0.101	E	0.126879	0.005981	0.598144
64	5.118	0.13	E	0.126725	0.005974	0.59742
65	5.612	0.155	E	0.103817	0.004894	0.489425

66	5.612	0.11	E	0.103817	0.004894	0.489425
67	5.687	0.152	E	0.100722	0.004748	0.474831
68	5.689	0.119	E	0.100641	0.004744	0.474448
69	6.328	0.166	E	0.077761	0.003666	0.366588
70	6.331	0.12	E	0.077667	0.003661	0.366145
71	6.332	0.167	E	0.077636	0.00366	0.365997
72	6.334	0.168	E	0.077573	0.003657	0.365701
73	6.587	0.224	E	0.070043	0.003302	0.330201
74	6.588	0.147	E	0.070014	0.003301	0.330068
75	6.619	0.155	E	0.069144	0.00326	0.325963
76	6.622	0.131	E	0.06906	0.003256	0.325569
77	7.379	0.172	Z	0.050878	0.002399	0.239855
78	7.606	0.168	Z	0.046424	0.002189	0.218855
79	7.608	0.153	Z	0.046386	0.002187	0.218679
80	7.759	0.173	Z	0.043644	0.002057	0.205749
81	7.798	0.175	Z	0.042962	0.002025	0.202536
82	7.833	0.105	Z	0.042359	0.001997	0.199695
83	8.185	0.128	Z	0.036749	0.001732	0.173246
84	8.339	0.15	Z	0.034535	0.001628	0.162806
85	8.718	0.078	Z	0.029636	0.001397	0.139712
86	8.791	0.099	Z	0.028775	0.001357	0.135656
87	8.909	0.086	Z	0.027437	0.001293	0.129346
88	9.362	0.131	Z	0.022852	0.001077	0.107733
89	9.464	0.116	Z	0.021931	0.001034	0.103387
90	9.597	0.138	Z	0.020784	0.00098	0.097984
91	9.662	0.132	Z	0.020246	0.000954	0.095447
92	9.676	0.156	Z	0.020132	0.000949	0.094909
93	9.737	0.135	Z	0.019643	0.000926	0.092601
94	10.242	0.115	Z	0.016021	0.000755	0.075525
95	10.327	0.122	E	0.01548	0.00073	0.072978
96	10.331	0.1	E	0.015455	0.000729	0.07286
97	10.336	0.1	Z	0.015424	0.000727	0.072713
98	10.428	0.21	Z	0.014862	0.000701	0.070063
99	10.449	0.124	Z	0.014736	0.000695	0.069472

100	10.45	0.15	E	0.01473	0.000694	0.069443
101	10.45	0.074	E	0.01473	0.000694	0.069443
102	10.495	0.166	Z	0.014465	0.000682	0.068194
103	10.545	0.13	Z	0.014176	0.000668	0.066831
104	10.763	0.125	Z	0.012982	0.000612	0.061202
105	10.816	0.118	E	0.012707	0.000599	0.059907
106	10.817	0.127	E	0.012702	0.000599	0.059883
107	10.847	0.133	Z	0.012549	0.000592	0.059162
108	10.895	0.107	E	0.012309	0.00058	0.058027
109	10.9	0.154	E	0.012284	0.000579	0.05791
110	10.919	0.069	E	0.01219	0.000575	0.057467
111	10.925	0.096	E	0.012161	0.000573	0.057328
112	10.99	0.116	E	0.011846	0.000558	0.055844
113	10.998	0.157	E	0.011807	0.000557	0.055664
114	11.353	0.096	E	0.010231	0.000482	0.048233
115	11.356	0.111	E	0.010219	0.000482	0.048175
116	11.428	0.122	E	0.009926	0.000468	0.046795
117	11.429	0.159	E	0.009922	0.000468	0.046776
118	11.483	0.076	E	0.009708	0.000458	0.045767
119	11.485	0.11	E	0.0097	0.000457	0.045731
120	11.491	0.122	E	0.009677	0.000456	0.04562
121	11.495	0.128	E	0.009661	0.000455	0.045546
122	11.517	0.114	E	0.009576	0.000451	0.045144
123	11.52	0.116	E	0.009564	0.000451	0.045089
124	11.576	0.15	E	0.009351	0.000441	0.044081
125	11.579	0.128	E	0.009339	0.00044	0.044028
126	11.698	0.18	E	0.008901	0.00042	0.041963
127	11.699	0.173	E	0.008898	0.000419	0.041946
128	11.723	0.08	E	0.008812	0.000415	0.041542
129	11.724	0.162	E	0.008808	0.000415	0.041525
130	11.775	0.126	E	0.008629	0.000407	0.040679
131	11.779	0.142	E	0.008615	0.000406	0.040614
132	11.799	0.129	E	0.008546	0.000403	0.040287
133	11.8	0.116	E	0.008542	0.000403	0.040271

134	12.704	0.169	Z	0.005931	0.00028	0.027959
135	12.801	0.136	Z	0.005703	0.000269	0.026886
136	12.864	0.189	Z	0.00556	0.000262	0.026211
137	12.872	0.152	Z	0.005542	0.000261	0.026126
138	13.944	0.103	Z	0.003595	0.000169	0.01695
139	13.977	0.124	Z	0.003548	0.000167	0.016726
140	14.049	0.165	Z	0.003446	0.000162	0.016247
141	14.061	0.099	Z	0.00343	0.000162	0.016168
142	14.283	0.137	Z	0.003136	0.000148	0.014782
143	14.35	0.157	Z	0.003052	0.000144	0.014388
144	14.36	0.125	Z	0.00304	0.000143	0.01433
145	14.447	0.156	Z	0.002935	0.000138	0.013836
146	14.529	0.108	Z	0.002839	0.000134	0.013385
147	14.536	0.126	Z	0.002831	0.000133	0.013347
148	14.603	0.181	Z	0.002756	0.00013	0.012991
149	14.61	0.103	Z	0.002748	0.00013	0.012955
150	14.619	0.096	Z	0.002738	0.000129	0.012908
151	14.621	0.173	Z	0.002736	0.000129	0.012897
152	14.628	0.123	Z	0.002728	0.000129	0.012861
153	14.634	0.133	Z	0.002721	0.000128	0.01283
154	14.717	0.157	Z	0.002632	0.000124	0.012407
155	14.719	0.089	Z	0.00263	0.000124	0.012397
156	14.75	0.157	Z	0.002597	0.000122	0.012243
157	14.752	0.193	Z	0.002595	0.000122	0.012233
158	14.755	0.165	Z	0.002592	0.000122	0.012218
159	14.756	0.115	Z	0.002591	0.000122	0.012213
160	14.775	0.131	Z	0.002571	0.000121	0.01212
161	14.788	0.185	Z	0.002557	0.000121	0.012057
162	14.836	0.117	Z	0.002508	0.000118	0.011825
163	14.841	0.095	Z	0.002503	0.000118	0.011801
164	14.842	0.137	Z	0.002502	0.000118	0.011797
165	14.865	0.1	Z	0.002479	0.000117	0.011688
166	14.867	0.11	Z	0.002477	0.000117	0.011678
167	14.88	0.128	Z	0.002464	0.000116	0.011617

168	14.951	0.138	Z	0.002395	0.000113	0.011289
169	15.012	0.128	Z	0.002336	0.00011	0.011014
170	15.053	0.127	Z	0.002298	0.000108	0.010834
171	15.055	0.107	Z	0.002296	0.000108	0.010825
172	15.181	0.131	Z	0.002182	0.000103	0.010288
173	15.182	0.115	Z	0.002181	0.000103	0.010284
174	15.624	0.18	Z	0.001825	8.6E-05	0.008604
175	15.624	0.113	Z	0.001825	8.6E-05	0.008604
176	15.74	0.124	Z	0.001742	8.21E-05	0.00821
177	15.742	0.139	Z	0.00174	8.2E-05	0.008203
178	17.04	0.176	Z	0.001031	4.86E-05	0.004858
179	17.062	0.121	Z	0.001021	4.82E-05	0.004815
180	17.416	0.151	Z	0.000885	4.17E-05	0.004174
181	17.542	0.151	Z	0.000842	3.97E-05	0.003967
182	19.797	0.143	Z	0.000339	1.6E-05	0.001597
183	19.799	0.12	Z	0.000338	1.6E-05	0.001595
184	19.944	0.123	Z	0.000319	1.5E-05	0.001505
185	19.95	0.112	Z	0.000318	1.5E-05	0.001501
186	20.361	0.087	Z	0.00027	1.27E-05	0.001272
187	20.364	0.193	Z	0.000269	1.27E-05	0.00127
188	20.489	0.164	Z	0.000256	1.21E-05	0.001208
189	20.494	0.184	Z	0.000256	1.21E-05	0.001205
190	20.495	0.18	Z	0.000256	1.2E-05	0.001205
191	20.537	0.16	Z	0.000251	1.18E-05	0.001184
192	20.545	0.101	Z	0.00025	1.18E-05	0.001181
193	20.552	0.11	Z	0.00025	1.18E-05	0.001177
194	20.586	0.114	Z	0.000246	1.16E-05	0.001161

Table S3: Conformational search of compound 32

Rank	ΔU kJ/mol	RMS	Diasteromer	w_i	p_i	p_i (%)
1	0	0.115	E	1	0.038448	3.84477
2	0.001	0.113	E	0.999596	0.038432	3.843218
3	0.113	0.155	E	0.955415	0.036734	3.673352
4	0.115	0.126	E	0.954644	0.036704	3.670388
5	0.53	0.156	E	0.807414	0.031043	3.10432
6	0.535	0.137	E	0.805786	0.030981	3.098061
7	0.62	0.112	E	0.77861	0.029936	2.993576
8	0.621	0.162	E	0.778296	0.029924	2.992368
9	2.04	0.14	E	0.438943	0.016876	1.687633
10	2.041	0.097	E	0.438765	0.01687	1.686952
11	2.122	0.083	Z	0.424653	0.016327	1.632692
12	2.13	0.16	Z	0.423284	0.016274	1.627428
13	2.141	0.159	E	0.421409	0.016202	1.620219
14	2.144	0.147	E	0.420899	0.016183	1.618258
15	2.23	0.145	Z	0.406539	0.01563	1.56305
16	2.243	0.128	Z	0.404412	0.015549	1.55487
17	2.265	0.108	Z	0.400837	0.015411	1.541124
18	2.294	0.145	Z	0.396172	0.015232	1.523191
19	2.354	0.138	Z	0.386693	0.014867	1.486746
20	2.389	0.123	Z	0.381269	0.014659	1.465891
21	2.474	0.154	Z	0.36841	0.014165	1.416452
22	2.478	0.13	Z	0.367816	0.014142	1.414167
23	2.594	0.192	E	0.350992	0.013495	1.349482
24	2.597	0.122	E	0.350567	0.013478	1.347849
25	2.671	0.177	E	0.340251	0.013082	1.308187
26	2.672	0.16	E	0.340114	0.013077	1.307659
27	2.675	0.143	Z	0.339702	0.013061	1.306077
28	2.678	0.162	Z	0.339291	0.013045	1.304496
29	2.831	0.174	E	0.318972	0.012264	1.226375
30	2.839	0.135	E	0.317944	0.012224	1.222422
31	2.925	0.107	Z	0.307097	0.011807	1.180718

32	2.927	0.125	Z	0.306849	0.011798	1.179765
33	3	0.164	E	0.29794	0.011455	1.145511
34	3.002	0.186	E	0.2977	0.011446	1.144587
35	3.024	0.165	E	0.295068	0.011345	1.134468
36	3.035	0.132	E	0.293761	0.011294	1.129442
37	3.126	0.097	Z	0.283167	0.010887	1.088711
38	3.129	0.188	Z	0.282824	0.010874	1.087394
39	3.23	0.157	E	0.271526	0.01044	1.043957
40	3.234	0.111	E	0.271088	0.010423	1.042273
41	3.906	0.125	E	0.206688	0.007947	0.794669
42	3.913	0.115	E	0.206105	0.007924	0.792427
43	3.961	0.12	E	0.202151	0.007772	0.777222
44	3.962	0.09	E	0.202069	0.007769	0.776909
45	4.068	0.117	E	0.193606	0.007444	0.744371
46	4.075	0.161	E	0.19306	0.007423	0.74227
47	4.077	0.121	E	0.192904	0.007417	0.741671
48	4.079	0.066	E	0.192748	0.007411	0.741073
49	4.149	0.126	E	0.187379	0.007204	0.720428
50	4.15	0.118	E	0.187303	0.007201	0.720137
51	4.268	0.146	E	0.178591	0.006866	0.686643
52	4.269	0.113	E	0.178519	0.006864	0.686366
53	4.278	0.133	E	0.177872	0.006839	0.683877
54	4.287	0.172	E	0.177227	0.006814	0.681398
55	4.347	0.147	E	0.172987	0.006651	0.665094
56	4.35	0.148	E	0.172777	0.006643	0.664289
57	4.449	0.122	E	0.16601	0.006383	0.638269
58	4.454	0.116	E	0.165675	0.00637	0.636982
59	4.486	0.175	E	0.163549	0.006288	0.628808
60	4.487	0.151	E	0.163483	0.006286	0.628554
61	4.513	0.127	E	0.161776	0.00622	0.621992
62	4.528	0.105	E	0.1608	0.006182	0.618238
63	4.529	0.108	E	0.160735	0.00618	0.617988
64	4.538	0.168	E	0.160152	0.006157	0.615748
65	4.706	0.137	E	0.149652	0.005754	0.575379

66	4.712	0.144	E	0.14929	0.00574	0.573987
67	4.819	0.137	E	0.14298	0.005497	0.549726
68	4.82	0.131	E	0.142922	0.005495	0.549504
69	5.26	0.098	E	0.119667	0.004601	0.46009
70	5.263	0.188	E	0.119522	0.004595	0.459534
71	5.342	0.118	E	0.115771	0.004451	0.445112
72	5.343	0.156	E	0.115724	0.004449	0.444932
73	5.73	0.127	Z	0.098989	0.003806	0.380589
74	5.748	0.103	Z	0.098272	0.003778	0.377834
75	5.878	0.131	E	0.093249	0.003585	0.35852
76	5.878	0.131	E	0.093249	0.003585	0.35852
77	5.925	0.113	Z	0.091497	0.003518	0.351783
78	5.925	0.118	E	0.091497	0.003518	0.351783
79	5.926	0.158	E	0.09146	0.003516	0.351641
80	6.009	0.103	Z	0.088446	0.003401	0.340056
81	6.133	0.133	E	0.084129	0.003235	0.323456
82	6.133	0.137	E	0.084129	0.003235	0.323456
83	6.21	0.093	E	0.081554	0.003136	0.313558
84	6.215	0.149	E	0.08139	0.003129	0.312925
85	7.903	0.117	Z	0.041179	0.001583	0.158325
86	8.038	0.108	Z	0.038996	0.001499	0.149929
87	8.43	0.157	Z	0.033289	0.00128	0.127989
88	8.555	0.117	Z	0.031651	0.001217	0.121692
89	9.03	0.133	E	0.026129	0.001005	0.100461
90	9.04	0.129	E	0.026024	0.001001	0.100057
91	9.147	0.079	E	0.024924	0.000958	0.095827
92	9.149	0.119	E	0.024904	0.000958	0.09575
93	9.547	0.126	Z	0.021208	0.000815	0.08154
94	9.567	0.144	Z	0.021038	0.000809	0.080885
95	9.569	0.097	E	0.021021	0.000808	0.08082
96	9.577	0.114	E	0.020953	0.000806	0.080559
97	9.66	0.122	E	0.020263	0.000779	0.077905
98	9.661	0.114	E	0.020254	0.000779	0.077874
99	9.886	0.136	Z	0.018496	0.000711	0.071113

100	9.93	0.149	Z	0.01817	0.000699	0.069861
101	9.948	0.134	Z	0.018039	0.000694	0.069356
102	9.951	0.15	Z	0.018017	0.000693	0.069272
103	10.103	0.142	E	0.016945	0.000651	0.06515
104	10.117	0.14	E	0.01685	0.000648	0.064783
105	10.208	0.154	E	0.016242	0.000624	0.062446
106	10.22	0.081	E	0.016163	0.000621	0.062145
107	10.276	0.145	Z	0.015802	0.000608	0.060756
108	10.302	0.18	Z	0.015637	0.000601	0.060121
109	10.503	0.116	E	0.014419	0.000554	0.055436
110	10.507	0.161	E	0.014395	0.000553	0.055347
111	10.519	0.116	E	0.014326	0.000551	0.05508
112	10.522	0.159	E	0.014309	0.00055	0.055013
113	10.594	0.12	E	0.013899	0.000534	0.053437
114	10.599	0.14	Z	0.013871	0.000533	0.05333
115	10.607	0.126	E	0.013826	0.000532	0.053158
116	10.609	0.162	E	0.013815	0.000531	0.053115
117	10.61	0.115	E	0.013809	0.000531	0.053093
118	10.619	0.149	E	0.013759	0.000529	0.052901
119	10.62	0.108	E	0.013754	0.000529	0.052879
120	10.636	0.138	Z	0.013665	0.000525	0.052539
121	10.649	0.165	Z	0.013594	0.000523	0.052264
122	10.678	0.134	Z	0.013435	0.000517	0.051656
123	10.683	0.179	Z	0.013408	0.000516	0.051552
124	10.684	0.151	E	0.013403	0.000515	0.051531
125	10.687	0.179	Z	0.013387	0.000515	0.051469
126	10.692	0.118	E	0.01336	0.000514	0.051365
127	10.706	0.164	Z	0.013284	0.000511	0.051075
128	10.734	0.127	Z	0.013135	0.000505	0.050501
129	10.746	0.111	E	0.013072	0.000503	0.050257
130	10.748	0.115	E	0.013061	0.000502	0.050217
131	10.76	0.162	E	0.012998	0.0005	0.049974
132	10.763	0.161	E	0.012982	0.000499	0.049914
133	10.8	0.119	E	0.01279	0.000492	0.049174

134	10.814	0.119	E	0.012718	0.000489	0.048897
135	10.823	0.109	Z	0.012672	0.000487	0.048719
136	10.827	0.133	Z	0.012651	0.000486	0.048641
137	10.85	0.088	E	0.012534	0.000482	0.048191
138	10.853	0.09	E	0.012519	0.000481	0.048133
139	10.908	0.107	Z	0.012244	0.000471	0.047076
140	10.998	0.191	Z	0.011807	0.000454	0.045397
141	11.014	0.169	Z	0.011731	0.000451	0.045105
142	11.107	0.099	Z	0.011299	0.000434	0.043443
143	11.152	0.141	Z	0.011096	0.000427	0.042661
144	11.244	0.119	Z	0.010691	0.000411	0.041106
145	11.286	0.123	Z	0.010512	0.000404	0.040415
146	11.371	0.113	Z	0.010157	0.000391	0.039052
147	11.416	0.143	Z	0.009974	0.000383	0.038349
148	11.497	0.14	Z	0.009654	0.000371	0.037116
149	11.575	0.138	Z	0.009354	0.00036	0.035965
150	11.58	0.105	Z	0.009336	0.000359	0.035893
151	11.73	0.184	Z	0.008787	0.000338	0.033784
152	11.734	0.162	Z	0.008773	0.000337	0.03373
153	11.792	0.134	Z	0.00857	0.000329	0.032949
154	12.879	0.124	Z	0.005526	0.000212	0.021247
155	13.014	0.099	Z	0.005233	0.000201	0.020121
156	13.308	0.11	Z	0.004648	0.000179	0.017869
157	13.313	0.152	Z	0.004638	0.000178	0.017833
158	13.39	0.234	Z	0.004496	0.000173	0.017288
159	13.5	0.175	Z	0.004301	0.000165	0.016537
160	13.519	0.157	Z	0.004268	0.000164	0.01641
161	13.558	0.163	Z	0.004202	0.000162	0.016154
162	13.561	0.137	Z	0.004197	0.000161	0.016135
163	13.655	0.138	Z	0.00404	0.000155	0.015534
164	14.335	0.106	Z	0.003071	0.000118	0.011805
165	14.336	0.106	Z	0.003069	0.000118	0.011801
166	14.495	0.107	Z	0.002879	0.000111	0.011067
167	14.496	0.157	Z	0.002877	0.000111	0.011063

168	14.916	0.103	Z	0.002429	9.34E-05	0.009338
169	14.923	0.119	Z	0.002422	9.31E-05	0.009311
170	15.019	0.147	Z	0.00233	8.96E-05	0.008957
171	15.021	0.104	Z	0.002328	8.95E-05	0.00895
172	17.645	0.106	E	0.000807	3.1E-05	0.003104
173	17.65	0.165	E	0.000806	3.1E-05	0.003097
174	17.671	0.153	E	0.000799	3.07E-05	0.003071
175	17.689	0.145	E	0.000793	3.05E-05	0.003049
176	17.751	0.118	E	0.000773	2.97E-05	0.002974
177	17.751	0.101	E	0.000773	2.97E-05	0.002974
178	17.756	0.132	E	0.000772	2.97E-05	0.002968
179	17.778	0.142	E	0.000765	2.94E-05	0.002941
180	17.807	0.175	E	0.000756	2.91E-05	0.002907
181	17.858	0.113	E	0.000741	2.85E-05	0.002848
182	18.229	0.099	E	0.000638	2.45E-05	0.002452
183	18.239	0.11	E	0.000635	2.44E-05	0.002442
184	18.247	0.13	E	0.000633	2.43E-05	0.002434
185	18.301	0.156	E	0.000619	2.38E-05	0.002382
186	18.309	0.183	E	0.000617	2.37E-05	0.002374
187	18.316	0.169	E	0.000616	2.37E-05	0.002367
188	18.327	0.101	E	0.000613	2.36E-05	0.002357
189	18.336	0.158	E	0.000611	2.35E-05	0.002348
190	18.371	0.187	E	0.000602	2.32E-05	0.002315
191	18.41	0.149	E	0.000593	2.28E-05	0.002279
192	18.581	0.079	E	0.000553	2.13E-05	0.002127
193	18.595	0.152	E	0.00055	2.12E-05	0.002115
194	18.621	0.116	E	0.000544	2.09E-05	0.002093
195	18.635	0.138	E	0.000541	2.08E-05	0.002081
196	18.653	0.151	E	0.000537	2.07E-05	0.002066
197	18.697	0.131	E	0.000528	2.03E-05	0.00203
198	18.851	0.144	E	0.000496	1.91E-05	0.001908
199	18.858	0.169	E	0.000495	1.9E-05	0.001902
200	18.858	0.161	E	0.000495	1.9E-05	0.001902
201	18.866	0.129	E	0.000493	1.9E-05	0.001896

202	18.932	0.113	E	0.00048	1.85E-05	0.001846
203	18.932	0.199	E	0.00048	1.85E-05	0.001846
204	20.601	0.147	Z	0.000245	9.41E-06	0.000941
205	20.602	0.152	Z	0.000245	9.41E-06	0.000941
206	20.738	0.146	Z	0.000232	8.91E-06	0.000891
207	20.749	0.153	Z	0.000231	8.87E-06	0.000887

Table S4: Boltzmann population analysis summary and sensitivity analysis

Compound	f_E	Counts ΔU ≤ 3kJ	Counts ΔU ≤ 5kJ	f_E -1 kJ shift	f_E +1 kJ shift	f_E -2 kJ shift	f_E +2 kJ shift
**30**	0.870862	E: 20<br>Z: 0	E: 42<br>Z: 4	0.87086193820944	0.8708619382094399	0.8708619382094396	0.8708619382094394
**31**	0.856619946	E: 16<br>Z: 0	E: 48<br>Z: 12	0.8566199464008506	0.8566199464008508	0.8566199464008507	0.8566199464008508
**32**	0.736912572	E: 19<br>Z: 14	E: 52<br>Z: 16	0.7369125719871179	0.7369125719871179	0.7369125719871177	0.7369125719871179

The shift columns demonstrate how the E population fraction (f_E) would shift when all relative conformer energies ΔU are systematically offset by ±1 and ±2 kJ·mol^{-1}. The values remain effectively unchanged (<0.001 absolute difference).

Cumulative Boltzmann population plots

Conformers were ordered from lowest to highest ΔU (Tables S1 – S3). Then the cumulative populations were computed for each conformer in the sorted list, ie the cumulative sum of p_i was computed separately for E and *Z*. Example: at conformer rank *n*,

$$\mathrm{CumE}(n) = \frac{\sum_{i \le n, i \in E} w_i}{\sum_j w_j}, \mathrm{CumZ}(n) = \frac{\sum_{i \le n, i \in Z} w_i}{\sum_j w_j}$$

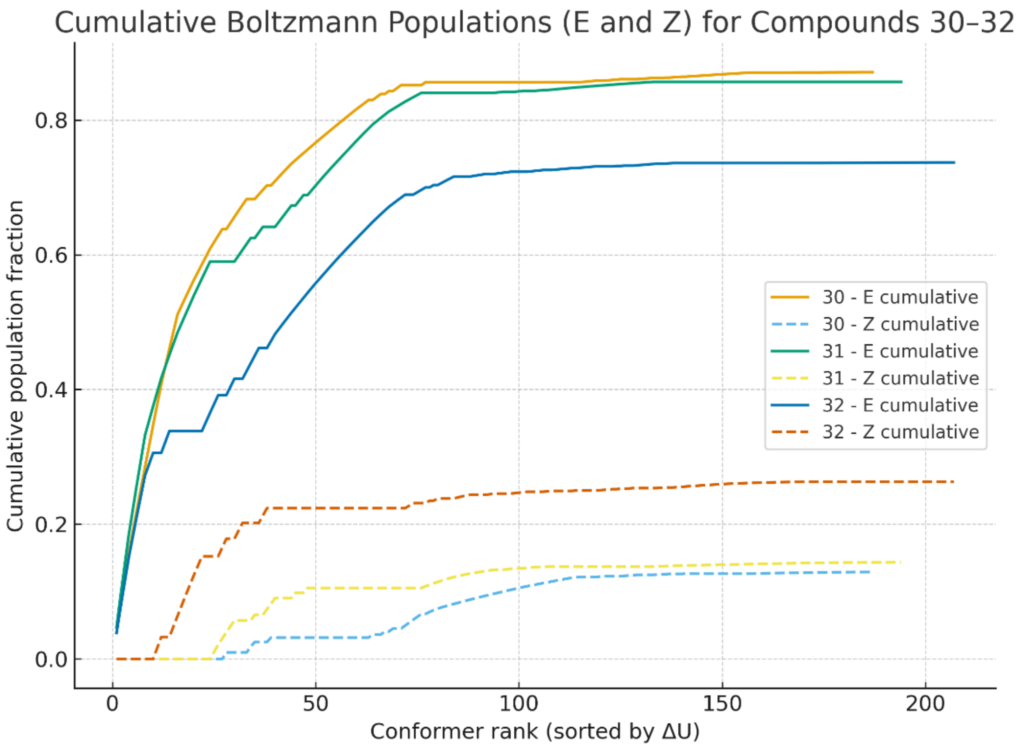

Fig. S4: The steepness of the E curve illustrates how quickly E dominates the population as conformers are added in order of energy. For **30** and **31**, the E curve rises sharply early on, showing that most low-energy conformers are E. For **32**, the Z curve contributes more within the first few kJ·mol^{-1}, so the E cumulative fraction rises more slowly → overall f_E is lower (~0.74).

Table S5: RMS statistics

Cmp	Isomer	count	mean	std	min	25%	50%	75%	max
**30**	E	102	0.133912	0.02629	0.063	0.117	0.129	0.14975	0.198
**30**	Z	85	0.1348	0.025695	0.081	0.119	0.137	0.148	0.198
**31**	E	96	0.133833333	0.030743991	0.069	0.1155	0.1295	0.155	0.224
**31**	Z	98	0.136285714	0.028665508	0.078	0.115	0.1315	0.157	0.21
**32**	E	128	0.134429688	0.026677627	0.066	0.11575	0.132	0.156	0.199
**32**	Z	79	0.136924051	0.026959025	0.083	0.115	0.138	0.1525	0.234

This is the output of the standard **descriptive statistics** for the RMS derivative values (σΔU proxies) across conformers of each diastereomer. Columns mean:

- **count**: number of conformers in that group (E or Z).
- **mean**: average RMS derivative across those conformers (typical optimization gradient magnitude).
- **std**: standard deviation of RMS values.
- **min**: lowest RMS in the group.
- **25% (Q1)**: first quartile (25% of conformers have RMS ≤ this).
- **50% (median)**: middle value of the distribution.
- **75% (Q3)**: third quartile (75% of conformers have RMS ≤ this).
- **max**: largest RMS in the group.
